# Combustion Resistant Borohydrides and Their Chemical Interactions with Li-Metal Surfaces: An Experimental and Theoretical Study

**DOI:** 10.1021/acscentsci.5c00043

**Published:** 2025-04-23

**Authors:** Anton W. Tomich, Stephen Proctor, Moon Young Yang, Jianjun Chen, Yifan Zhao, Edward Chen, Tridip Das, Boris V. Merinov, William A. Goddard, Juchen Guo, Vincent Lavallo

**Affiliations:** † Department of Chemistry, 8790 University of California Riverside, Riverside, California 92521, United States; ‡ Materials and Process Simulations Center, 6469 California Institute of Technology Pasadena, California 91125, United States; § Department of Chemical and Environmental Engineering, University of California Riverside, Riverside, California 92521, United States

## Abstract

Borohydrides are important molecular entities for a myriad of applications from organic synthesis to components of functional materials and devices. All borohydrides have been thought to be susceptible to spontaneous ignition when exposed to a flame. Herein we demonstrate that this is not always true by identifying several borohydride rich materials that are resistant to combustion when contacted with a torch. One of these materials is a Li^+^ salt of a carborane anion that depending on its coordination environment exists as a unique ionic liquid that has a nearly naked Li^+^ countercation. This has provided us with the first opportunity to spectroscopically probe the interactions of such carborane anions with Li metal in a solvent free environment. We found that this carborane anion is immune to deleterious reduction at Li-metal surfaces, as evidenced by XPS, EDS and SEM analysis of the Li-Metal surface after exposure to the ionic liquid. Additionally, NMR analysis of the ionic liquid after stirring it with Li powder shows no reaction. Calculations show that the cage skeleton is reduced at the surface monolayer, but as the reduced form is removed from contact with Li-metal, the cage reverts to the *closo*-form, demonstrating reversibility.

## Graphical abstract

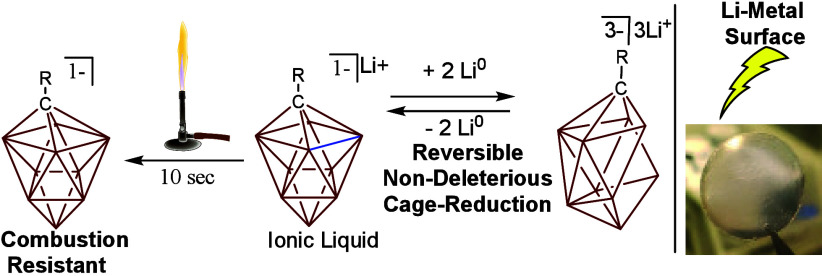

## 1 Introduction

In 1912, Alfred Stock reported the first characterization of borohydrides, such as B_4_H_10_, using high vacuum air free chemistry techniques.^1^ Such specialized glass manifolds were necessary to characterize these species, as all compounds in this original paper were pyrophoric when in contact with air. Most neutral borohydrides such as pentaborane (B_5_H_9_ and B_5_H_11_) are notoriously pyrophoric producing green flames upon combustion, due to the thermal excitation and emission from boron atoms.^2^ At the beginning of the cold war, the energy content and combustibility of these materials drew the attention of military powers and the petroleum industry as potential candidates for high energy rocket fuels and gasoline additives, respectively. Ultimately their application as high energy fuels were not effective because the extremely hard BO byproducts destroyed the rocket engines.^3–6^ All of the neutral borohydrides form via B–H aggregation reactions involving elimination of H_2_ from the lighter borohydrides to ultimately form the thermodynamic sink decaborane-14 (B_10_H_14_), which is not pyrophoric at room temperature, but highly combustible.^5^ Ionic borohydrides like BH_4_^1–^ are highly combustible,^7^ and all anionic carborane and boron cluster compounds are assumed to be equally combustible in air with an open flame.

Borohydrides and carboranes have found a myriad of applications in organic synthesis,^8,9^ medicine,^10,11^ polymeric materials,^12,13^ tools for nuclear waste remediation,^14^ ligand/catalyst design,^15–20^ chemical vapor deposition,^21^ the study of reactive cations,^22–26^ and most recently as materials for electrochemical energy storage.^27–33^ With respect to energy storage, while anionic icosahedral borohydrides have recently been employed as electrode materials, the most important breakthroughs have been in the use of isoelectronic *closo*-deltahedral carborane anion salts as electrolytes. Of course, for battery applications, it would be ideal to have electrolyte systems that are combustion resistant and contain no free flammable organic solvents.^34^ While there have been reports of interesting solid-state ion conductivity data with solid borohydrides and carboranes,^35–41^ there are no known room-temperature alkali-metal ionic liquids of borohydrides or carboranes.^42–44^ Since the transition from Li-ion to Li-metal batteries would constitute a major breakthrough in electrochemical energy storage, it is of great interest to study the chemical interactions of reductively stable and combustion resistant electrolytes at a Li-metal surface to understand the principles underlying such systems. Herein, we report a novel carborane salt that forms subzero ionic liquids when the Li^+^ countercation is coordinatively unsaturated by THF solvent molecules. Both the desolvated and sparingly solvated forms of this species demonstrate high resistance to combustion. On the other hand, we show that carborane and borohydride salts of K^+^ and Cs^+^ are all combustible when in contact with an open flame, which highlights the special role that Li^+^, and sometimes Na^+^, play in this unusual combustion resistance. Given the unprecedented nonreactivity of the ionic liquid, we investigate here the direct interactions of a pure carborane salt with metallic Li surfaces in the absence of free solvent. We demonstrate both experimentally and computationally that this material is immune to irreversible decomposition via chemical reduction by the Li metal. Calculations indicate that the mechanism at the Li-surface involves reversible reduction/cluster expansion forming a charge transfer complex with a soft potential energy surface that readily dissociates from the metal surface to reform the *closo*-structure unscathed.

## 2 Results

While designing advanced electrolytes for Mg-based batteries,^30,32^ we serendipitously discovered the Li^+^ carborane salt **LiC**_**10**_ **-THF**_**4**_ ([Li­(THF)_4_+]­[*n*-decyl-CB_9_H_9_1-]), which contains a ten carbon hydrocarbon tail fused to the C-vertex of the cluster anion and four THF molecules coordinated to the Li^+^ cation (Figure 1b). While compound **LiC**_**10**_ **-THF**_**4**_ could be obtained directly following alkylation of the dianionic [CB_9_H_9_]^2–^ species, a modified literature procedure was employed to achieve Li salts of alkylated carborane species in high purity (Figures 1a, S8–S10, S58–S84, S104–S117). Interestingly, freshly prepared **LiC**_**10**_ **-THF**_**4**_ exists as a room temperature ionic liquid with a melting point of −24.5 °C. However, this material is only metastable as it slowly crystallizes over time, presumably due to the loss of THF solvent coordinated to the Li^+^ cation. Further concentration of the material under high vacuum yields the low melting point solid **LiC**_**10**_ **-THF** (mp = 51.8 °C) with a single coordinated THF molecule (Figures S108–S113). Exhaustive desolvation under vacuum and heating to 195 °C results in the formation of the solvent free lithium salt, **LiC**_**10**_ (mp = 90.0 °C) (Figures S104–S107).

**Figure 1:**
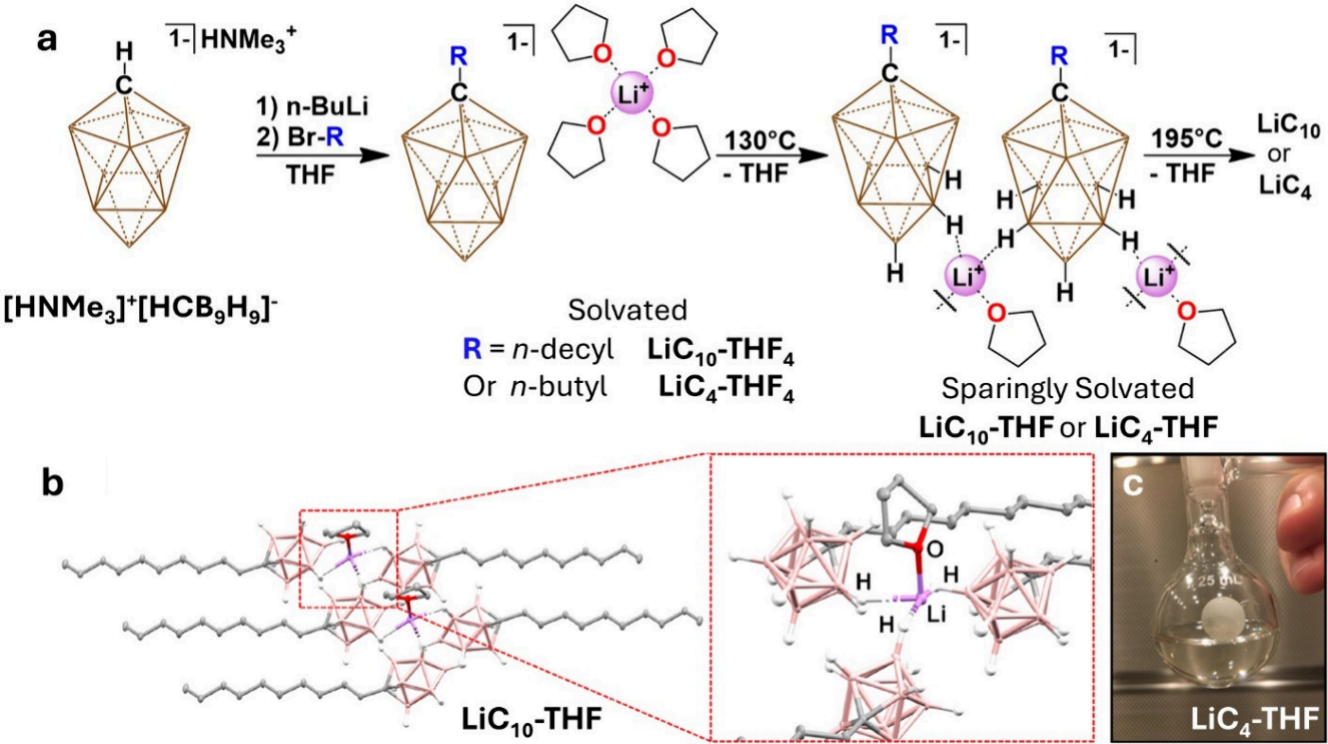
(a) Synthetic scheme detailing [HCB_9_H_9_^1–^] functionalization and desolvation procedure (brown, unlabeled vertices indicate B–H). (b) Solid-state structure of **LiC**_**10**_ **-THF** depicting distorted tetrahedral coordination environment of Li^+^ (Boron, pink; Carbon, gray; Oxygen, red; Lithium, purple; Hydrogen, white. Hydrogen omitted from alkyl moiety for clarity.) (c) Photo of room temperature ionic liquid **LiC**_**4**_ **-THF**.

The low melting points for these Li^+^ carborane salts are extremely unusual, if not unprecedented, which piqued our interest toward the possibility of chemically modifying the cage to form a phase-stable room temperature Li^+^ ionic liquid. Such an unusual compound could have potential applications in Li^+^ ion catalysis,^45^ ion thrusters for spacecraft,^46^ and as an electrolyte in electrochemical energy storage devices.^47^ Hence, we proceeded to utilize our expertise in carborane surface modifications to engineer a material with a lower liquidus range and resistance to spontaneous crystallization. We hypothesized that shortening the 10-carbon decyl-tail of the carborane would reduce the van der Waals (vdW) interactions between ions, discouraging THF loss, which might yield a phase stable RT ionic liquid. Hence, we prepared **LiC**_**4**_ **-THF**_**4**_ with a butyl hydrocarbon tail instead of a decyl chain (Figure 1a). We were initially disappointed that **LiC**_**4**_ **-THF**_**4**_ was isolable as a solid (mp = 38.2 °C) compared to **LiC**_**10**_ **-THF**_**4**_ (mp = −24.5 °C). However, we were delighted and surprised to find that when **LiC**_**4**_ **-THF**_**4**_ is partially desolvated to a material with only one coordinated THF molecule **LiC**_**4**_ **-THF**, a stable liquid material with a very low melting point (mp = −57 °C) is formed (Figure 1, **c**, S135). The fact that **LiC**_**10**_ **-THF** and the reengineered **LiC**_**4**_ **-THF** have an over 100 °C difference in melting points highlights the profound effects that surface modifications can have on the physical properties of these nanoclusters (Figure S133).

To gain further insight into the exact chemical structure of **LiC**_**4**_ **-THF** and **LiC**_**10**_ **-THF**, we performed multinuclear (^1^H, ^11^B, ^13^C, and ^7^Li) magnetic resonance (NMR) spectroscopy analysis and high-resolution mass spectrometry (Figures S69–S74, S108–S113, S136, S137). The NMR data confirm the formation of compounds containing a single THF molecule bound to Li^+^, a single Li^+^ species, and a single carborane cage that retains its local C_4v_ symmetry, which is consistent with our proposed formulation. Indeed, variable temperature ^11^B­{^1^H} NMR experiments of neat **LiC**_**4**_ **-THF** reveal this symmetry is maintained in subzero conditions until the coalescence of peaks near the compound’s melting point (Figures S134, 138). The fact that the ^11^B spectra maintains its symmetry implies that the interaction between the clusters and the Li^+^ cations are weak and the carborane and Li^+^ cation are freely mobile in this liquid. Otherwise, the formation of a strong contact ion pair would result in broken symmetry. While it is not surprising that we were not able to grow single crystals of **LiC**_**4**_ **-THF** given its extremely low melting point, we were able to analyze its cousin **LiC**_**10**_ **-THF** via single crystal diffraction (Figure S140). In the solid state, **LiC**_**10**_ **-THF** forms an extended network of Li^+^ cations in distorted tetrahedral coordination environments. A portion of the crystal lattice is shown in Figure 1b. It highlights the solvent and anion/cation interactions, as well as the positioning of the hydrocarbon tail. Aside from the one coordinated THF molecule per Li^+^ cation, the other three vertices of the tetrahedron are occupied with B–H interactions arising from σ-complexation with three different carborane anions. We believe that this structure is a snapshot of one possible coordination environment in solution so that **LiC**_**4**_ **-THF** likely engages in analogous Li–H–B bonding. However, as indicated by ^11^B NMR, these interactions are dynamic and rapidly exchanging as each cluster tumbles in solution. While our inability to crystallize **LiC**_**4**_ **-THF** prohibited a single crystal diffraction study, we have determined the crystal structure computationally (vide infra, Figure S139).

We anticipated that **LiC**_**4**_ **-THF**, comprised primarily of a carborane anion functionalized with an extended hydrocarbon tail, would demonstrate combustion behavior similar to those of all other known borohydrides. Remarkably, flame tests undertaken with a sample of **LiC**_**4**_ **-THF** provide no evidence of combustion after 10 s of flame exposure (Figure 2b, and Movie S1) nor decomposition as indicated by NMR analysis (Figures S75–S77, S82–S84). However, after 20 s of exposure, the material is completely destroyed, leaving behind an insoluble film, presumably boron oxide. Interestingly, the completely desolvated solid form **LiC**_**4**_ displays similar combustion resistance (Figure 2a, S62–S68), indicating that this unusual property is not solely due to the fact that **C**_**4**_ **-THF** is a RT Ionic Liquid with low vapor pressure and low surface area (Figure S133). Despite full saturation of Li^+^, and formation of the solvent-separated ion pair **LiC**_**4**_ **-THF**_**4**_, there is similarly no visual combustion of the material (Figure 2c, and S79–S84).

**Figure 2:**
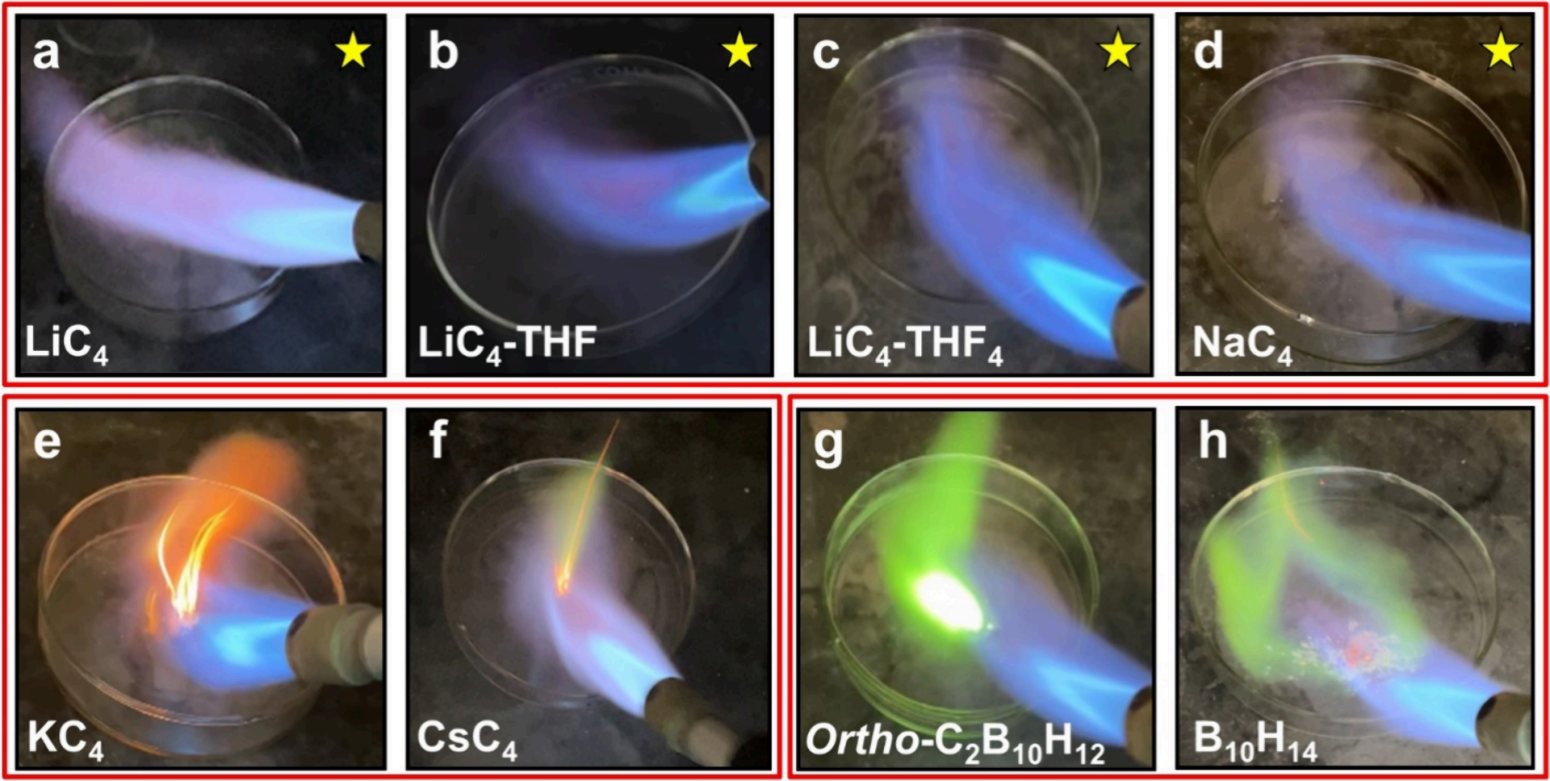
Combustion behavior of **C**_**4**_ species, alkali metal analogues, and borohydrides. (a-c) combustion resistant **LiC**_**4**_, **LiC**_**4**_ **-THF**, and **LiC**_**4**_ **-THF**_**4**_. (d-f) alkali metal cation analogues **NaC**_**4**_, **KC**_**4**_, and **CsC**_**4**_ (g, h) Charge neutral carborane *Ortho*-C_2_B_10_H_12_ and “decaborane”, B_10_H_14_. Note: Star indicates a lack of combustion behavior.

Due to this unexpected behavior, we sought to probe compositional factors that impart this unusual physical property. Variables to consider include the degree of cation solvation, the influence of the hydrocarbon tail, and the nature of the cation–anion interaction. We first looked at the [HCB_9_H_9_^1–^] parent cluster’s interactions with flame as it is absent the hydrocarbon tail key to achieving the liquid **LiC**_**4**_ **-THF** material. Interestingly, [Li­(THF)_*n*_^+^]­[HCB_9_H_9_^1–^] (*n* = 1, 4) displayed similar combustion resistance even though these solvates are room temperature solids (Figures S11–S14, S19–S31). However, the fully desolvated [Li^+^]­[HCB_9_H_9_^1–^] combusts immediately (Figures S15–17). We next investigated how the nature of the countercation affects the combustion resistance by preparing the Na^+^, K^+^, and Cs^+^ salts of the materials (Figures S1–S7, S32–S51, S52–S57, S85–S103). While the Na^+^ salts could be isolated as **NaC**_**4**_ and **NaC**_**4**_ **-THF**_**2**_, the K^+^ and Cs^+^ salts could only be isolated in fully desolvated forms **KC**_**4**_ and **CsC**_**4**_, likely due to the weaker metal ligand bonds for these heavier alkali cations. To summarize, all of these compounds are RT solids and neither **NaC**_**4**_ nor **NaC**_**4**_ **-THF**_**2**_ ignite when contacted with a flame (Figure 2d). In stark contrast **KC**_**4**_ and **CsC**_**4**_ instantly ignite when contacted with flame (Figure 2e,f). In contrast, the facile combustion of other carborane clusters (o-carborane) and borohydrides (decaborane-14) is apparent (Figure 2g,h). Additionally, we confirmed that [Na^+^]­[HCB_9_H_9_^1–^], [Na­(THF)_2_^+^]­[HCB_9_H_9_^1–^], [K^+^]­[HCB_9_H_9_^1–^] [Cs^+^]­[HCB_9_H_9_^1–^], BH_3_–NEt_3_, LiBH_4_ all ignite when exposed to a Bunsen burner (Figure S132). Furthermore, the isoelectronic dianionic lithium borohydride cluster salts [2Li^+^]­[B_10_H_10_^2–^] and [2Li­(THF)_2_^+^]­[B_10_H_10_^2–^] ignite instantly on contact with a flame (Figures S118–132).

Considering the extreme combustion resistance of ionic liquid **LiC**_**4**_ **-THF**, we undertook a more quantitative investigation of the thermal stability of **LiC**_**4**_ **-THF** via thermal gravimetric analysis (TGA). Gradual mass loss was observed upon heating to 150 °C until a plateau corresponding to 75% of the original mass was maintained beyond 300 °C (Figure 3a). The calculated mass contribution of [Li^+^]­[C_4_H_9_CB_9_H_9_^1–^] to **LiC**_**4**_ **-THF** (∼72%) agrees well with the actual (75%) value, which is slightly higher than expected. The ∼3% difference in mass is likely due to a small quantity of adventitious moisture obtained during transfer of the sample to the instrument. To characterize the solvation environment of Li^+^ in the presence of 1 eq. of THF, we employed Raman spectroscopy to compare **LiC**_**4**_ **-THF** to diluted solutions of the salt which demonstrate that no free THF is present in the ionic liquid (Figure 3b). The diminished THF peak exhibits a distinct 15 cm^–1^ shift to higher frequencies, indicating a solution comprised solely of the Li^+^-THF solvation complex and lacking a free-solvent signature. Encouraged by the unusual combustion resistance of **LiC**_**4**_ **-THF** and an ionic liquid composed primarily of a material renowned for its chemically inert nature, we envisioned a unique opportunity to investigate the reactivity of this material with Li-metal. Recently, it has been reported, albeit with zero spectroscopic evidence and only electrochemical measurements, solid solutions of [Li^+^]­[HCB_9_H_9_^1–^] and its larger cousin [Li^+^]­[HCB_11_H_11_^1–^] are stable in contact with this material.^40^ Typical Li^+^ salts utilized in battery electrolytes are composed of weakly coordinating anions such as LiPF_6_ and LiTFSI. These species have been long been understood to decompose and contribute to a solid-electrolyte interphase (SEI) when in contact with Li-metal surfaces.^48^ Since **LiC**_**4**_ **-THF** lacks free solvent available to quickly undergo reductive decomposition at the Li metal surface, we became curious if the SEI would be comprised primarily from anion decomposition. To explore the reductive stability of **LiC**_**4**_ **-THF**, lithium metal foil was soaked in the solution for 1 month anticipating that minimal chemical reactivity would be observed. Indeed, the Li-metal soaked in **LiC**_**4**_ **-THF** yielded no corrosion of the foil surface, as evidenced by electron microscopy (Figure 3c). Suspecting that the relatively low surface area of the foil was not sufficient to observe reactivity with the ionic liquid, we stirred **LiC**_**4**_ **-THF** on Li metal powder in an effort to observe reduction of the species. Indeed, no evidence of carborane cage decomposition was revealed by ^11^B NMR analysis of the ionic liquid after 1 month (Figure 3d).

**Figure 3:**
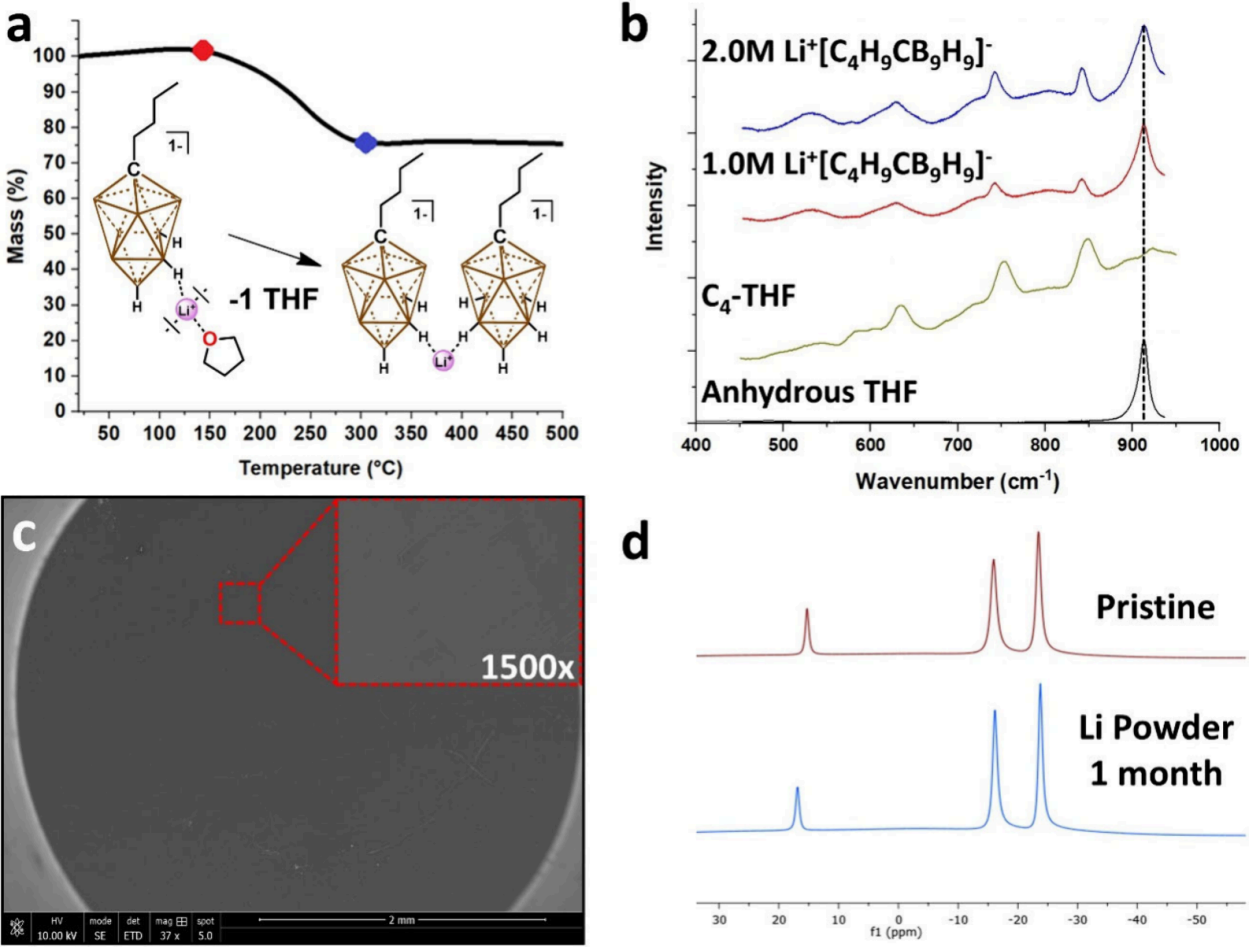
(a) Thermal gravimetric analysis demonstrating thermal stability of **LiC**_**4**_ **-THF** (red) and mass attributed to lithium salt content (blue). (b) Raman spectra of varying concentrations of [Li^+^]­[C_4_H_9_CB_9_H_9_^1–^] in THF, **LiC**_**4**_ **-THF**, and THF. (c) SEM image of Li foil after soaking in **LiC**_**4**_ **-THF**, (inset) 1500× magnification of Li foil surface and (d) ^11^B­{^1^H} NMR spectra of pristine **LiC**_**4**_ **-THF** material (red) and **LiC**_**4**_ **-THF** stirred on Li metal powder (blue).

## 3 Discussion

To gain an understanding of the resulting surface composition, energy dispersive x-ray spectroscopy (EDS) was employed for elemental mapping the Li metal surface after the 1 month soak in **LiC**_**4**_ **-THF** (Figure 4a-d). The resulting chemical reduction of the ionic liquid yields a surface chemical morphology largely composed of oxygen and carbon containing species, suggesting the SEI is derived primarily from THF and adventitious oxygen. Despite efforts to wash the Li metal surface of excess **LiC**_**4**_ **-THF**, a small quantity of boron remained. In prior investigations, we have demonstrated that [HCB_11_H_11_^1–^] salts and their alkyl-functionalized derivatives exhibit reductive stability in the presence of Na and Mg metal, respectively.^30,31^ Given the highly reducing nature of Li metal (−3.04 V vs SHE), we sought to further characterize the persistent surface species. X-ray Photoelectron Spectroscopy (XPS) was employed to identify specific binding energies associated with chemical bonding of species present at the surface (Figure 4e-h). We identified C–C, C–O, C=O, and Li–O binding energies corroborating the presence of THF derived species observed in the EDS mapping. Peaks in the B 1s region at 187 and 188 eV corresponding to the presence of B–B and C–B bonding, respectively, matching previously reported prior XPS studies, suggesting that cluster integrity is maintained even in highly reducing environments.^30,31^ Prior to this study, no weakly coordinating anion has been reported to exhibit absolute reductive stability in the presence of Li metal.

**Figure 4:**
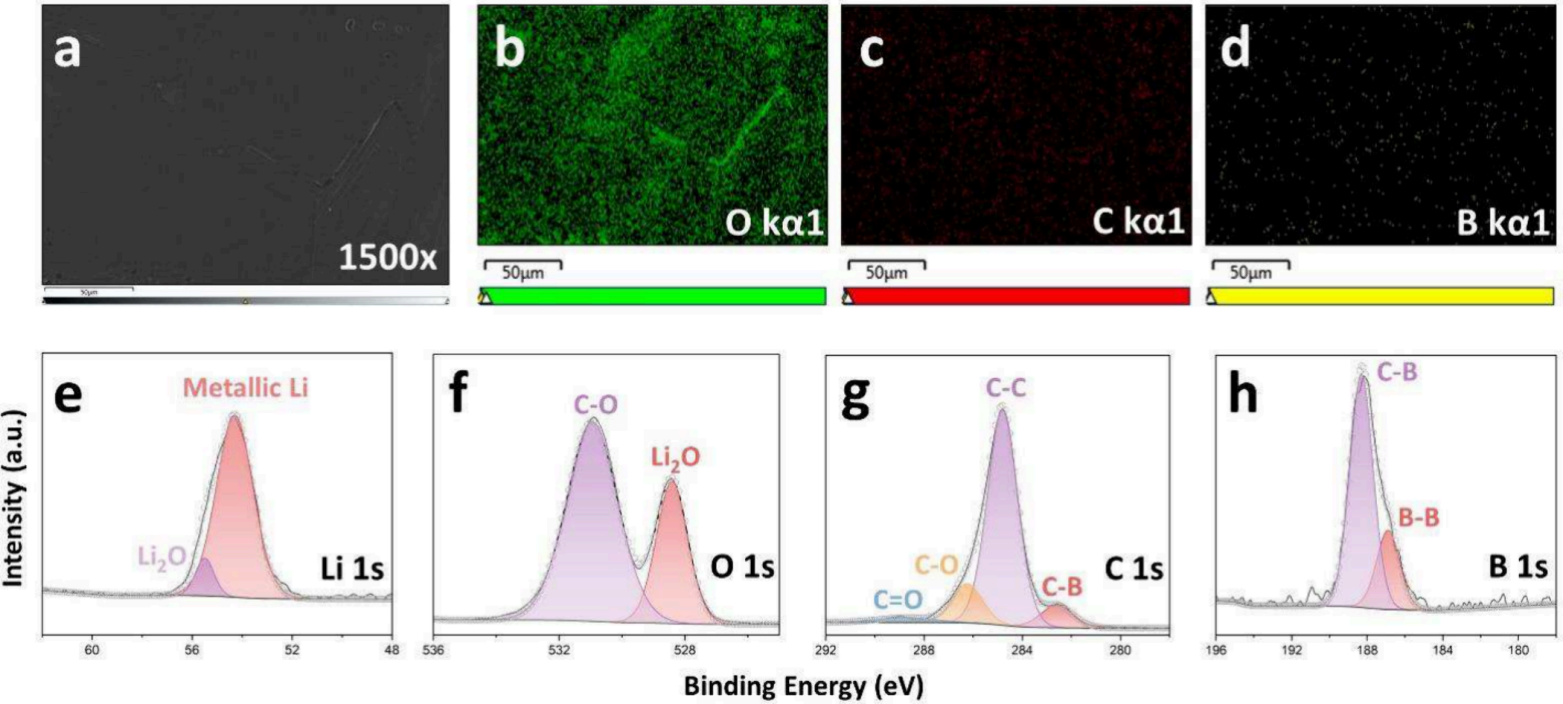
Surface characterization of Li metal foil soaked in **LiC**_**4**_ **-THF** for 1 week. (a) SEM image of Li metal surface at 1500× magnification. (b–d) Corresponding EDS elemental mapping of Li metal surface. (e–h) XPS measurement of Li metal surface.

To provide a better understanding of the unique stability of this anion against Li-metal, we performed ab initio molecular dynamics (AIMD) simulations using an interface model of the Li-metal and **LiC**_**4**_ **-THF** for 100 ps at 300 K (Figure 5a). Interestingly, the carborane molecules (*closo*-) in contact with the Li electrode at the interface quickly incorporate one or two Li^+^ ions to form the reduced (*arachno*-) cluster in the simulation. However, these reduced carborane molecules were stable, with no further cage rupturing during the 100 ps MD simulation. The reduction of the carborane molecules at the surface involves opening the carborane cage to include one or two Li^+^ ions in the expanded cluster at the interface.

**Figure 5:**
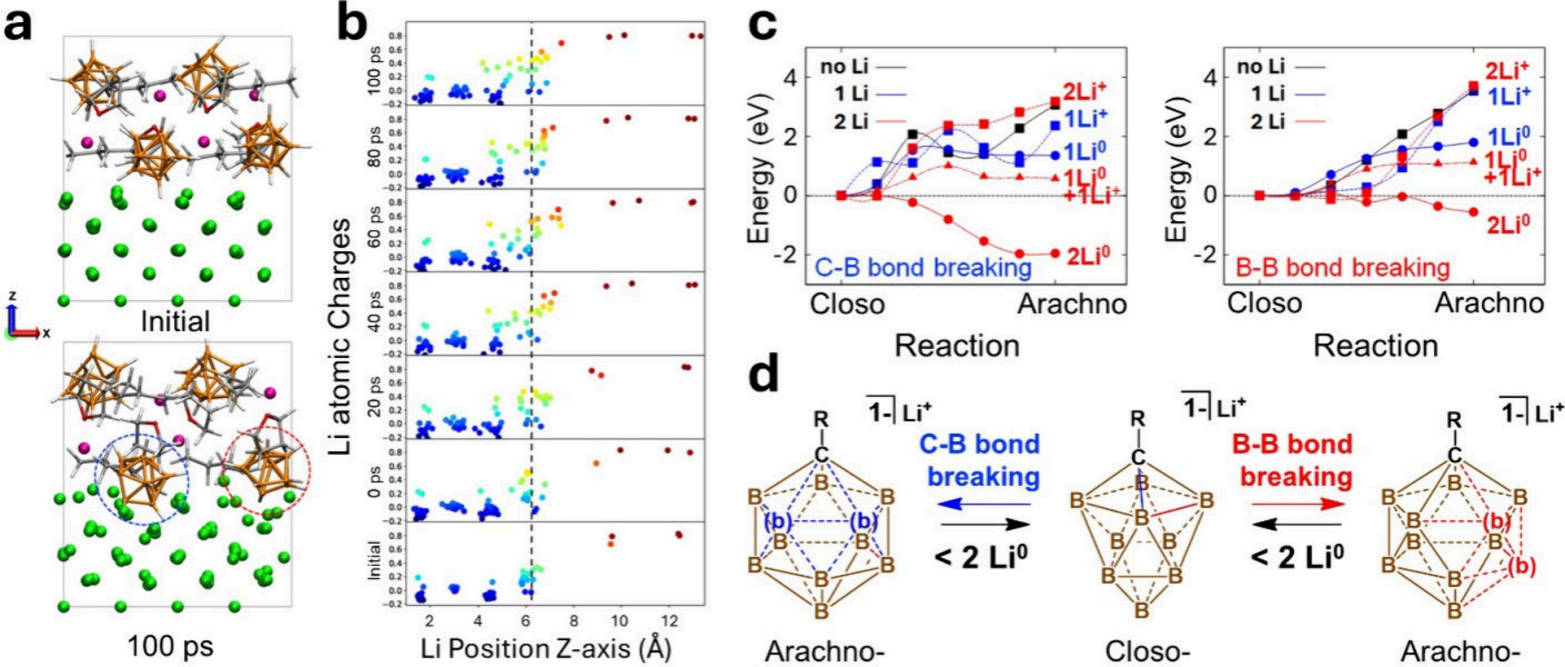
Results from DFT calculations. (a) Snapshots of structures before (top) and after (bottom) the 100 ps AIMD simulation. Gray, orange, red, white, magenta, and green indicate carbon, boron, oxygen, hydrogen, Li (in **LiC**_**4**_ **-THF**), and Li metal, respectively. (b) Atomic charge analysis for Li along the axis perpendicular to the surface, with the atomic charges indicated by the color (neutral: blue; +1: red) every 20 ps of the trajectory (0 ps at the bottom, 100 ps at the top). (c) Energy profiles of the carborane cage opening, depending on the number and charge state of Li. (d) Schematic figure representing the reversible cage opening reaction of the carborane molecule (“b” denotes the icosahedral site occupied by Li^+^ following reduction). The C–B bond and B–B bond broken carborane molecules after the AIMD simulation are indicated by blue and red circles in (a).

Figure 5b shows the predicted net atomic charges (NACs) for Li as a function of Li positions from every 20 ps structures of the trajectory up to 100 ps. We find that Li atoms within the Li metal slab are neutral (net charge of ±0.2), whereas Li atoms within the cage at the first monolayer of the liquid LiC_4_-THF phase have NAC ∼ +0.8. That is, as Li goes to the first monolayer of Li–ionic liquid interface and incorprates into the *closo*- cluster to form the *arachno*- cluster of the carborane with an additional one or two Li^+^ ions, the NAC increased to ∼ + 0.8. We further find that this *closo-arachno* transition is reversible. Figure 5c shows the energy profiles along the transition between *closo*-carborane and *arachno*-carborane by the number and charge state of Li. Our results show that the modified *closo*-carborane, **LiC**_**4**_ derived species, becomes *arachno*-carborane as one or two Li atoms are incorporated as Li^+^ into the cluster (Li^+^ occupation denoted by “b”). However, this *arachno-expanded* carborane returns to *closo*-carborane as the cluster migrates to the second monolayer, with the complexed Li removed, indicating that this *closo-arachno* transition is reversible depending on the Li environment nearby the carborane (Figure 5d). Taken together, this Li-associated reversible transition of carborane without decomposition explains the chemical stability of this anion against the Li metal.

## 4 Conclusions

Over a hundred years after Stock’s seminal discovery of pyrophoric borohydrides, we show that it is possible to prepare borohydride-rich materials that do not spontaneously ignite in contact with an open flame. This is important because many next-generation technologies will benefit from the properties we describe above, and it also highlights the extreme chemical stability of certain carborane anions. For example, we speculate the ionic liquid **LiC**_**4**_ **-THF** may be employed as a safe alternative electrolyte for Li-ion batteries and even new high energy density electrochemical cells based on Li-metal. The novel mechanism of reversible cage reduction at the metal surface is also fundamentally interesting and perhaps gives us a glimpse into new possible functionalization strategies to tune the cage properties. Efforts to address this effect are currently ongoing.

## 5 Methods

All essential experimental and computational procedures and data are available in the attached Supporting Information.

## Supplementary Material









## Notes

Additional experimental details, materials, and methods including detailed synthetic information, accompanying multinuclear NMR (^11^B, ^1^H, ^13^C, ^7^Li, ^23^Na), theoretical calculations, and crystal structure refinement (PDF)
X-ray data (CIF)
Transparent Peer Review report available (PDF)

## Notes

Movie showing flame tests undertaken with a sample of **LiC**_**4**_ **-THF**.
