# Supplementary material for: Combustion Resistant Borohydrides and Their Chemical Interactions with Li-Metal Surfaces: An Experimental and Theoretical Study
Source: ACS Cent Sci. 2025 Apr 23;11(5):734–41. doi: 10.1021/acscentsci.5c00043 (PMC12164935; doi:10.1021/acscentsci.5c00043)
Supplement: Supplementary file 1 [file oc5c00043_si_001.pdf]

Supporting Information

Combustion Resistant Borohydrides and their Chemical Interactions with Li Metal Surfaces: An Experimental and Theoretical Study

Anton W. Tomich, Stephen Proctor, Moon Young Yang, Jianjun Chen, Yifan Zhao, Edward Chen, Tridip Das, Boris V. Merinov, William A. Goddard III, Juchen Guo*, Vincent Lavallo**

Dr. Anton W. Tomich, Stephen Proctor, Edward Chen, Dr. Vincent Lavallo
Department of Chemistry
University of California, Riverside,
California 92521, United States
E-mail: vincent.lavallo@ucr.edu

Dr. Moon Young Yang, Dr. Tridip Das, Dr. Boris V. Merinov,
Dr. William A. Goddard III
Materials and Process Simulations Center
California Institute of Technology
Pasadena, California 91125, United States
E-mail: merinov@caltech.edu, wag@caltech.edu

Jianjun Chen, Dr. Yifan Zhao, Dr. Juchen Guo
Department of Chemical and Environmental Engineering University of California, Riverside,
California 92521, United States
E-mail: jguo@engr.ucr.edu

Table of Contents

General Experimental……………………………………………………………………………………S2
Synthetic Procedures and Spectroscopic Data……………………………………………………S3
Flame Tests (all images) and Melting Points………………………………………………………S76
Differential Scanning Calorimetry……………………………………………………………………S77
Mass Spectrometry ……………………………………………………………………………………S78
Electrolyte Images………………………………………………………………………………………S79
Computational Methods…………………………………………………………………………………S79
Single Crystal Images – vl382at (LiC_{10}-THF) …………………………………………………S81
Crystal Data and Structure Refinement – vl382at (LiC_{10}-THF)………………………………S82
References……………………………………………………………………………………………S96

General Experimental

All manipulations were carried out using standard Schlenk or glovebox techniques under a purified elemental argon (99.995%, glovebox, Schlenk line) unless otherwise stated. Dry THF was obtained via distillation under argon from potassium using benzophenone ketyl radical as an indicator. Unless specifically stated, reagents were purchased from commercial vendors and used without further purification. Nuclear magnetic resonance (NMR) spectroscopy was carried out using: Bruker Avance 600 MHz and Bruker NEO 400 MHz (Prodigy LN2 cryoprobe), Varian Inova 500 MHz and Bruker NEO 600 (CP-MAS). NMR chemical shifts are reported in parts per million (ppm) with ^{1}H and ^{13}C chemical shifts referenced to the residual non-deutero solvent. High-resolution mass spectrometry (HRMS) was collected on an Agilent Technologies 6210 (TOF LC/MS) featuring a direct injection with multimode electrospray ionization/atmospheric-pressure chemical ionization (ESI/APCI).

Melting Point Characterization

Melting point determination was performed on Büchi B-545 Melting point apparatus following sealing of compound in a capillary tube under inert atmosphere. Melting points were measured over a 25 °C – 400 °C range at a 5 °C/min ramp rate. The melting point of **LiC_{10}-THF_4** was estimated using a n-octane/$CO_{2(s)}$ cooling bath monitored via digital thermometer. Samples were contained under Argon atmosphere and submerged in the solution where their liquidus range was monitored as the bath was allowed to cool/warm to/from -50°C. Temperature was controlled by successive additions of dry ice. Differential Scanning Calorimetry of **LiC_4-THF** was performed on a Perkin Elmer DSC 8000 operated under nitrogen atmosphere at 3K/min scan rate following a isothermic cooling of the sample at -70°C for 1 hour.

General Procedure for Flame Tests

All salts were either prepared anhydrous in a glovebox or dried under high temperature and vacuum to remove residual water prior to being removed in a capped vial under $N_{2(g)}$ before burning. Each salt was individually placed onto a glass petri dish and promptly exposed to the tip of a Bunsen burner for 3-10s to observe any combustion. NMRs were taken before and after each experiment to observe any decomposition or changes. Often times, the NMR of the samples that combusted remained mostly unchanged due to insoluble decomposition products visible on the petri dish. However, the salts that did not burn were completely soluble.

Li Metal Compatibility

Commercial 750 µm Li foil (Alfa Aesar) was used as an electrode material. A clean Li nugget was first obtained by cutting the surface of a Li rod (99.8% Strem Chemicals). The Li nugget was laminated to have a flat surface, thickness approximately 500um to 1mm. Li anodes were submerged in a solution of neat **LiC_4-THF** and stored in a sealed/taped vial under argon atmosphere prior to surface characterization. Li powder was freshly prepared from Li metal ingots in silicon oil, a quantity of which was heated to 200°C, immersion blended, and washed with pentane under argon atmosphere. Li powder was added to ~1 mL of neat **LiC_4-THF** and stirred at room temperature under argon atmosphere for a period of 1 month. A small sample of the solution was taken and dissolved in $CDCl_3$ for spectroscopic characterization.

Scanning Electron Microscopy (SEM) and Energy Dispersive X-ray Spectrometry (EDXS):

The surface morphology of the lithium metal was measured using SEM (Nova Nano S450, 18 kV). The soaked samples were thoroughly rinsed with hexane to eliminate any residual electrolytes and then dried at room temperature for 24 hours in an argon-filled glovebox. Subsequently, the samples were transported to the SEM facility inside a stainless-steel tube with KF-flange sealing. The elemental mapping of the samples was collected using an EDS spectrometer coupled with the SEM.

X-ray Photoelectron Spectroscopy (XPS):

XPS measurements were conducted using a Kratos AXIS Supra with an Al Kα source (1486.7 eV) at the UC Irvine Materials Research Institute (IMRI). Following the SEM sample preparation procedure, the samples were transported to the XPS facility inside a stainless-steel tube with KF flange sealing. Subsequently, the samples were loaded into the sample chamber within the glovebox, which was integrated with the Kratos AXIS Supra for XPS analysis. All peaks of XPS data were analyzed by Avantage and calibrated with the reference peak of C 1s at 284.8 eV (the adventitious carbon).

Synthetic Procedures and Spectroscopic Data

Synthesis of [Cs$^+$][HCB$_9$H$_9$$^{1-}$] – Figures S1 – S7

[Cs$^+$][HCB$_9$H$_9$$^{1-}$] was prepared following known literature procedures from decaborane ($B_{10}H_{14}$) which was sublimed prior to use.[1] For the flame test, **[Cs$^+$][HCB$_9$H$_9$$^{1-}$]** was then dried under vacuum (0.5 torr) at 160 °C overnight to remove residual water. m.p. > 400 °C.

^{11}B{^{1}H} NMR (128 MHz, d$_6$-acetone): δ 30.84 ppm (s, 1 B-H), 18.56 ppm (s, 4 B-H), -24.00 ppm (s, 4 B-H). ^{11}B NMR (128 MHz, d$_6$-acetone) δ 30.84 (d, *J* = 149.7 Hz, 1 B-H), -18.57 (d, *J* = 149.5 Hz, 4 B-H), -24.02 (d, *J* = 135.3 Hz, 4 B-H). ^{1}H NMR (400 MHz, d$_6$-acetone) δ 5.41 (q, *J* = 151.3 Hz, 1 B-H), 4.61 (s, 1H), 1.00 (td, *J* = 296.4, 143.1 Hz, 8 B-H). ^{13}C NMR (101 MHz, d$_6$-acetone) δ 53.44.

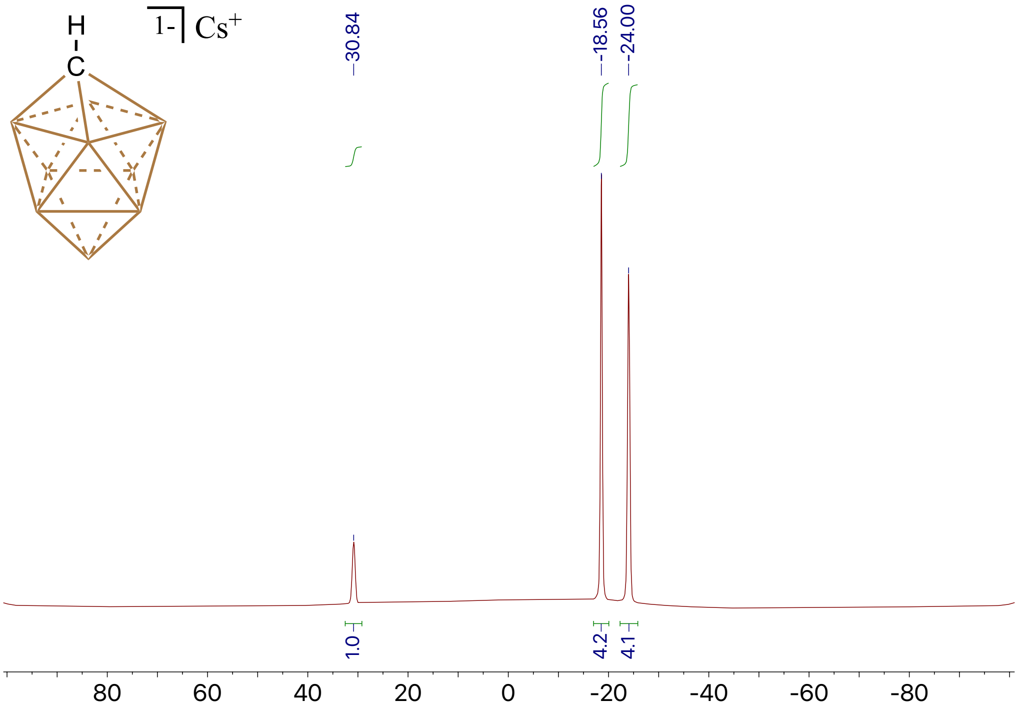

Figure S1. ^{11}B{^{1}H} NMR of **[Cs$^+$][HCB$_9$H$_9$$^{1-}$]** in d$_6$-acetone.

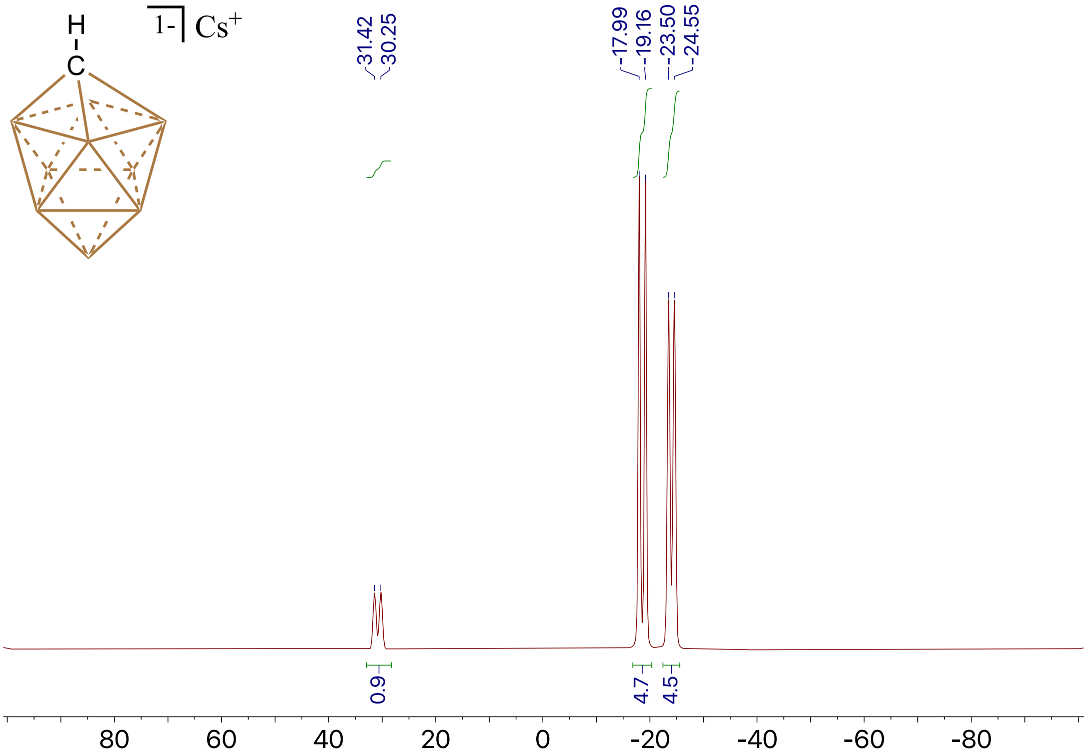

Figure S2. ^{11}B NMR of **$[Cs^+][HCB_9H_9{}^{1-}]$** in d_6-acetone.

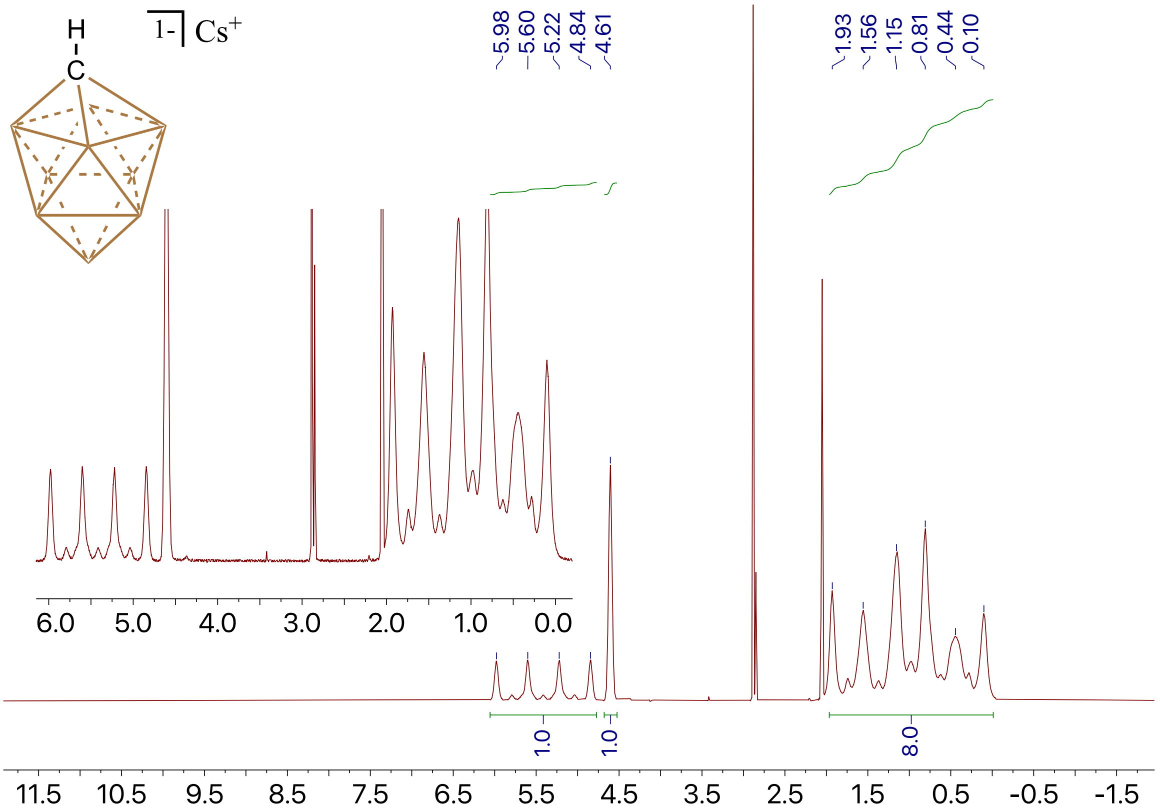

Figure S3. ^1H NMR of **$[Cs^+][HCB_9H_9{}^{1-}]$** in wet d_6-acetone. Note: H_2O and HDO observed at 2.88 ppm.

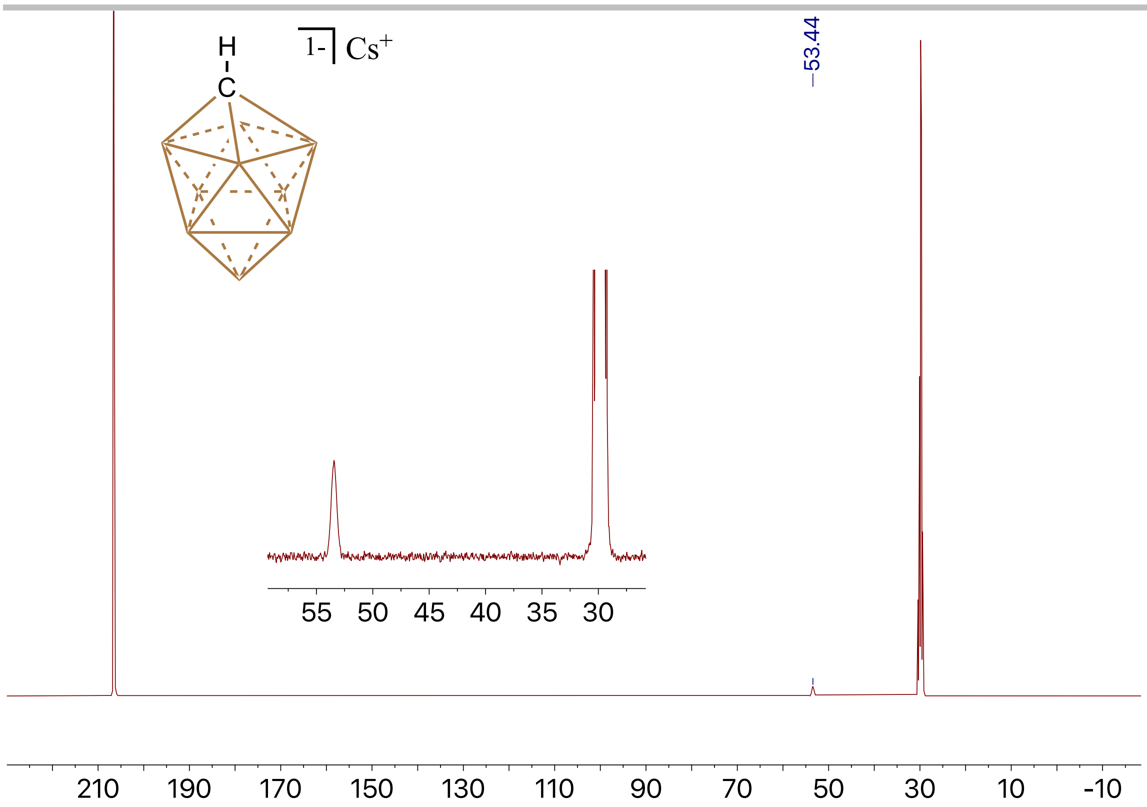

Figure S4. $^{13}C\{^1H\}$ NMR of **[Cs$^+$][HCB$_9$H$_9$$^{1-}$]** in d$_6$-acetone. Carboranyl C is at 53.44 ppm.

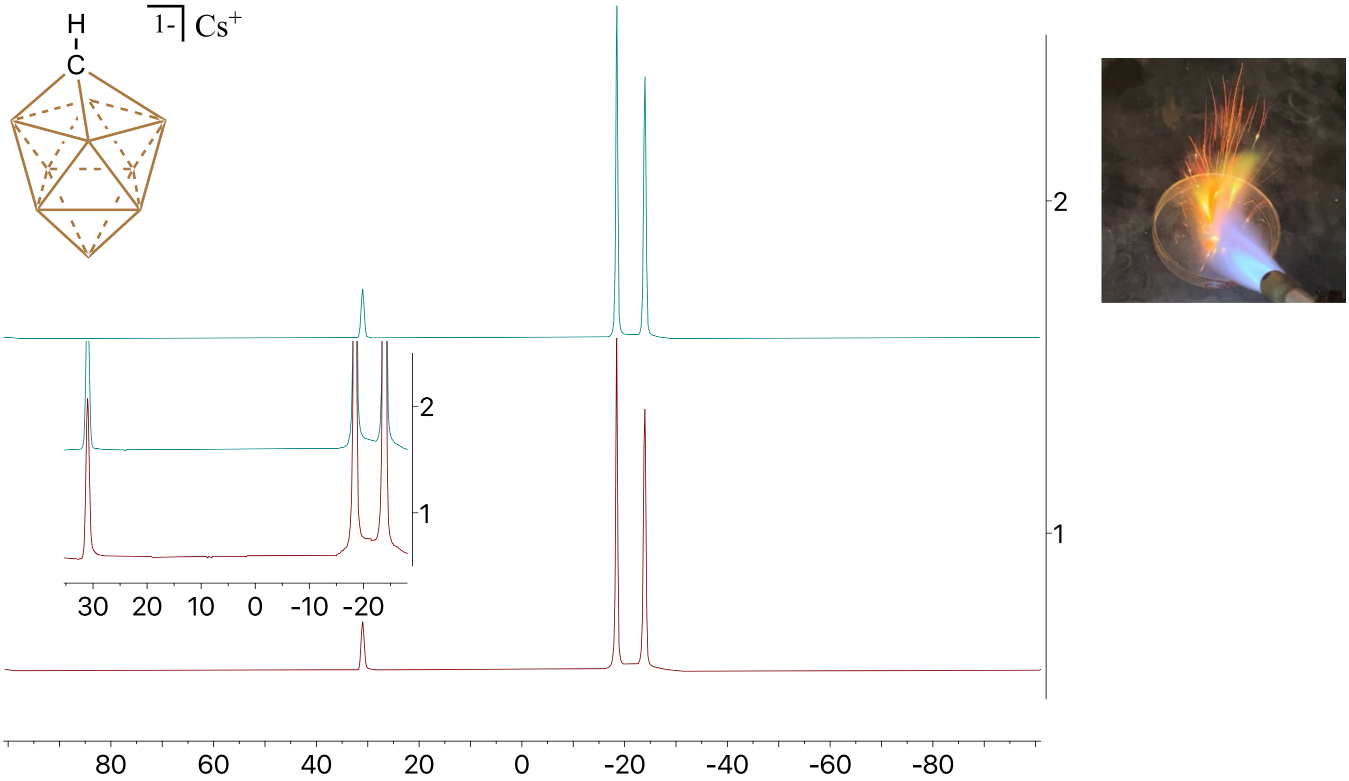

Fig S5. $^{11}B\{^1H\}$ NMR of **[Cs$^+$][HCB$_9$H$_9$$^{1-}$]** before (above) and after (below) burning 3s in d$_6$-acetone.

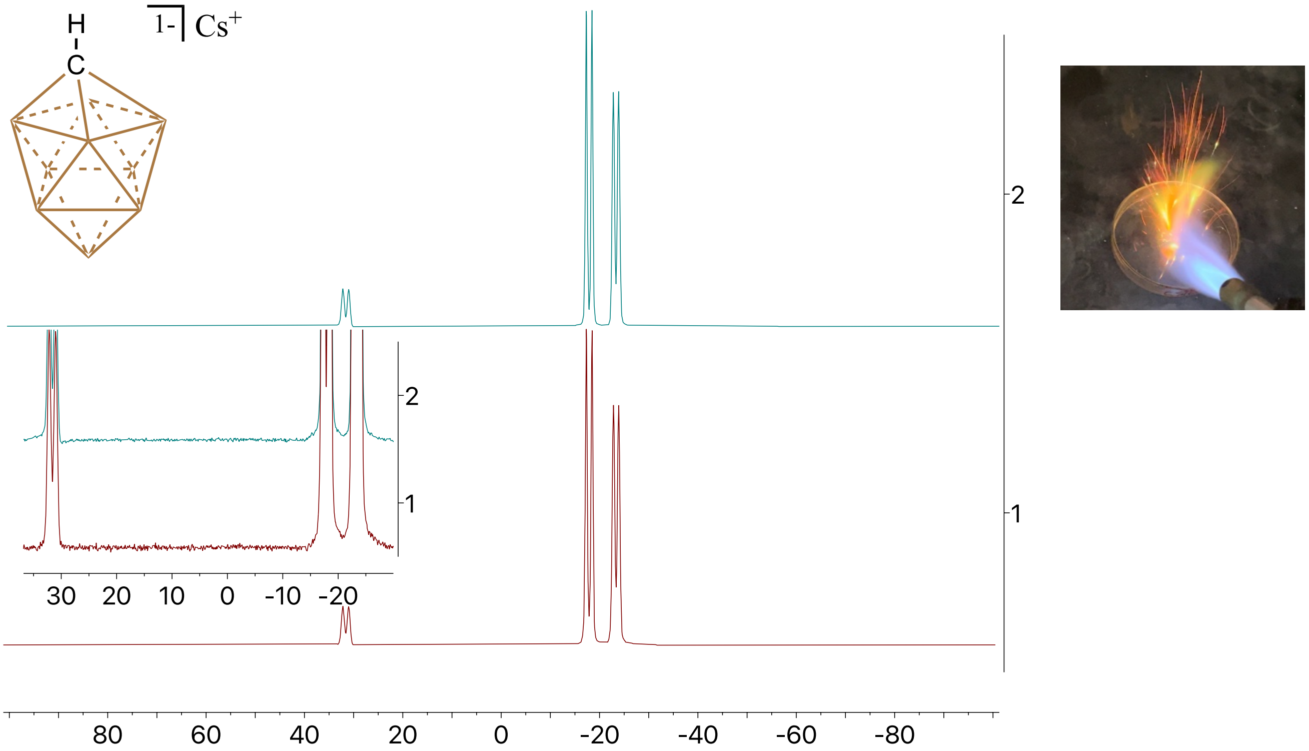

Fig S6. ^{11}B NMR of **[Cs⁺][HCB₉H₉¹⁻]** before (above) and after (below) burning 3s in d₆-acetone.

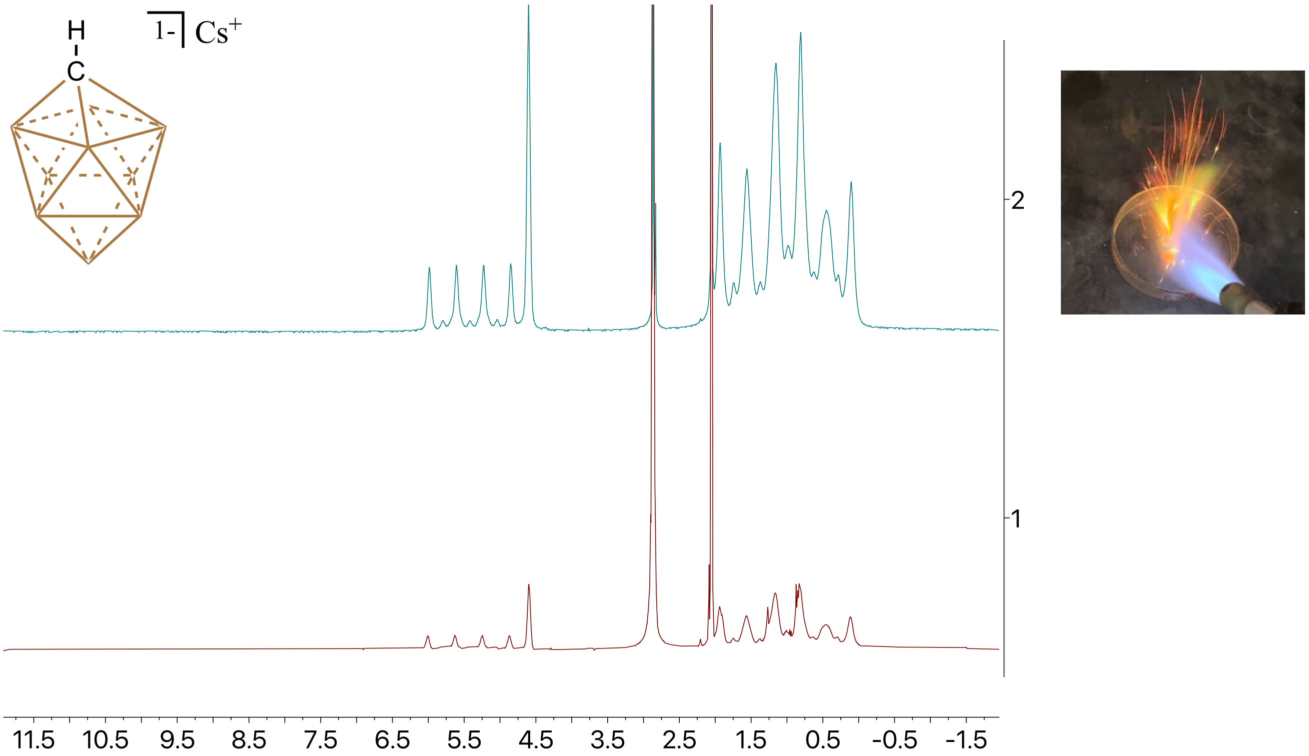

Fig S7. ^{1}H NMR of **[Cs^{+}][$HCB_9H_9^{1-}$]** before (above) and after (below) burning 3s in d_6-acetone

Synthesis of [$HNMe_3^+$][$HCB_9H_9^{1-}$] – Figures S8 – S10

[$HNMe_3^+$][$HCB_9H_9^{1-}$] was prepared by dissolution of **[Cs^+][$HCB_9H_9^{1-}$]** in boiling water followed by the addition of NMe_3•HCl. The aqueous solution of **[$HNMe_3^+$][$HCB_9H_9^{1-}$]** was allowed to cool to room temperature which resulted in the crystallization of **[$HNMe_3^+$][$HCB_9H_9^{1-}$]**. **[$HNMe_3^+$][$HCB_9H_9^{1-}$]** was dried for 12 hours in vacuo at 170°C and was stored in a glovebox under nitrogen atmosphere before use.

^{11}B{^{1}H} NMR (128 MHz, d_6-acetone) δ 30.31 (s, 1 B-H), -19.10 (s, 4 B-H), -24.56 (s, 4 B-H). ^{11}B NMR (128 MHz, d_6-acetone) δ 30.35 (d, *J* = 150.8 Hz, 1 B-H), -19.10 (d, *J* = 148.6 Hz, 4 B-H), -24.59 (d, *J* = 136.2 Hz, 4 B-H). ^{1}H NMR (400 MHz, d_6-acetone) δ 5.43 (q, *J* = 151.5 Hz, 1 B-H), 4.60 (s, 1H), 3.20 (s, 9H), 1.01 (td, *J* = 294.9, 142.8 Hz, 8 B-H).

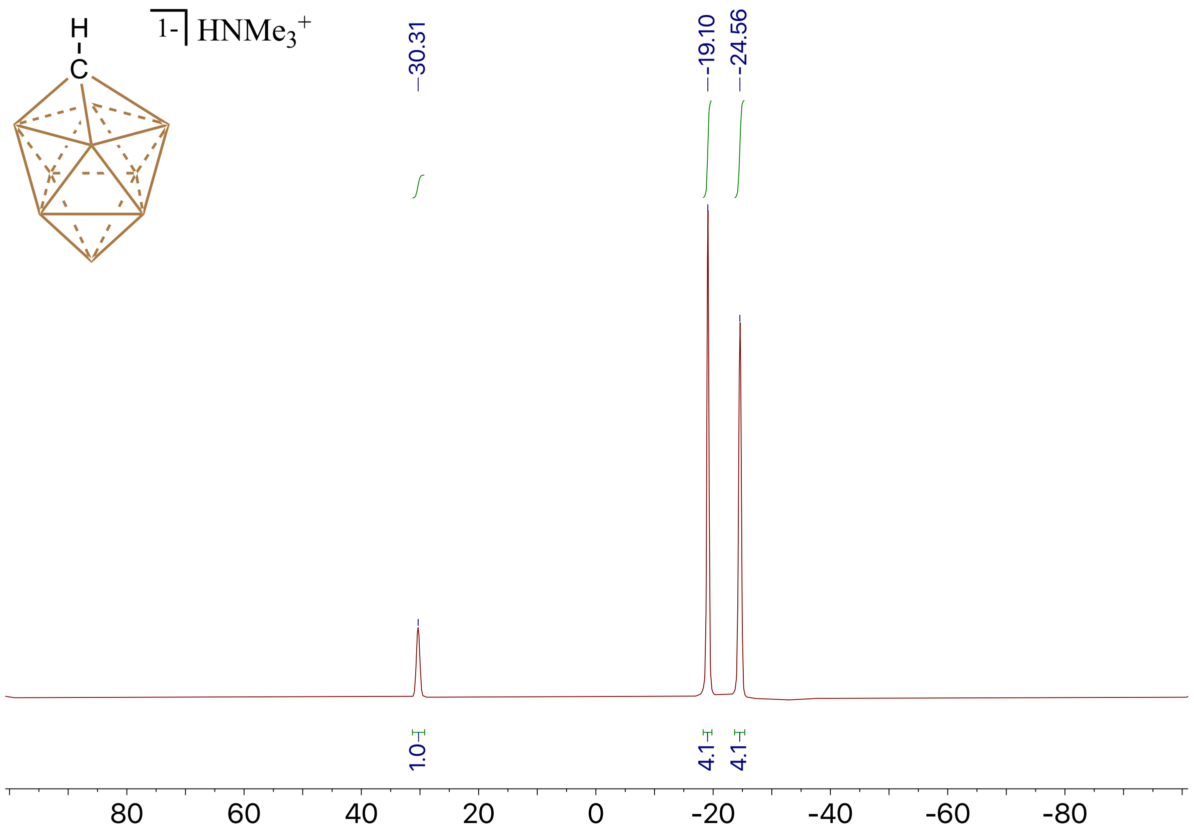

Figure S8. ^{11}B{^{1}H} NMR of **[$HNMe_3^+$][$HCB_9H_9^{1-}$]** in d_6-acetone.

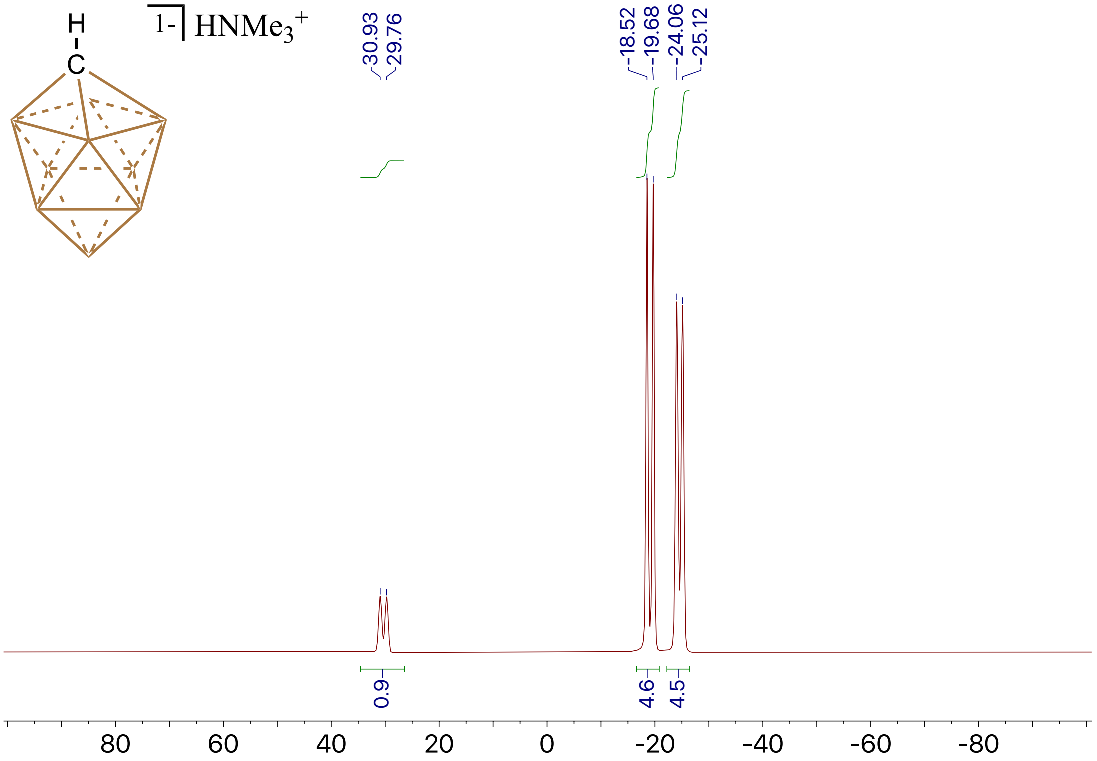

Figure S9. ^{11}B NMR of **[$HNMe_3^+$][$HCB_9H_9^{1-}$]** in d_6-acetone.

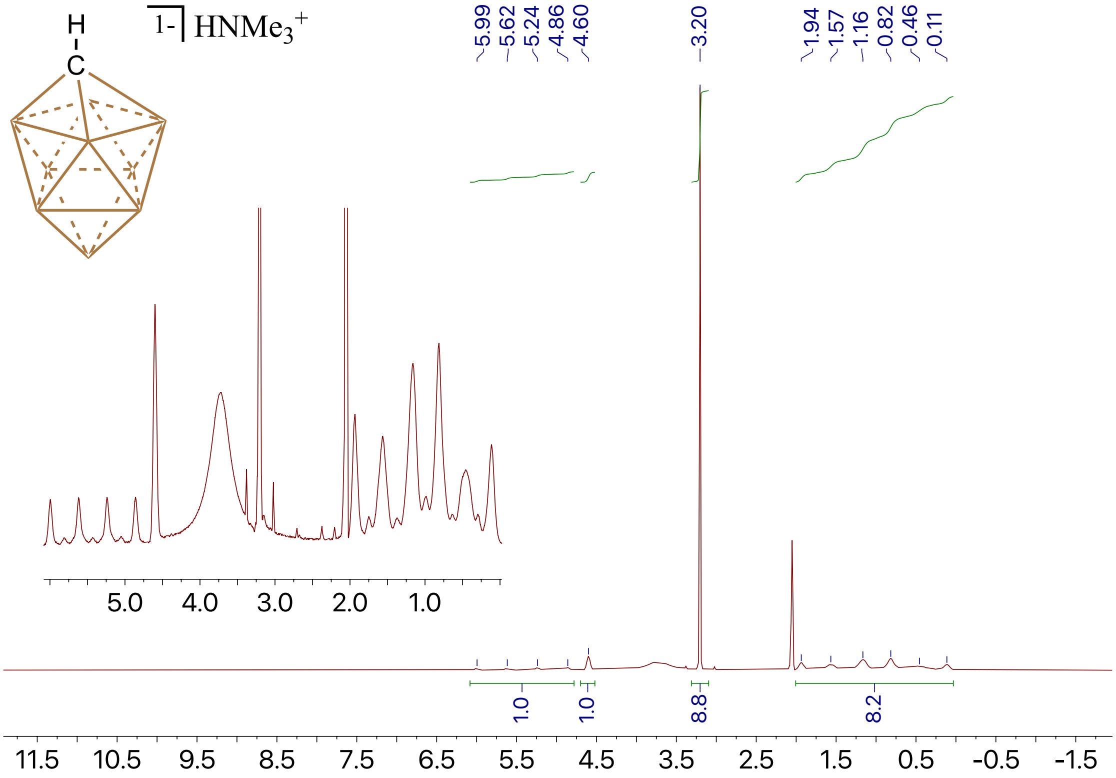

Figure S10. ^{1}H NMR of **[$HNMe_3^+$][$HCB_9H_9^{1-}$]** in wet d_6-acetone. Note: appearance of a broad singlet at 3.8 ppm is water.

Synthesis of [Li(THF)$_n$$^+$][HCB$_9H_9$$^{1-}$] where n = 0, 1, 4 – Figures S11 – S31

140 mg of **[HNMe$_3$$^+$][HCB$_9H_9$$^{1-}$]** was dissolved in dry THF to yield a clear and colorless solution. Upon addition of excess LiH (1.5 molar equiv.), the solution became murky and effervescence of gaseous H_2 and NMe_3 byproducts occurred rapidly. This solution was allowed to stir overnight, then pipette filtered through glass microfiber filter papers and celite. Removal of THF under reduced pressure and at room temperature yields the coordinatively saturated **[Li(THF)$_4$$^+$][HCB$_9H_9$$^{1-}$]** (m.p. = 89.5 – 91.5 °C) as a white solid (yield: 271 mg, 84%). Further desolvation at 120 °C for 12 hours yields the sparingly solvated **[Li(THF)$_1$$^+$][HCB$_9H_9$$^{1-}$]** (m.p. = 79.5 – 85.8 °C) and extensive desolvation at 190 °C for 48 hours yields THF-free **[Li$^+$][HCB$_9$H$_9$$^{1-}$]** (m.p. = 348.2 – 351.1 °C).

n=0: ^{11}B NMR (128 MHz, d$_6$-acetone) δ 29.82 (s, 1 B-H), -19.67 (s, 4 B-H), -25.13 (s, 4 B-H). ^{11}B NMR (128 MHz, d$_6$-acetone) δ 29.82 (d, *J* = 151.4 Hz, 1 B-H), -19.67 (d, *J* = 149.0 Hz, 4 B-H), -25.15 (d, *J* = 136.1 Hz, 4 B-H). ^{1}H NMR (400 MHz, d$_6$-acetone) δ 5.43 (q, *J* = 151.6 Hz, 1 B-H), 4.59 (s, 1H), 0.82 (dt, *J* = 280.0, 148.6 Hz, 8 B-H). ^{7}Li NMR (156 MHz, d$_6$-acetone) δ 4.34.
n=1: ^{11}B{^{1}H} NMR (128 MHz, d$_6$-acetone) δ 30.30 (s, 1 B-H), -19.20 (s, 4 B-H), -24.70 (s, 4 B-H). ^{11}B NMR (128 MHz, d$_6$-acetone) δ 30.27 (d, *J* = 154.5 Hz, 1 B-H), -19.23 (d, *J* = 148.5 Hz, 4 B-H), -24.70 (d, *J* = 135.5 Hz, 4 B-H). ^{1}H NMR (400 MHz, d$_6$-acetone) δ 5.44 (q, *J* = 151.5 Hz, 1 B-H), 4.59 (s, 1H), 3.62 (m, 4H), 1.79 (m, 4H). ^{7}Li NMR (156 MHz, d$_6$-acetone) δ 1.54.
n=4: ^{11}B{^{1}H} NMR (128 MHz, d$_6$-acetone) δ 31.96 (s, B-H), -17.54 (s, 4 B-H), -23.02 (s, 4 B-H). ^{11}B NMR (128 MHz, d$_6$-acetone) δ 31.96 (d, *J* = 150.8 Hz), -17.56 (d, *J* = 148.8 Hz), -23.04 (d, *J* = 135.1 Hz). ^{1}H NMR (400 MHz, d$_6$-acetone) δ 5.44 (q, *J* = 151.7 Hz, 1 B-H), 4.59 (s, 1H), 3.62 (m, 15H), 1.79 (m, 15H), 0.82 (dt, *J* = 280.0, 148.6 Hz, 8 B-H). ^{7}Li NMR (233 MHz, d$_6$-acetone) δ 2.67 (s)

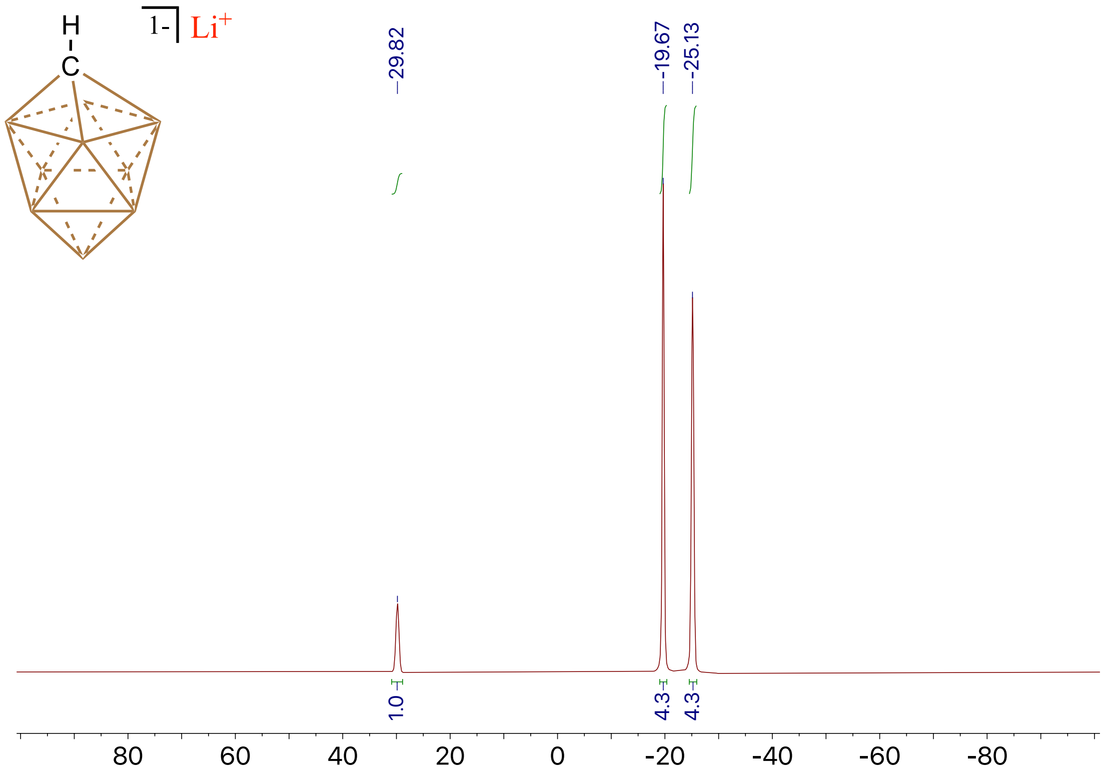

Figure S11. ^{11}B{^{1}H} NMR of **[Li$^+$][HCB$_9$H$_9$$^{1-}$]** in d$_6$-acetone.

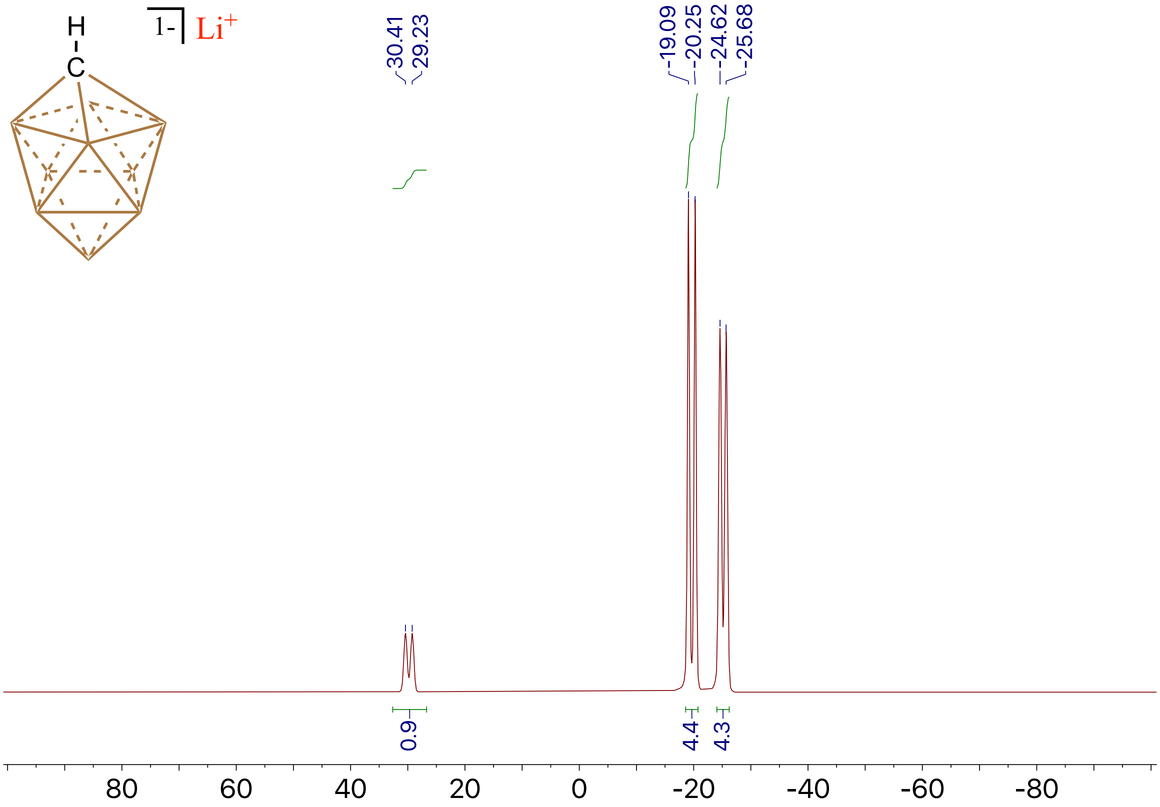

Figure S12. ^{11}B NMR of **[Li^{+}][HCB$_9$H$_9$$^{1-}$]** in d$_6$-acetone.

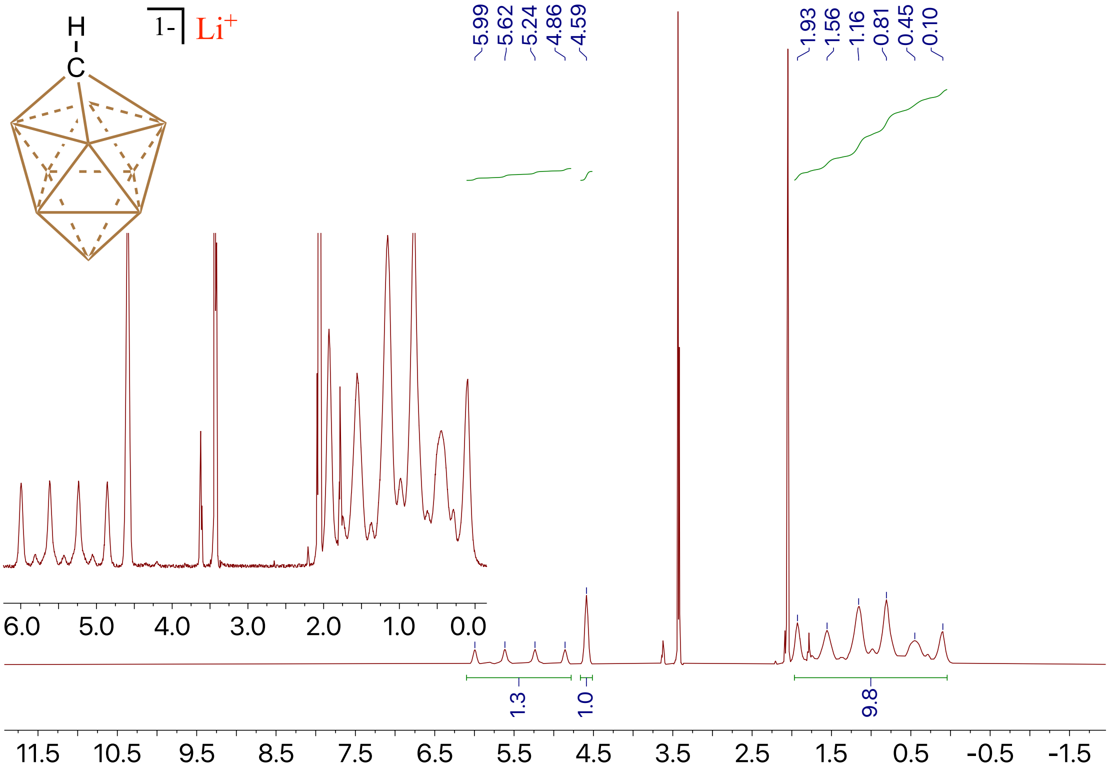

Figure S13. ^{1}H NMR of **[Li^{+}][HCB$_9$H$_9$$^{1-}$]** in d$_6$-acetone. Note: There are 2.5 THF molecules per 100 [Li^{+}][HCB$_9$H$_9$$^{1-}$].

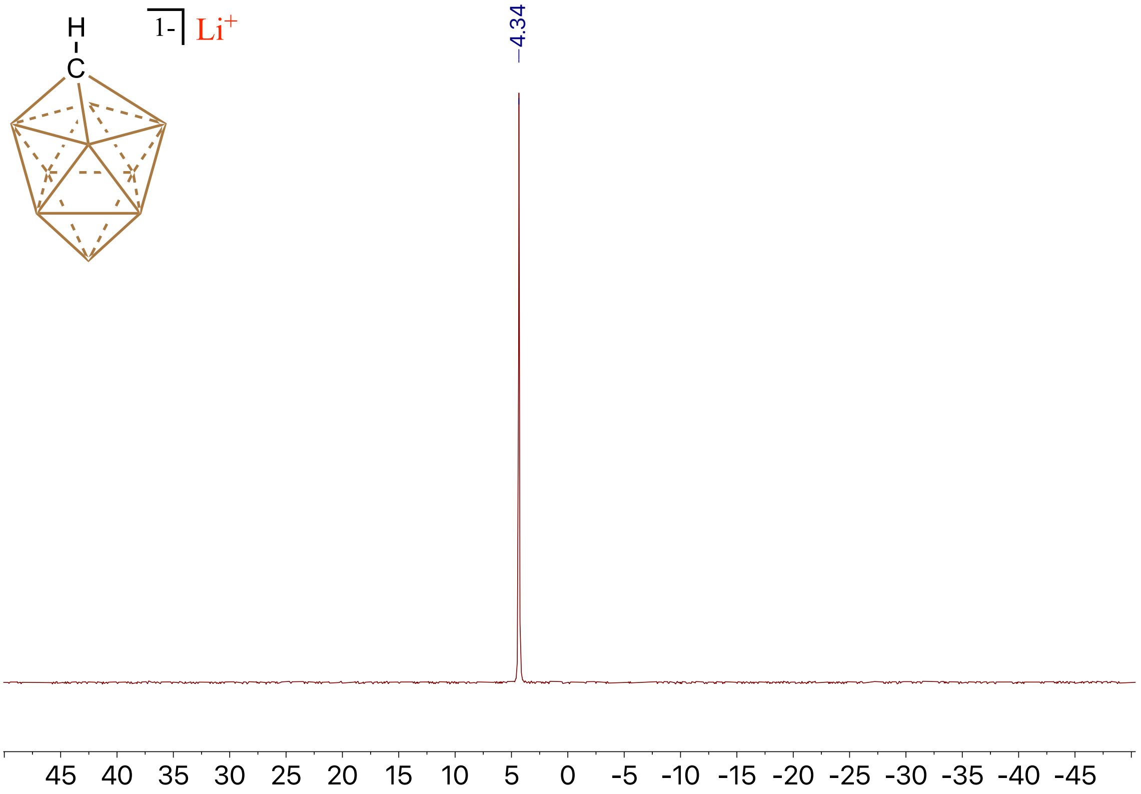

Figure S14. ^{7}Li NMR of **[Li^{+}][HCB$_9$H$_9$$^{1-}$]** in d$_6$-acetone.

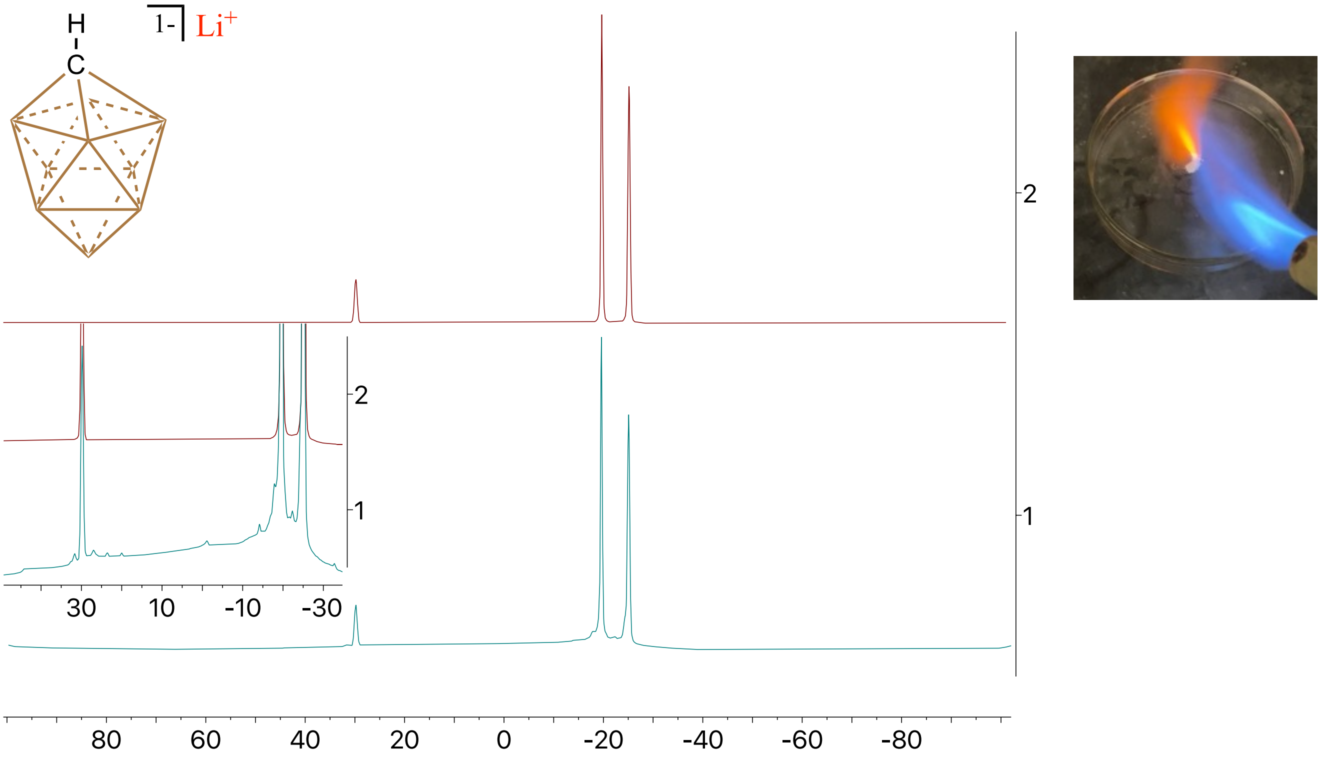

Figure S15. ^{11}B{^{1}H} NMR of **[Li^{+}][HCB$_9$H$_9$$^{1-}$]** before (above) and after (below) burning 3s in d$_6$-acetone. Minor decomposition observed.

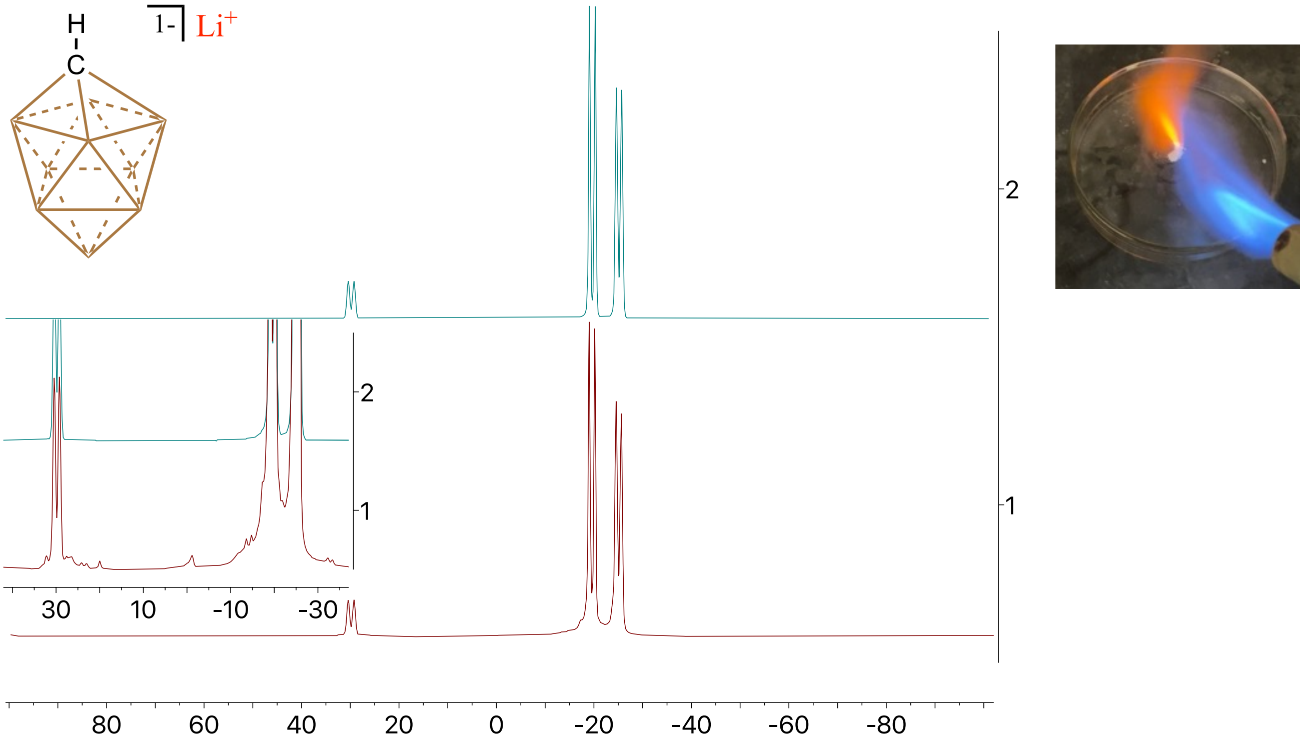

Figure S16. ^{11}B NMR of **[Li$^+$][HCB$_9$H$_9$$^{1-}$]** before (above) and after (below) burning 3s in d$_6$-acetone. Minor decomposition observed.

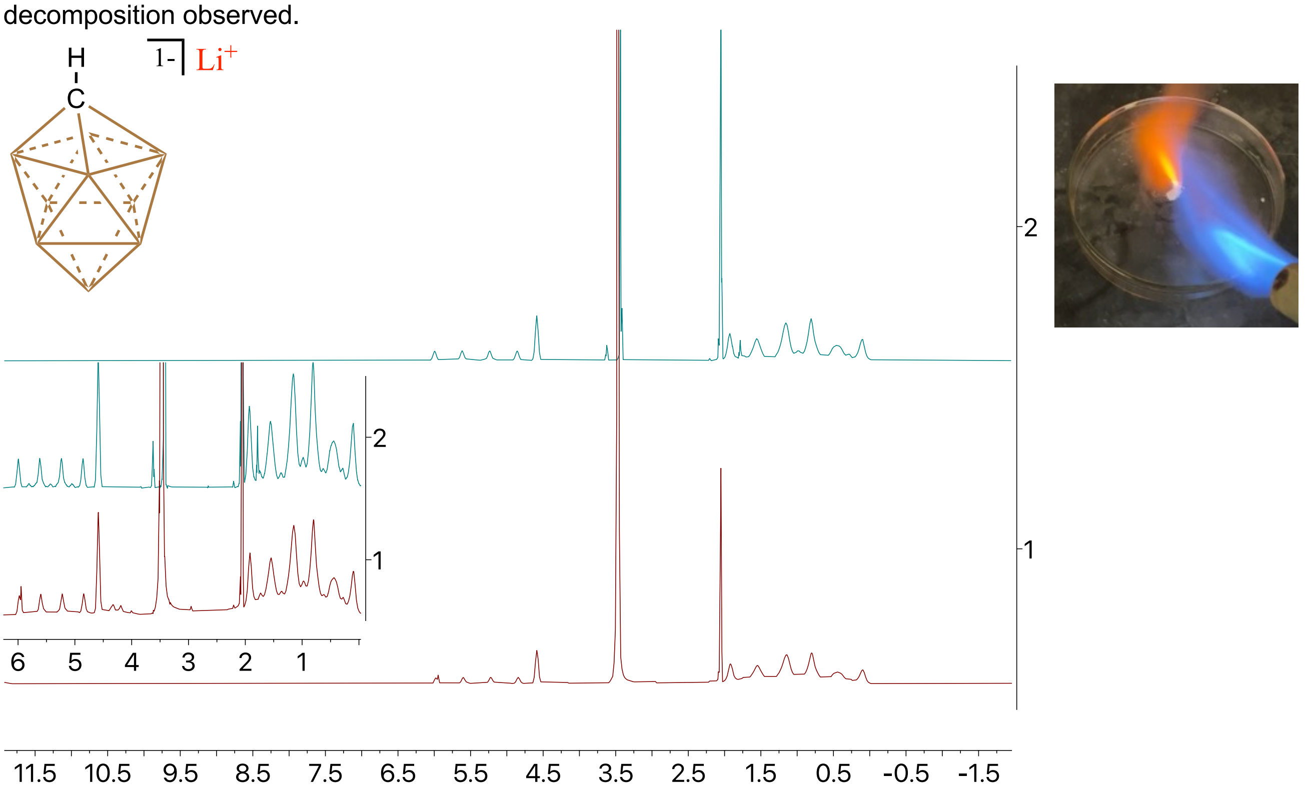

Figure S17. ^{1}H NMR of **[Li$^+$][HCB$_9$H$_9$$^{1-}$]** before (above) and after (below) burning 3s in d$_6$-acetone. Note: water peaks arise at 3.4 ppm due to strong coordination to naked Li$^+$.

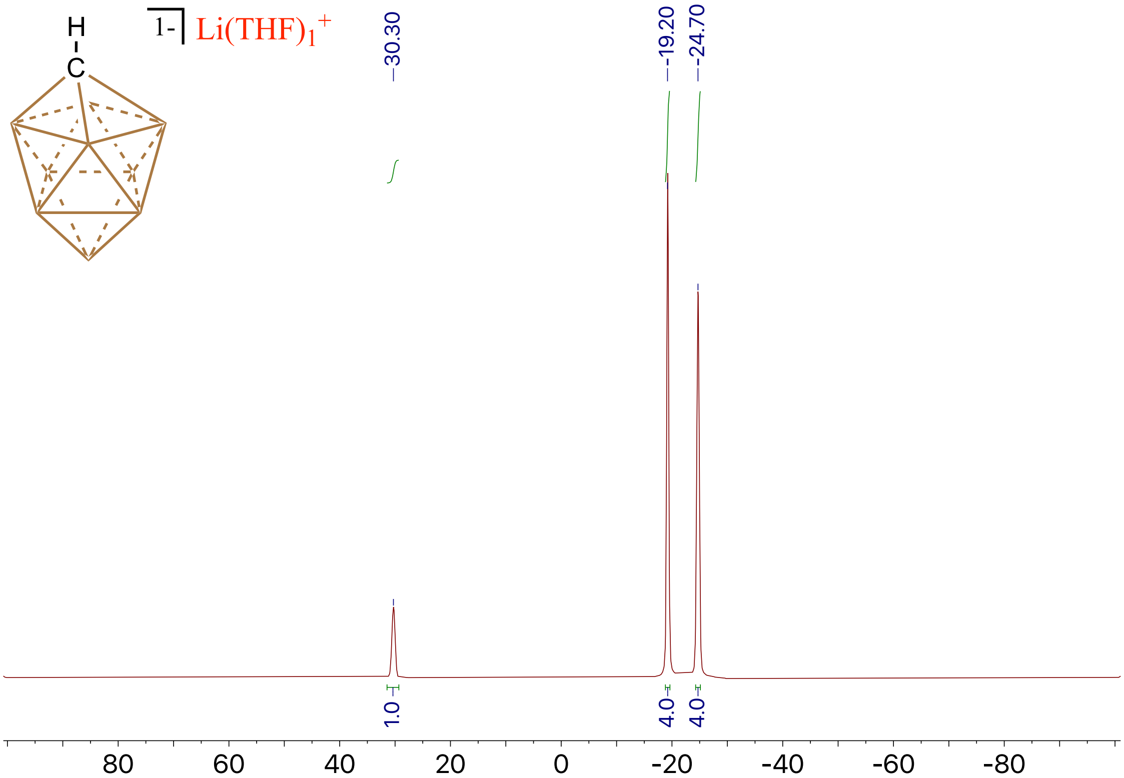

Figure S18. $^{11}B\{^1H\}$ NMR of **$[Li(THF)_1^+][HCB_9H_9^{1-}]$** in d_6-acetone.

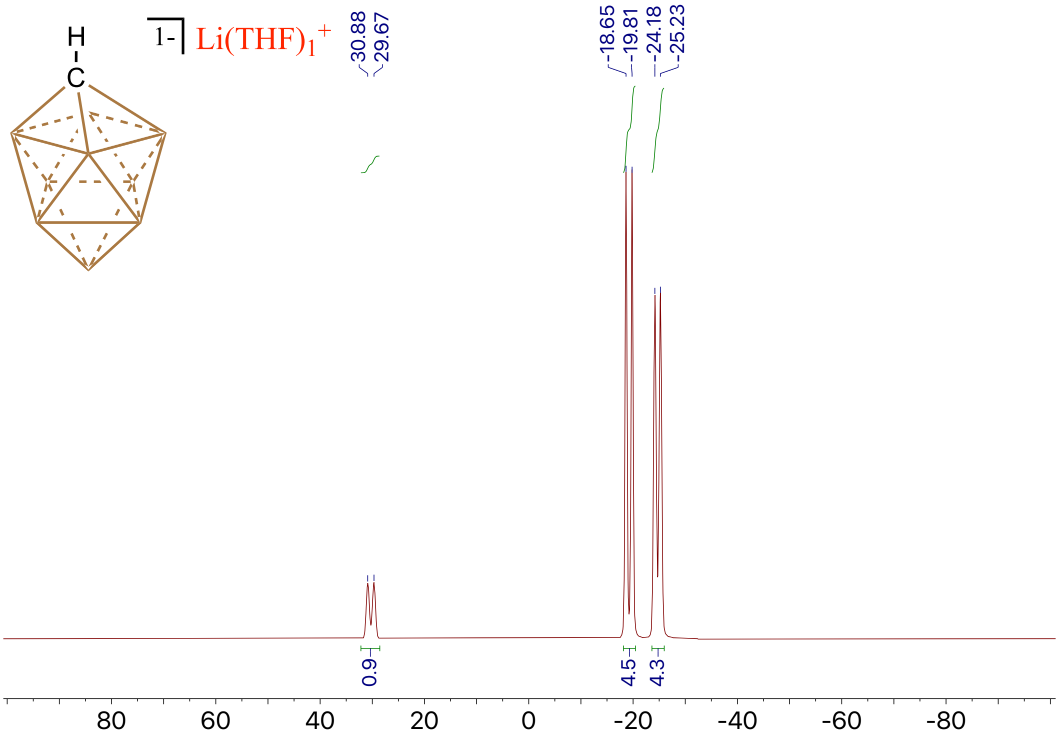

Figure S19. ^{11}B NMR of **$[Li(THF)_1^+][HCB_9H_9^{1-}]$** in d_6-acetone.

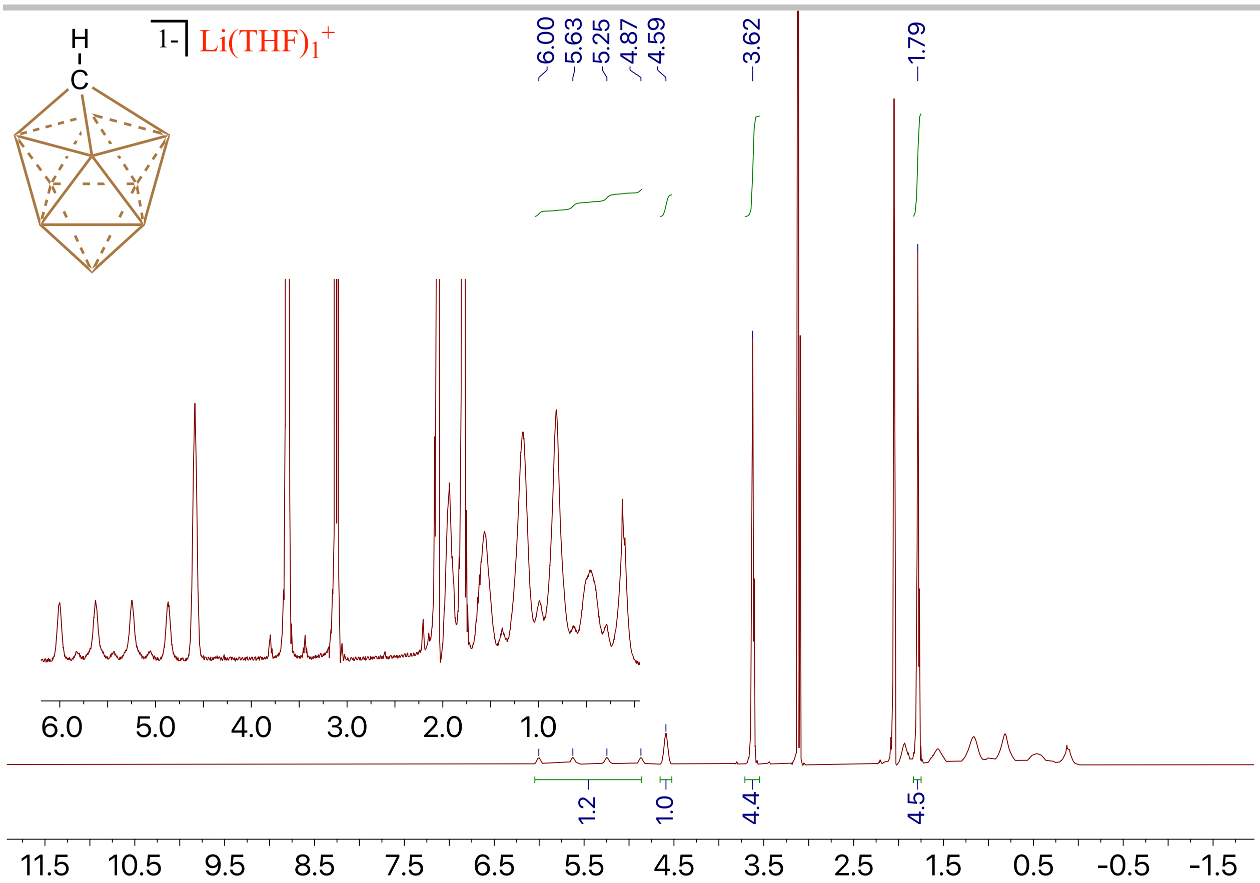

Figure S20. ^{1}H NMR of **[Li(THF)$_1$$^{+}$][HCB$_9H_9$$^{1-}$]** in wet d$_6$-acetone. Note water peaks arise at 3.1 ppm.

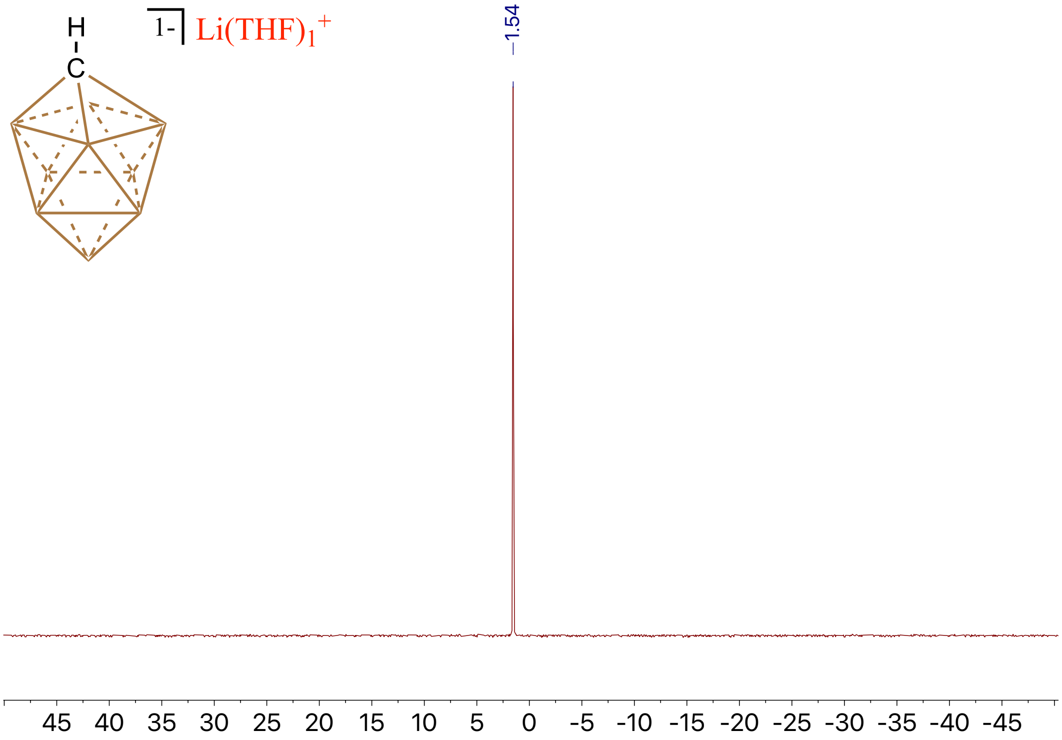

Figure S21. ^{7}Li NMR of **[Li(THF)$_1$$^{+}$][HCB$_9H_9$$^{1-}$]** in d$_6$-acetone.

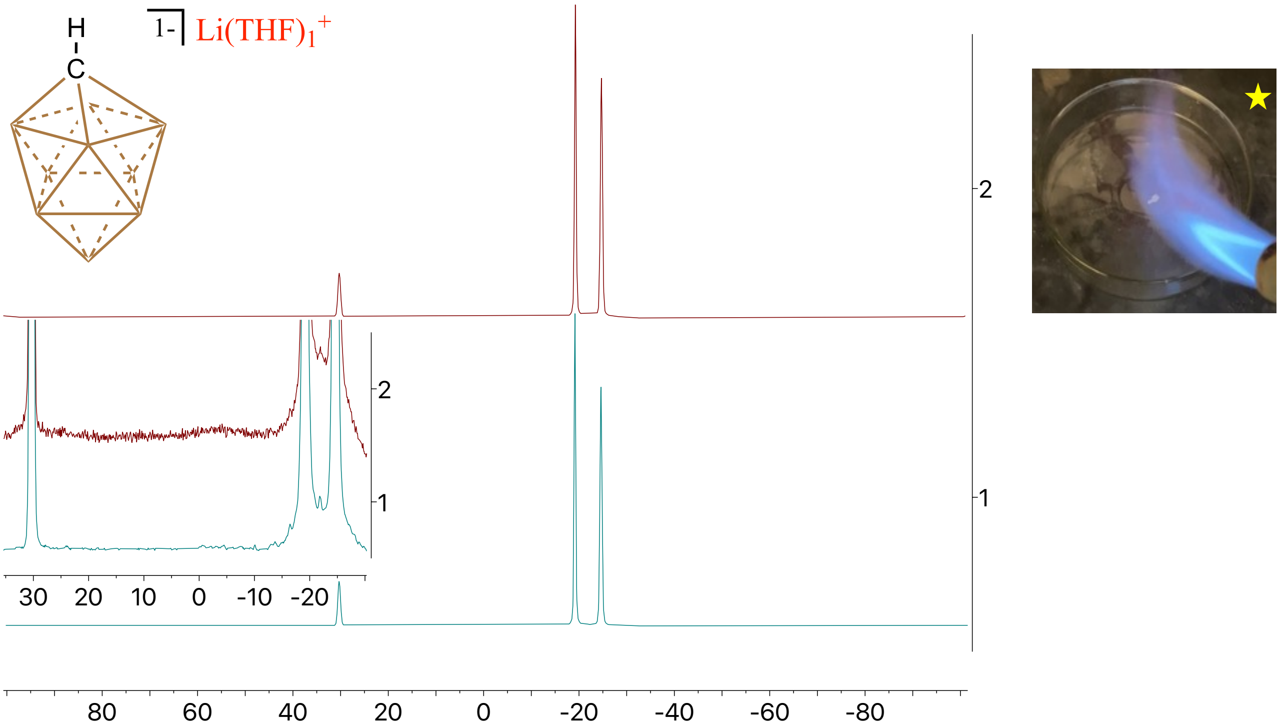

Figure S22. $^{11}B\{^{1}H\}$ NMR of **[Li(THF)$_1^+$][HCB$_9$H$_9^{1-}$]** before (above) and after (below) burning 3s in d_6-acetone.

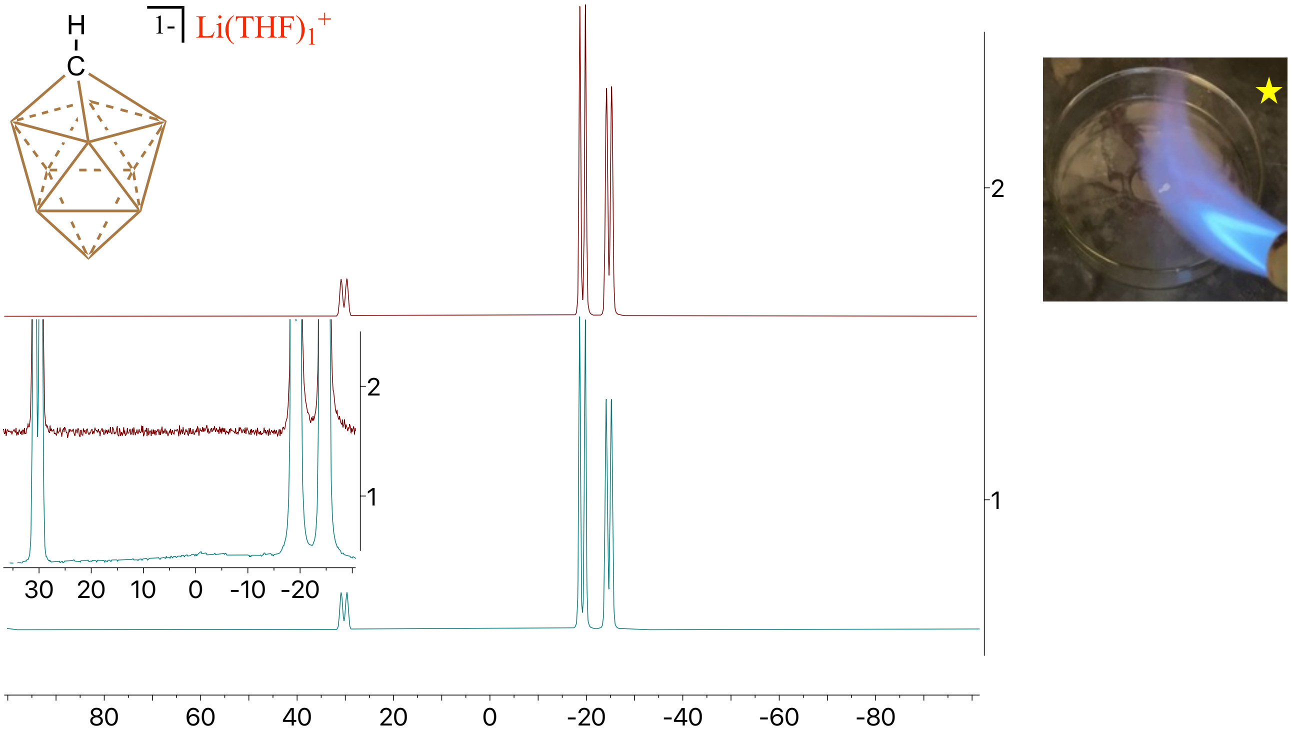

Figure S23. ^{11}B NMR of **[Li(THF)$_1^+$][HCB$_9$H$_9^{1-}$]** before (above) and after (below) burning 3s in d_6-acetone.

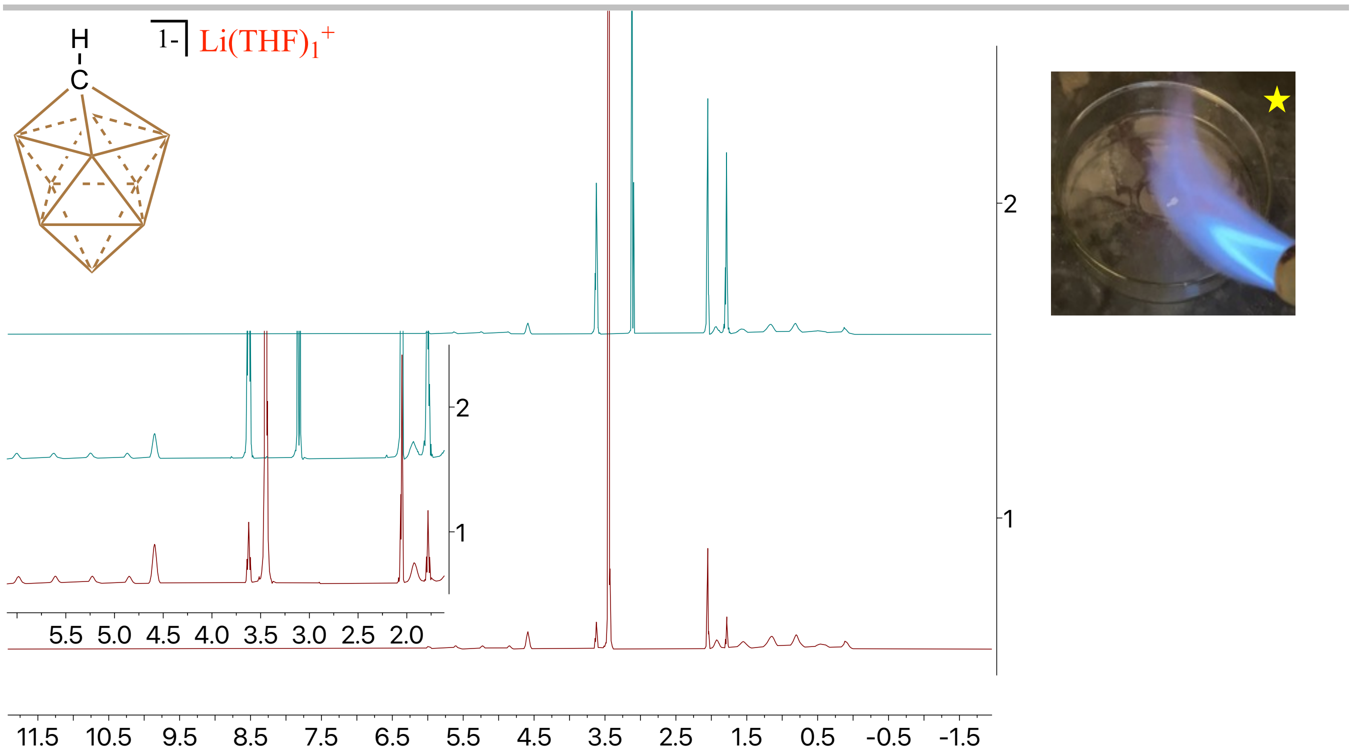

Figure S24. ^{1}H NMR of **[Li(THF)$_1$$^+$][HCB$_9H_9$$^{1-}$]** before (above) and after (below) burning 3s in d$_6$-acetone. Note: water peaks arise at 3.1 (above) and 3.4 ppm (below).

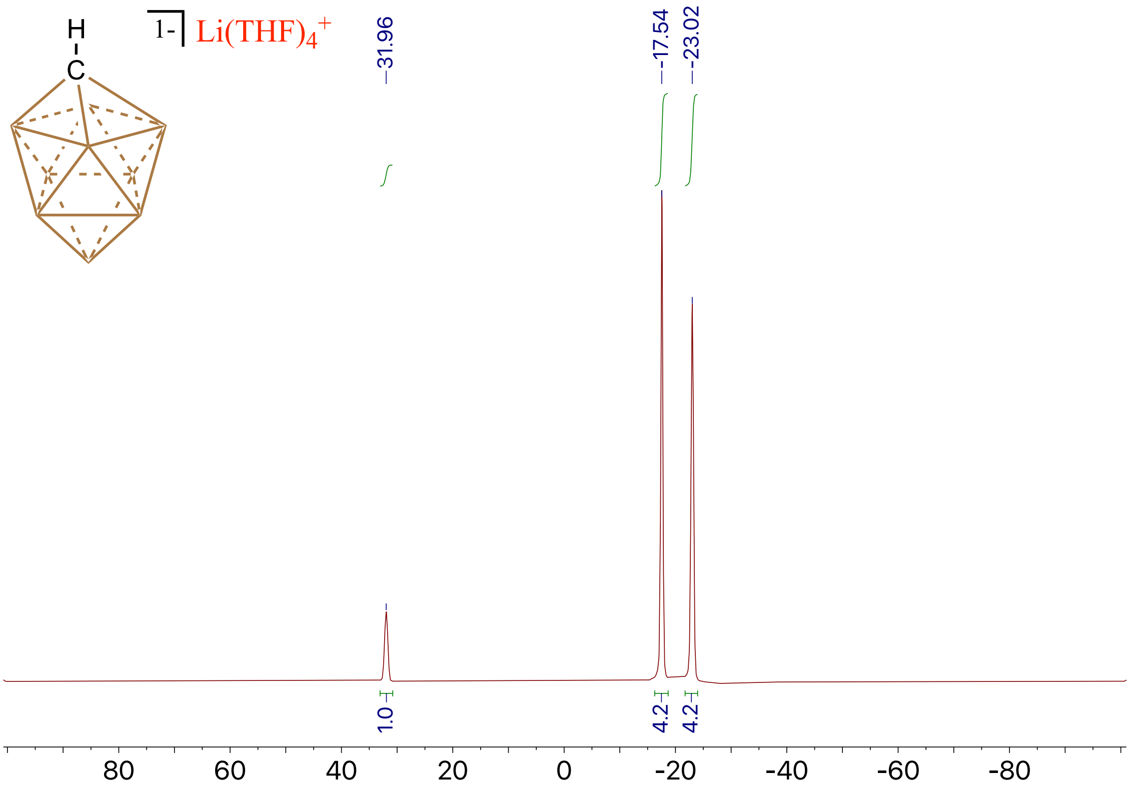

Figure S25. $^{11}B\{^{1}H\}$ NMR of **[Li(THF)$_4$$^+$][HCB$_9H_9$$^{1-}$]** in d$_6$-acetone.

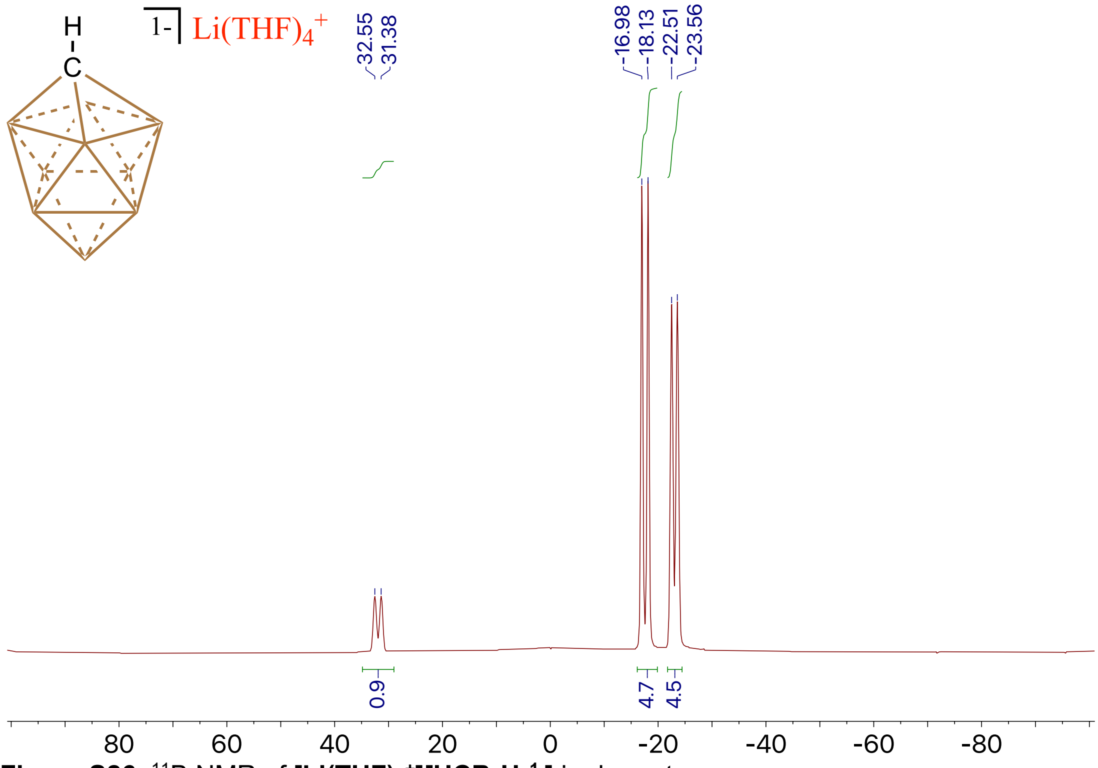

Figure S26. ^{11}B NMR of **[Li(THF)$_4$$^+$][HCB$_9H_9$$^{1-}$]** in d$_6$-acetone.

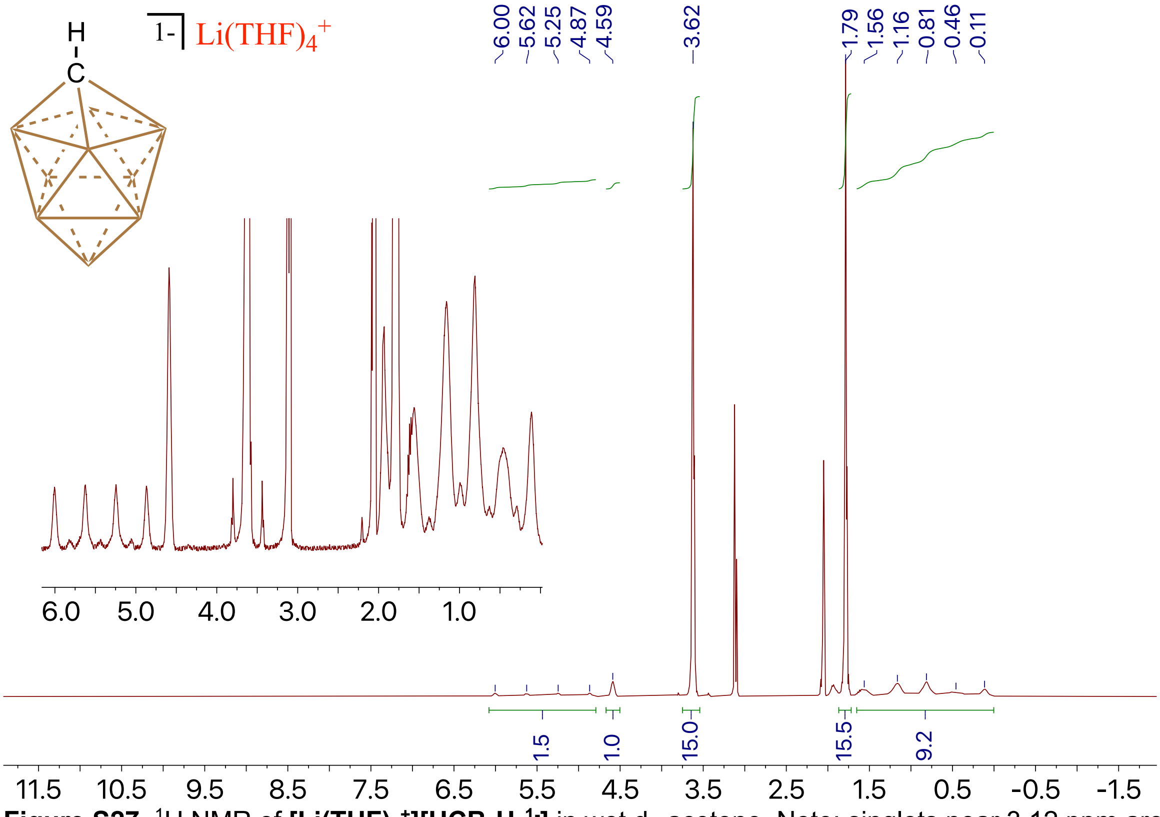

Figure S27. ^{1}H NMR of **[Li(THF)$_4$$^+$][HCB$_9H_9$$^{1-}$]** in wet d$_6$-acetone. Note: singlets near 3.12 ppm are water.

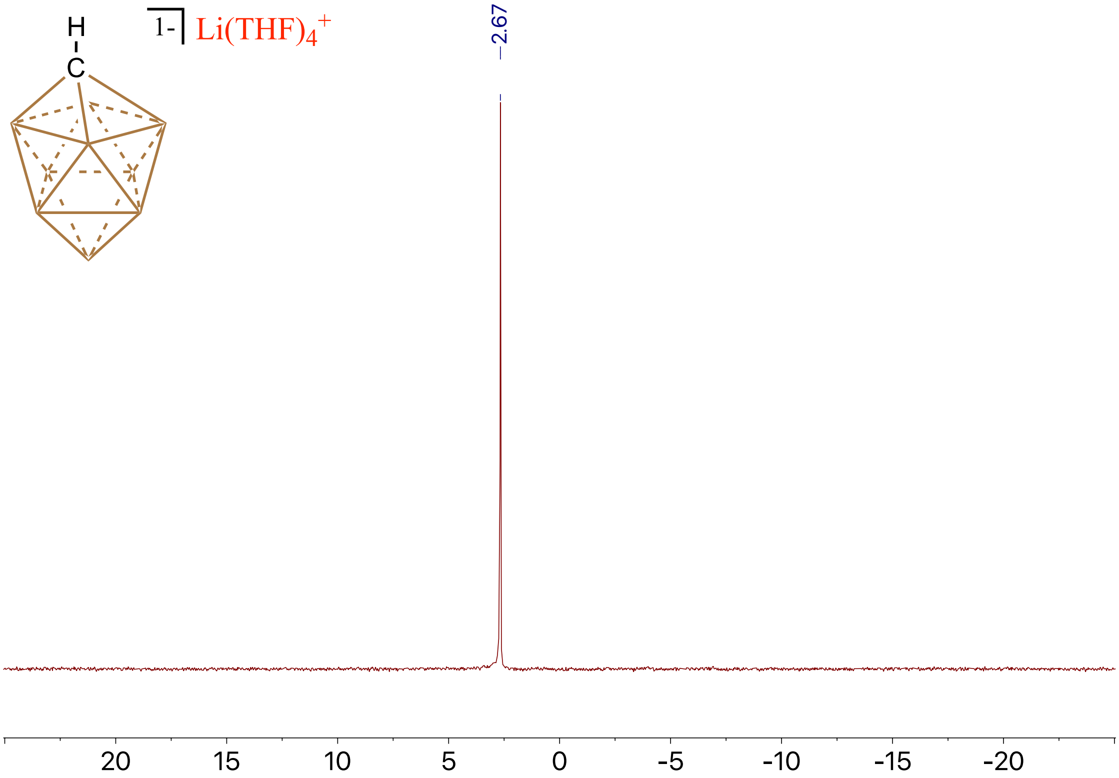

Figure S28. ^{7}Li NMR of **[Li(THF)$_4^+$][HCB$_9$H$_9^{1-}$]** in wet d$_6$-acetone.

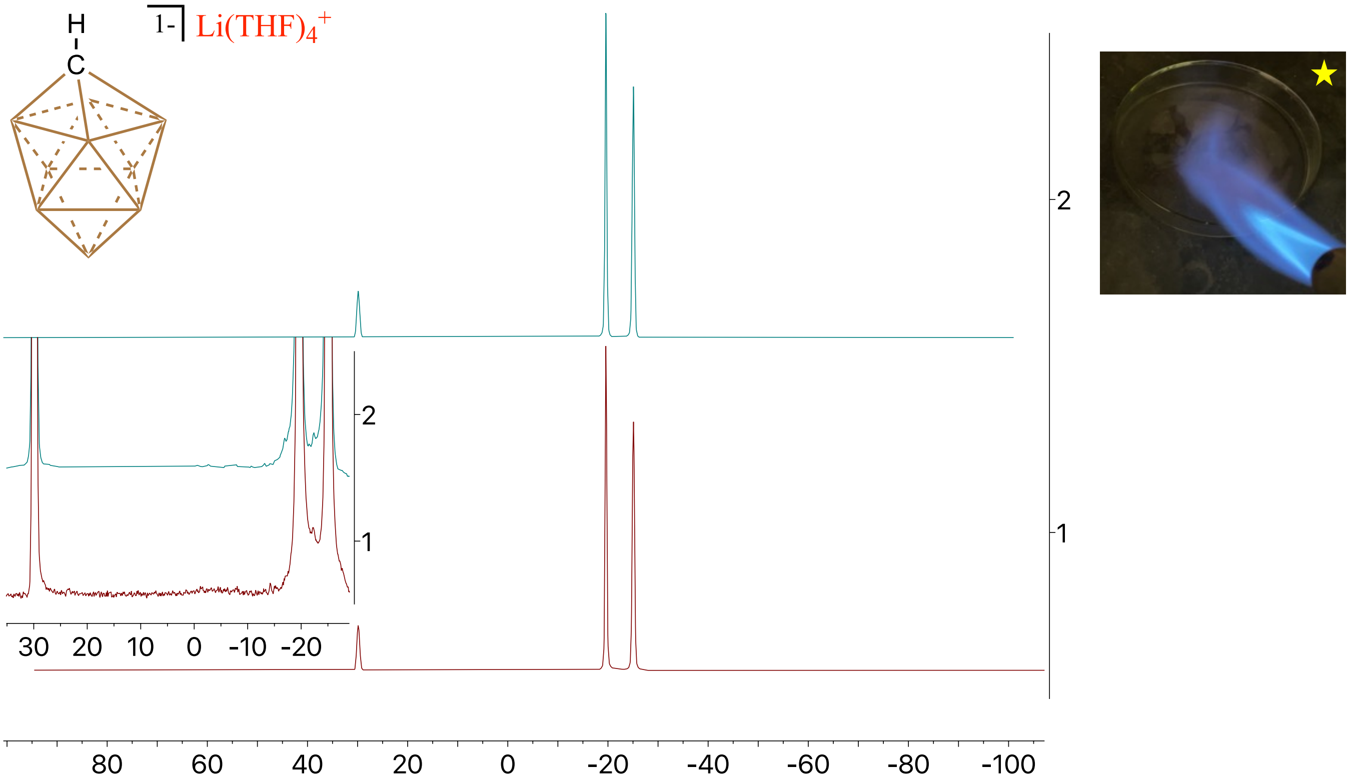

Figure S29. ^{11}B{^{1}H} NMR of **[Li(THF)$_4^+$][HCB$_9$H$_9^{1-}$]** before (above) and after (below) burning 3s in d$_6$-acetone.

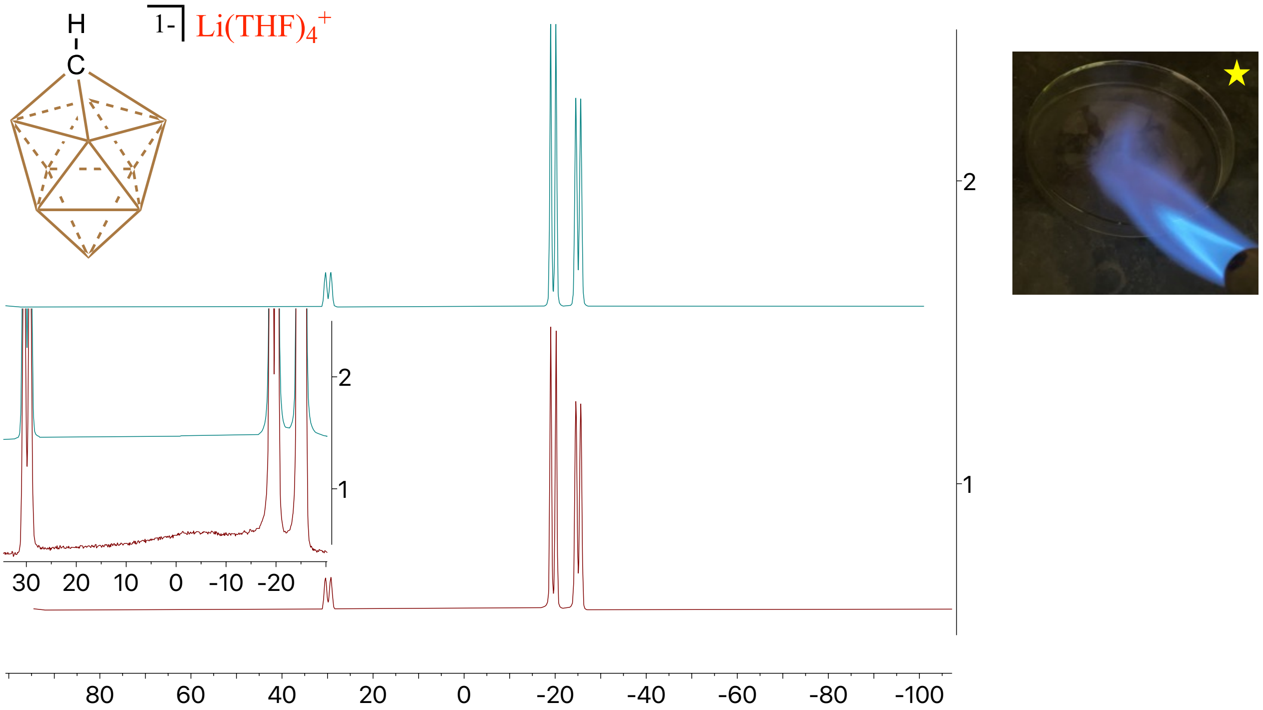

Figure S30. ^{11}B NMR of **[Li(THF)$_4$$^+$][HCB$_9H_9$$^{1-}$]** before (above) and after (below) burning 3s in d$_6$-acetone.

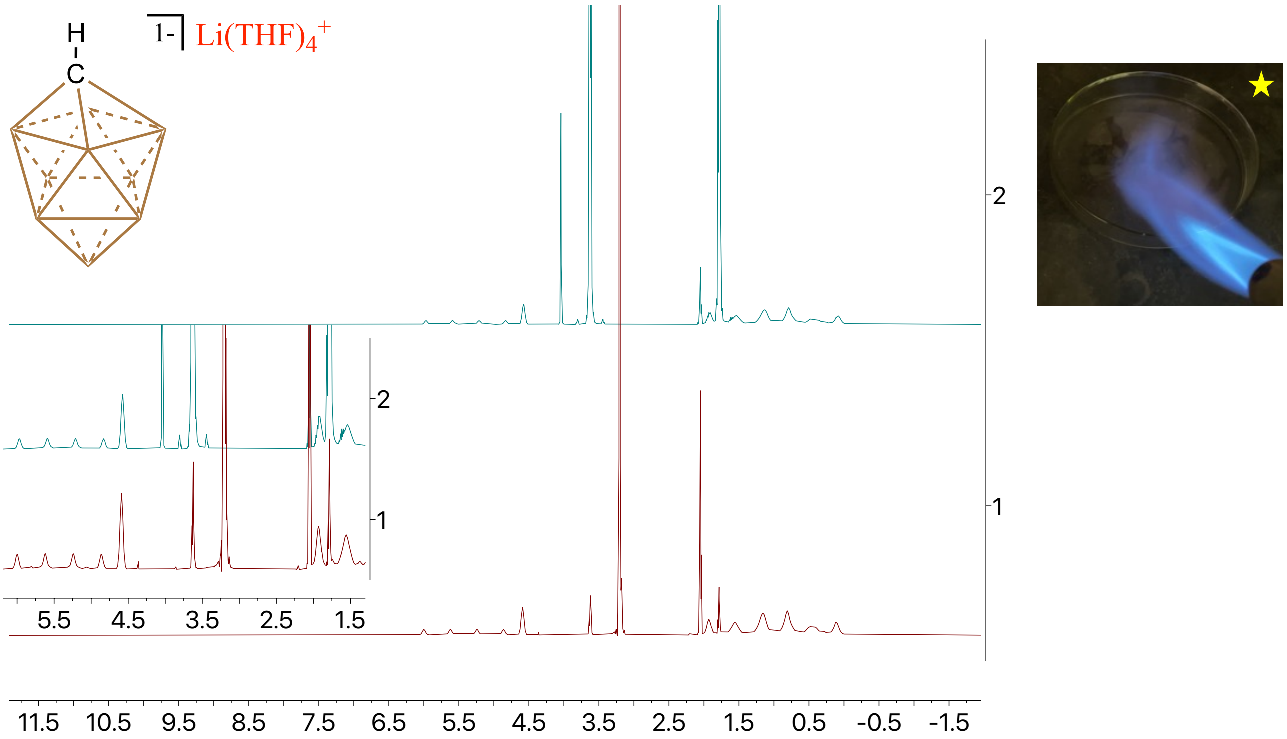

Figure S31. ^{1}H NMR of **[Li(THF)$_4$$^+$][HCB$_9H_9$$^{1-}$]** before (above) and after (below) burning 3s in d$_6$-acetone. Note: water peaks arise at 4.0 (above) and 3.2 ppm (below).

Synthesis of [Na(THF)$_n^+$][$HCB_9H_9^{1-}$] where n = 0, 2 – Figures S32 – S45

136 mg of **[$HNMe_3^+$][$HCB_9H_9^{1-}$]** was dried under vacuum at 160 °C prior to bringing into the glovebox. Excess addition (1.5 equiv.) of NaH immediately effervesced $H_{2(g)}$ and $NMe_{3(g)}$, but was allowed to stir overnight to ensure reaction completeness. The solution was filtered twice through glass microfiber filter papers and subsequently dried under vacuum until solid to yield **[$Na(THF)_2^+$][$HCB_9H_9^{1-}$]** (m.p. = 98.2 – 99.5 °C) (yield: 191 mg, 88%). Further desolvation at 150 °C for 18 hours yields the THF-free **[Na^+][$HCB_9H_9^{1-}$]** (m.p. > 400°C).

n = 0. $^{11}B\{^1H\}$ NMR (128 MHz, d_6-acetone) δ 30.69 (s, 1 B-H), -18.80 (s, 4 B-H), -24.31 (s, 4 B-H). ^{11}B NMR (128 MHz, d_6-acetone) δ 30.68 (d, *J* = 150.5 Hz, 1 B-H), -18.83 (d, *J* = 148.6 Hz, 4 B-H), -24.31 (d, *J* = 136.0 Hz, 4 B-H). ^1H NMR (400 MHz, d_6-acetone) δ 5.43 (q, *J* = 151.6 Hz, 1 B-H), 4.59 (s, 1H), 1.00 (td, *J* = 294.5, 143.2 Hz, 8 B-H). ^{23}Na NMR (106 MHz, d_6-acetone) δ -7.79.
n = 2. $^{11}B\{^1H\}$ NMR (128 MHz, d_6-acetone) δ 29.93 (s, B-H), -19.58 (s, 4 B-H), -25.06 (s, 4 B-H). ^{11}B NMR (128 MHz, d_6-acetone) δ 29.94 (d, *J* = 149.3 Hz, B-H), -19.57 (d, *J* = 149.3 Hz, 4 B-H), -25.04 (d, *J* = 137.0 Hz, 4 B-H). ^1H NMR (400 MHz, d_6-acetone) δ 5.44 (q, *J* = 151.6 Hz, 1 B-H), 4.59 (s, 1 B-H), 3.62 (m, 8H), 1.78 (m, 8H), 1.01 (td, *J* = 293.2, 142.5 Hz, 8 B-H). ^{23}Na NMR (106 MHz, d_6-acetone) δ -5.95 (s).

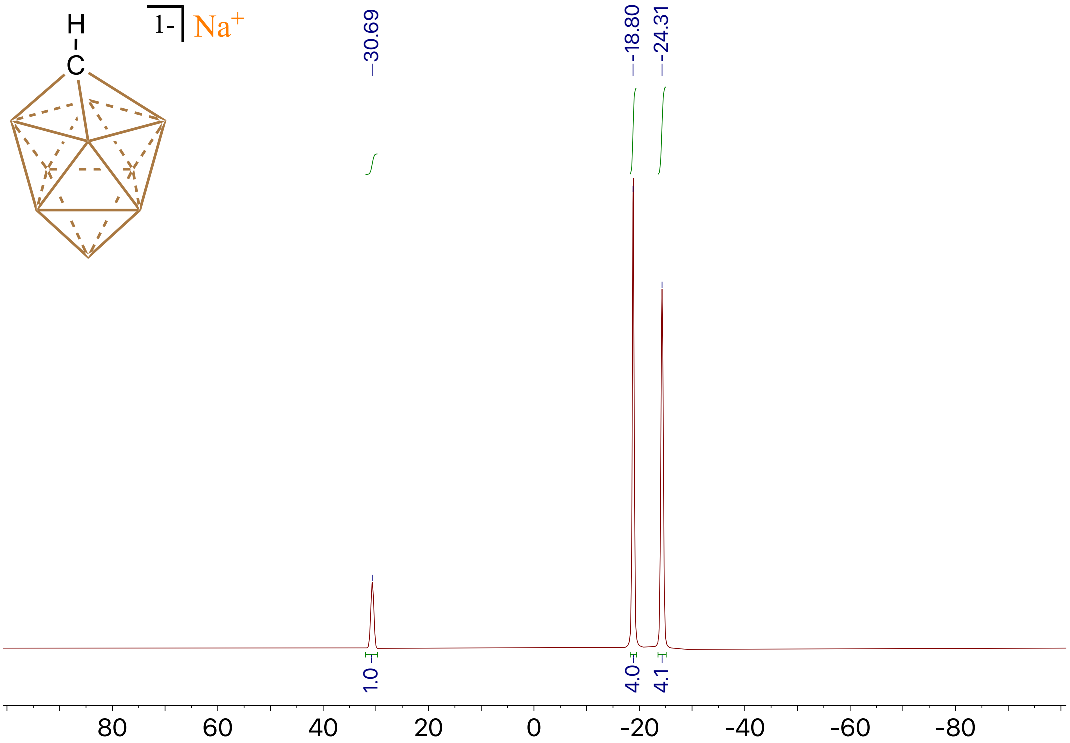

Figure S32. $^{11}B\{^1H\}$ NMR of **[Na^+][$HCB_9H_9^{1-}$]** in d_6-acetone.

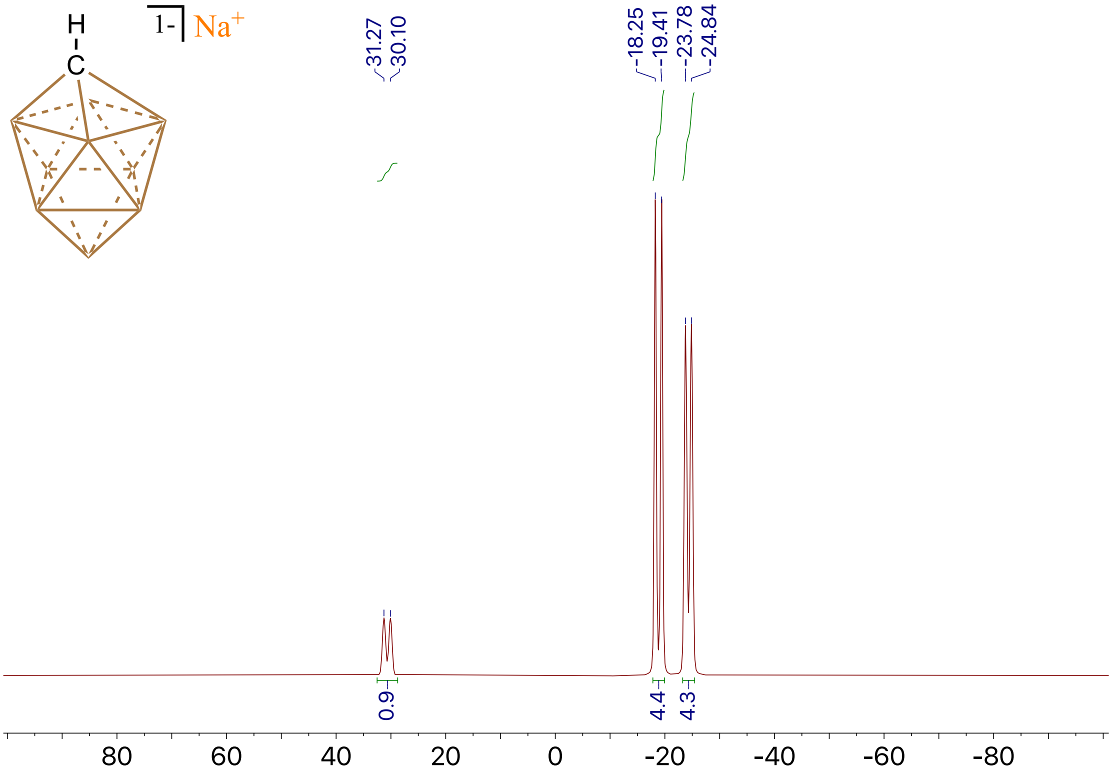

Figure S33. ^{11}B NMR of **[Na^{+}][HCB$_9$H$_9$$^{1-}$]** in d$_6$-acetone.

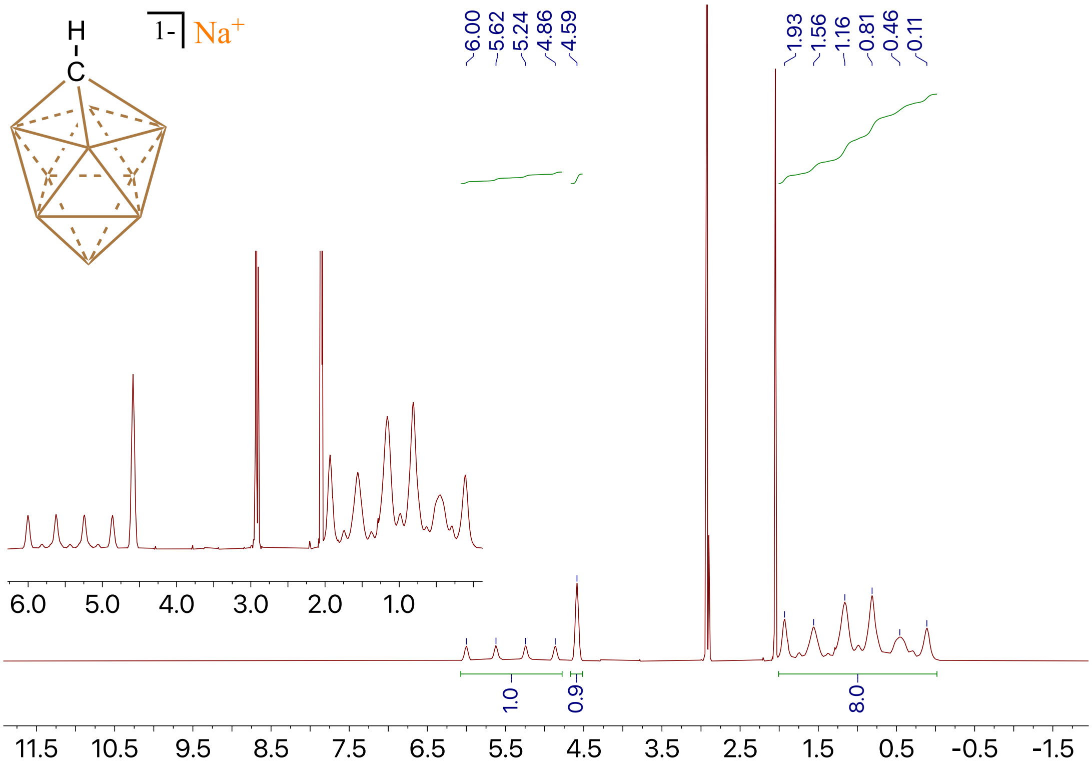

Figure S34. ^{1}H NMR of **[Na^{+}][HCB$_9$H$_9$$^{1-}$]** in wet d$_6$-acetone.

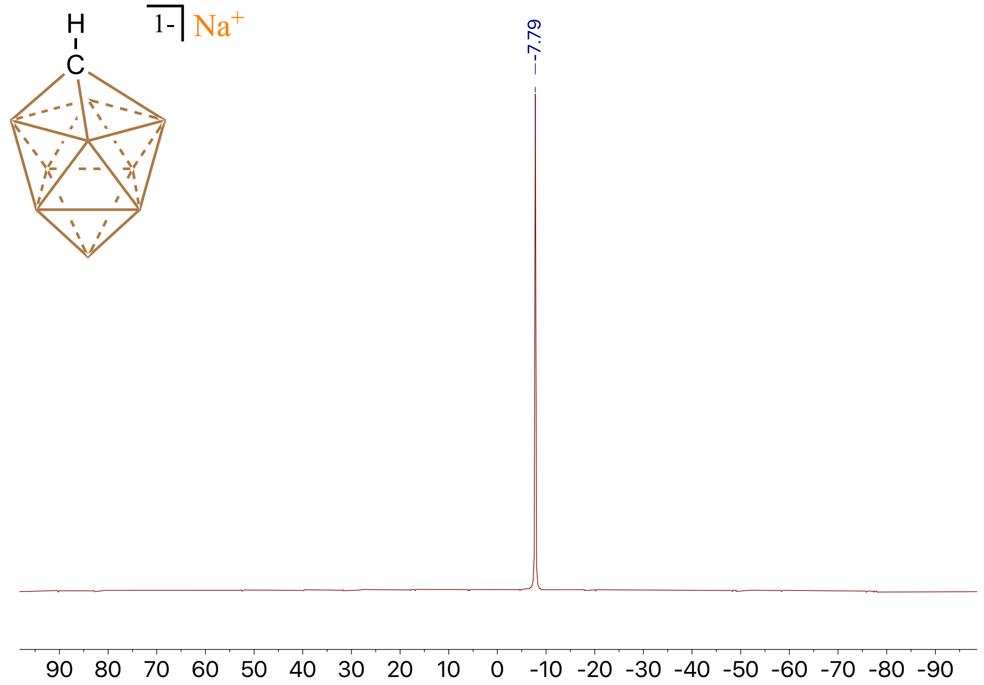

Figure S35. ^{23}Na NMR of **[Na^{+}][HCB$_9$H$_9$$^{1-}$]** in d$_6$-acetone.

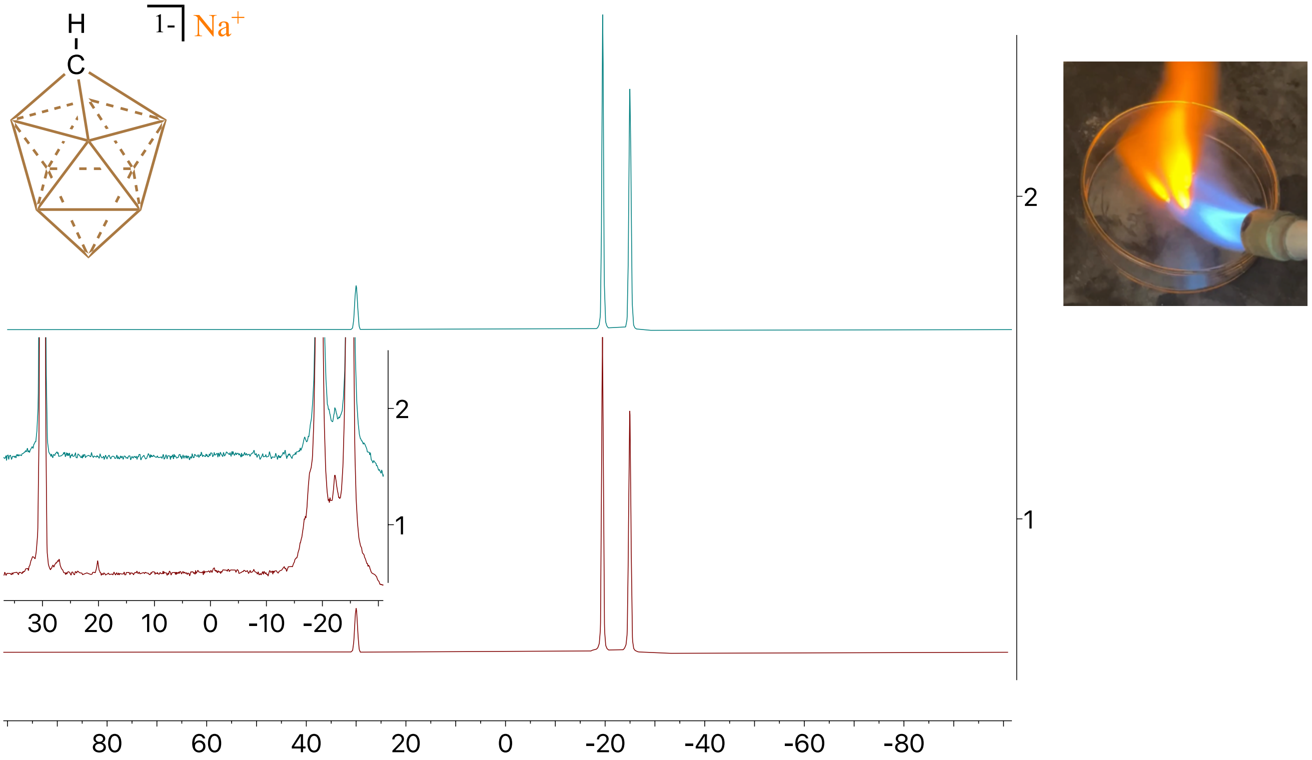

Figure S36. ^{11}B{^{1}H} NMR of **[Na^{+}][HCB$_9$H$_9$$^{1-}$]** before (above) and after (below) burning 3s in d$_6$-acetone. Minor decomposition into boric acid observed at 20 ppm.

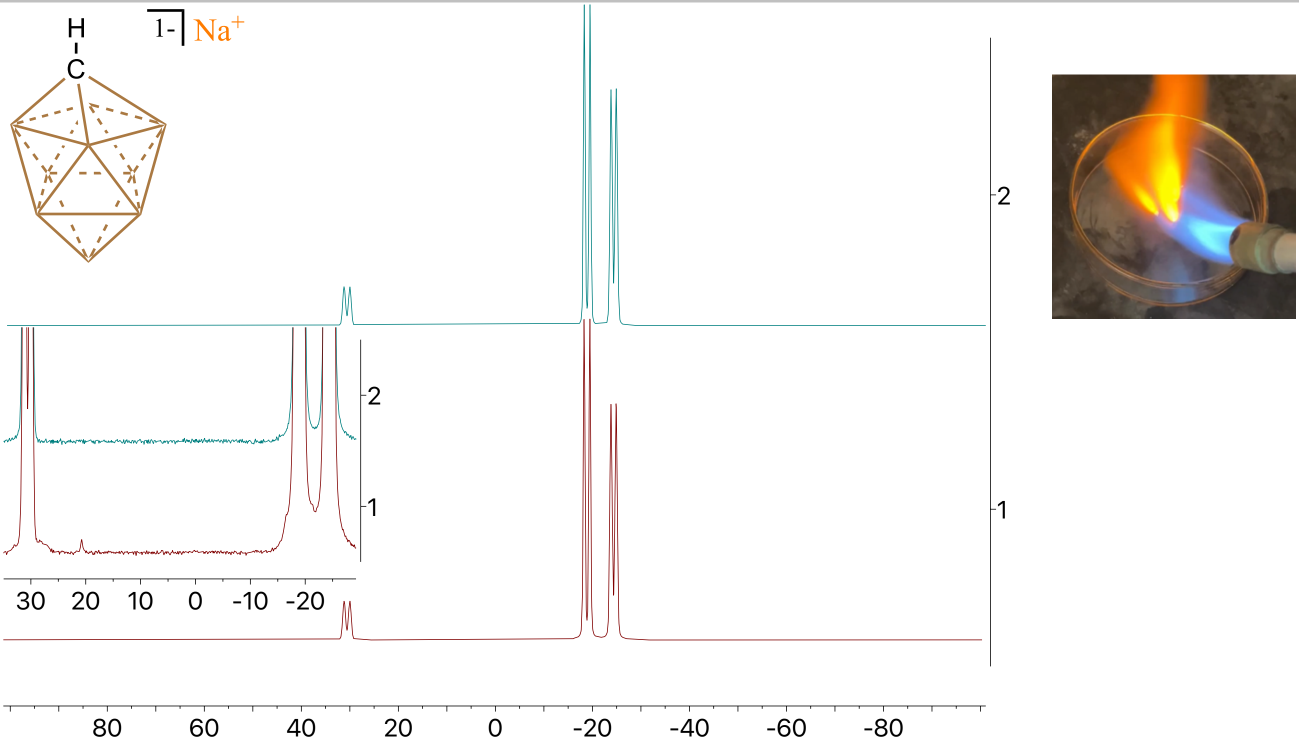

Figure S37. ^{11}B NMR of **$[Na^+][HCB_9H_9{}^{1-}]$** before (above) and after (below) burning 3s in d_6-acetone. Minor decomposition into boric acid observed at 20 ppm

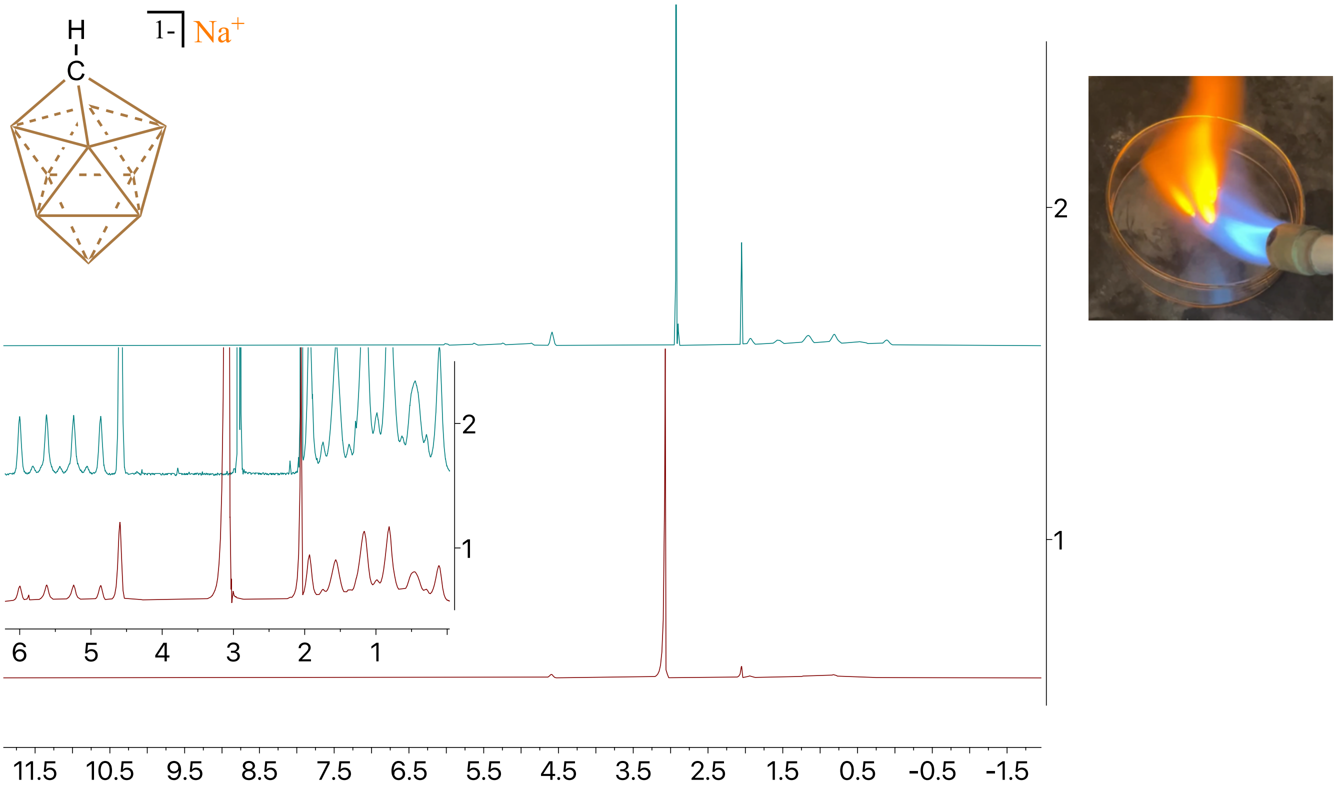

Figure S38. ^{1}H NMR of **$[Na^+][HCB_9H_9{}^{1-}]$** before (above) and after (below) burning 3s in d_6-acetone. Note: water peaks arise at 2.8 (above) and 3.1 ppm (below).

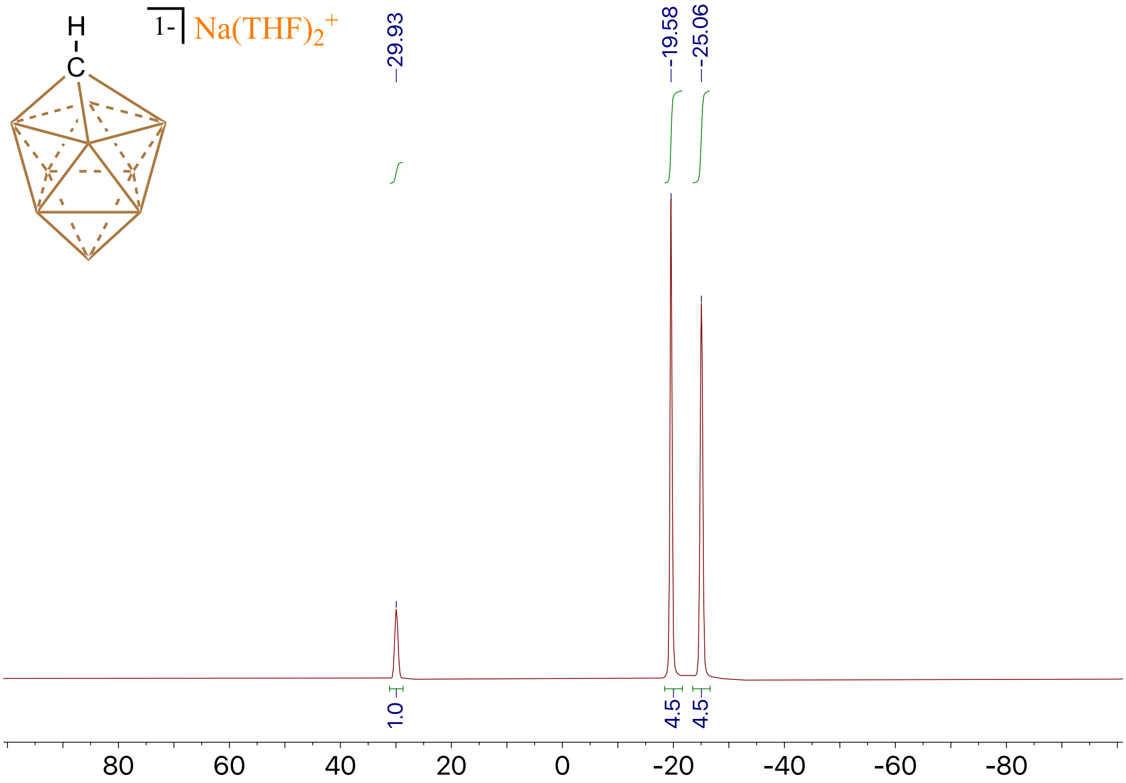

Figure S39. $^{11}B\{^{1}H\}$ NMR of **[Na(THF)$_2^+$][HCB$_9$H$_9^{1-}$]** in wet d$_6$-acetone.

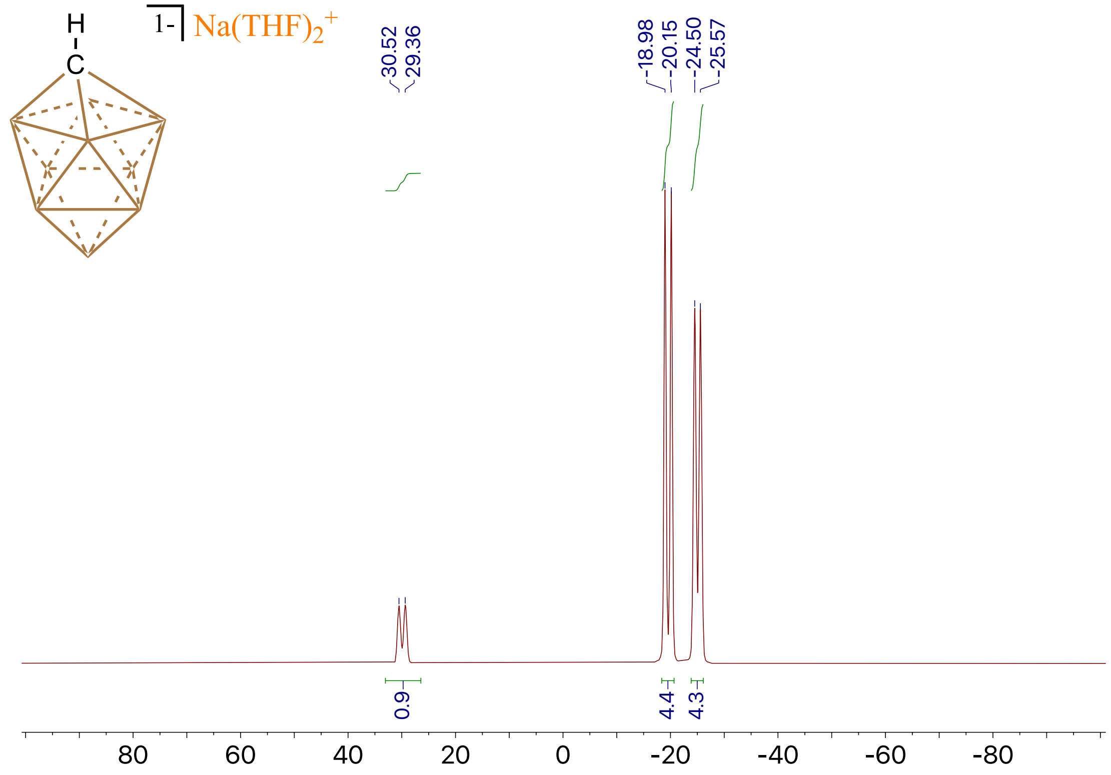

Figure S40. ^{11}B NMR of **[Na(THF)$_2^+$][HCB$_9$H$_9^{1-}$]** in wet d$_6$-acetone.

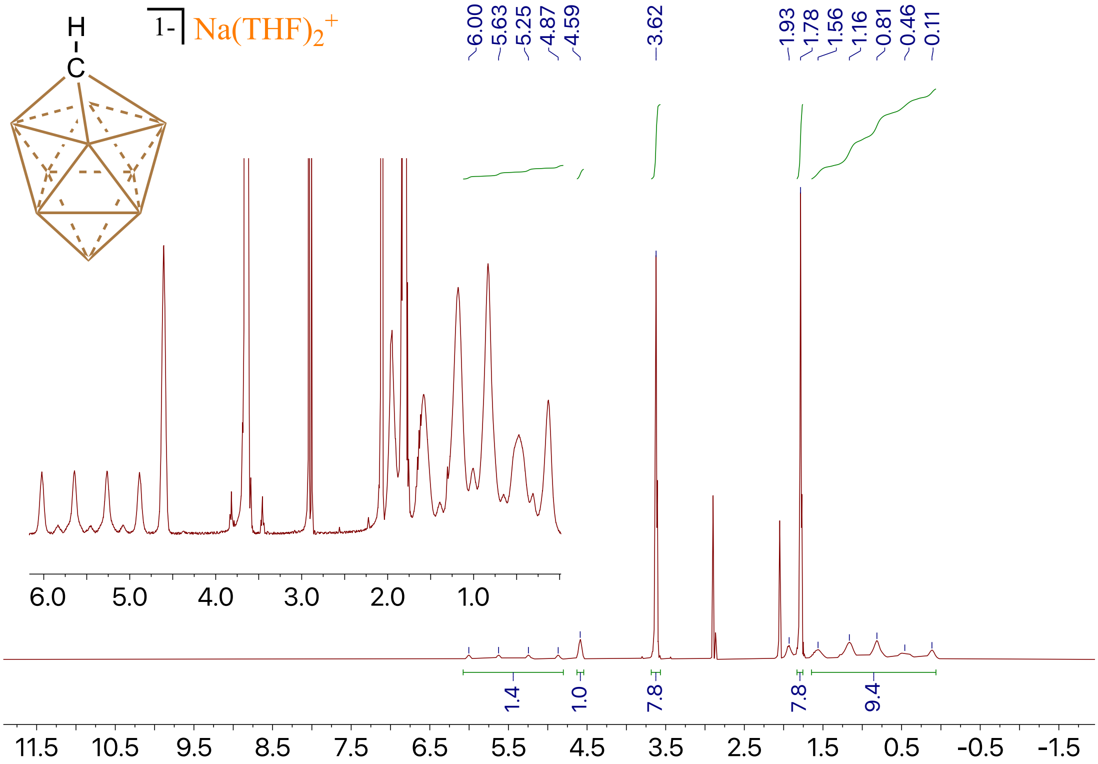

Figure S41. ^{1}H NMR of **$[Na(THF)_2^+][HCB_9H_9^{1-}]$** in wet d_6-acetone. Note: residual water from solvent appears at 2.87 ppm.

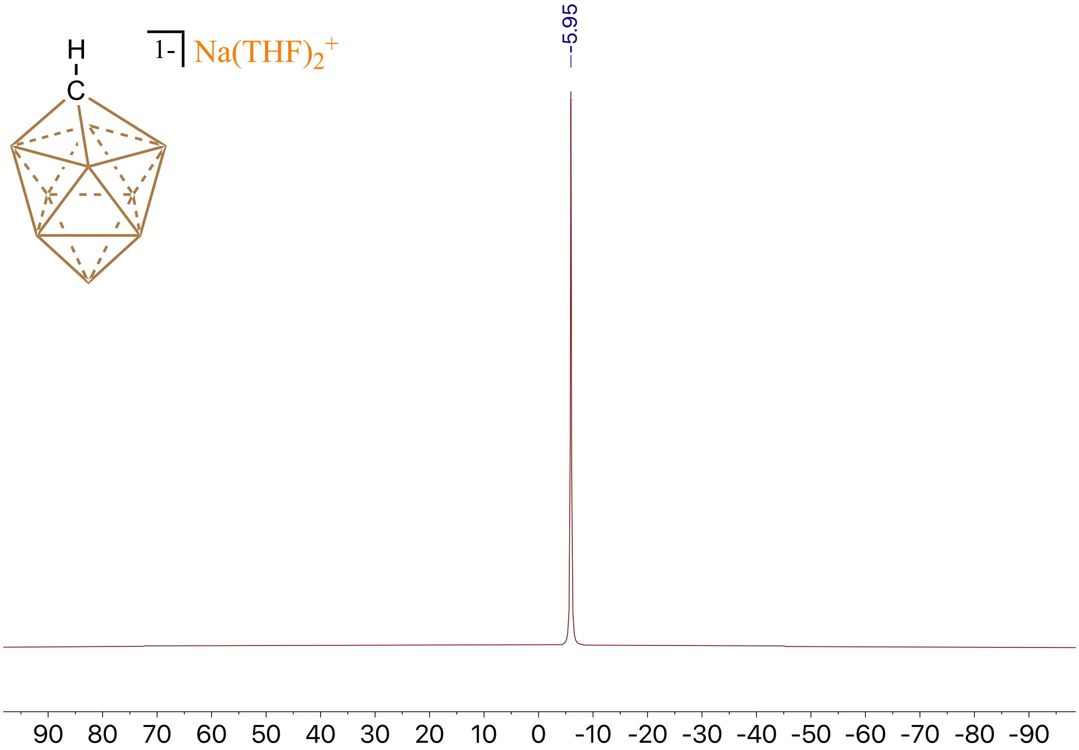

Figure S42. ^{23}Na NMR of **$[Na(THF)_2^+][HCB_9H_9^{1-}]$** in wet d_6-acetone.

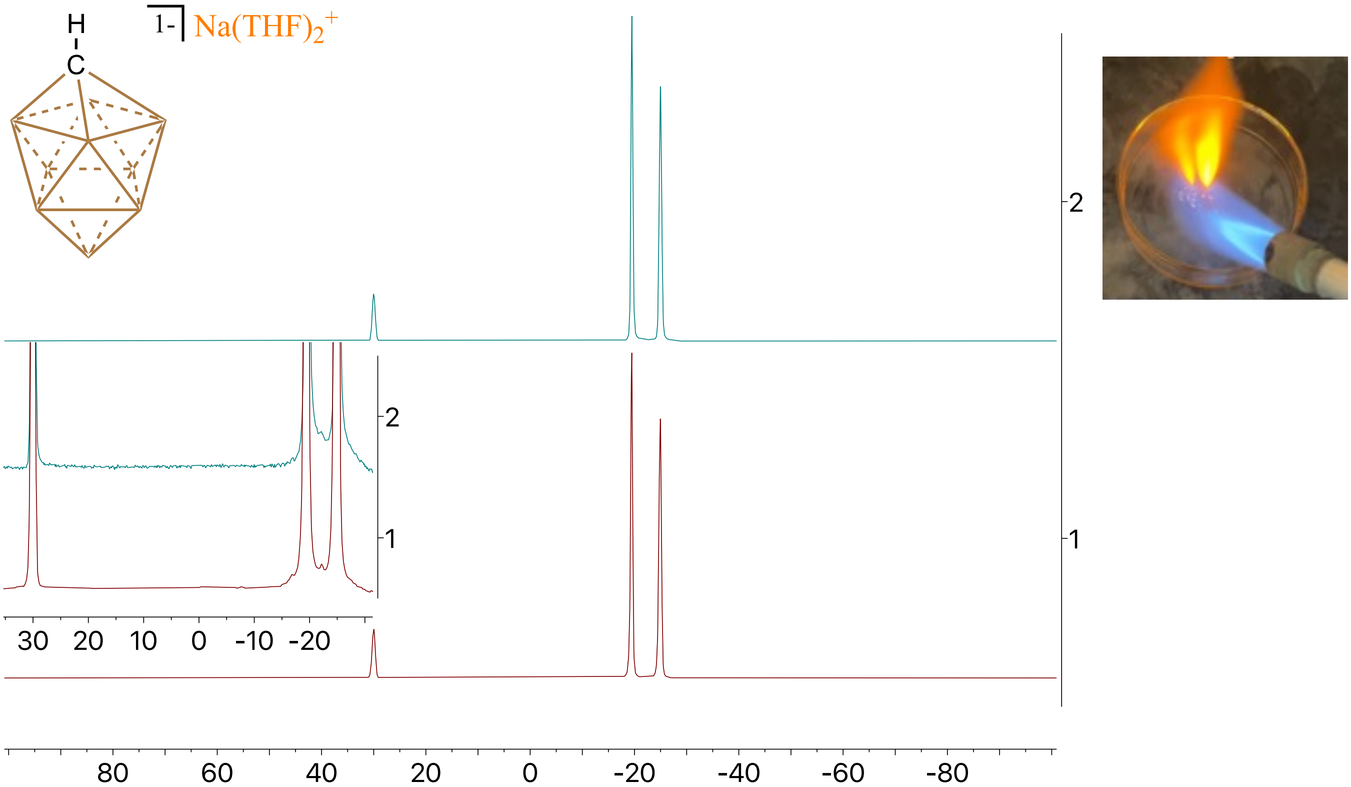

Figure S43. $^{11}B\{^{1}H\}$ NMR of **$[Na(THF)_2^+][HCB_9H_9^{1-}]$** before (above) and after (below) burning 3s in d_6-acetone.

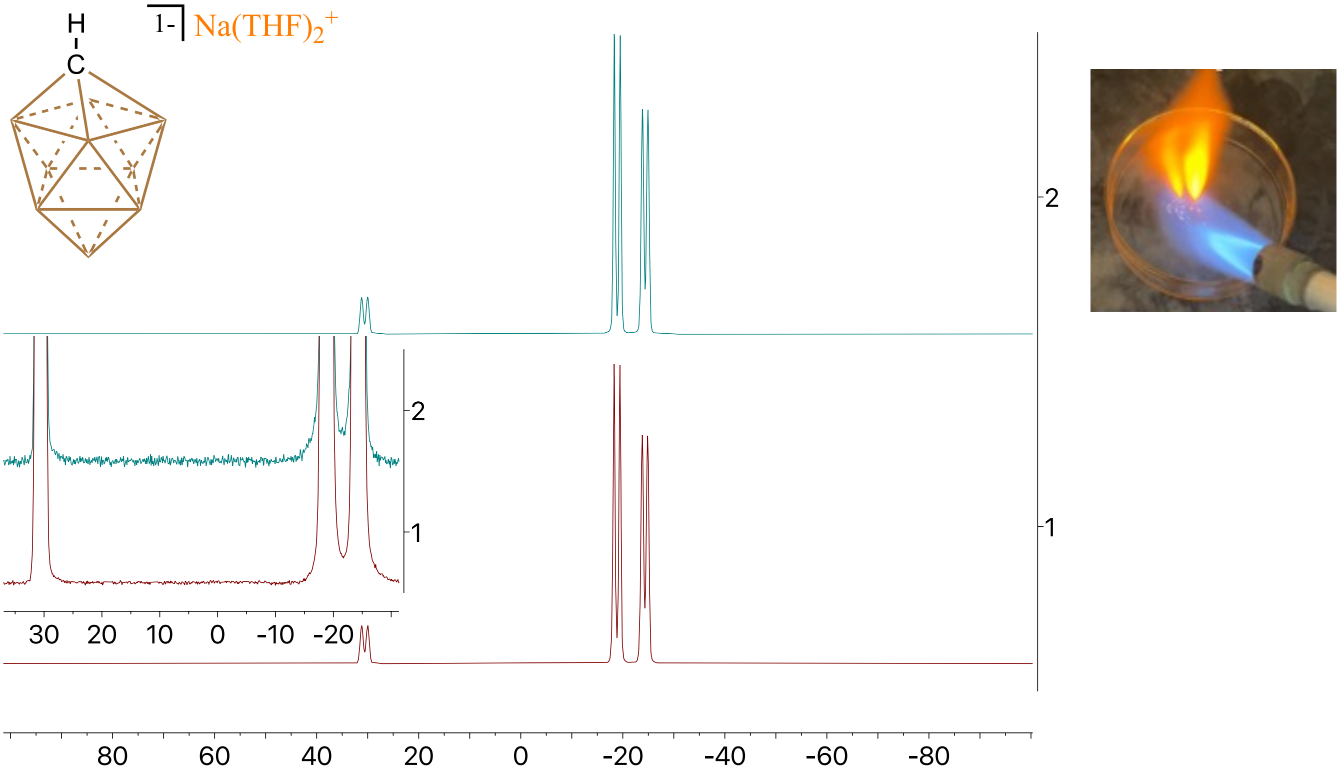

Figure S44. ^{11}B NMR of **$[Na(THF)_2^+][HCB_9H_9^{1-}]$** before (above) and after (below) burning 3s in d_6-acetone.

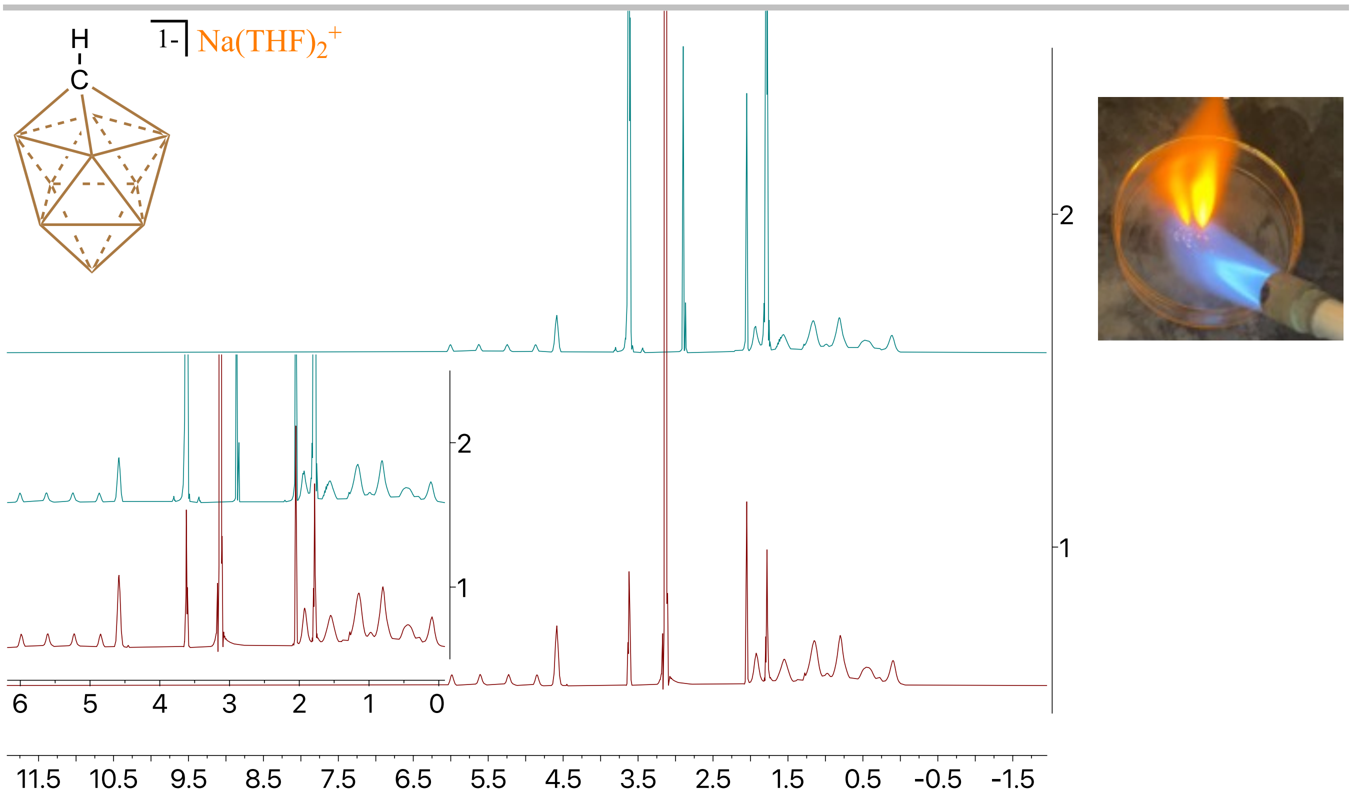

Figure S45. ^{1}H NMR of **[Na(THF)$_2^+$][HCB$_9$H$_9^{1-}$]** before (above) and after (below) burning 3s in d$_6$-acetone. Note: water peaks arise at 2.8 (above) and 3.1 ppm (below).

Synthesis of [K$^+$][HCB$_9$H$_9^{1-}$] – Figures S46 – S51

116 mg **[HNMe$_3^+$][HCB$_9$H$_9^{1-}$]** was dried under vacuum at 160 °C prior to bringing into the glovebox. The addition of 1.1 molar equiv. KHMDS (95%, Sigma-Aldrich) was stirred for 2 hours and the solvent was subsequently removed at room temperature. THF-free **[K$^+$][HCB$_9$H$_9^{1-}$]** was then recrystallized from a mixture of Et_2O and hexanes (v/v 1:2) at -30 °C, filtered while cold, and dried under vacuum at room temperature (yield = 59 mg, 58%, m.p. > 400 °C).

$^{11}B\{^{1}H\}$ NMR (128 MHz, d$_6$-acetone) δ 30.85 (s, B-H), -18.62 (s, 4 B-H), -24.10 (s, 4 B-H). ^{11}B NMR (128 MHz, d$_6$-acetone) δ 30.84 (d, J = 150.9 Hz, 1 B-H), -18.64 (d, J = 149.3 Hz, 4 B-H), -24.12 (d, J = 135.4 Hz, 4 B-H). ^{1}H NMR (400 MHz, d$_6$-acetone) δ 5.43 (q, J = 151.5 Hz, 1H), 4.59 (s, 1H), 1.10 (td, J = 294.5, 143.2 Hz, 8 B-H).

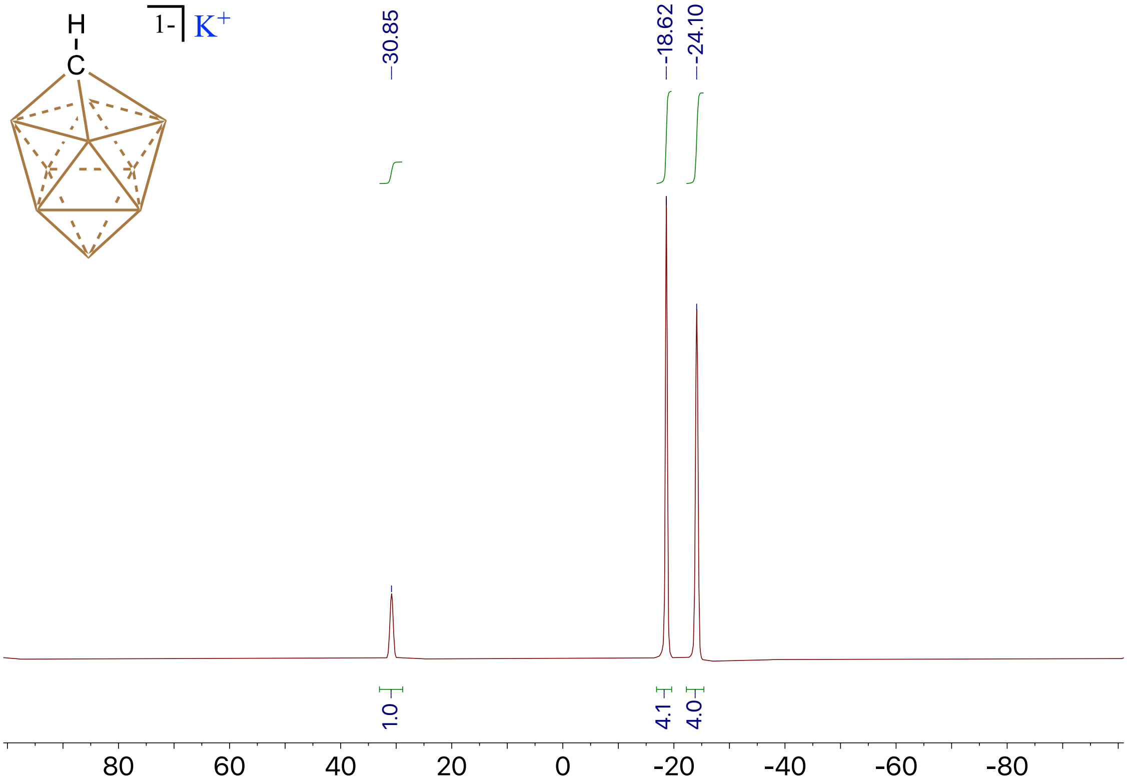

Figure S46. $^{11}B\{^1H\}$ NMR of **[K$^+$][HCB$_9$H$_9$$^{1-}$]** in wet d$_6$-acetone.

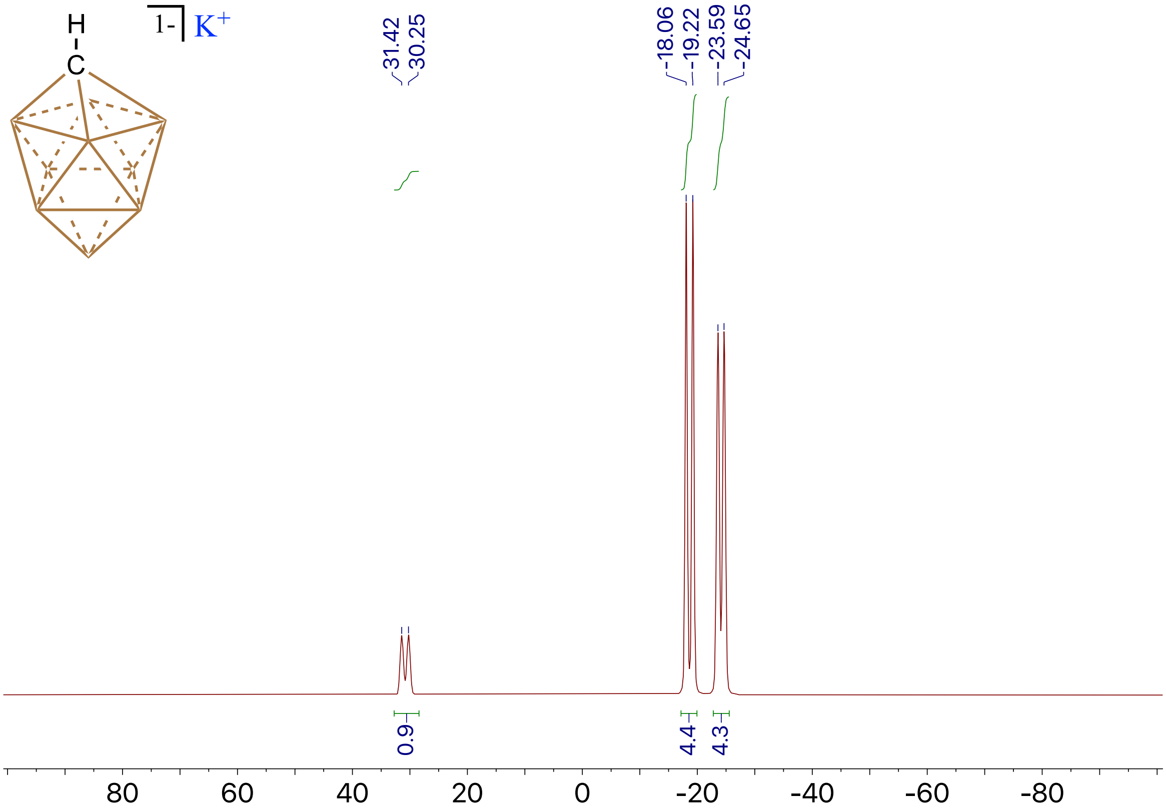

Figure S47. ^{11}B NMR of **[K$^+$][HCB$_9$H$_9$$^{1-}$]** in wet d$_6$-acetone.

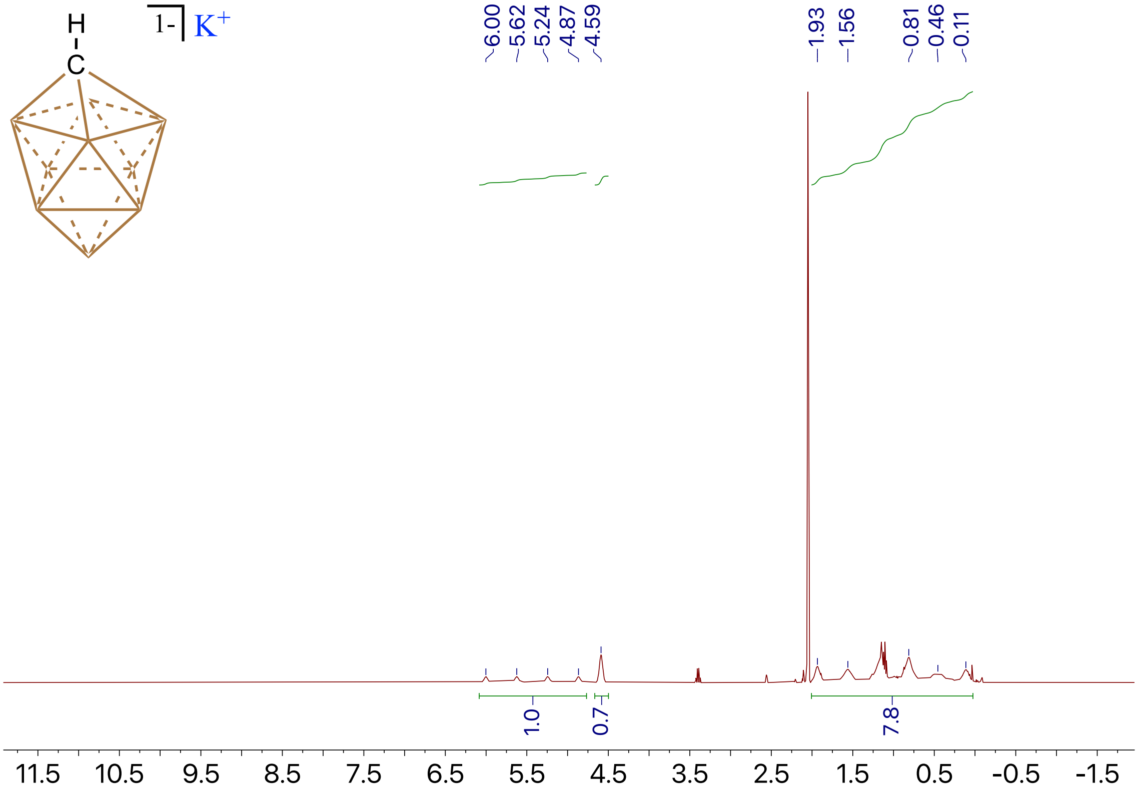

Figure S48. ^{1}H NMR of **[K$^+$][HCB$_9$H$_9$$^{1-}$]** in d$_6$-acetone. Note: a small amount of Et$_2$O is present from work-up at 1.10 and 3.41 ppm. Loss of HNMe$_3^+$ singlet (see Figure S10) indicates formation of K$^+$ salt.

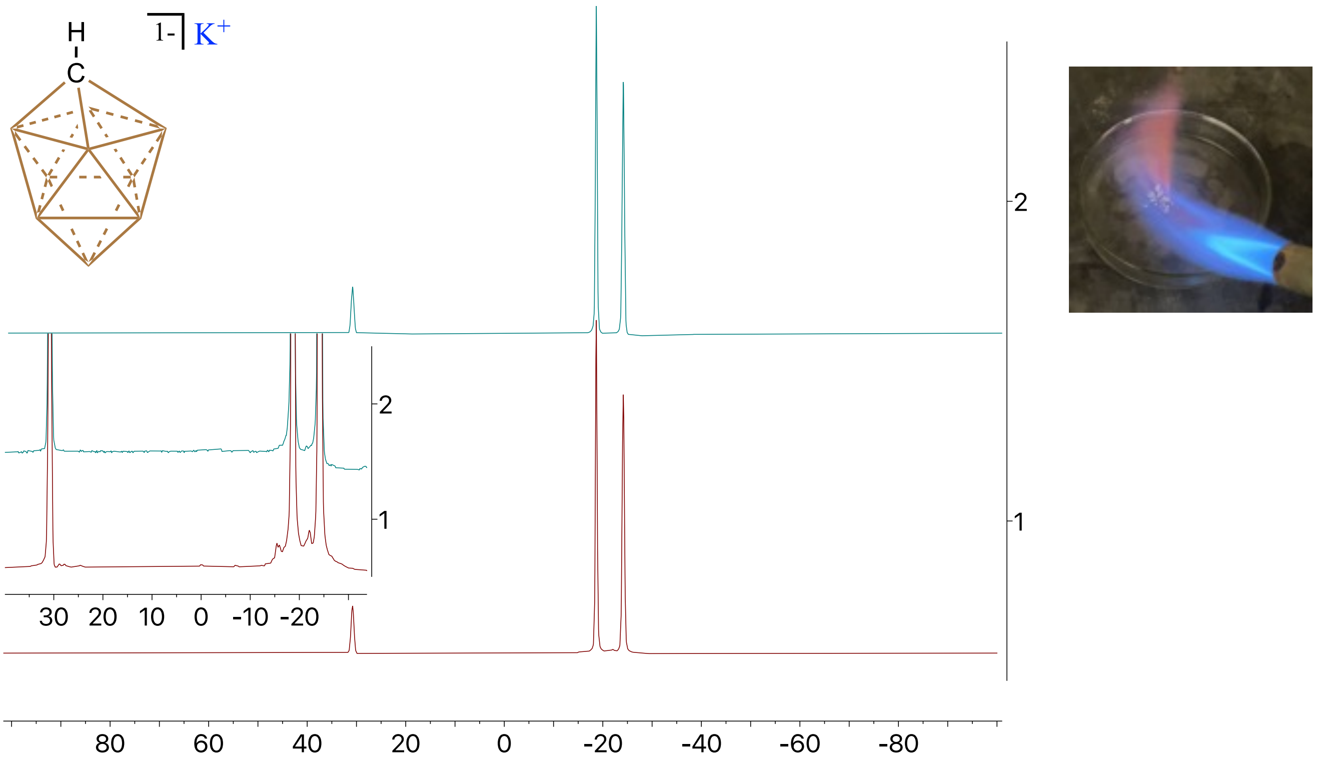

Figure S49. ^{11}B{^{1}H} NMR of **[K$^+$][HCB$_9$H$_9$$^{1-}$]** before (above) and after (below) burning 3s in d$_6$-acetone. Minor decomposition observed at 29, 0, -9, -15 and -22 ppm.

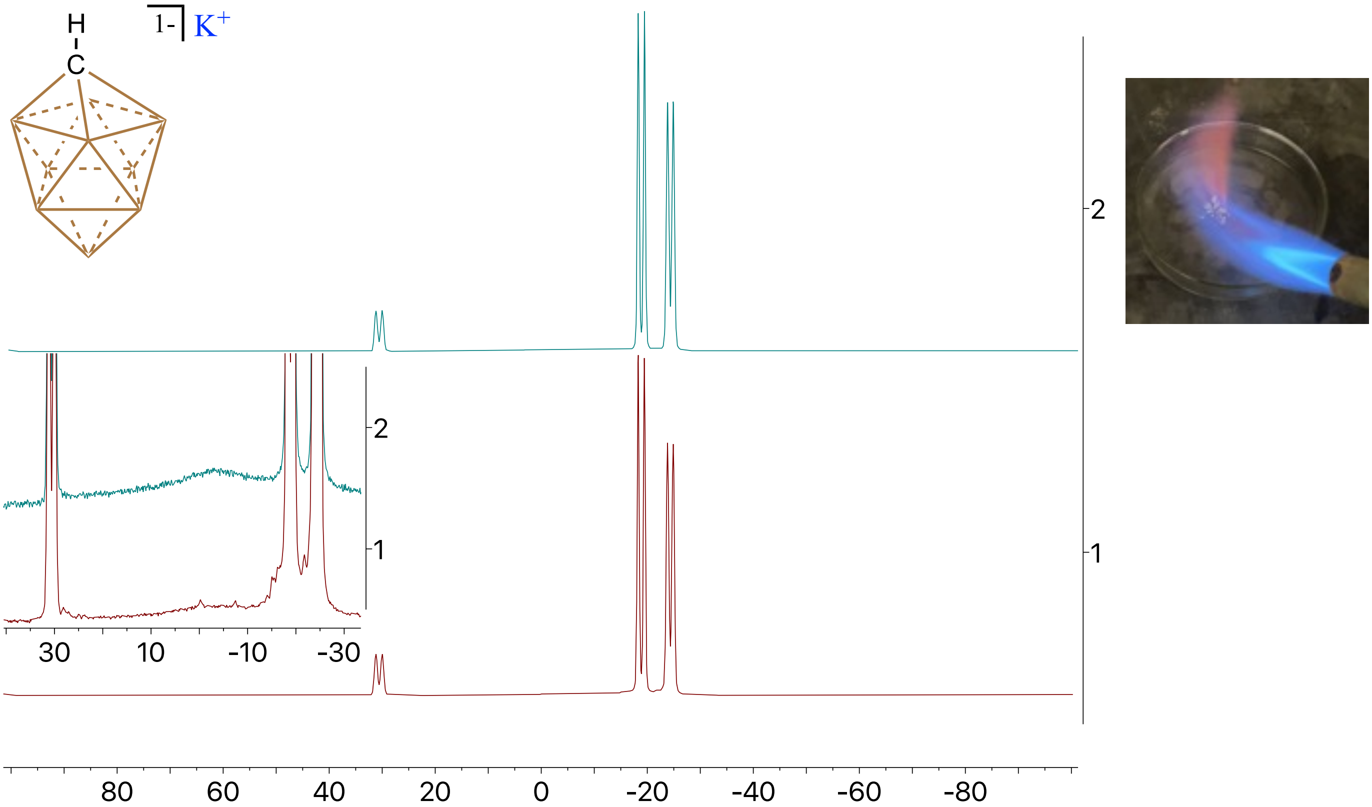

Figure S50. ^{11}B NMR of **[K$^+$][HCB$_9$H$_9$$^{1-}$]** before (above) and after (below) burning 3s in d$_6$-acetone. Minor decomposition observed at 29, 0, -9, -15 and -22 ppm.

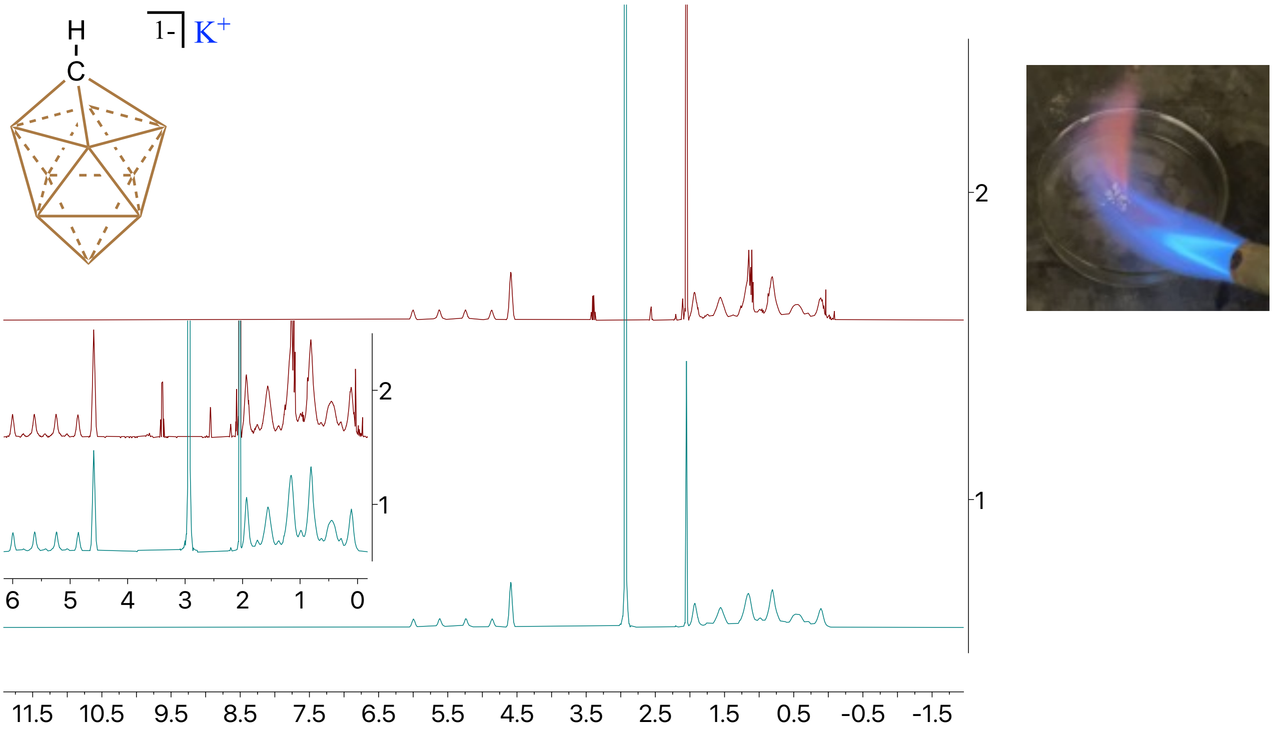

Figure S51. ^{1}H NMR of **[K$^+$][HCB$_9$H$_9$$^{1-}$]** before (above) and after (below) burning 3s in d$_6$-acetone. Note: water peaks arise at 2.8 (above) and 3.1 ppm (below). Note: a tiny amount of Et_2O remains present on (before) NMR from workup. H_2O appears in (bottom) at 2.9 ppm.

Synthesis of CsC$_4$ ([Cs^+][$C_4H_9CB_9H_9^{1-}$]) **from [HNMe$_3^+$][HCB$_9$H$_9^{1-}$]** – Figures S52 – S57

[Cs^+][$C_4H_9CB_9H_9^{1-}$] was prepared from a modified literature procedure.[2] Working in a glovebox, 12.1 g (67.4 mmol) **[$HNMe_3^+$][$HCB_9H_9^{1-}$]** was dissolved in 200 mL of THF. In a separate container, 59.3 mL (148.3 mmol, 2.2 eq.) of 2.5 M n-BuLi solution in hexane was concentrated in-vacuo. The n-BuLi was added dropwise to the stirring THF solution until the effervescence of NMe_3 and H_2 seized. The solution was allowed to react for an additional 30 minutes before confirming full conversion of **[$HNMe_3^+$][$HCB_9H_9^{1-}$]** to **[$2Li^+$][$CB_9H_9^{2-}$]** by ^{11}B NMR. The solution of **[$2Li^+$][$CB_9H_9^{2-}$]** was then added dropwise to a stirring solution of pentane where a white precipitate immediately formed. The pentane solution was allowed to settle for 10 minutes following the complete addition of the THF solution and was decanted. The slurry comprised mainly of **[$2Li^+$][$CB_9H_9^{2-}$]** was dried in-vacuo to remove the remaining pentane. 8.7 mL (80 mmol, 1.2 eq) 1-bromo-butane was added to the dry **[$2Li^+$][$CB_9H_9^{2-}$]** followed by 100 mL of THF. The reaction was left to stir for 12 hours. Following reaction completion, an addition funnel was charged with the THF solution containing **[Li^+][$C_4H_9CB_9H_9^{1-}$]** and added dropwise to a stirring 600 mL solution of pentane. 200 mL of H_2O was added followed by the addition of 20.0 g of CsCl. The biphasic solution was allowed to stir for 15 minutes before the solution was filtered yielding **[Cs^+][$C_4H_9CB_9H_9^{1-}$]** as a white powder. The compound was recrystallized from 500 mL of boiling water producing 18.9 g (91.0 % yield) **[Cs^+][$C_4H_9CB_9H_9^{1-}$]** in high purity (m.p. = 282.2 – 285.8 °C).

^{11}B{^{1}H} NMR (128 MHz, d$_6$-acetone) δ 26.82 (s, 1 B-H), -15.93 (s, 4 B-H), -23.68 (s, 4 B-H). ^{11}B NMR (128 MHz, d$_6$-acetone) δ 26.68 (d, *J* = 149.1 Hz, 1 B-H), -16.10 (d, *J* = 147.6 Hz, 4 B-H), -23.85 (d, *J* = 136.7 Hz, 4 B-H). ^{1}H NMR (400 MHz, d$_6$-acetone) δ 5.22 (q, *J* = 150.8 Hz, 1 B-H), 3.32 – 2.98 (m, 2H), 2.06 – 1.93 (m, 2H), 1.56 (h, *J* = 8.0 Hz, 2H), 1.03 (t, *J* = 7.3 Hz, 3H). ^{1}H{^{11}B} NMR (600 MHz, d$_6$-acetone) δ 5.21 (s, 1 B-H), 3.09 (m, 2H), 1.97 (m, 2H), 1.54 (h, *J* = 8.0 Hz, 2H), 1.45 (s, 4 B-H), 1.01 (t, *J* = 7.4 Hz, 3H), 0.65 (s, 4 B-H). HRMS: **1$_{butyl}$** = [$C_4H_9CB_9H_9^{1-}$] Calculated: 176.22878 m/z. Experimental: 176.2292 m/z.

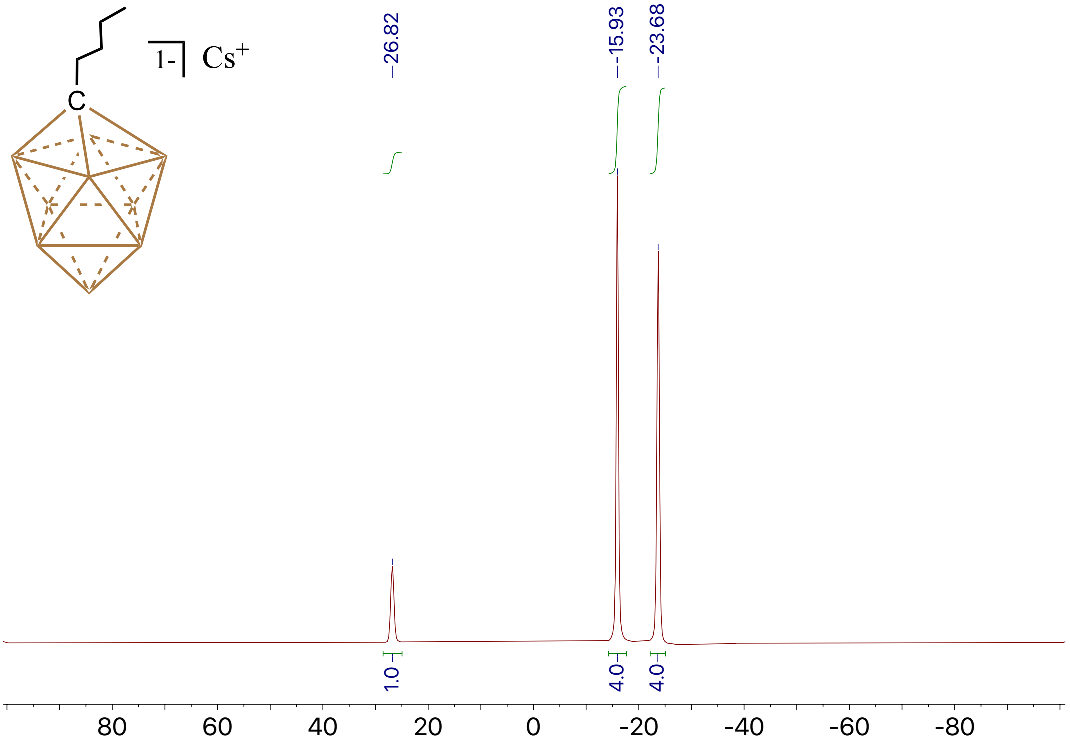

Figure S52. ^{11}B{^{1}H} NMR of **CsC$_4$** ([Cs^+][$C_4H_9CB_9H_9^{1-}$]) in wet d$_6$-acetone. Note: the C-alkylation causes a substantial shift in the antipodal and belt boron peaks compared to **[Cs^+][$HCB_9H_9^{1-}$]** seen in Figure S1.

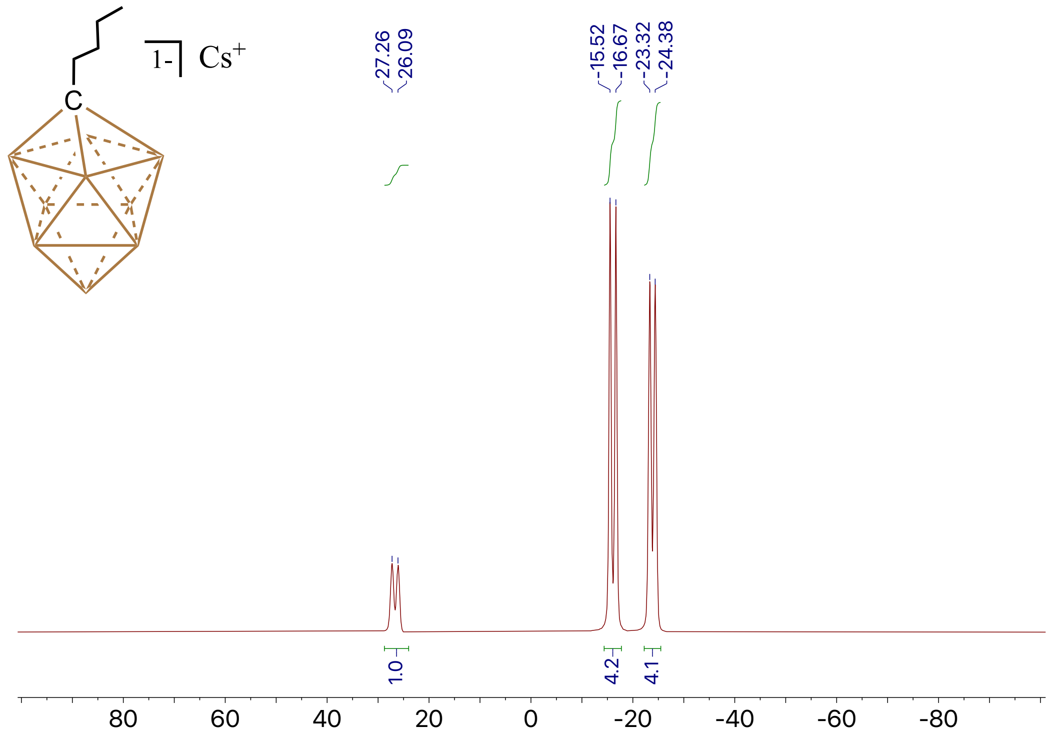

Figure S53. ^{11}B NMR of **CsC4** ($[Cs^+][C_4H_9CB_9H_9{}^{1-}]$) in wet d_6-acetone.

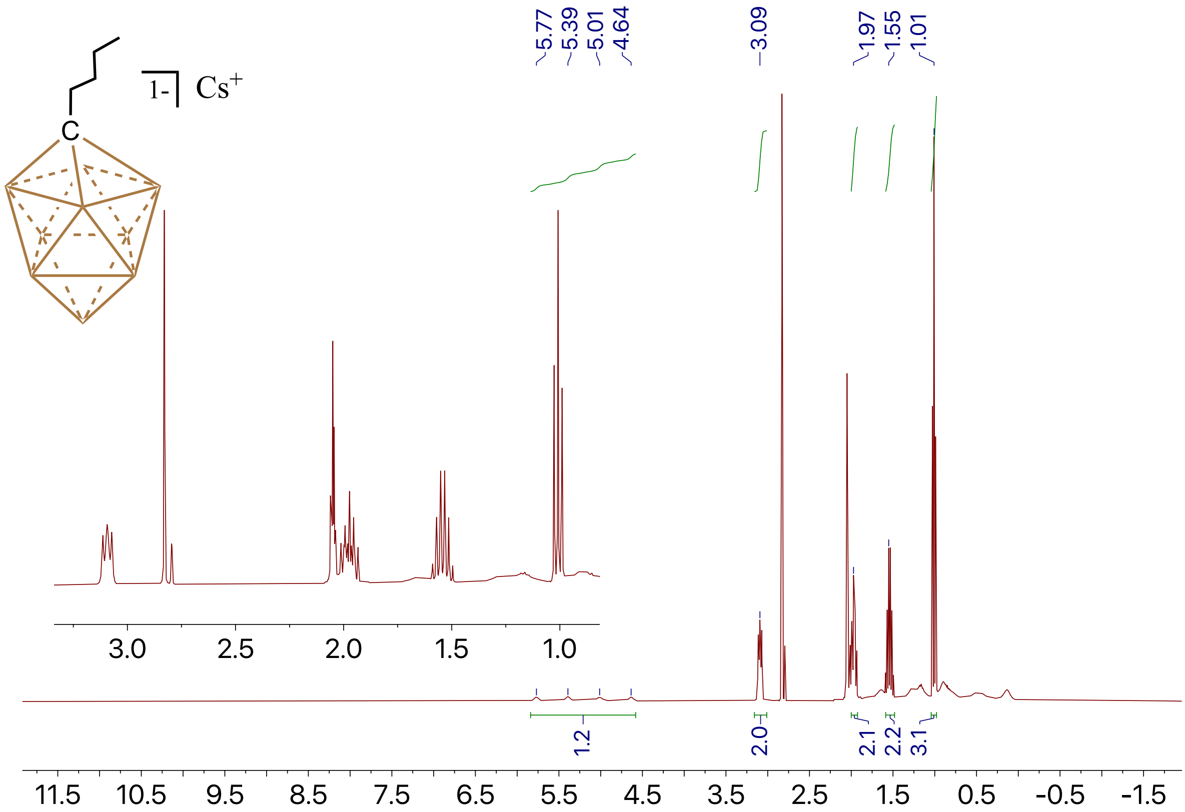

Figure S54. ^1H NMR of **CsC4** ($[Cs^+][C_4H_9CB_9H_9{}^{1-}]$) in wet d_6-acetone. Note: water from solvent appears at 2.82 ppm. Additionally, the alkylation causes the loss of the carborane C-H peak at 4.61 ppm seen in Figure S3.

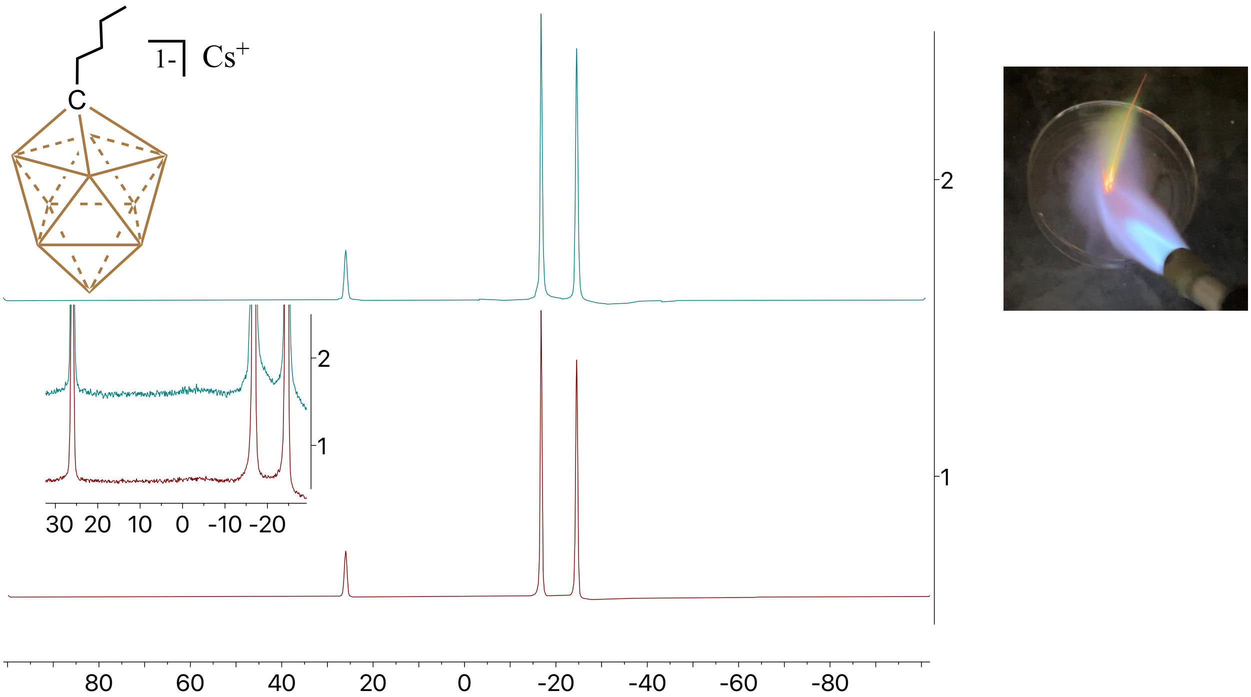

Figure S55. $^{11}B\{^{1}H\}$ NMR of **CsC$_4$** ($[Cs^+][C_4H_9CB_9H_9^{1-}]$) before (above) and after (below) burning 3s in d_6-acetone.

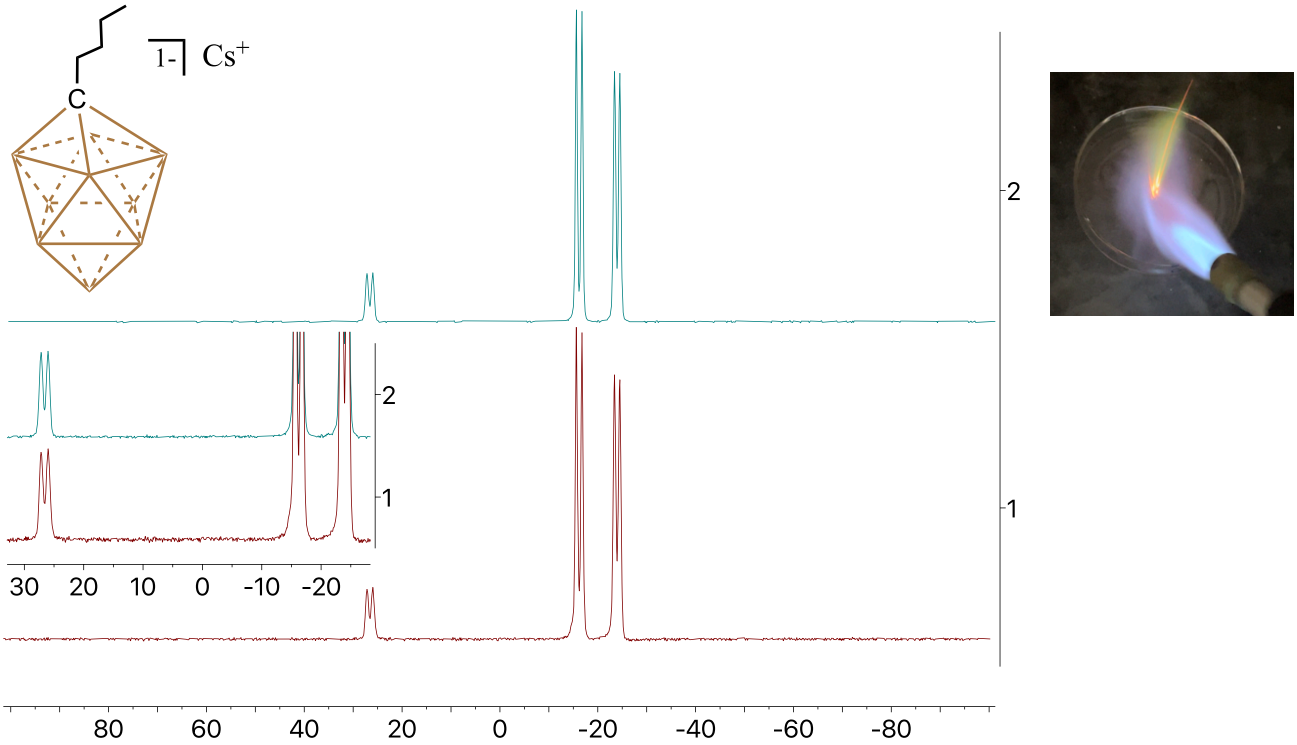

Figure S56. ^{11}B NMR of **CsC$_4$** ($[Cs^+][C_4H_9CB_9H_9^{1-}]$) before (above) and after (below) burning 3s in d_6-acetone.

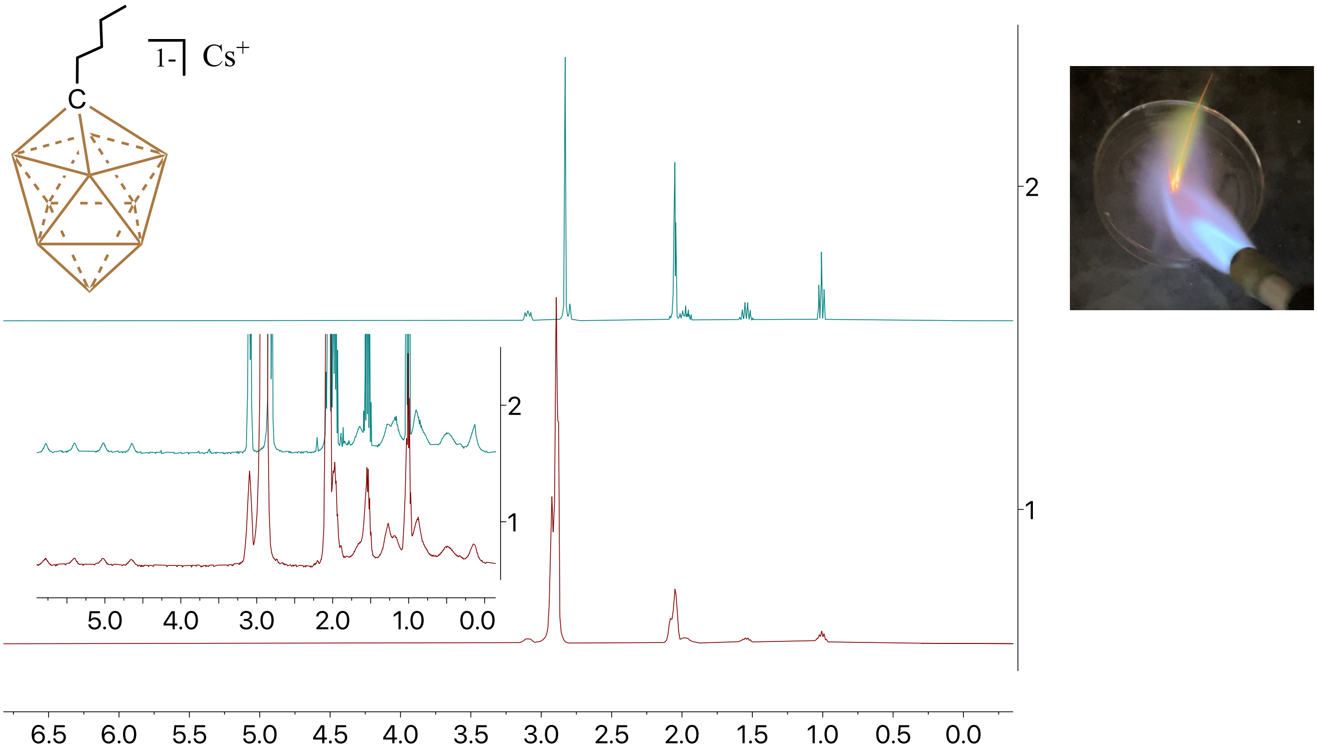

Figure S57. ^{1}H NMR of **CsC$_4$** ([Cs$^+$][$C_4H_9CB_9H_9^{1-}$]) before (above) and after (below) burning 3s in d$_6$-acetone. Note: water peaks arise at 2.8 ppm.

Synthesis of [HNMe$_3^+$][$C_4H_9CB_9H_9^{1-}$] from [Cs$^+$][$C_4H_9CB_9H_9^{1-}$] – Figures S58 – S61

18.9 g of **[Cs$^+$][$C_4H_9CB_9H_9^{1-}$]** and 25.0 g of HNMe$_3$Cl were suspended in a biphasic mixture of 200 mL CHCl$_3$ and 200 mL H_2O. The mixture was stirred vigorously for 20 minutes, or until no solid remained in either layer. The two layers were then separated and the aqueous layer was further extracted with more CHCl$_3$. Separation of the organic layer, followed by subsequent removal of the CHCl$_3$ under reduced pressure, yielded **[HNMe$_3^+$][$C_4H_9CB_9H_9^{1-}$]**. This salt was further purified of excess HNMe$_3$Cl, CsCl, or unwanted **[Cs$^+$][$C_4H_9CB_9H_9^{1-}$]** via dissolution into CHCl$_3$, filtration, removal of solvent under reduced pressure, and repetition of this process utilizing a minimum of cold acetone. Loss of Cs$^+$ can be tracked via ^{133}Cs NMR. Upon removal of the acetone, the white solid was stirred on cold water for 15 minutes, filtered, and washed with hexanes before being dried under vacuum at 75 °C for 2 days to remove residual water.

^{11}B{^{1}H} NMR (192 MHz, d$_6$-acetone) δ 26.97 (s, 1 B-H), -15.76(s, 4 B-H), -23.51 (s, 4 B-H). ^{11}B NMR (192 MHz, d$_6$-acetone) δ 26.99 (d, *J* = 151.8 Hz, 1 B-H), -15.76 (d, *J* = 147.7 Hz, 4 B-H), -23.51 (d, *J* = 133.9 Hz, 4 B-H). ^{1}H NMR (600 MHz, d$_6$-acetone) δ 5.19 (q, 150.9 Hz, 1 B-H), 3.21 (s, 9H), 3.09 (t, *J* = 7.4 Hz, 2H), 1.97 (m, 2H), 1.55 (h, *J* = 8.0 Hz, 2H), 1.01 (t, *J* = 7.4 Hz, 3H). ^{13}C NMR (101 MHz, d$_6$-acetone) δ 72.88, 46.21, 35.36, 34.99, 23.88, 14.51.

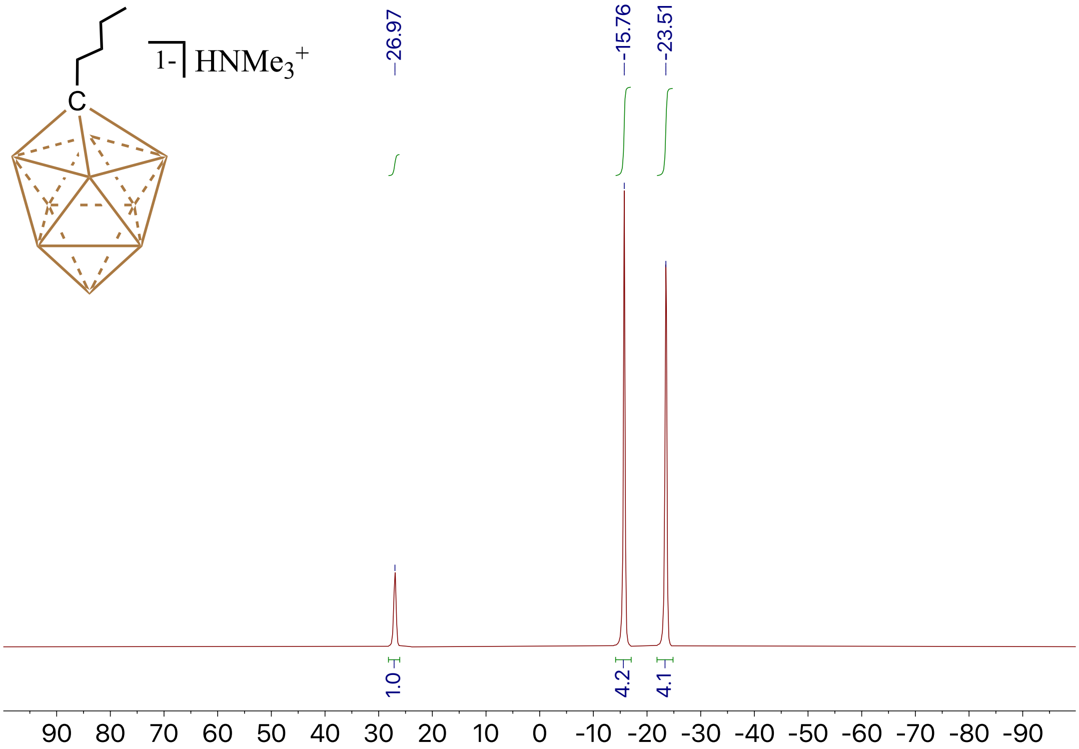

Figure S58. $^{11}B\{^{1}H\}$ NMR of **[$HNMe_3^+$][$C_4H_9CB_9H_9^{1-}$]** in wet d_6-acetone.

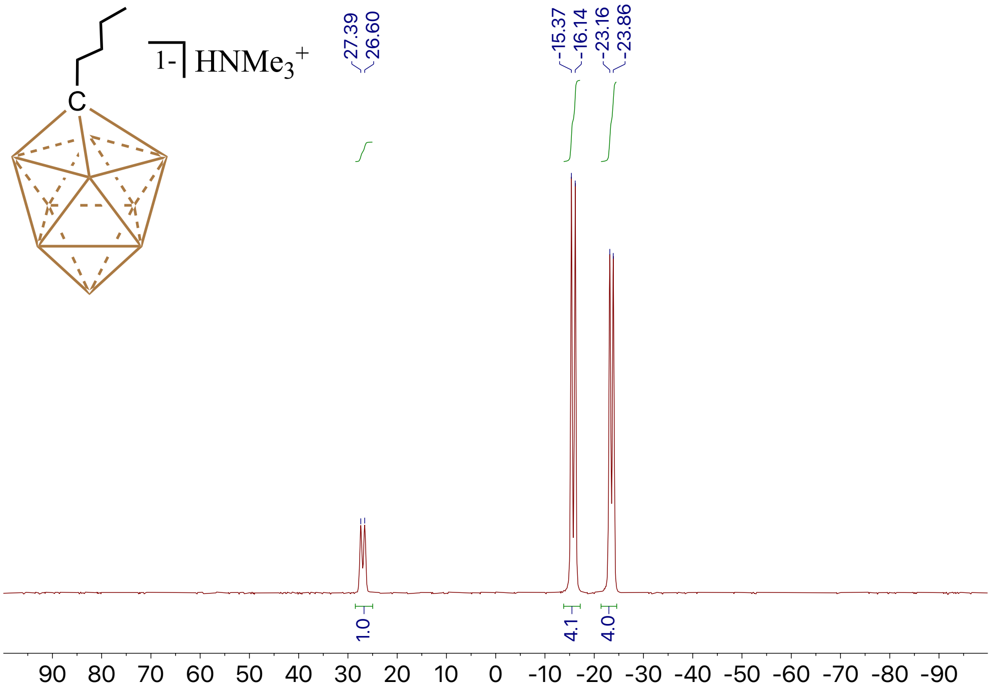

Figure S59. ^{11}B NMR of **[$HNMe_3^+$][$C_4H_9CB_9H_9^{1-}$]** in wet d_6-acetone.

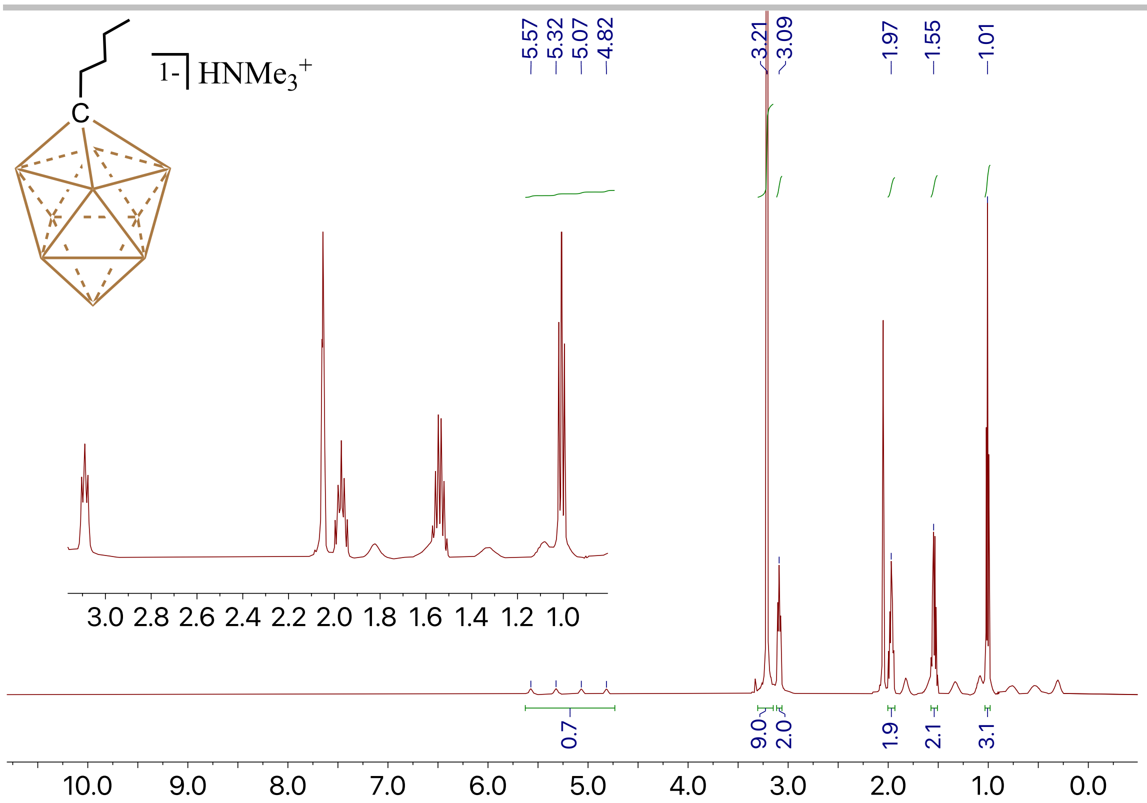

Figure S60. ^{1}H NMR of **[HNMe$_3^+$][C$_4$H$_9$CB$_9$H$_9^{1-}$]** in wet d$_6$-acetone. Note: HNMe$_3^+$ appears as singlet at 3.21 ppm.

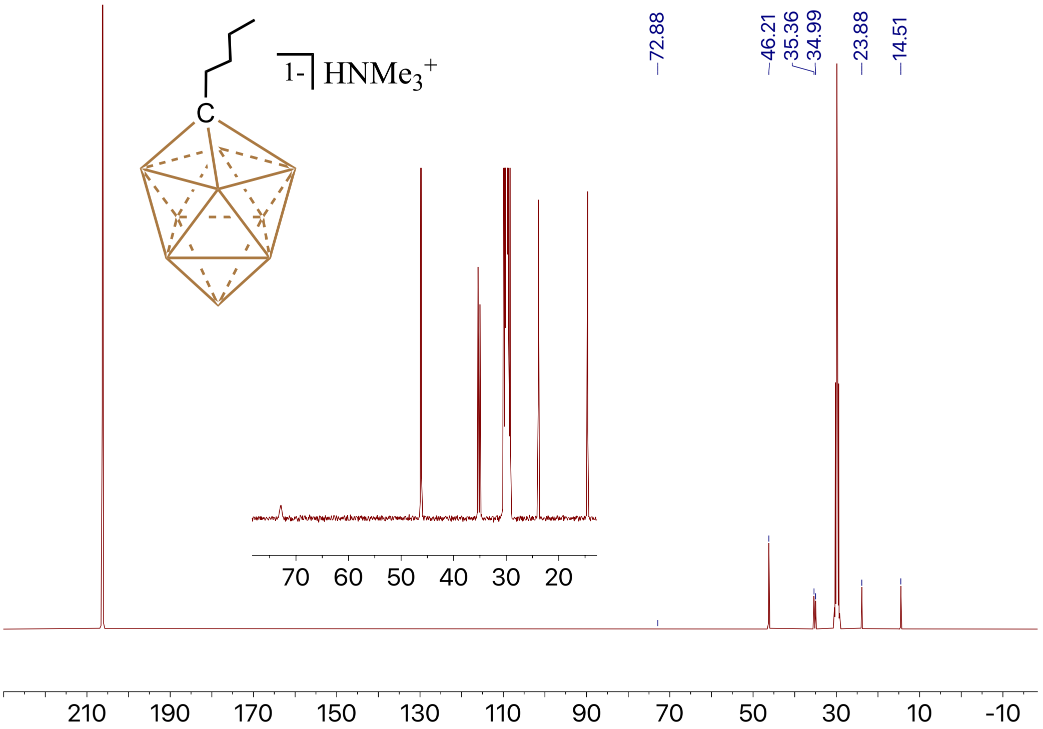

Figure S61. $^{13}C\{^{1}H\}$ NMR of **[HNMe$_3^+$][C$_4$H$_9$CB$_9$H$_9^{1-}$]** in d$_6$-acetone. Note: HNMe$_3^+$ appears as singlet at 46.21 ppm. Carboranyl C is at 72.88 ppm.

Synthesis of LiC4-THFn : [Li(THF)$_n^+$][$C_4H_9CB_9H_9^{1-}$] where n = 0, 1, 4. – Figures S62 – S84

[$HNMe_3^+$][$C_4H_9CB_9H_9^{1-}$] was dried *in-vacuo* at 80°C for 24 hours prior to use. Working in a glovebox, 5.0 g of **[$HNMe_3^+$][$C_4H_9CB_9H_9^{1-}$]** was dissolved in a 50 mL of THF. LiH (1.5 g, excess) was added slowly until the effervescence of NMe_3 and H_2 gaseous biproducts seized. The solution was left to stir for 3 hours to ensure reaction completion. The slurry was filtered twice through a Whatman GF/A grade glass microfiber filter to remove excess LiH. The resulting clear solution was concentrated in-vacuo to yield **LiC_4-THF_4** as a fluffy white powder (9.84 g, 98.5% yield, m.p. = 37.5 – 38.9 °C). The powder was subsequently heated at 130 °C in-vacuo for 16 hours while stirring to yield **LiC_4-THF** (m.p. = -57.1 °C via DSC). The presence of 1 molar equ. of THF:Li^+ was confirmed by ^1H NMR with peak integration referenced to the methylene (CH_2) at δ = 3.2 ppm. To remove all THF, this salt was heated at 190 °C in-vacuo for 3 days while stirring to yield **LiC_4** (m.p. = 106.0 – 107.6 °C). Loss of THF was observed by ^1H NMR.

Alternatively, working in a glovebox, 5.0 g of **[$HNMe_3^+$][$C_4H_9CB_9H_9^{1-}$]**$^-$ was dissolved in a 50 mL of THF. LiH was added carefully until the effervescence of NMe_3 and H_2 gaseous biproducts siezed upon which an excess of LiH was added and left to stir for 3 hours to ensure reaction completion. The solution was filtered twice through a Whatman GF/A grade glass microfiber filter to remove excess LiH. The resulting clear solution was concentrated in-vacuo followed by drying at 190°C for 48 hours to yield solvent free **LiC_4** as a white solid. Working in a glovebox, 0.234 g (1.27 mmol) **LiC_4** was measured using a balance and stirred in 5 mL of pentane. 1.27 mmol of THF was measured via micropipette and added to the stirring slurry. The reaction was allowed to stir 2 hours to ensure homogeneity. The pentane was removed in vacuo at 25 °C yielding the desired product which was confirmed by ^1H NMR to contain the desired product with peak integration referenced to the methylene (CH_2) at δ = 3.2 ppm.

LiC_4: $^{11}B\{^1H\}$ NMR (96 MHz, $CDCl_3$, 25°C): ∂ = 14.82, -15.96, -23.56 ppm. ^{11}B NMR (96 MHz, $CDCl_3$, 25°C): ∂ = 14.81, -15.98, -23.53 ppm. ^1H NMR (600 MHz, $CDCl_3$, 25°C): ∂ = 3.23 (t, 2H), 2.01 (m, 2H) ppm, 1.6 (m, 2H), 1.06 (t, 3H) ppm. ^7Li NMR (233 MHz, $CDCl_3$, 25°C): ∂ = 0.58 ppm.

LiC_4-THF: $^{11}B\{^1H\}$ NMR (96 MHz, $CDCl_3$, 25°C): ∂ = 15.4, -16.11, -23.67 ppm. ^{11}B NMR (96 MHz, $CDCl_3$, 25°C): ∂ = 16.18, -15.48, -23.11 ppm. ^1H NMR (600 MHz, $CDCl_3$, 25°C): ∂ = 3.85 (m, 4H), 3.21 (t, 2H), 2.04 -1.94 (m, 6H) ppm, 1.6 (m, 2H), 1.06 (t, 3H) ppm. $^1H\{^{11}B\}$ NMR (600 MHz, $CDCl_3$, 25°C): ∂ = 4.92 (bs, 1H, B-H), 3.86 (m, 4H), 3.22 (t, 2H), 2.01 (m, 2H), 1.94 (m, 4H), 1.75 (bs, 4H, B-H), 1.62 (m, 2H), 1.07 (t, 3H), 0.79 (bs, 4H, B-H) ppm. ^{13}C NMR (151 MHz, $CDCl_3$, 25°C): ∂ = 83.05, 69.03, 34.44, 33.97, 25.41, 23.12, 14.23 ppm. ^7Li NMR (233 MHz, $CDCl_3$, 25°C): ∂ = 0.34 ppm.

LiC_4-THF_4: $^{11}B\{^1H\}$ NMR (96 MHz, $CDCl_3$, 25°C): ∂ = 19.41, -17.49, -25.32 ppm. ^{11}B NMR (96 MHz, $CDCl_3$, 25°C): ∂ = 23.65, -16.87, -24.74 ppm. ^1H NMR (600 MHz, $CDCl_3$, 25°C): ∂ = 3.86 (m, 16H), 3.20 (t, 2H), 2.04 -1.94 (m, 18H) ppm, 1.59 (m, 2H), 1.06 (t, 3H) ppm. $^1H\{^{11}B\}$ NMR (600 MHz, $CDCl_3$, 25°C): ∂ = 5.05 (bs, 1H, B-H), 3.77 (m, 16H), 3.12 (t, 2H), 2.01-1.94 (m, 18H), 1.51 (m, 6H, B-H), 1.31 (m, 3H), 1.00 (t, 3H), 0.63 (bs, 4H, B-H) ppm.

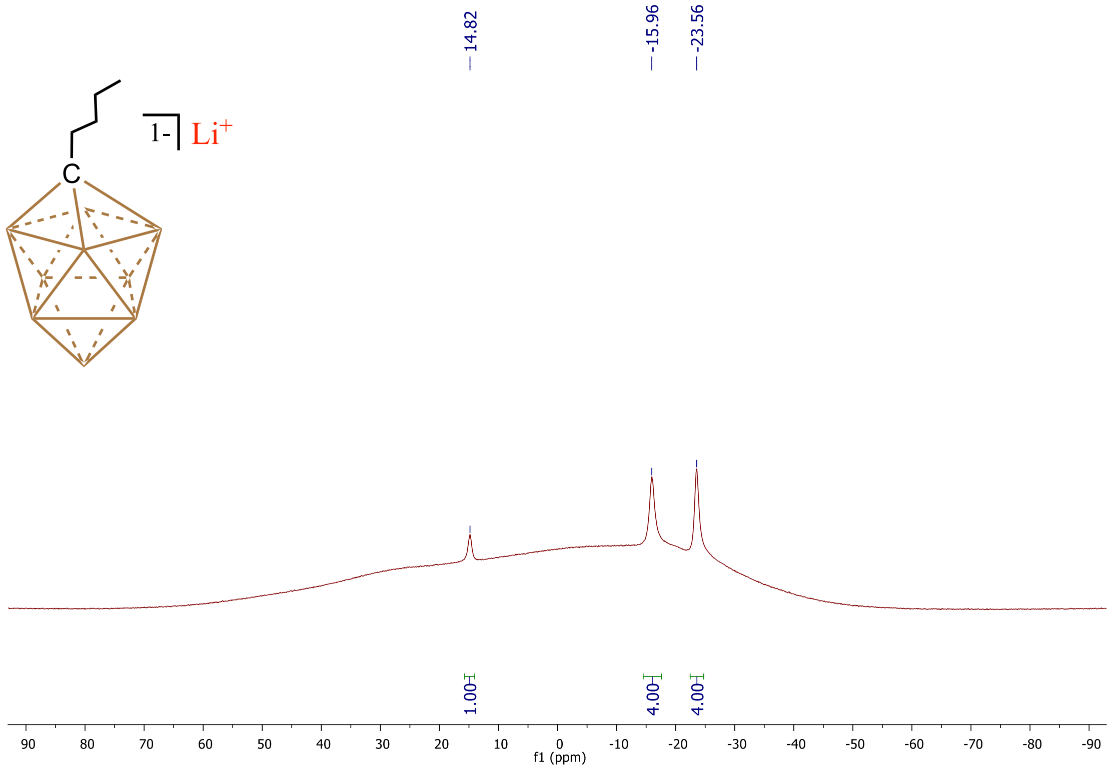

Figure S62. $^{11}B\{^1H\}$ NMR spectra of **LiC$_4$** ($[Li^+][C_4H_9CB_9H_9^{1-}]$) in anhydrous $CDCl_3$

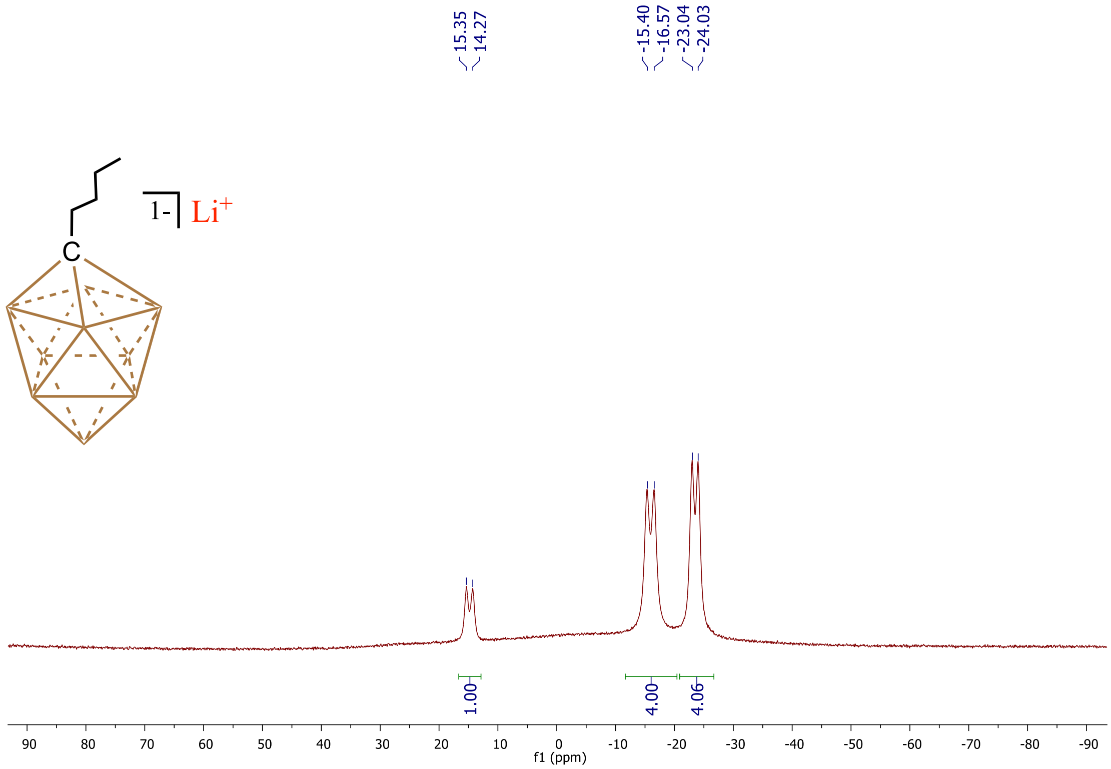

Figure S63. ^{11}B NMR spectra of **LiC$_4$** ($[Li^+][C_4H_9CB_9H_9^{1-}]$) in anhydrous $CDCl_3$

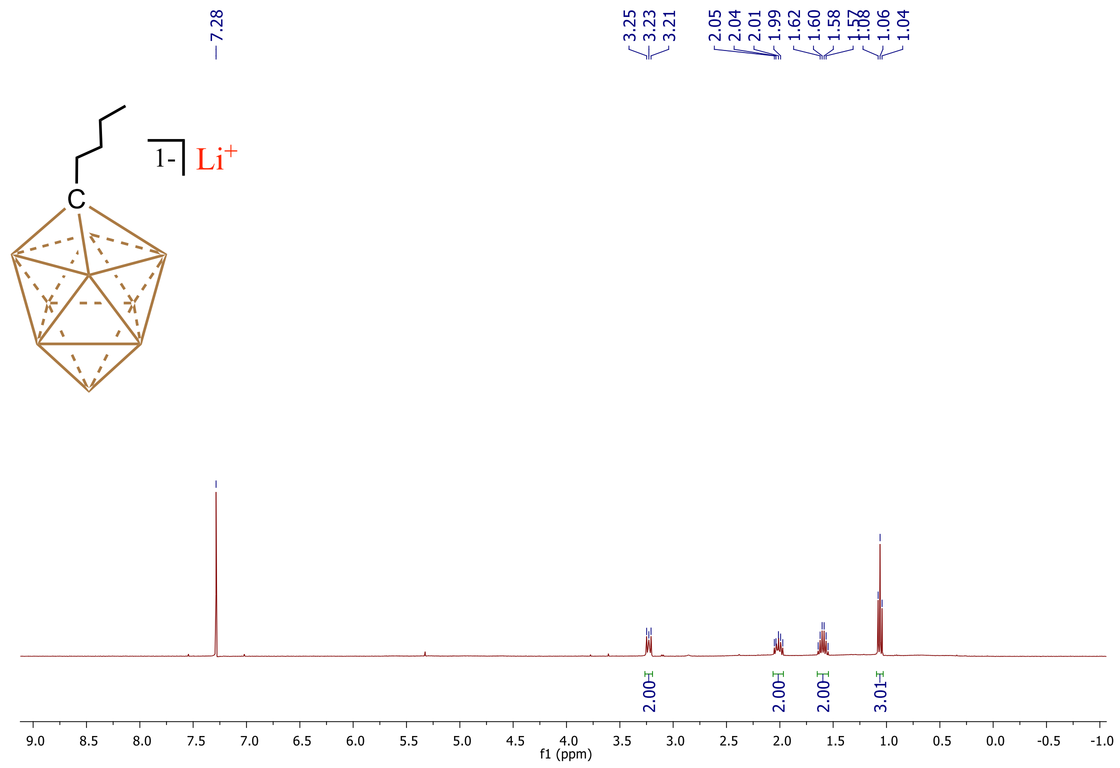

Figure S64. ^{1}H NMR spectra of **LiC4** ($[Li^{+}][C_4H_9CB_9H_9^{1-}]$) in anhydrous $CDCl_3$

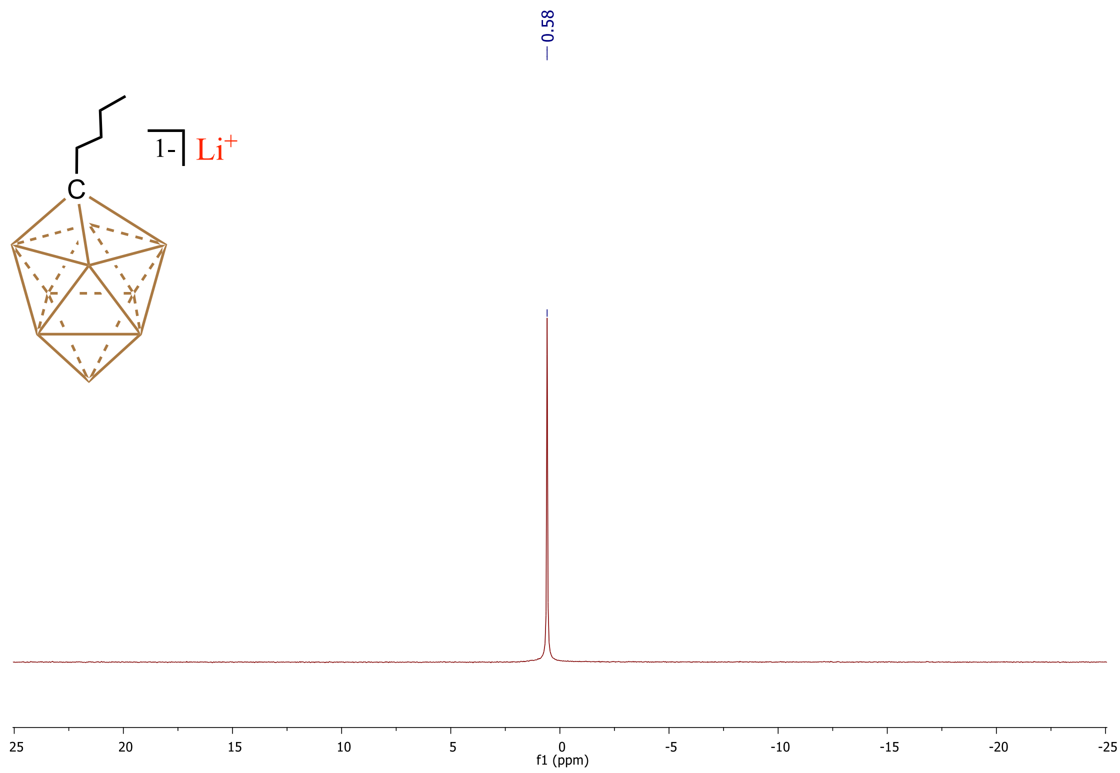

Figure S65. ^{7}Li NMR spectra of **LiC4** ($[Li^{+}][C_4H_9CB_9H_9^{1-}]$) in anhydrous $CDCl_3$

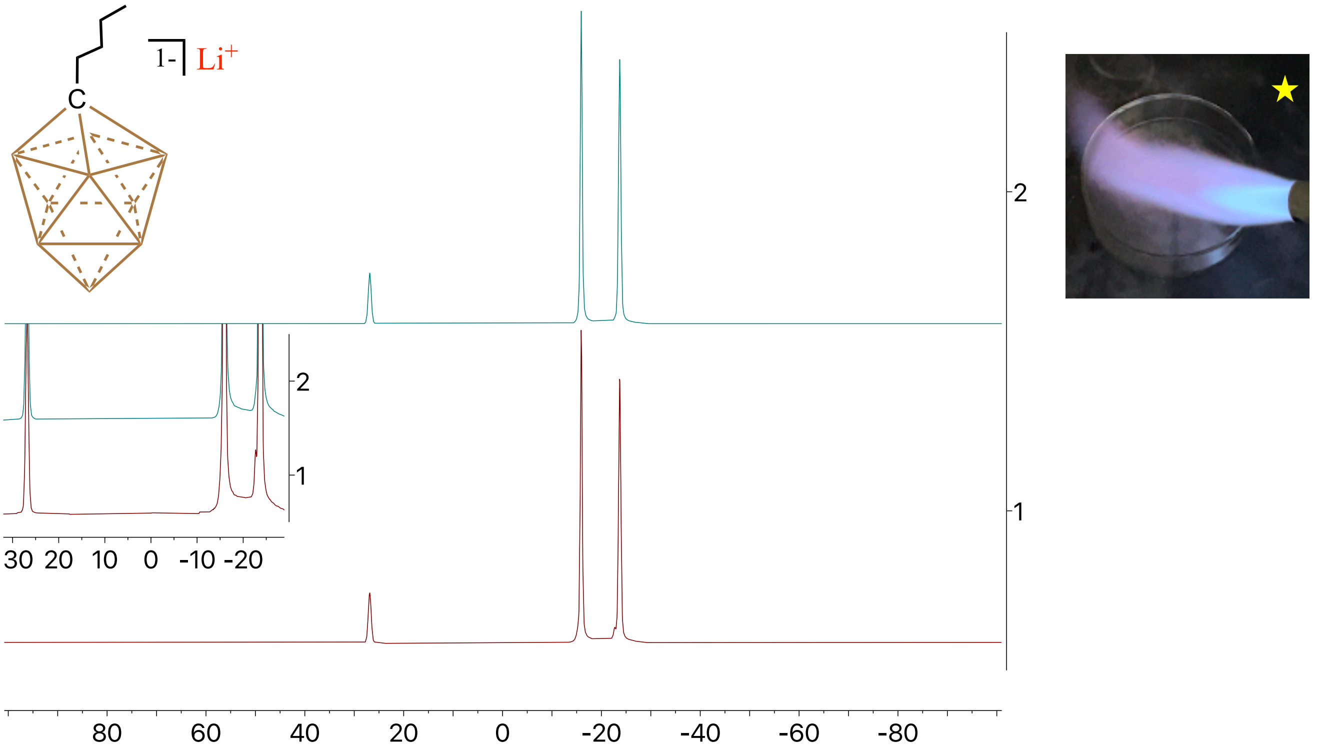

Figure S66. $^{11}B\{^{1}H\}$ NMR of **LiC4** ($[Li^+][C_4H_9CB_9H_9^{1-}]$) before (above) and after (below) burning 3s in d6-acetone.

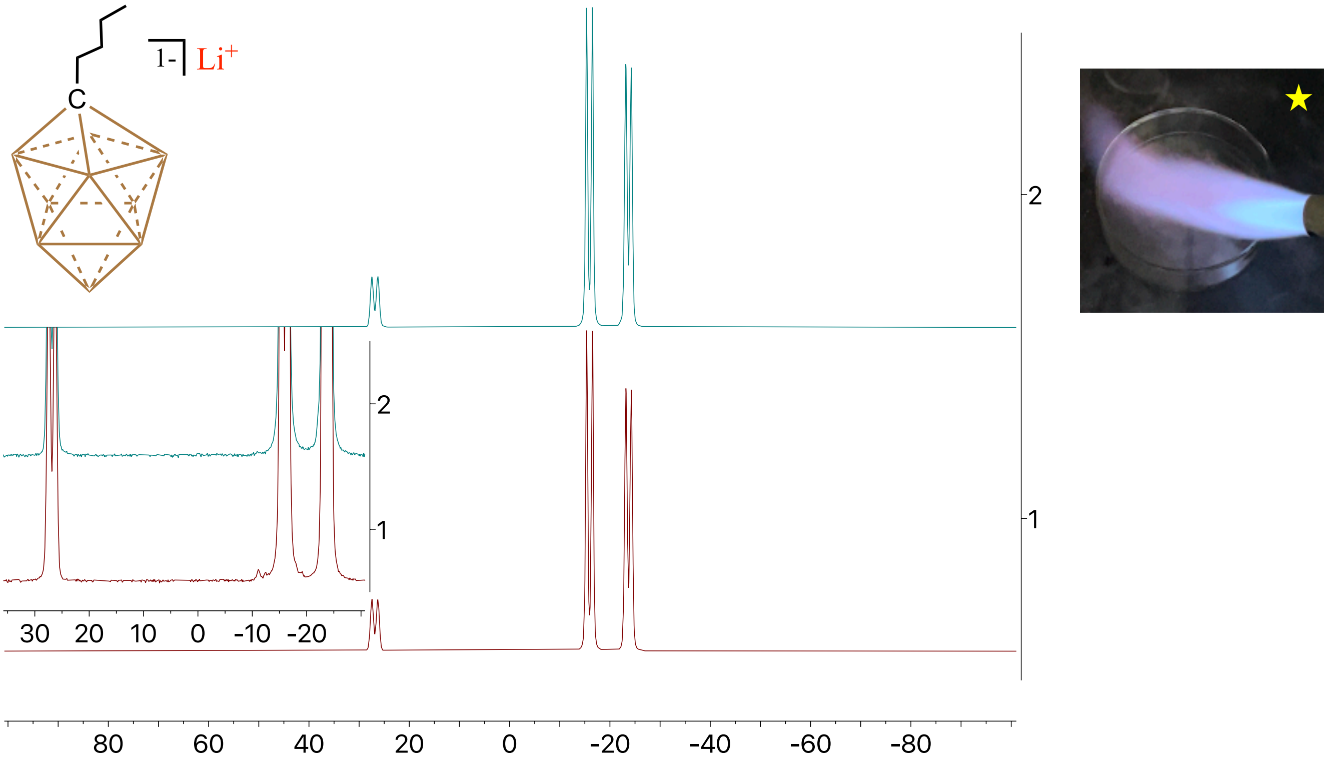

Figure S67. ^{11}B NMR of **LiC4** ($[Li^+][C_4H_9CB_9H_9^{1-}]$) before (above) and after (below) burning 3s in d6-acetone.

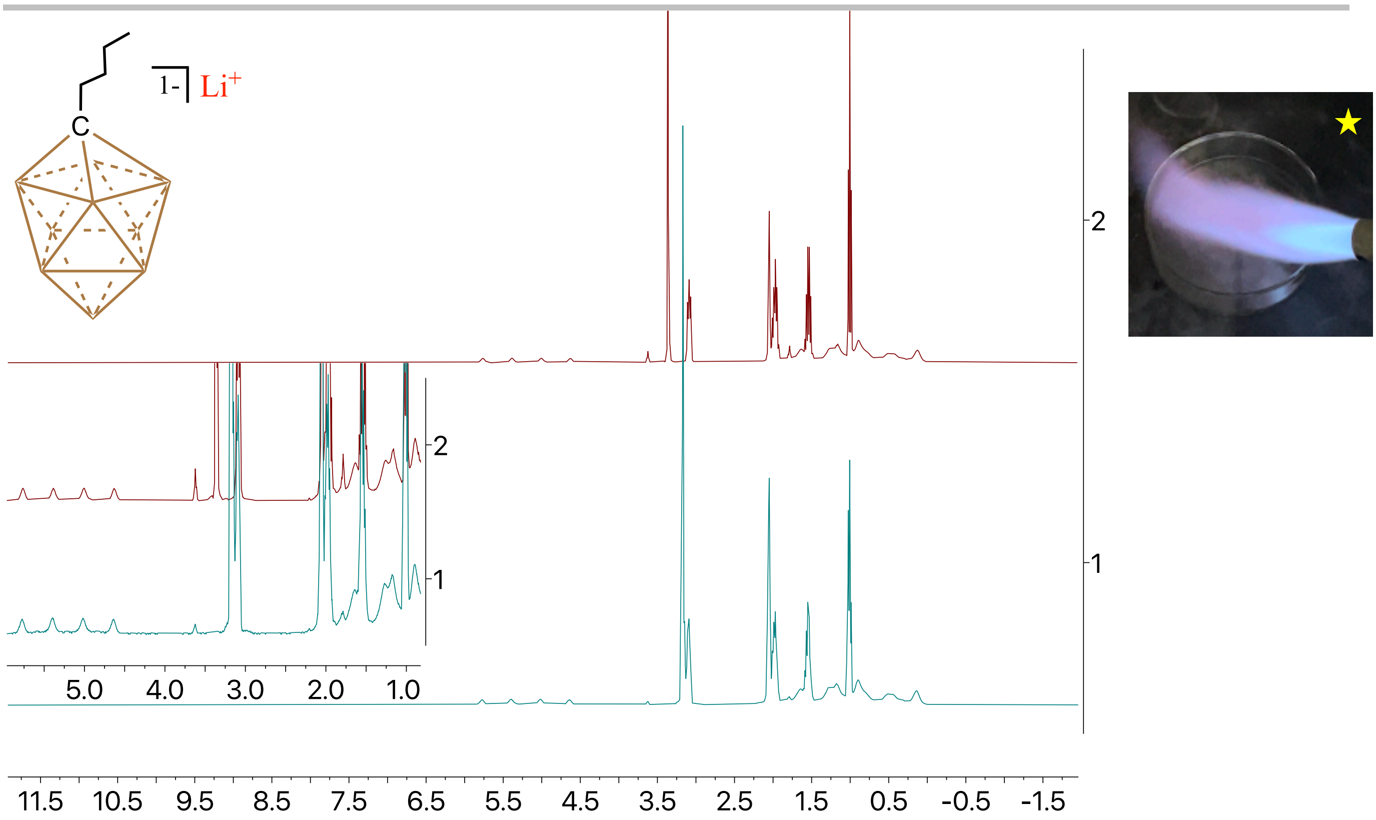

Figure S68. ^{1}H NMR of **LiC₄** ($[Li^{+}][C_4H_9CB_9H_9{}^{1-}]$) before (above) and after (below) burning 3s in d_6-acetone. Note: water peaks arise at 3.3 (above) and 3.2 ppm (below).

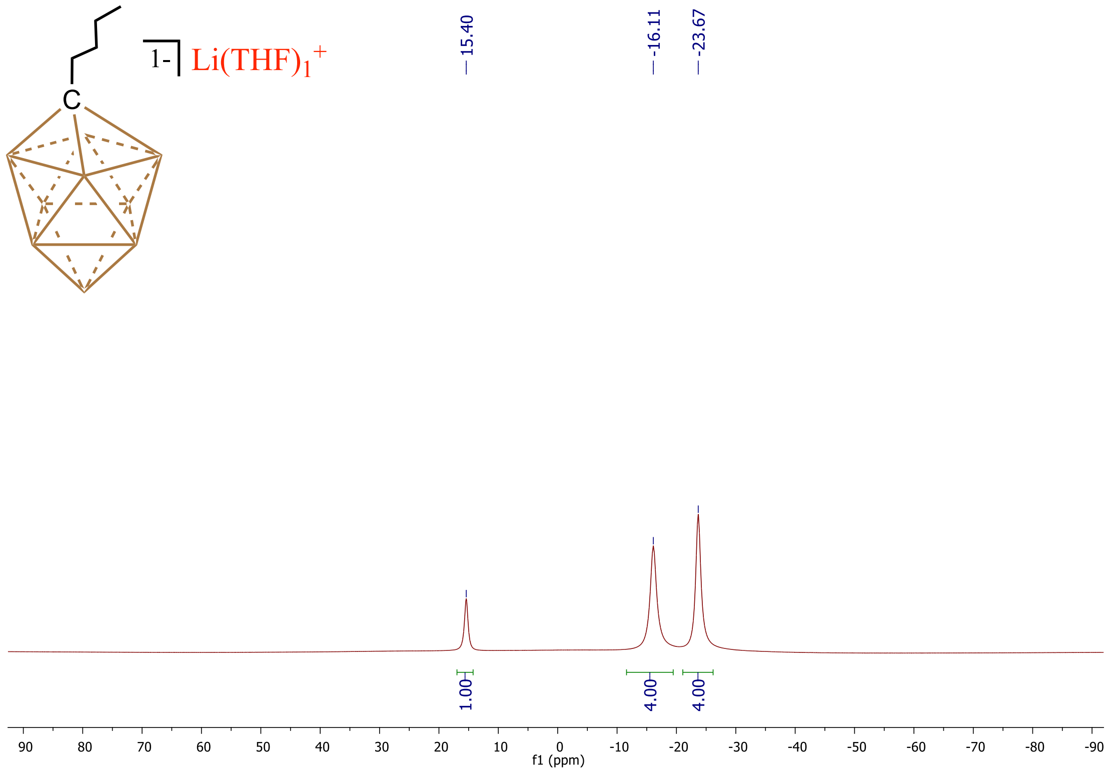

Figure S69. $^{11}B\{^{1}H\}$ NMR spectra of **LiC₄-THF** ($[Li(THF)_1{}^{+}][C_4H_9CB_9H_9{}^{1-}]$) in anhydrous $CDCl_3$

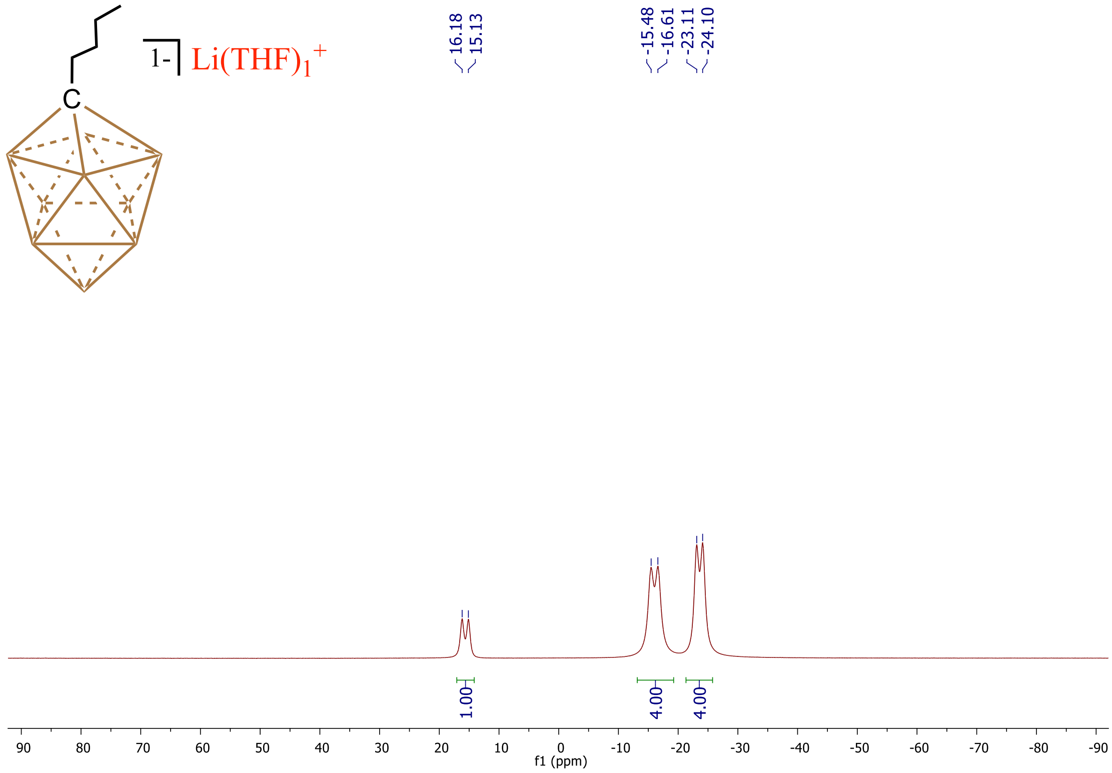

Figure S70. ^{11}B NMR spectra of **LiC$_4$-THF** ($[Li(THF)_1^{+}][C_4H_9CB_9H_9^{1-}]$) in anhydrous $CDCl_3$

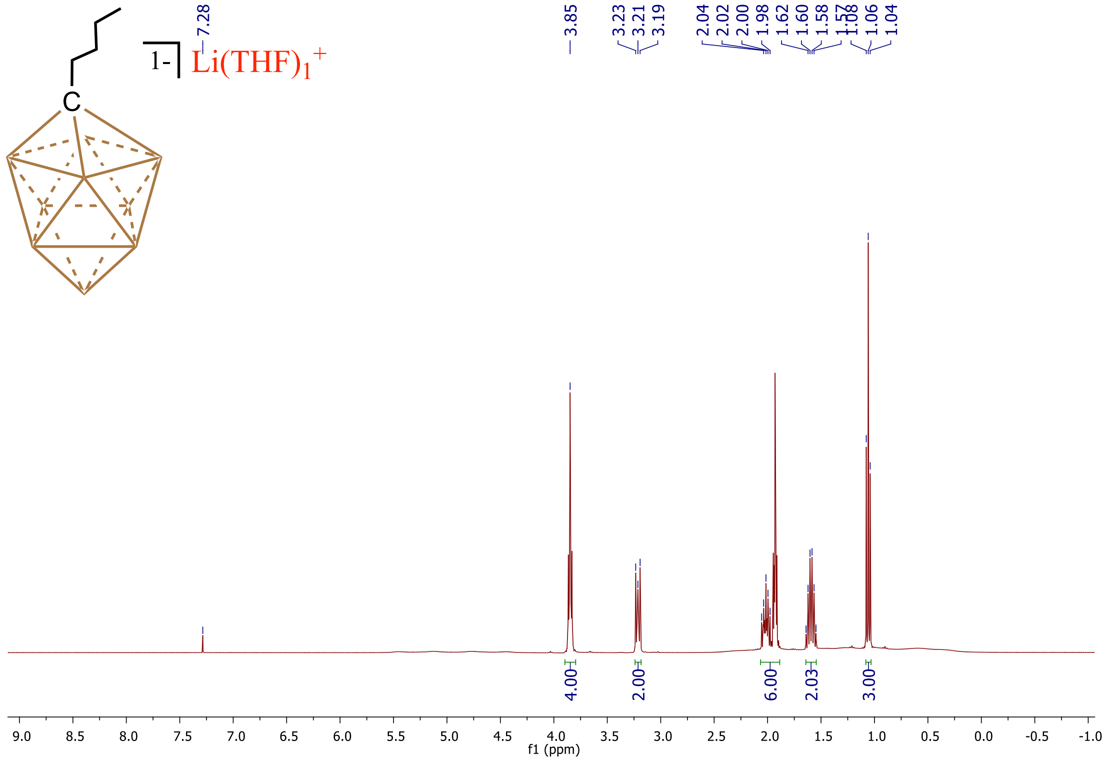

Figure S71. ^{1}H NMR spectra of **LiC$_4$-THF** ($[Li(THF)_1^{+}][C_4H_9CB_9H_9^{1-}]$) in anhydrous $CDCl_3$

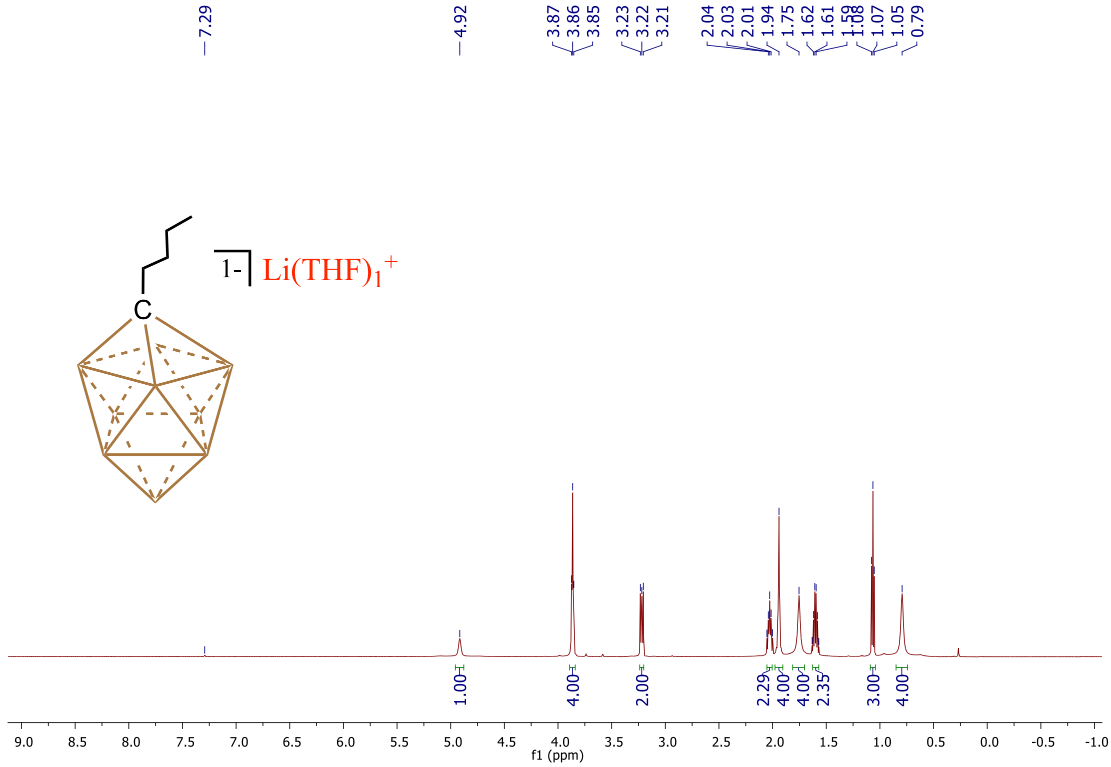

Figure S72. $^{1}H\{^{11}B\}$ NMR spectra of **LiC4-THF** ($[Li(THF)_1^{+}][C_4H_9CB_9H_9^{1-}]$) in anhydrous $CDCl_3$

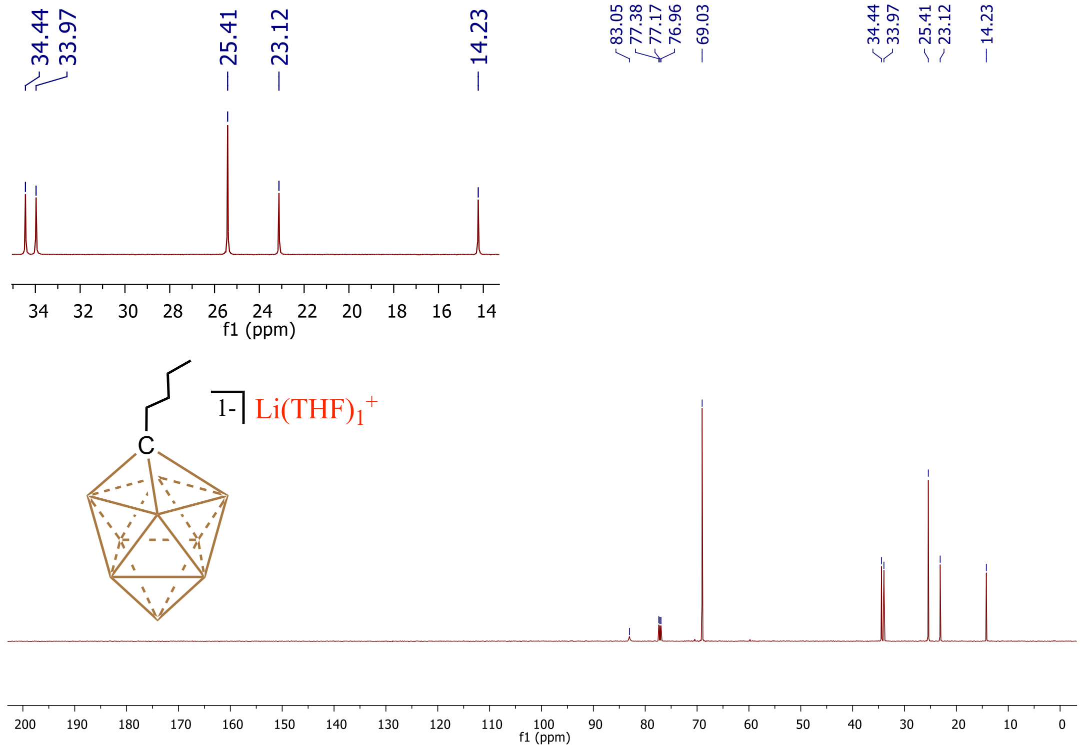

Figure S73. ^{13}C NMR spectra of **LiC4-THF** ($[Li(THF)_1^{+}][C_4H_9CB_9H_9^{1-}]$) in anhydrous $CDCl_3$

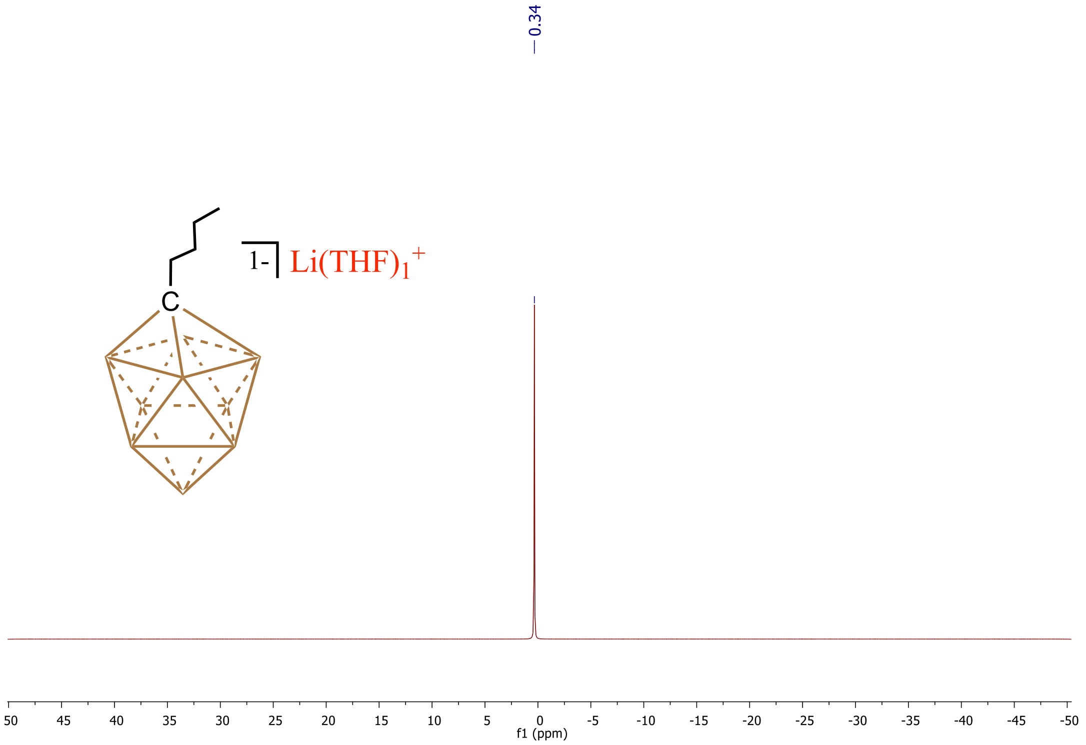

Figure S74. ^{7}Li NMR spectra of **LiC$_4$-THF** ([Li(THF)$_1$$^+$][C$_4H_9CB_9H_9$$^{1-}$]) in anhydrous CDCl$_3$

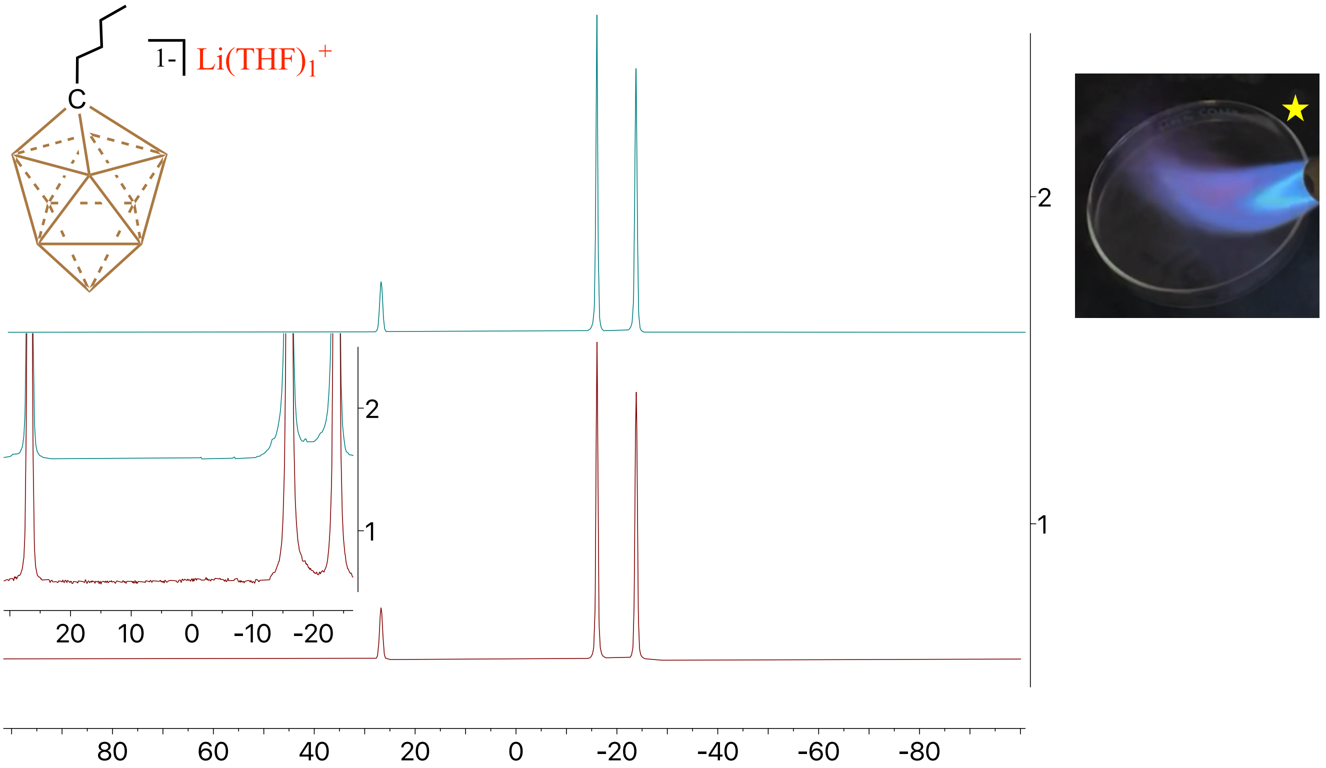

Figure S75. ^{11}B{^{1}H} NMR of **LiC$_4$-THF** ([Li(THF)$_1$$^+$][C$_4H_9CB_9H_9$$^{1-}$]) before (above) and after (below) burning 10s in d$_6$-acetone.

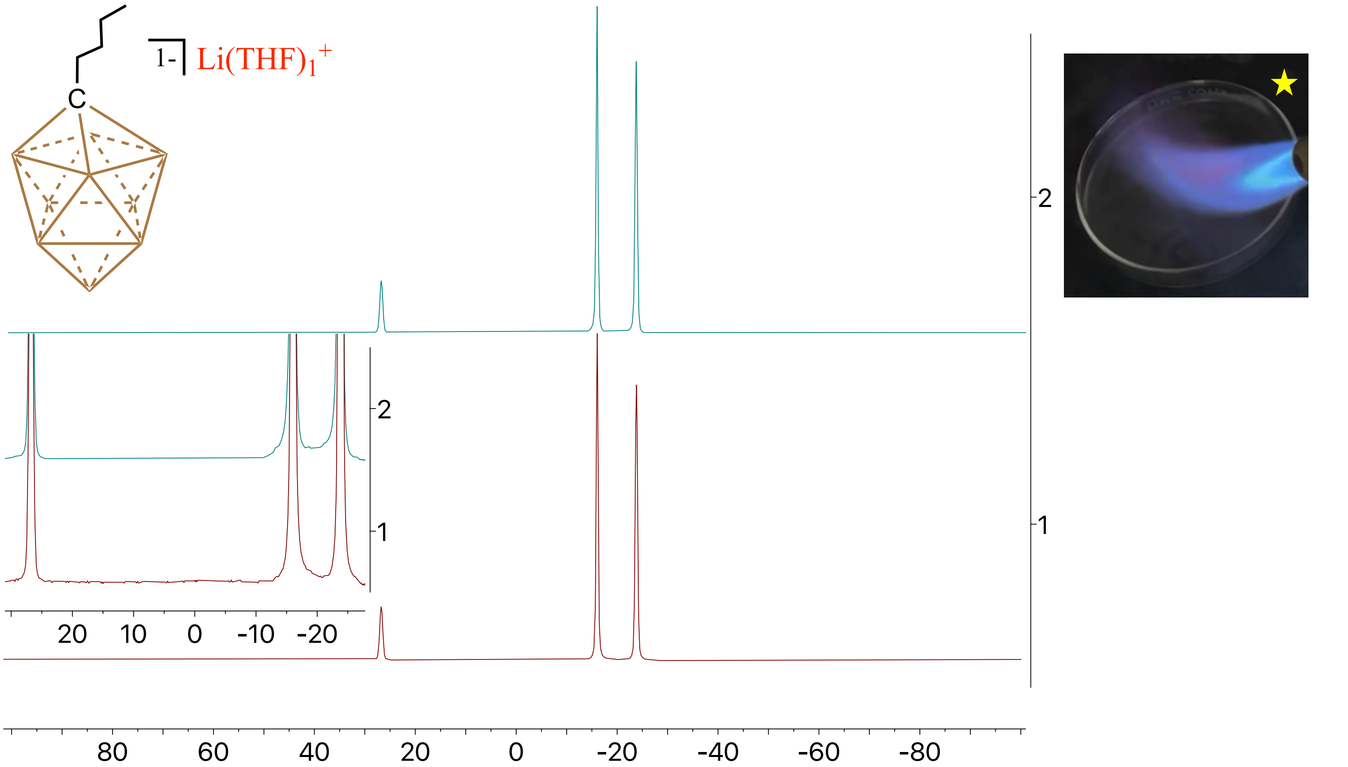

Figure S76. ^{11}B NMR of **LiC$_4$-THF** ([Li(THF)$_1$$^+$][C$_4H_9CB_9H_9$$^{1-}$]) before (above) and after (below) burning 10s, in d$_6$-acetone.

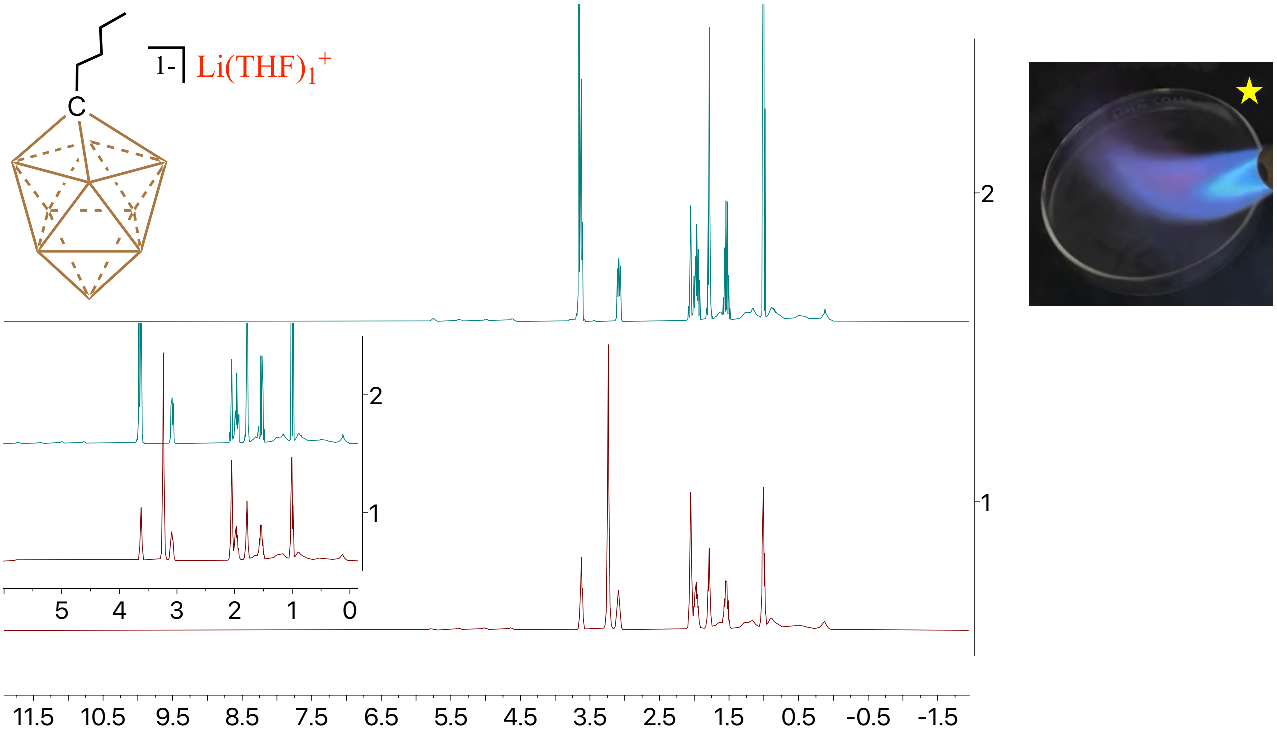

Figure S77. ^{1}H NMR of **LiC$_4$-THF** ([Li(THF)$_1$$^+$][C$_4H_9CB_9H_9$$^{1-}$]) before (above) and after (below) burning 10s in d$_6$-acetone. Note: water peaks arise at 3.7 (above) and 3.2 ppm (below).

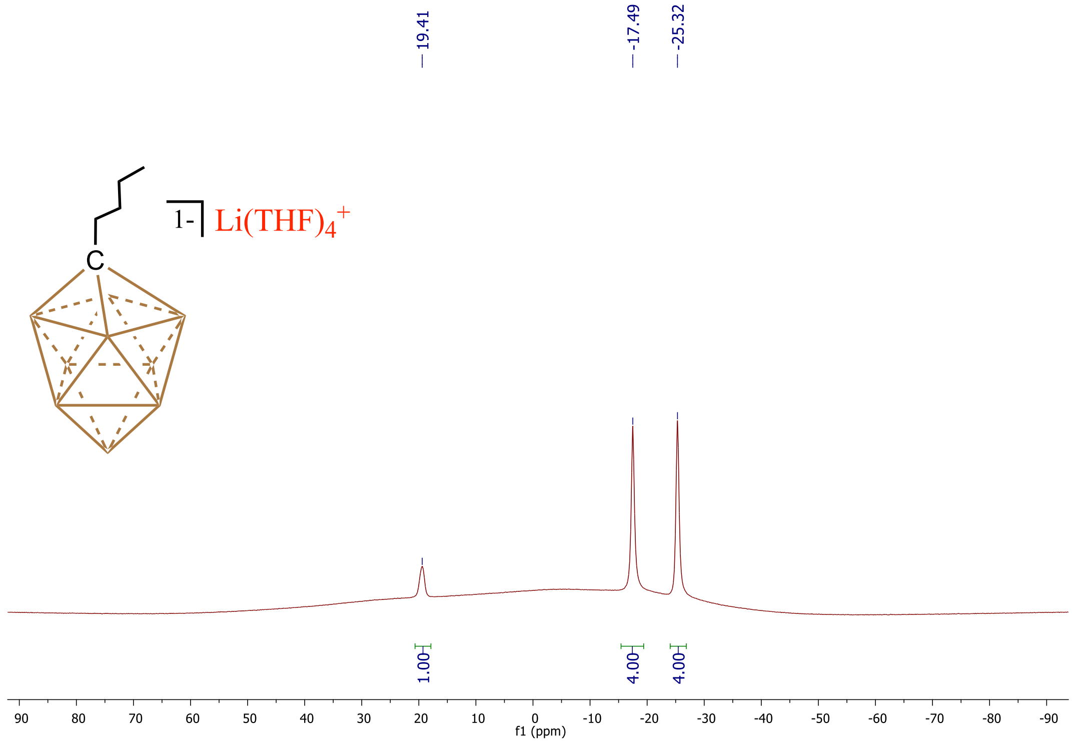

Figure S78. $^{11}B\{^1H\}$ NMR spectra of **LiC$_4$-THF$_4$** ($[Li(THF)_4^+][C_4H_9CB_9H_9^{1-}]$) in anhydrous $CDCl_3$

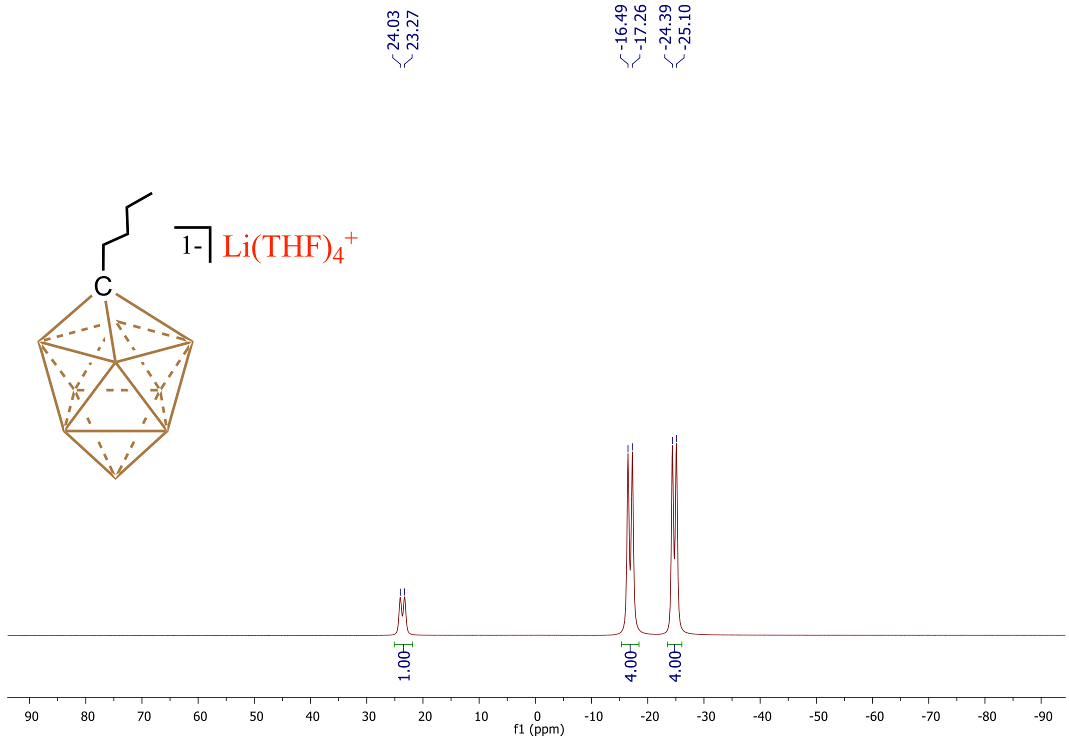

Figure S79. ^{11}B NMR spectra of **LiC$_4$-THF$_4$** ($[Li(THF)_4^+][C_4H_9CB_9H_9^{1-}]$) in anhydrous $CDCl_3$

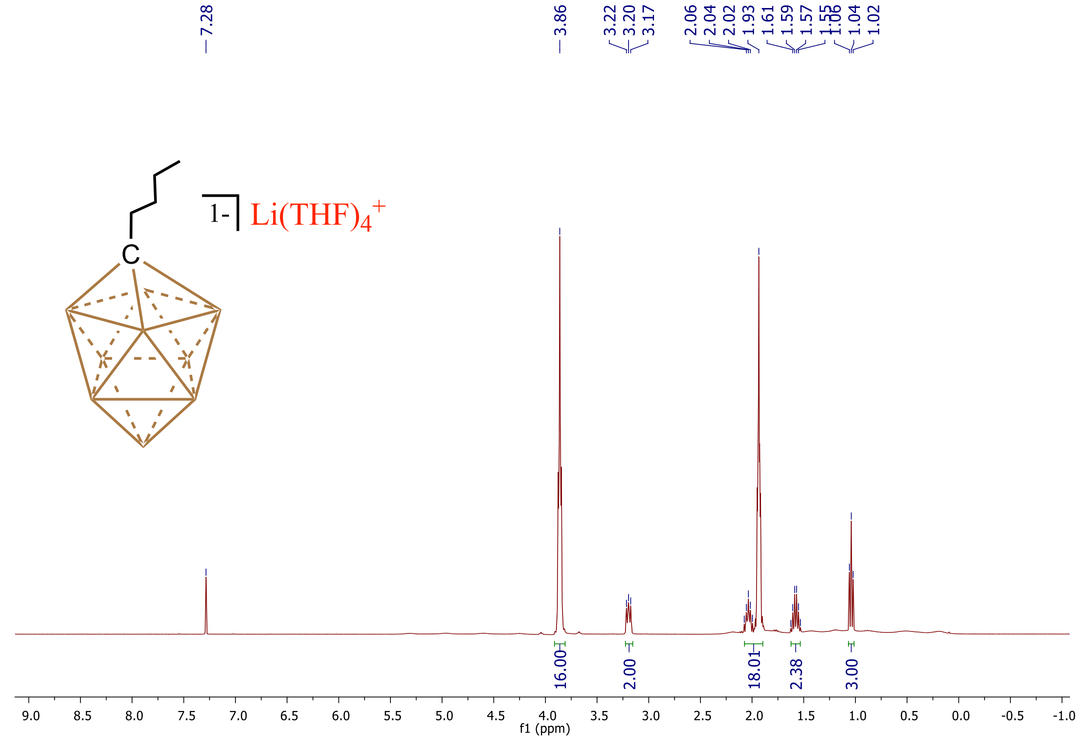

Figure S80. ^{1}H NMR spectra of **LiC₄-THF₄** ([Li(THF)$_4^+$][$C_4H_9CB_9H_9^{1-}$]) in anhydrous $CDCl_3$

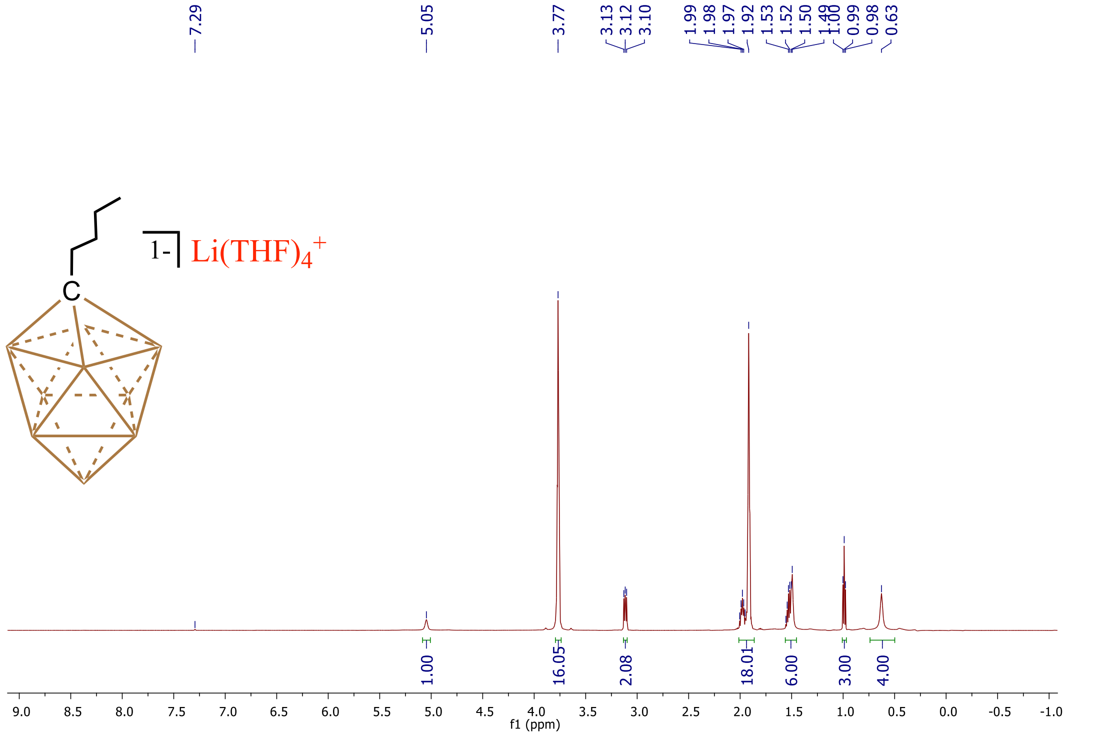

Figure S81. ^{1}H{^{11}B} NMR spectra of **LiC₄-THF₄** ([Li(THF)$_4^+$][$C_4H_9CB_9H_9^{1-}$]) in anhydrous $CDCl_3$

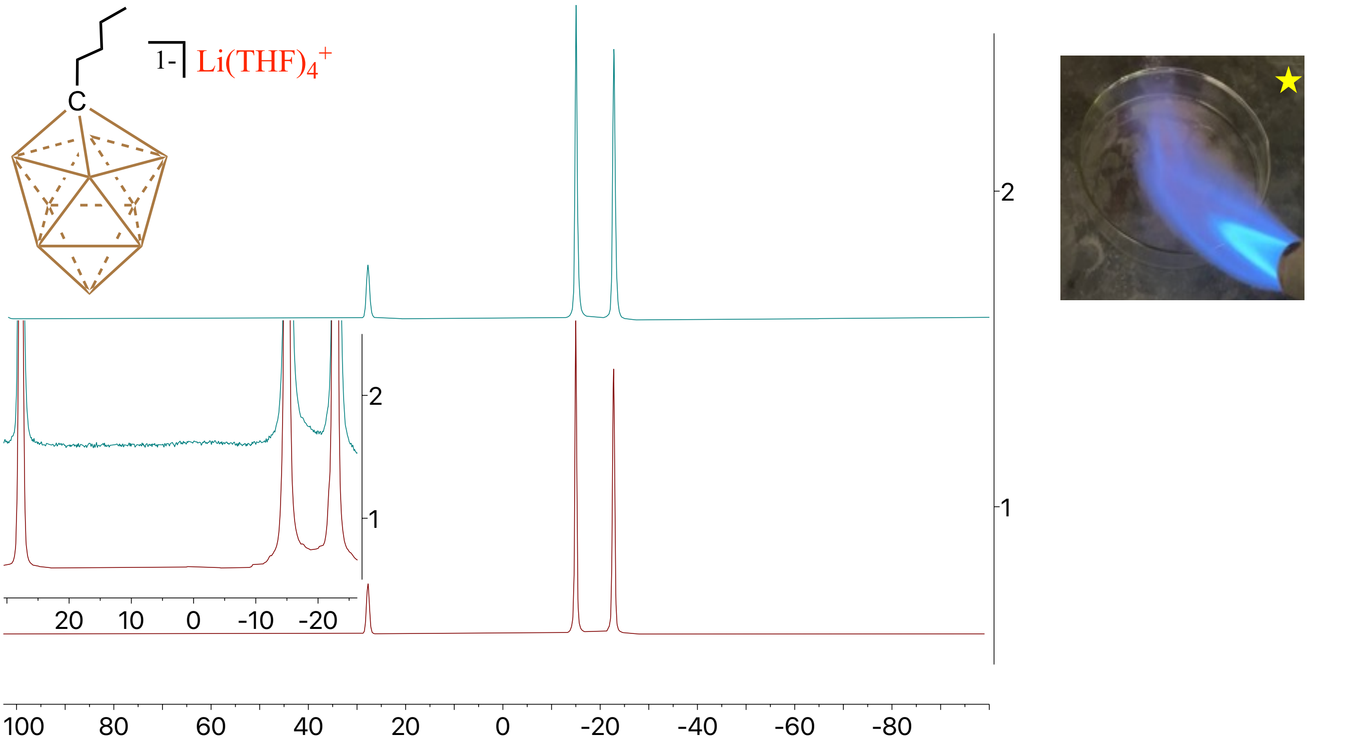

Figure S82. $^{11}B\{^1H\}$ NMR of **LiC4-THF4** ($[Li(THF)_4^+][C_4H_9CB_9H_9^{1-}]$) before (above) and after (below) burning 3s in d6-acetone.

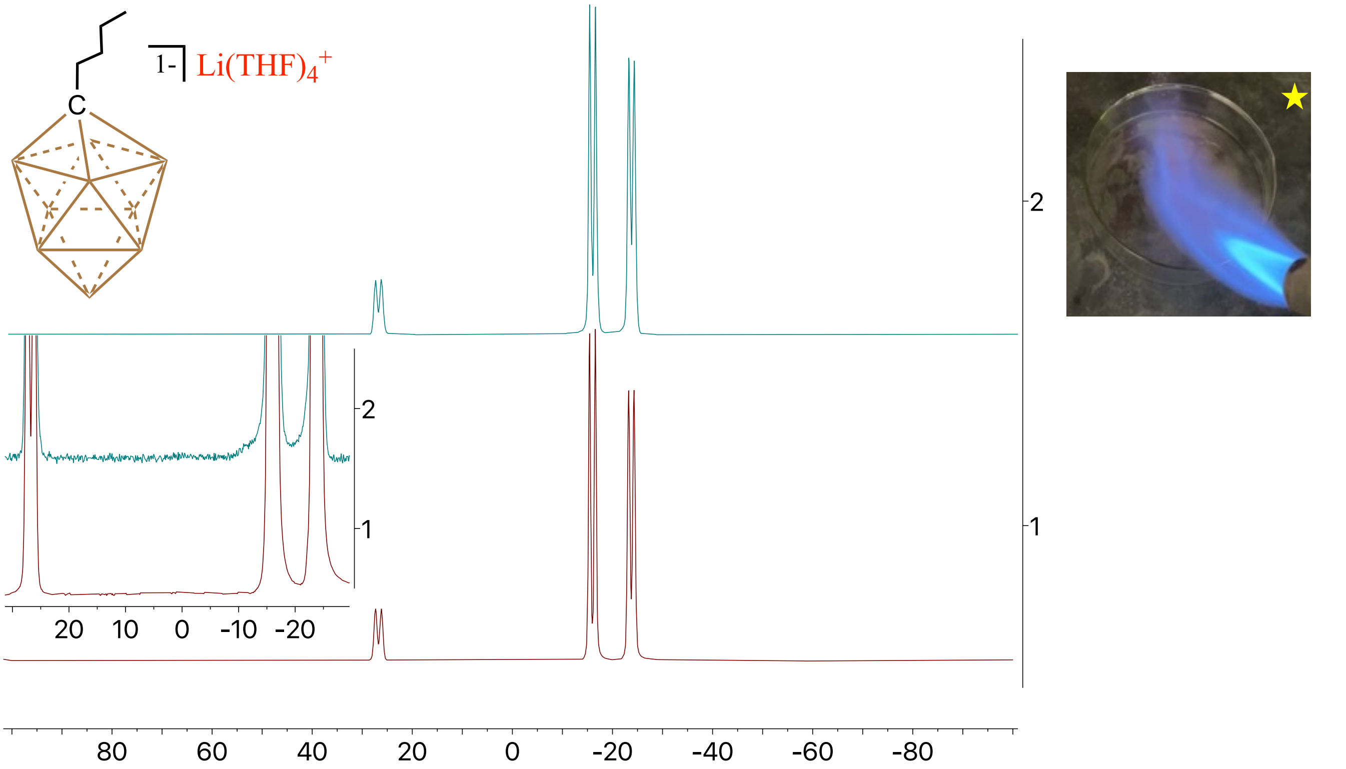

Figure S83. ^{11}B NMR of **LiC4-THF4** ($[Li(THF)_4^+][C_4H_9CB_9H_9^{1-}]$) before (above) and after (below) burning 3s in d6-acetone.

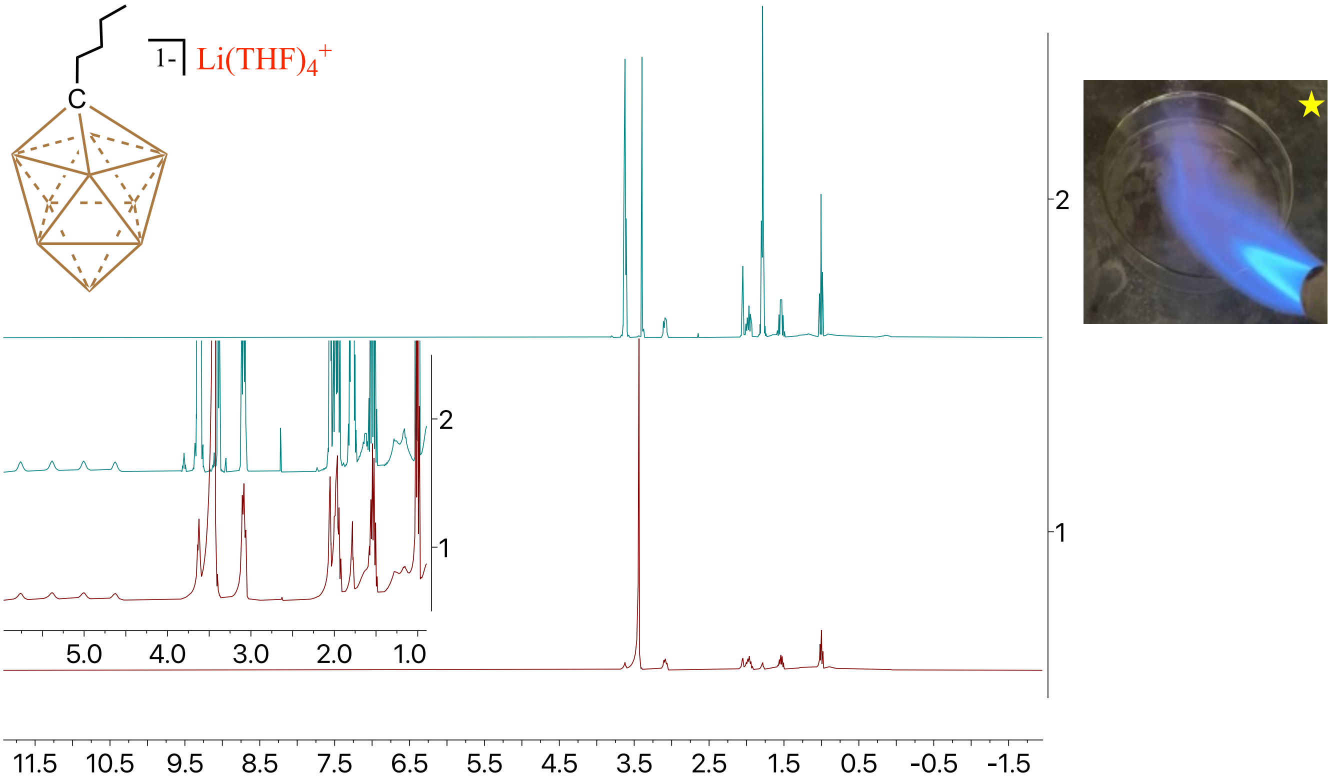

Figure S84. ^{1}H NMR of **LiC$_4$-THF$_4$** ($[Li(THF)_4^+][C_4H_9CB_9H_9^{1-}]$) before (above) and after (below) burning 3s in d_6-acetone. Note: water peaks arise at 3.4 ppm.

Synthesis of NaC$_4$-THF$_n$: $[Na(THF)_n^+][C_4H_9CB_9H_9^{1-}]$ where n = 0, 2 – Figures S85 – S97

$[Na(THF)_n^+]$ $[C_4H_9CB_9H_9^{1-}]$ was synthesized in the same manner as **$[Na(THF)_n^+][HCB_9H_9^{1-}]$**.

n = 0. (m.p. = 116.1 – 119.5 °C) : ^{11}B{^{1}H} NMR (128 MHz, d_6-acetone) δ 26.81 (s, 1 B-H), -16.01 (s, 4 B-H), -23.79 (s, 4 B-H). ^{11}B NMR (128 MHz, d_6-acetone) δ 26.85 (d, J = 150.2 Hz), -15.97 (d, J = 147.6 Hz), -23.75 (d, J = 136.9 Hz). ^{1}H NMR (400 MHz, d_6-acetone) δ 5.21 (q, 150.8 Hz, 1 B-H), 3.14 – 3.05 (m, 2H), 2.05 – 1.91 (m, 2H), 1.54 (h, J = 7.4 Hz, 2H), 1.01 (t, J = 7.4 Hz, 3H). ^{23}Na NMR (106 MHz, d_6-acetone) δ -5.96.

n = 2. (m.p. = 64.8 – 67.1 °C) : ^{11}B{^{1}H} NMR (128 MHz, d_6-acetone) δ 25.93 (s, 1 B-H), -16.92 (s, 4 B-H), -24.70 (s, 4 B-H). ^{11}B NMR (128 MHz, d_6-acetone) δ 25.91 (d, J = 151.5 Hz, 1 B-H), -16.92 (d, J = 149.7 Hz, 4 B-H), -24.69 (d, J = 136.8 Hz, 4 B-H). ^{1}H NMR (400 MHz, d_6-acetone) δ 5.21 (q, J = 151.7 Hz, 1 B-H), 3.62 (m, 8H), 3.09 (t, 2H), 1.97 (m, 2H), 1.79 (s, 8H), 1.55 (h, J = 8.0 Hz, 2H), 1.01 (t, J = 7.4 Hz, 3H). ^{23}Na NMR (106 MHz, d_6-acetone) δ -6.01 (s).

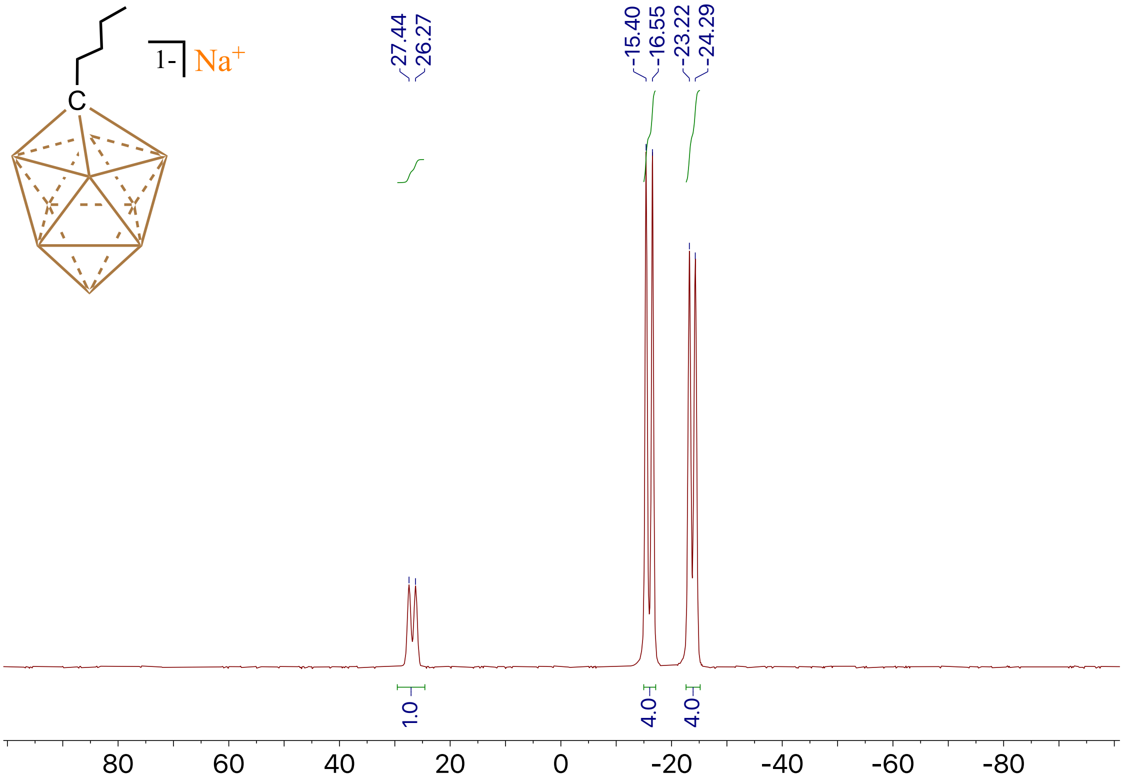

Figure S85. ^{11}B NMR of **NaC4** ($[Na^+][C_4H_9CB_9H_9^{1-}]$) in d_6-acetone.

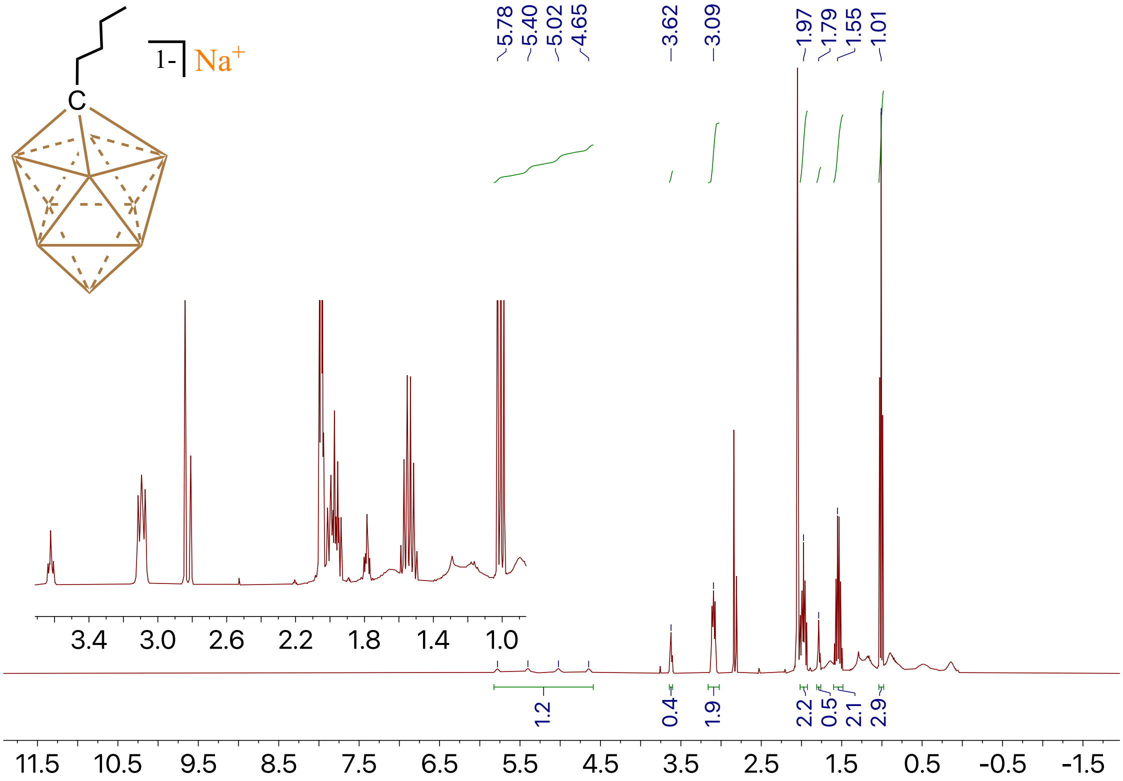

Figure S86. ^1H NMR of **NaC4** ($[Na^+][C_4H_9CB_9H_9^{1-}]$) in d_6-acetone. Note: Minor amounts of coordinated THF appears at 3.6 and 1.8 ppm. There are roughly 1 THF molecule per 10 $[Na^+][HCB_9H_9^{1-}]$.

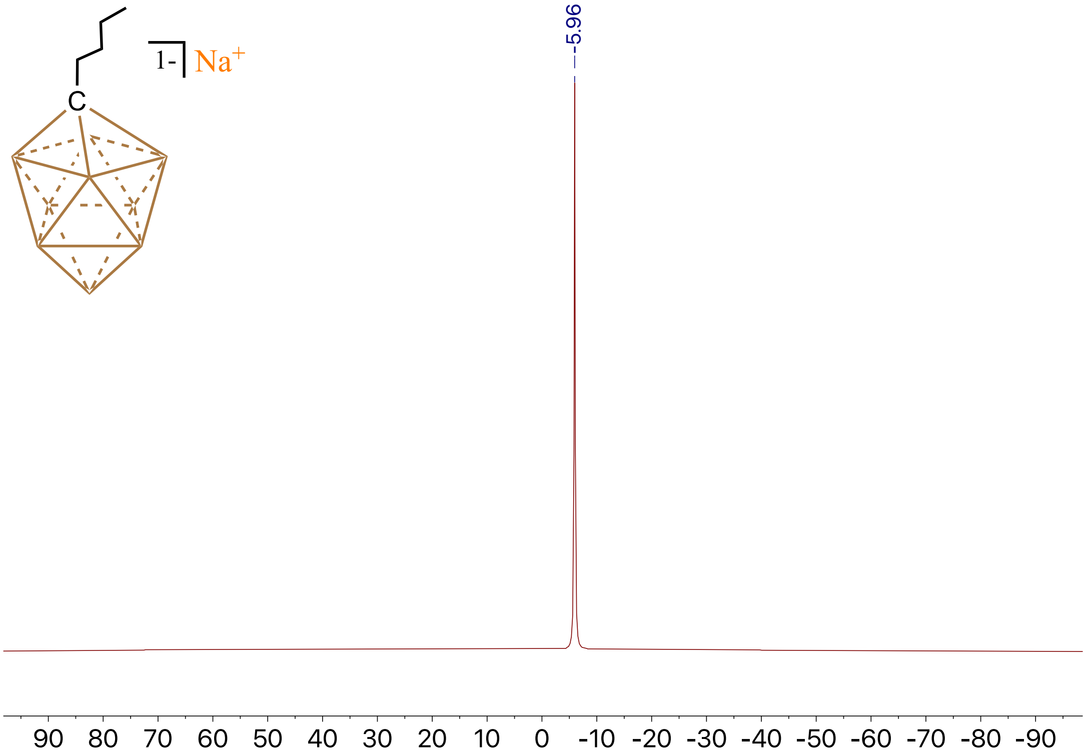

Figure S87. ^{23}Na NMR of **NaC$_4$** ([Na$^+$][$C_4H_9CB_9H_9^{1-}$]) in d_6-acetone.

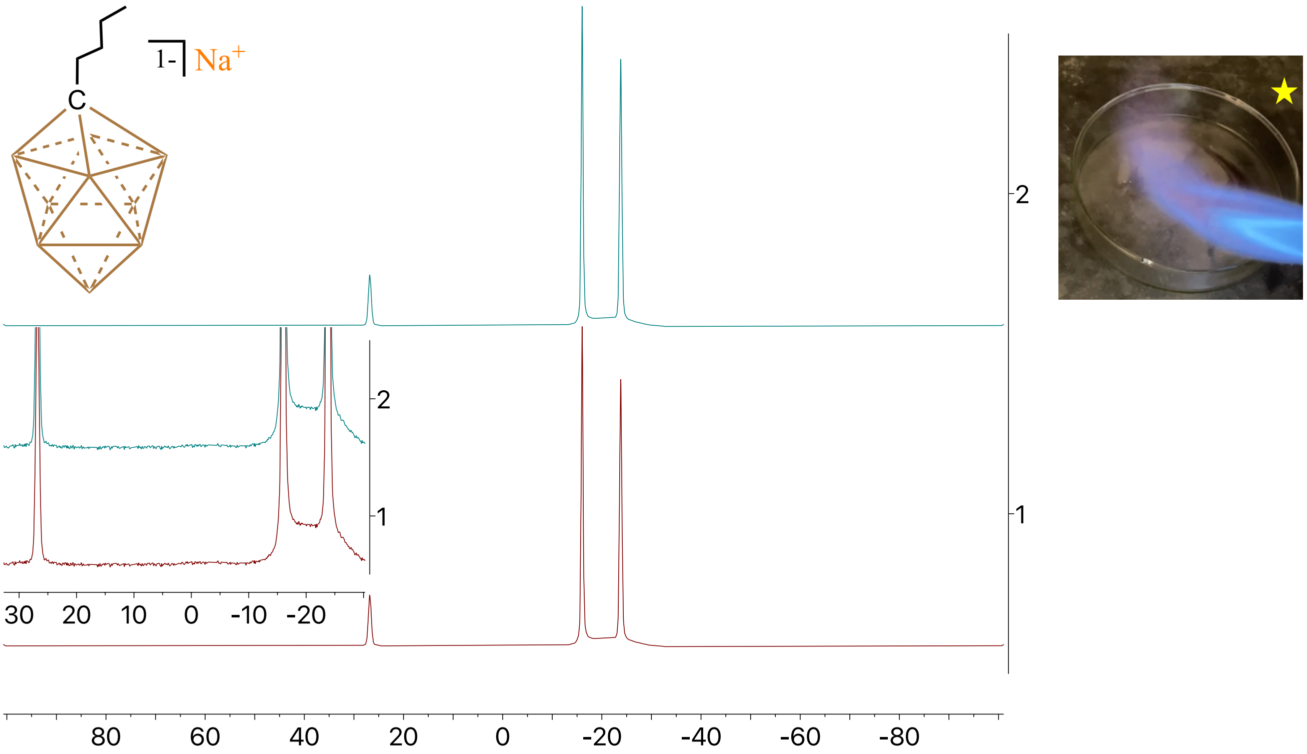

Figure S88. ^{11}B{^{1}H} NMR of **NaC$_4$** ([Na$^+$][$C_4H_9CB_9H_9^{1-}$]) before (above) and after (below) burning 3s in d_6-acetone.

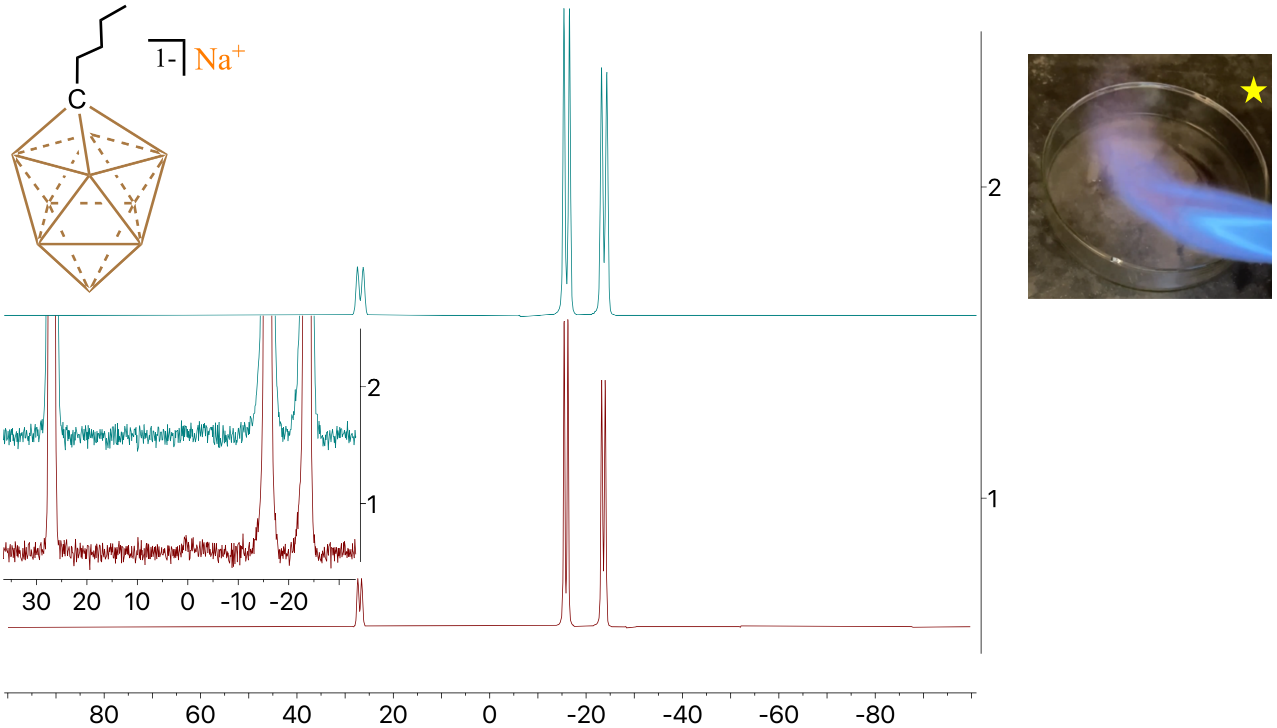

Figure S89. ^{11}B NMR of **NaC4** ($[Na^+][C_4H_9CB_9H_9{}^{1-}]$) before (above) and after (below) burning 3s in d_6-acetone.

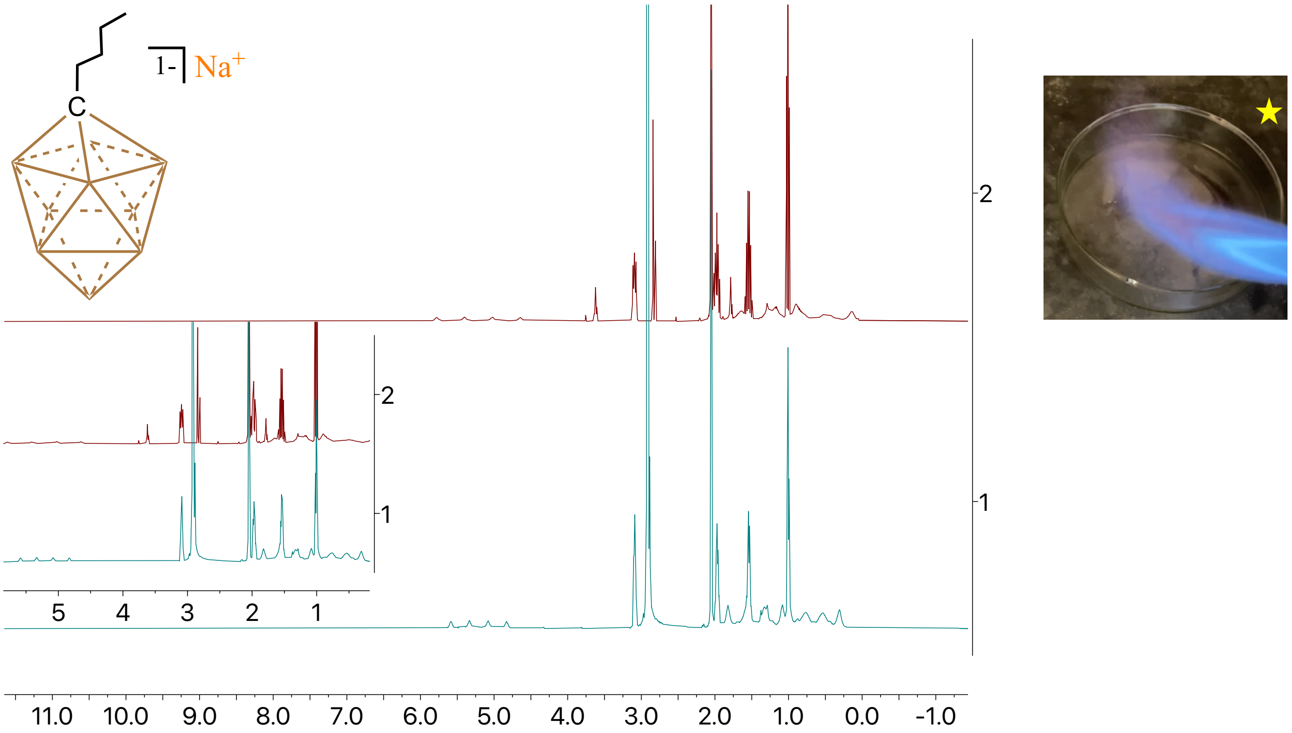

Figure S90. ^{1}H NMR of **NaC4** ($[Na^+][C_4H_9CB_9H_9{}^{1-}]$) before (above, 400 MHz) and after (below, 600 MHz) burning 3s in d_6-acetone. Note: water peaks arise at ~2.8 ppm.

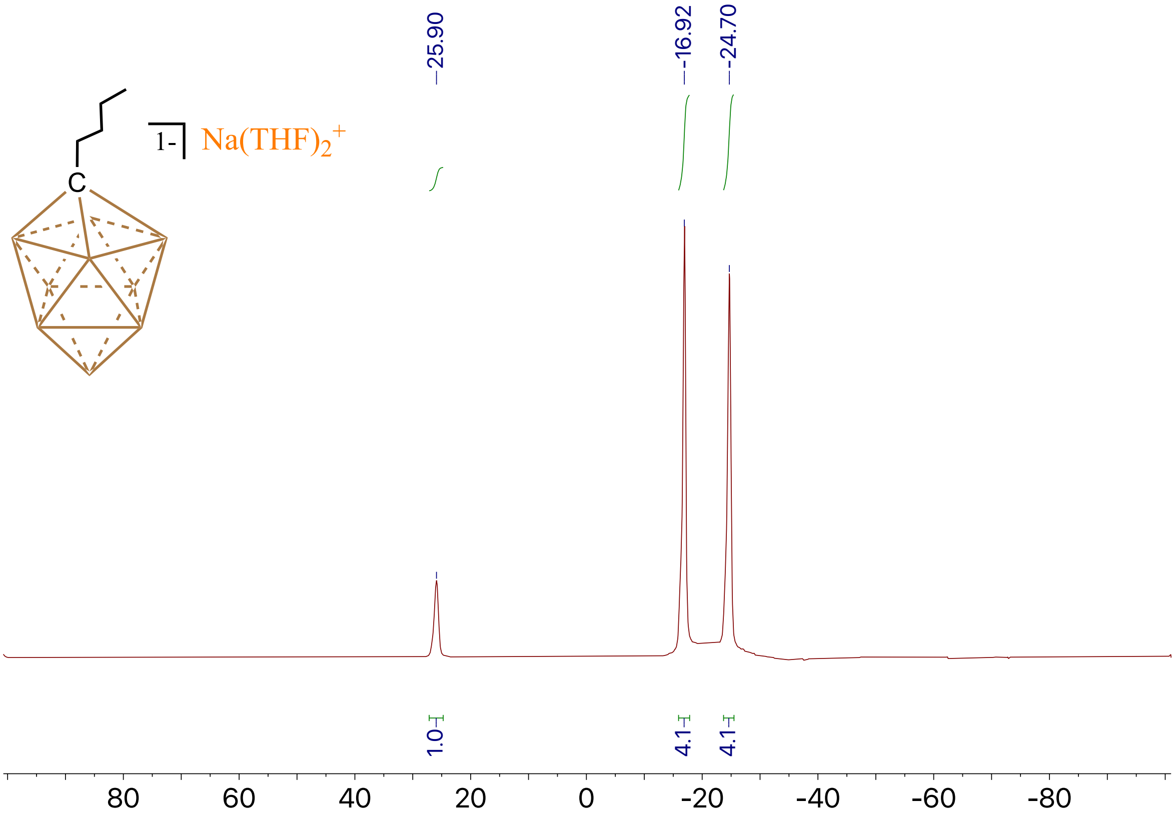

Figure S91. $^{11}B\{^{1}H\}$ NMR of **NaC$_4$-THF$_2$** ($[Na(THF)_2^{+}][C_4H_9CB_9H_9^{1-}]$) in d_6-acetone.

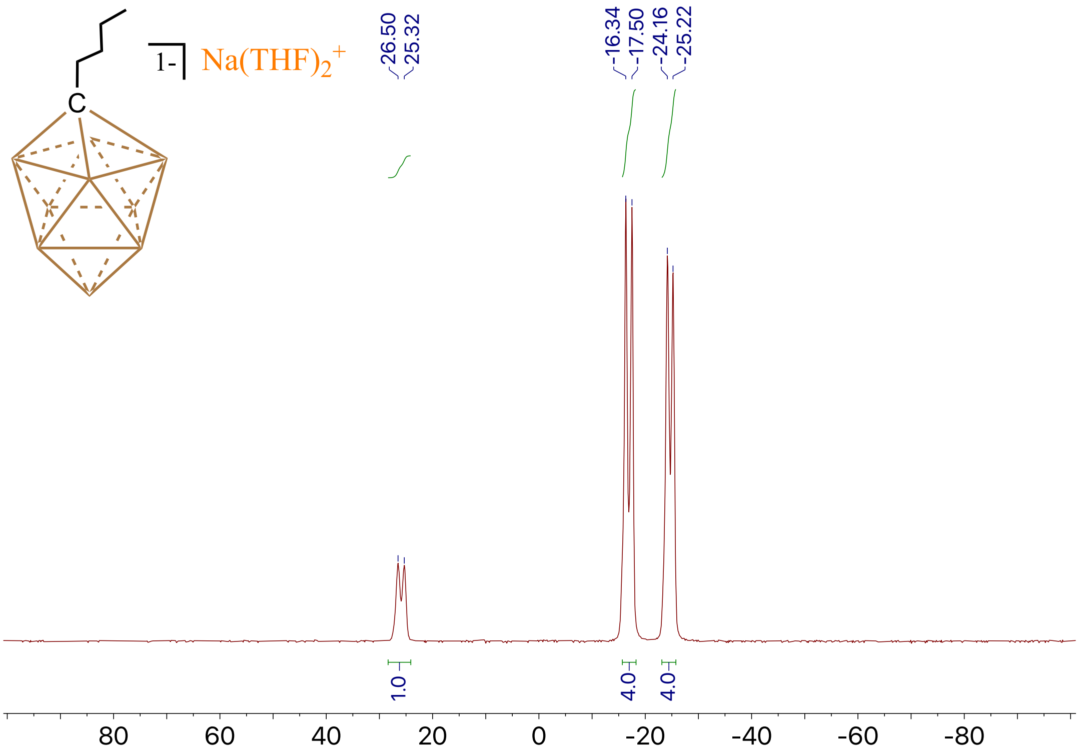

Figure S92. ^{11}B NMR of **NaC$_4$-THF$_2$** ($[Na(THF)_2^{+}][C_4H_9CB_9H_9^{1-}]$) in d_6-acetone.

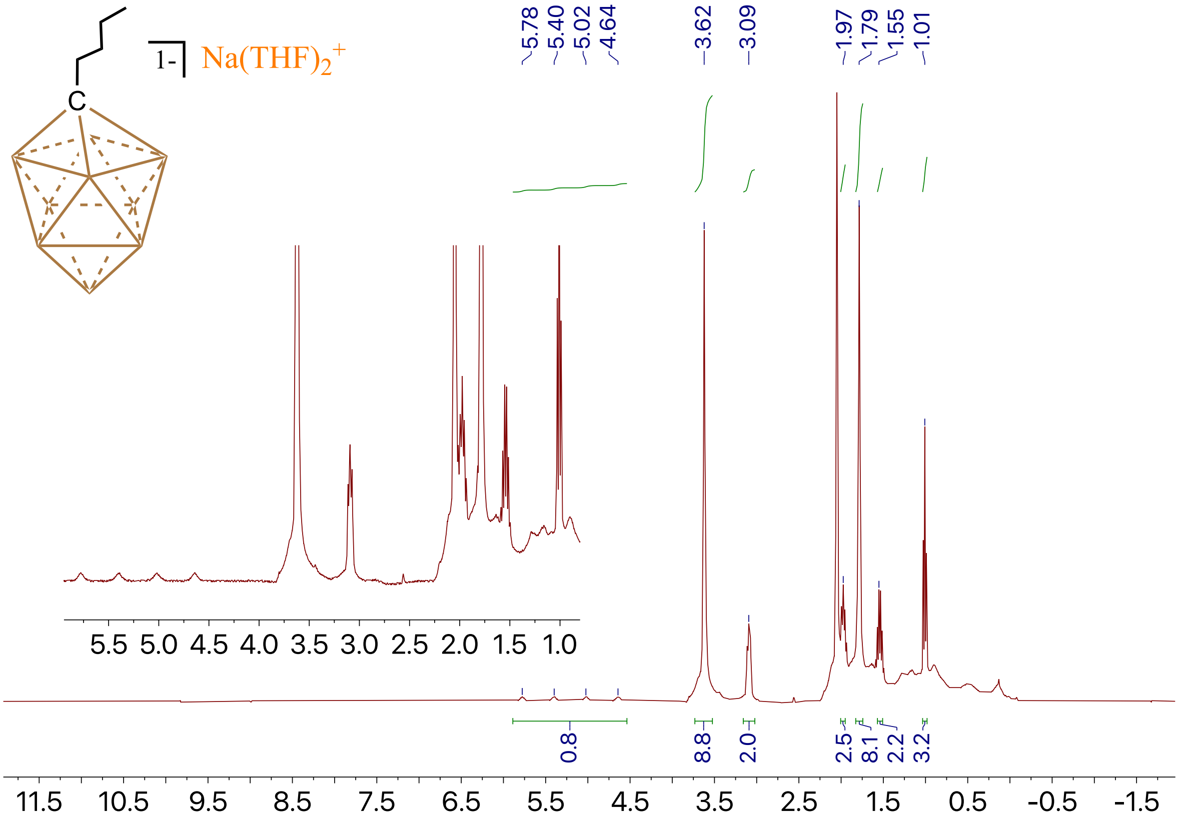

Figure S93. ^{1}H NMR of **NaC$_4$-THF$_2$** ($[Na(THF)_2{}^{+}][C_4H_9CB_9H_9{}^{1-}]$) wet d$_6$-acetone. Note: THF appears at 1.78 and 3.62 ppm.

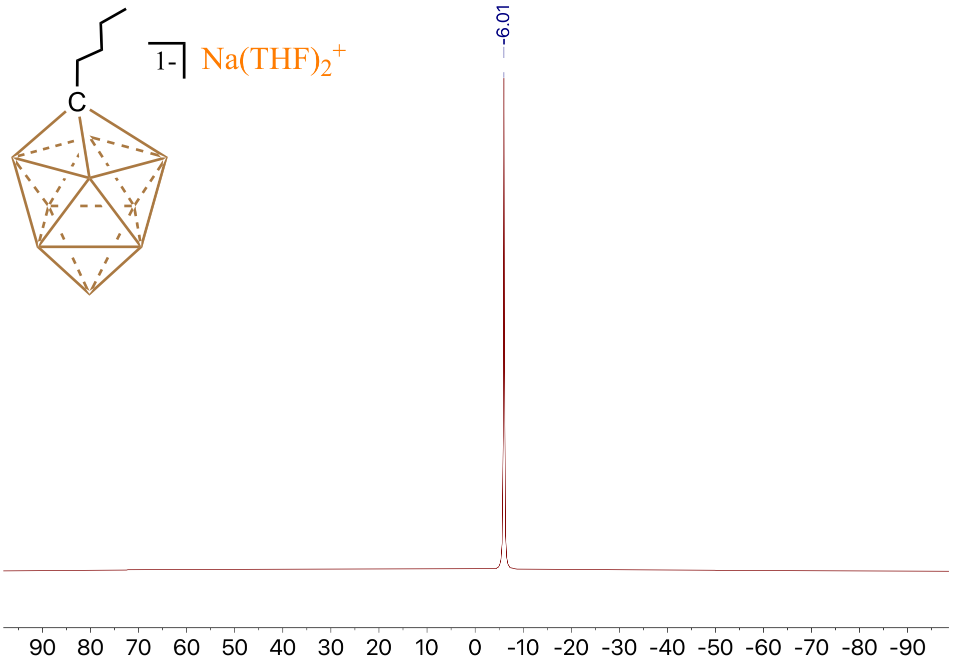

Figure S94. ^{23}Na NMR of **NaC$_4$-THF$_2$** ($[Na(THF)_2{}^{+}][C_4H_9CB_9H_9{}^{1-}]$) wet d$_6$-acetone.

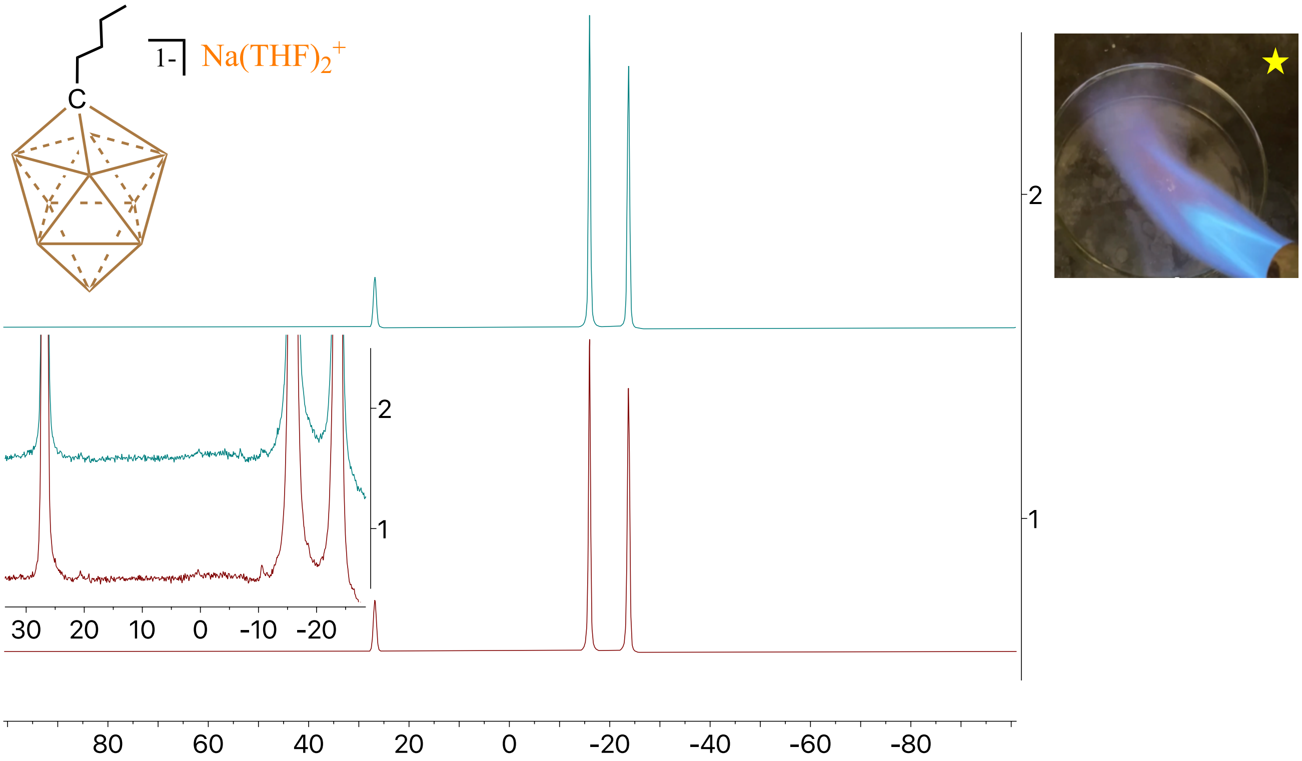

Fig S95. $^{11}B\{^1H\}$ NMR of **NaC$_4$-THF$_2$** ($[Na(THF)_2^+][C_4H_9CB_9H_9^{1-}]$) before (above) and after (below) burning 3s in d$_6$-acetone. Minor decomposition observed at 20 and -10 ppm.

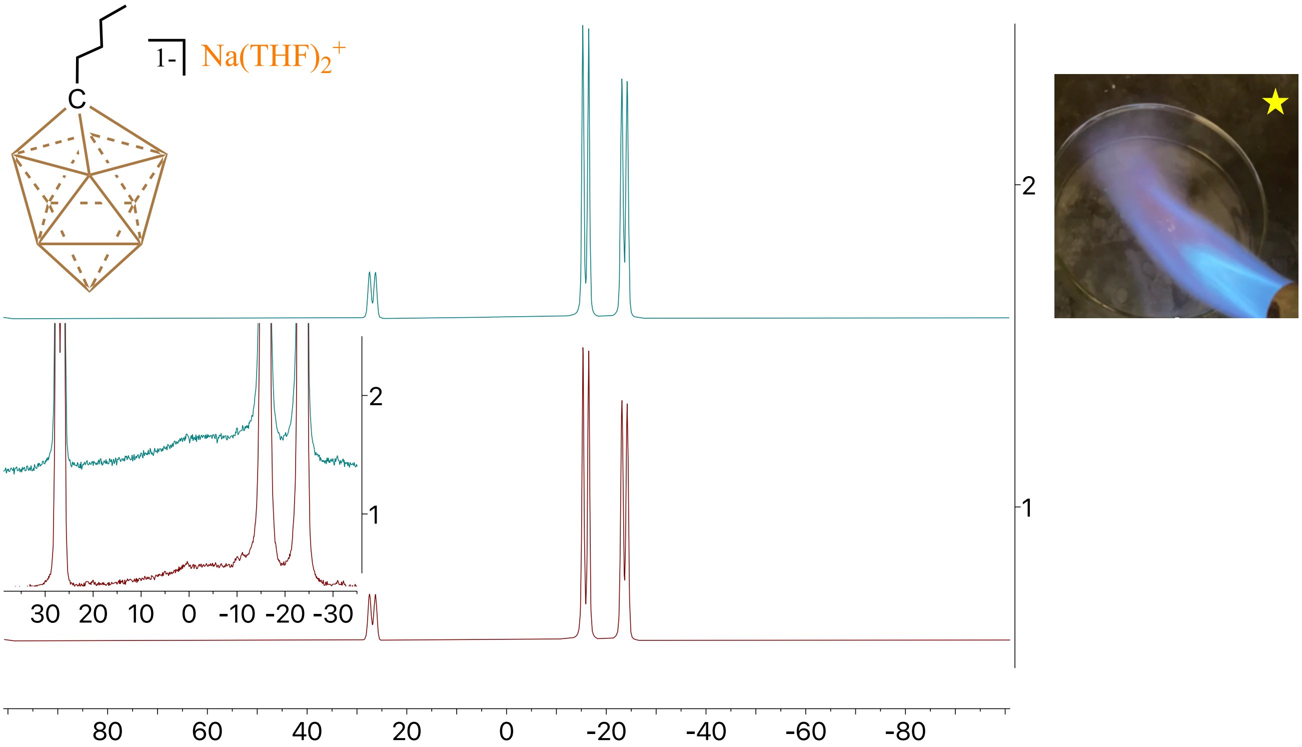

Figure S96. ^{11}B NMR of **NaC$_4$-THF$_2$** ($[Na(THF)_2^+][C_4H_9CB_9H_9^{1-}]$) before (above) and after (below) burning 3s in d$_6$-acetone. Minor decomposition observed at 20, -10, and -30 ppm.

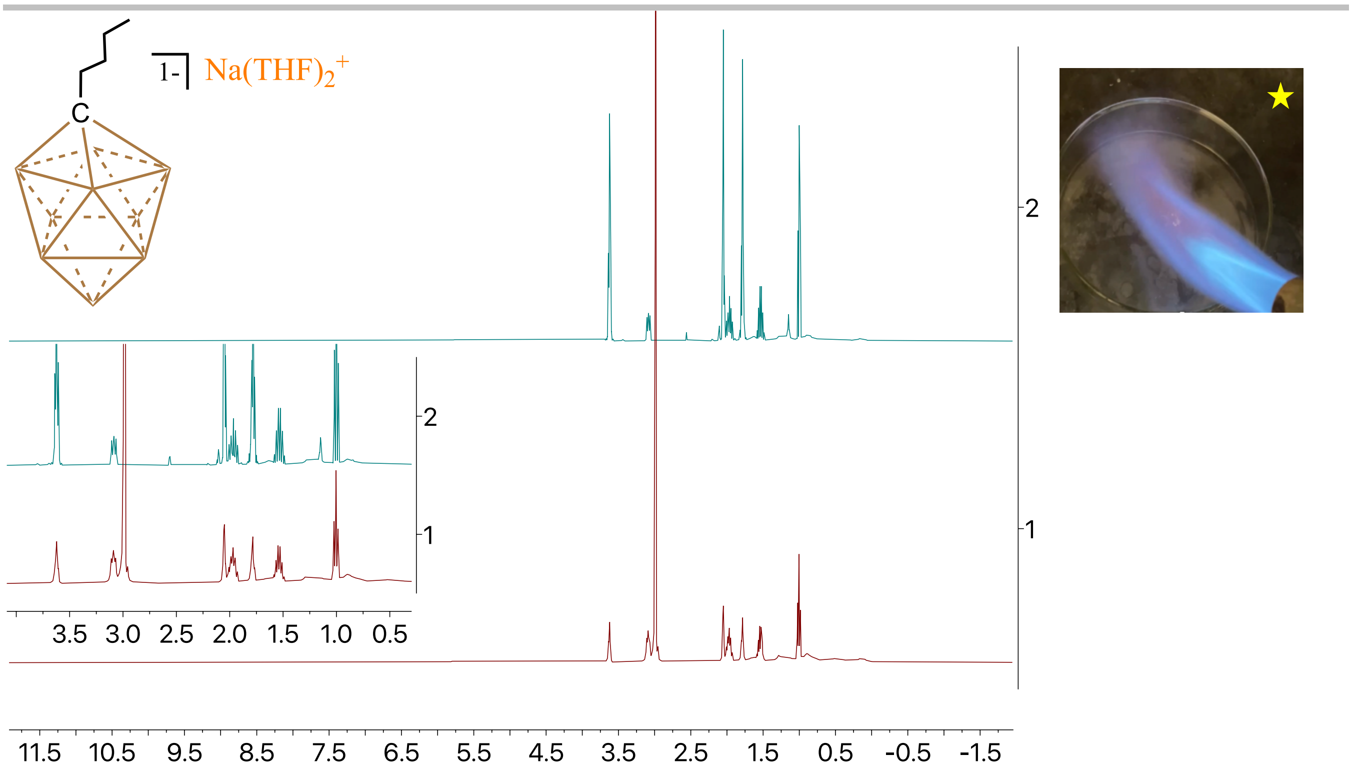

Figure S97. ^{1}H NMR of **NaC$_4$-THF$_2$** ([Na(THF)$_2^+$][$C_4H_9CB_9H_9^{1-}$]) before (above) and after (below) burning 3s in d$_6$-acetone. Note: a water peak arises at 2.8 ppm (below).

Synthesis of KC$_4$: [K$^+$][$C_4H_9CB_9H_9^{1-}$] – Figures S98 – S103

Due to the solubility of **[K$^+$][$C_4H_9CB_9H_9^{1-}$]** and KHMDS being similar and thus difficult to separate, we opted to make this salt by utilizing KH. 100 mg of [HNMe$_3^+$]1$_{butyl}$ was dissolved in 3 mL of THF. The addition of 1.5 molar equiv. KH yielded H_2 and NMe_3 gases. The reaction was allowed to stir overnight to ensure completeness. The KH was filtered out of the solution, and the solvent removed in vacuo to yield a white salt, THF-free [K$^+$][$C_4H_9CB_9H_9^{1-}$] (yield = 81 mg, 89%, m.p. = 260.4 – 262.3 °C).

^{11}B{^{1}H} NMR (128 MHz, d$_6$-acetone) δ 26.68 (s, 1 B-H), -16.05 (s, 4 B-H), -23.82 (s, 4 B-H). ^{11}B NMR (128 MHz, d$_6$-acetone) δ 26.70 (d, J = 150.1 Hz, 1 B-H), -16.05 (d, J = 147.1 Hz, 4 B-H), -23.82 (d, J = 133.9 Hz, 4 B-H). ^{1}H NMR (400 MHz, d$_6$-acetone) δ 5.19 (q, J = 150.1, 1 B-H), 3.13 – 3.05 (m, 2H), 2.03 – 1.91 (m, 2H), 1.54 (h, J = 8.0 Hz, 2H), 1.00 (t, J = 7.4 Hz, 3H).

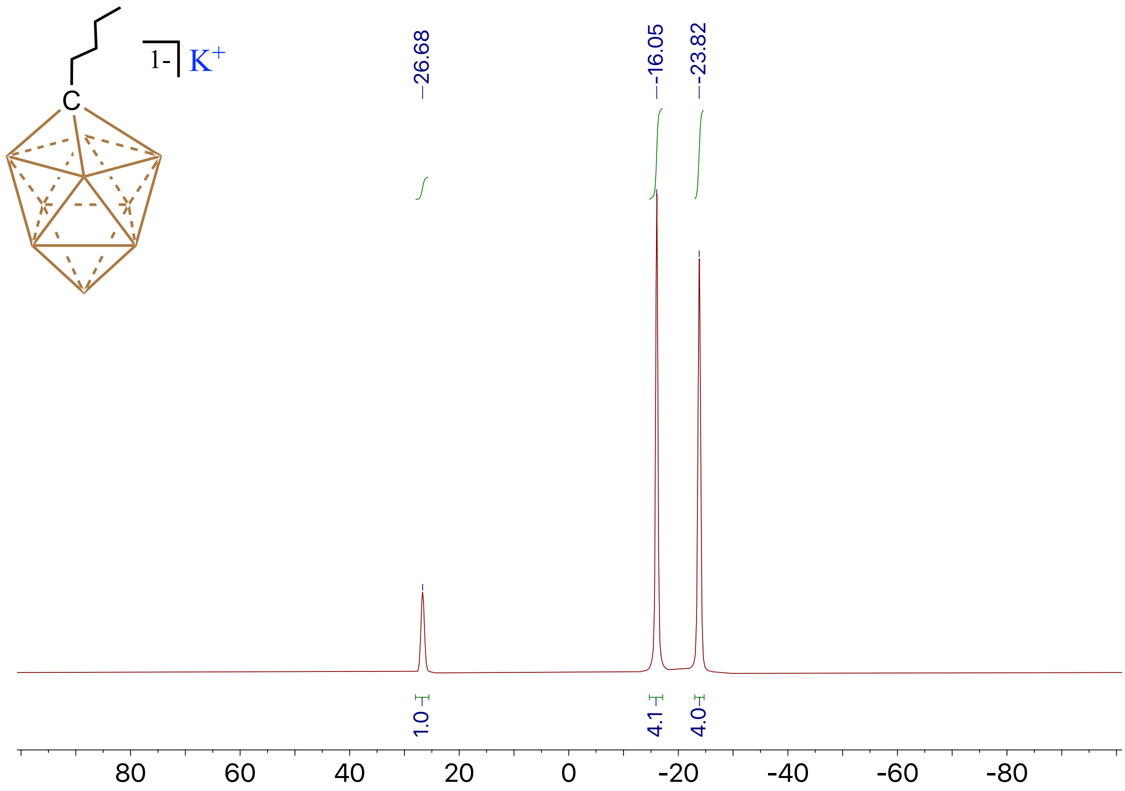

Figure S98. $^{11}B\{^{1}H\}$ NMR of **KC**$_4$ ($[K^+][C_4H_9CB_9H_9{}^{1-}]$) d$_6$-acetone.

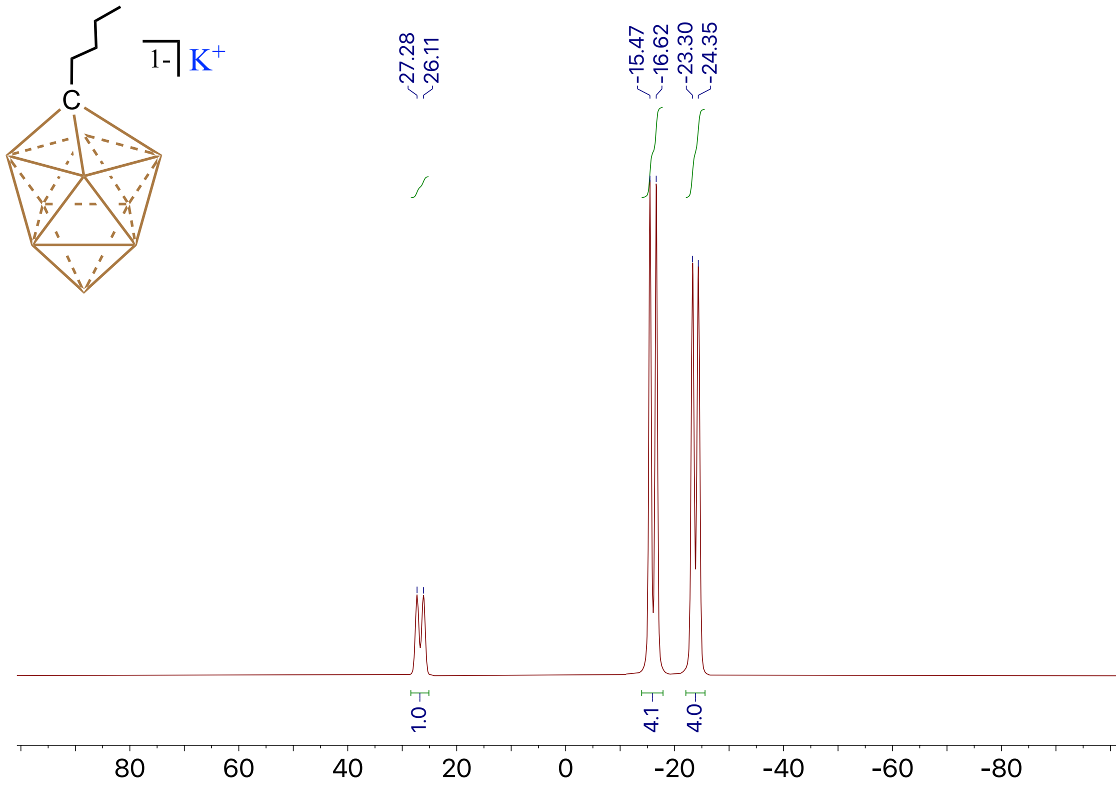

Figure S99. $^{11}B\{^{1}H\}$ NMR of **KC**$_4$ ($[K^+][C_4H_9CB_9H_9{}^{1-}]$) d$_6$-acetone.

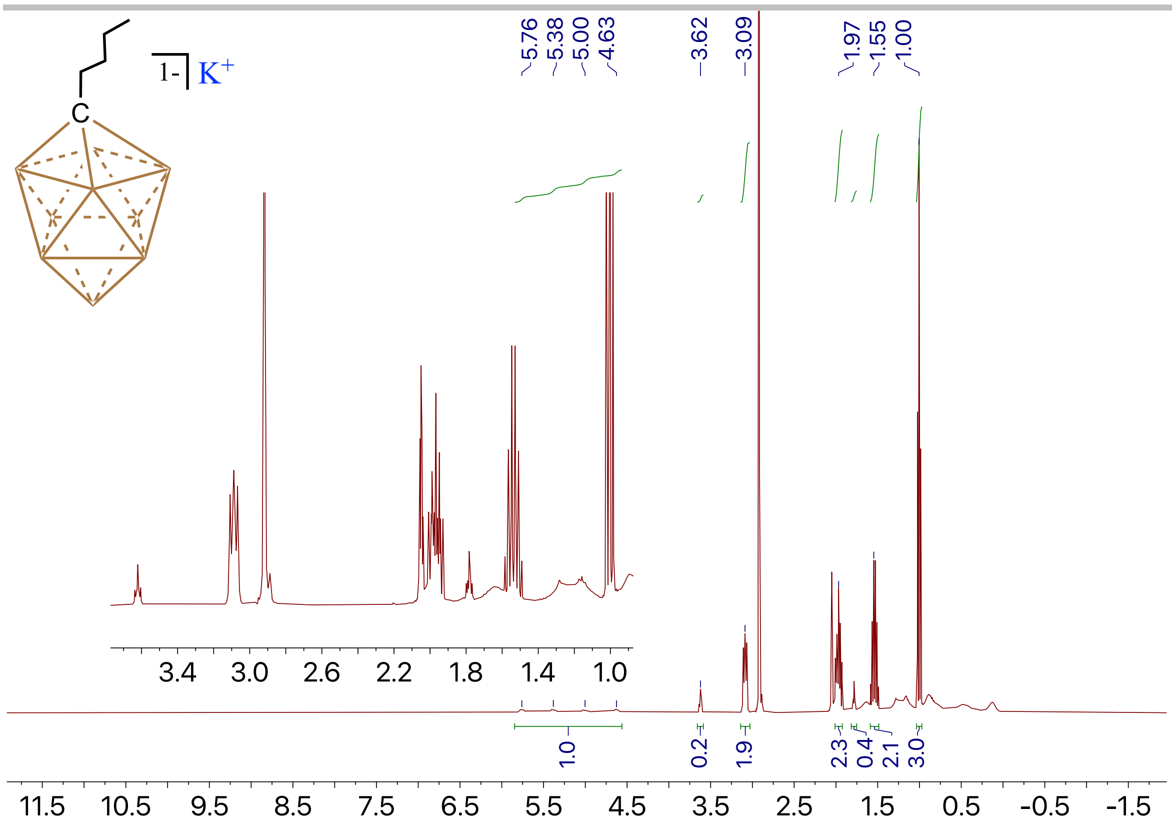

Figure S100. ^{1}H NMR of **KC_4** ($[K^+][C_4H_9CB_9H_9{}^{1-}]$) wet d_6-acetone. Note: Minor amounts of THF appears at 1.78 and 3.62 ppm. There are ~5 THF molecules per 100 **$[K^+][C_4H_9CB_9H_9{}^{1-}]$**.

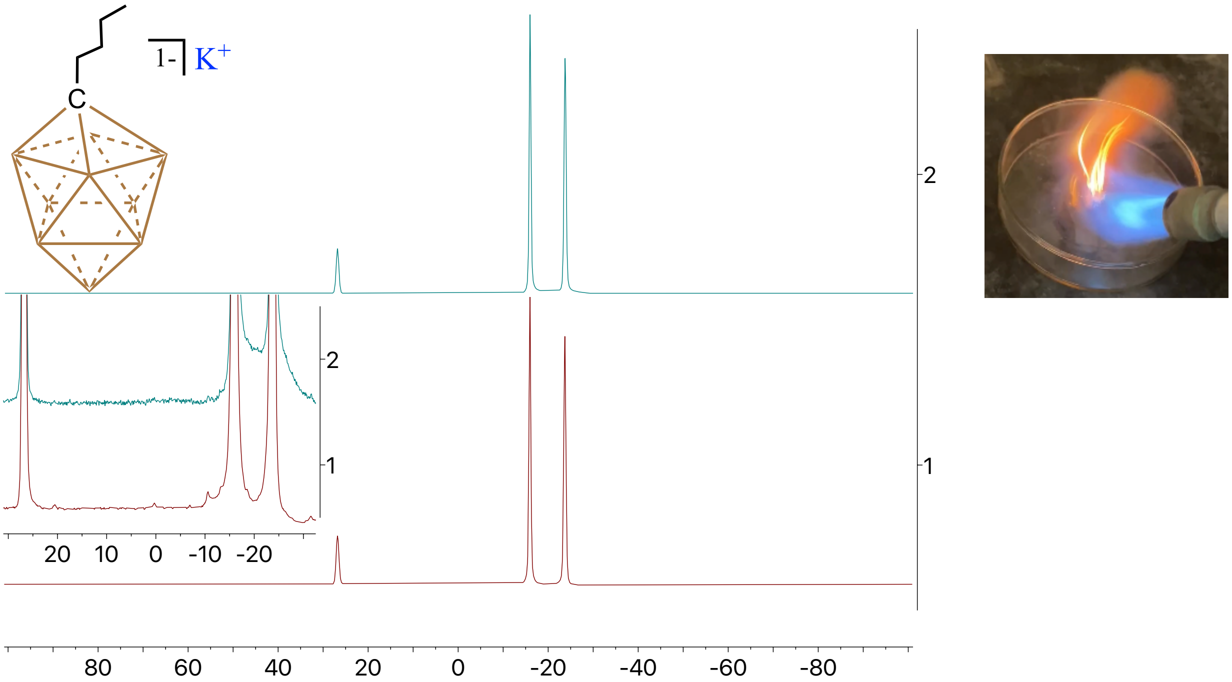

Figure S101. $^{11}B\{^{1}H\}$ NMR of **KC_4** ($[K^+][C_4H_9CB_9H_9{}^{1-}]$) before (above) and after (below) burning 3s in d_6-acetone. Minor decomposition observed at 20, 0, -10 ppm.

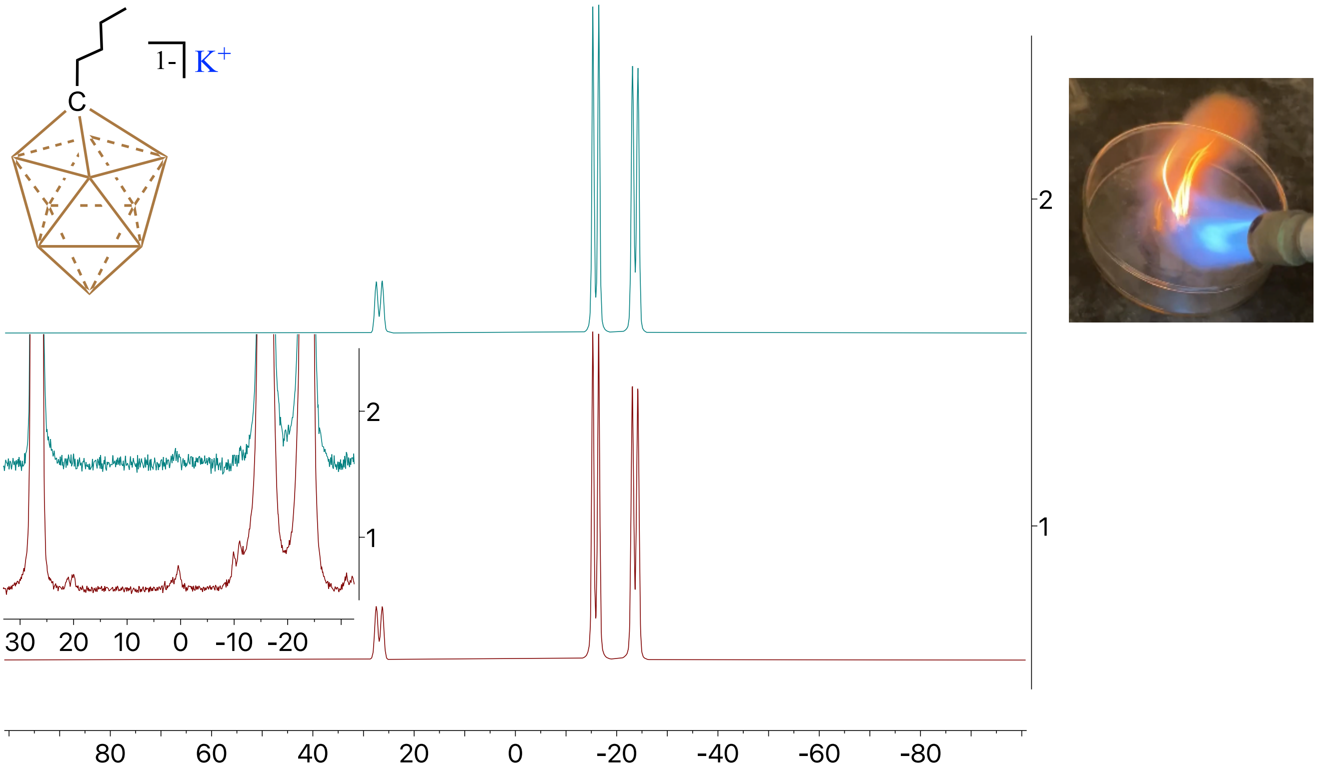

Figure S102. ^{11}B NMR of **KC4** ($[K^+][C_4H_9CB_9H_9{}^{1-}]$) before (above) and after (below) burning 3s in d_6-acetone. Minor decomposition observed at 20, 0, -10 ppm.

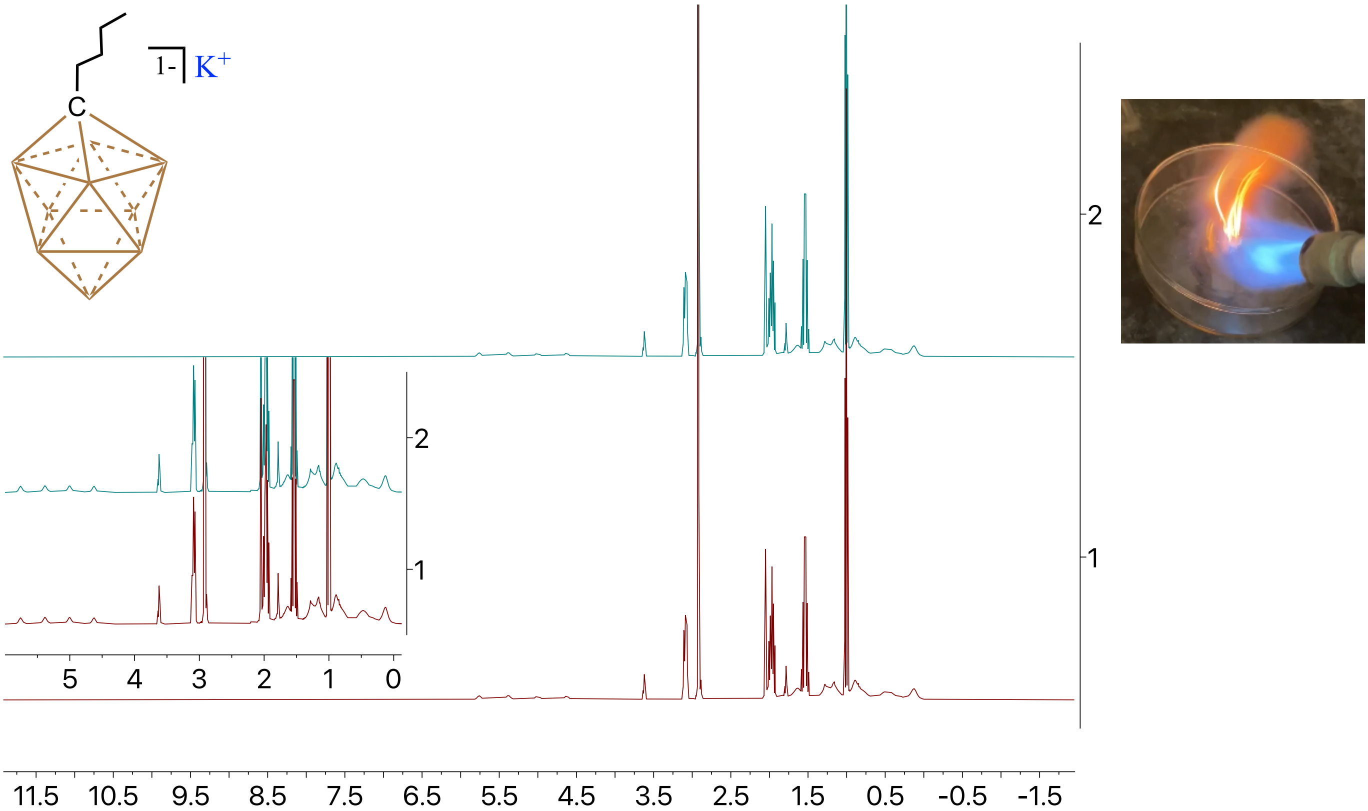

Figure S103. ^1H NMR of **KC4** ($[K^+][C_4H_9CB_9H_9{}^{1-}]$)before (above) and after (below) burning 3s in d_6-acetone. Note: water peaks arise at 2.9 ppm.

Synthesis of $[Cs^+][C_{10}H_{21}CB_9H_9^{1-}]$ from $[HNMe_3^+][HCB_9H_9^{1-}]$

A similar alkylation procedure was followed for the preparation of **$[HNMe_3^+][C_{10}H_{21}CB_9H_9^{1-}]$**. 2.0 g (11.1 mmol) was dissolved in 50 mL of THF. In a separate container, 9.8 mL (24.4 mmol, 2.2 eq.) of 2.5 M n-BuLi solution in hexane was concentrated in-vacuo and added dropwise to the stirring THF solution. Following isolation of the dianionic solid, 2.8 mL (1.2 eq.) 1-bromo-decane was added to the solid followed by 20 mL of THF. The workup, purification, and salt metathesis procedures are identical to those described in procedure **2** above. Yield **$[Cs^+][C_{10}H_{21}CB_9H_9^{1-}]$** : 3.86 g (88.7 %).

Synthesis of LiC_{10}-THF_n : $[Li(THF)_n^+][C_{10}H_{21}CB_9H_9^{1-}]$ where n = 0, 1, 4. – Figures S104 – S117

$[HNMe_3^+][C_{10}H_{21}CB_9H_9^{1-}]$ was dried *in-vacuo* at 80°C for 24 hours prior to use. Working in a glovebox, 5.0 g of **$[HNMe_3^+][C_{10}H_{21}CB_9H_9^{1-}]$** was dissolved in a 50 mL of THF. LiH (1.5 g, excess) was added slowly until the effervescence of NMe_3 and H_2 gaseous biproducts seized. The solution was left to stir for 3 hours to ensure reaction completion. The slurry was filtered twice through a Whatman GF/A grade glass microfiber filter to remove excess LiH. The resulting clear solution was concentrated in-vacuo to yield **LiC_{10}-THF_4** as a fluffy white powder (9.84 g, 98.5% yield). The powder was subsequently heated at 130 °C in-vacuo for 16 hours while stirring to yield **LiC_{10}-THF.** The presence of 1 molar equ. of THF:Li^+ was confirmed by ^{1}H NMR with peak integration referenced to the methylene (CH_2) at δ = 3.2 ppm. To remove all THF, this salt was heated at 190 °C in-vacuo for 3 days while stirring to yield **LiC_4**. Loss of THF was observed by ^{1}H NMR.

n=0. ^{11}B{^{1}H} NMR (96 MHz, $CDCl_3$, 25°C): ∂ = 13.80, -15.96, -23.52 ppm. ^{11}B NMR (96 MHz, $CDCl_3$, 25°C): ∂ = 13.86, -15.85, -23.48 ppm. ^{1}H NMR (600 MHz, $CDCl_3$, 25°C; 3.20 (t, 2H), 2.00 (m, 2H) ppm, 1.55 – 1.30 (m, 14H), 0.91 (t, 3H) ppm. ^{1}H{^{11}B} NMR (600 MHz, $CDCl_3$, 25°C): ∂ = 5.10 (bs, 1H, B-H), 3.20 (t, 2H), 2.0 (m, 2H), 1.82 (bs, 4H, B-H), 1.55 (m, 2H), 1.45 – 1.32 (m, 14H), 0.92 (t, 3H), 0.80 (bs, 4H, B-H) ppm.

n=1. ^{11}B{^{1}H} NMR (96 MHz, $CDCl_3$, 25°C): ∂ = 17.34, -16.03, -23.65 ppm. ^{11}B NMR (96 MHz, $CDCl_3$, 25°C): ∂ = 17.64, -15.69, -23.33 ppm. ^{1}H NMR (600 MHz, $CDCl_3$, 25°C): ∂ = 3.87 (m, 4H), 3.20 (t, 2H), 2.03 -1.94 (m, 6H) ppm, 1.56 – 1.32 (m, 16H), 0.92 (t, 3H) ppm. ^{1}H{^{11}B} NMR (600 MHz, $CDCl_3$, 25°C): ∂ = 4.89 (bs, 1H, B-H), 3.87 (m, 4H), 3.21 (t, 2H), 2.04 – 1.95 (m, 6H), 1.75 (bs, 4H, B-H), 1.57 (m, 2H), 1.45 – 1.32 (m, 14H), 0.92 (t, 3H), 0.79 (bs, 4H, B-H) ppm. ^{13}C NMR (151 MHz, $CDCl_3$, 25°C): ∂ = 86.07, 69.14, 34.25, 32.34, 31.98, 30.00, 29.76, 29.74, 29.72, 29.42, 25.39, 22.75, 14.17 ppm. ^{7}Li NMR (233 MHz, $CDCl_3$, 25°C): ∂ = 0.08 ppm.

n=4. ^{11}B{^{1}H} NMR (96 MHz, $CDCl_3$, 25°C): ∂ = 22.03, -16.98, -24.85 ppm. ^{11}B NMR (96 MHz, $CDCl_3$, 25°C): ∂ = 22.05, -16.97, -24.84 ppm. ^{1}H NMR (600 MHz, $CDCl_3$, 25°C): ∂ = 3.83 (m, 16H), 3.16 (t, 2H), 2.04 -1.94 (m, 18H) ppm, 1.55 (m, 2H), 1.4-1.25 (m, 12H), 0.90 (t, 3H) ppm. ^{1}H{^{11}B} NMR (600 MHz, $CDCl_3$, 25°C): ∂ = 4.96 (bs, 1H, B-H), 3.83 (m, 16H), 3.16 (t, 2H), 2.06 – 1.94 (m, 18H), 1.57 (m, 2H), 1.56 (bs, 4H, B-H) 1.45 – 1.25 (m, 12H), 0.90 (t, 3H), 0.67 (bs, 4H, B-H) ppm. ^{13}C NMR (151 MHz, $CDCl_3$, 25°C): ∂ = 86.07, 69.14, 34.25, 32.34, 31.98, 30.00, 29.76, 29.74, 29.72, 29.42, 25.39, 22.75, 14.17 ppm. ^{7}Li NMR (233 MHz, $CDCl_3$, 25°C): ∂ = 0.08 ppm

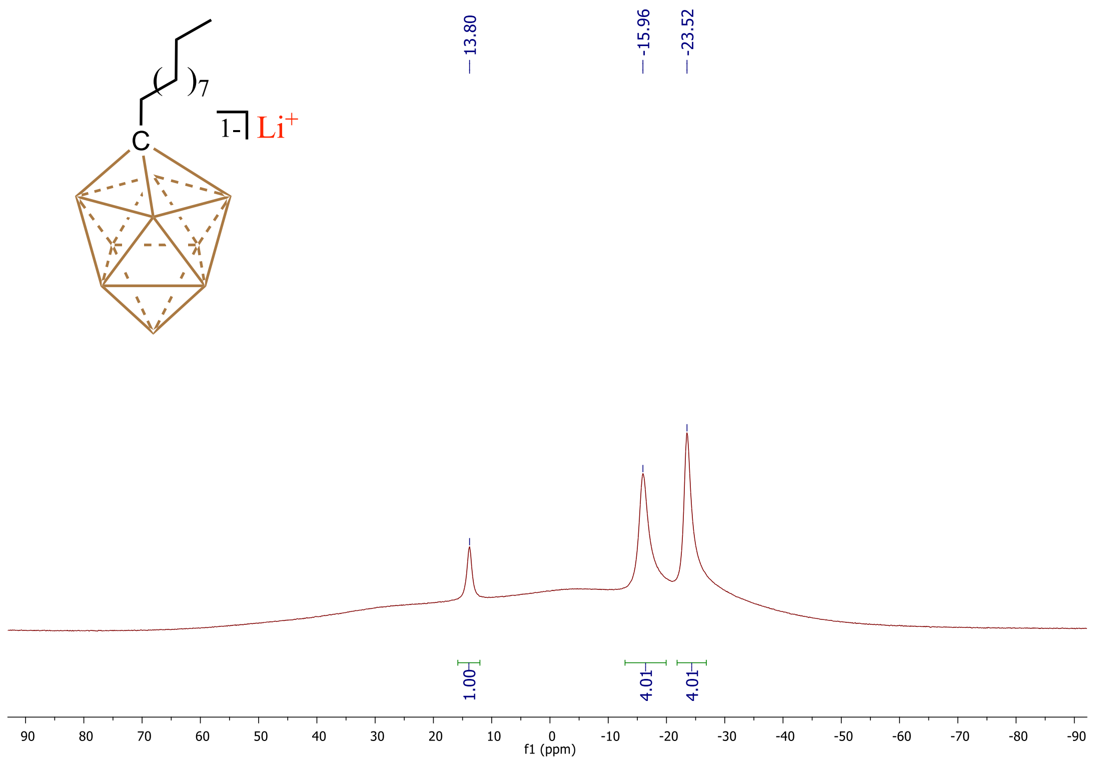

Figure S104. $^{11}B\{^{1}H\}$ NMR spectra of **LiC10** ($[Li^{+}][C_{10}H_{21}CB_{9}H_{9}^{1-}]$) in anhydrous $CDCl_3$

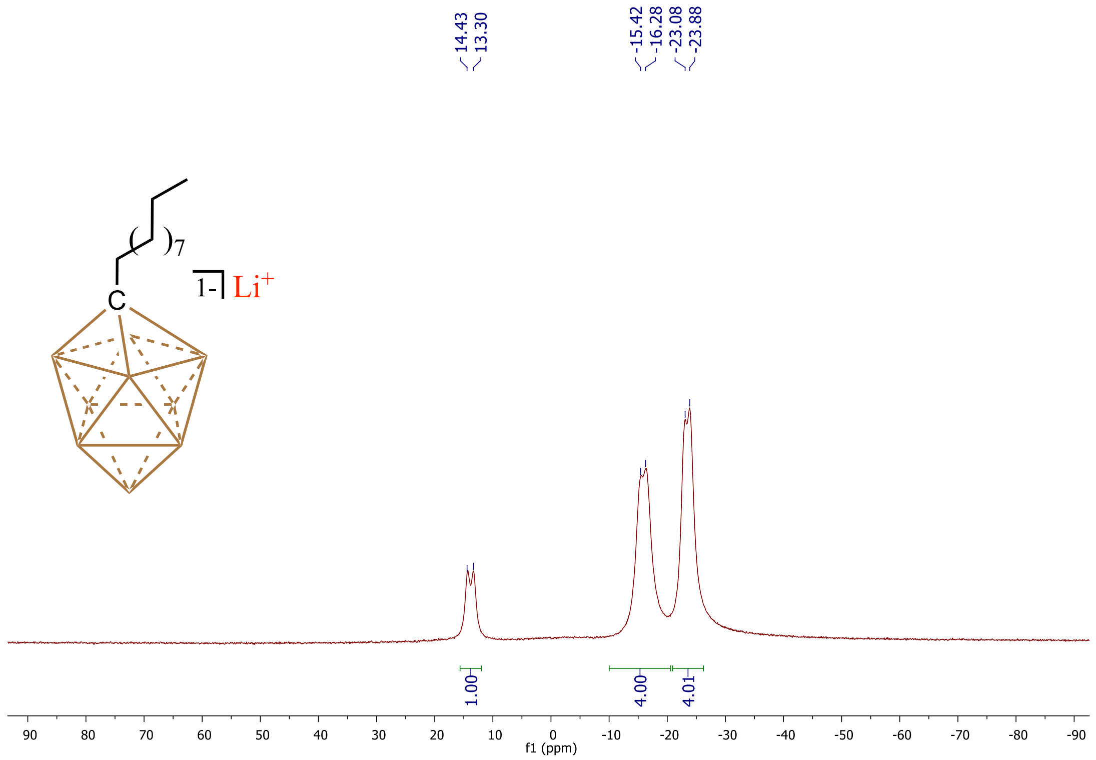

Figure S105. ^{11}B NMR spectra of **LiC10** ($[Li^{+}][C_{10}H_{21}CB_{9}H_{9}^{1-}]$) in anhydrous $CDCl_3$

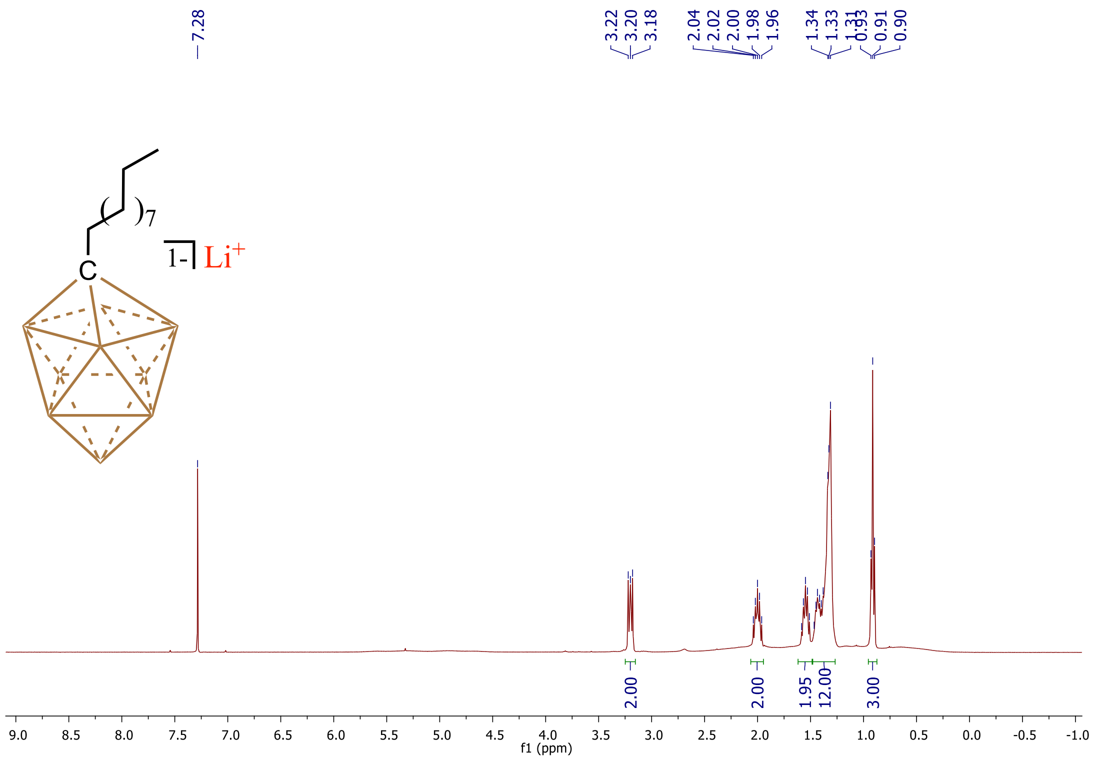

Figure S106. ^{1}H NMR spectra of **LiC10** ([Li^{+}][$C_{10}H_{21}CB_9H_9{}^{1-}$]) in anhydrous $CDCl_3$

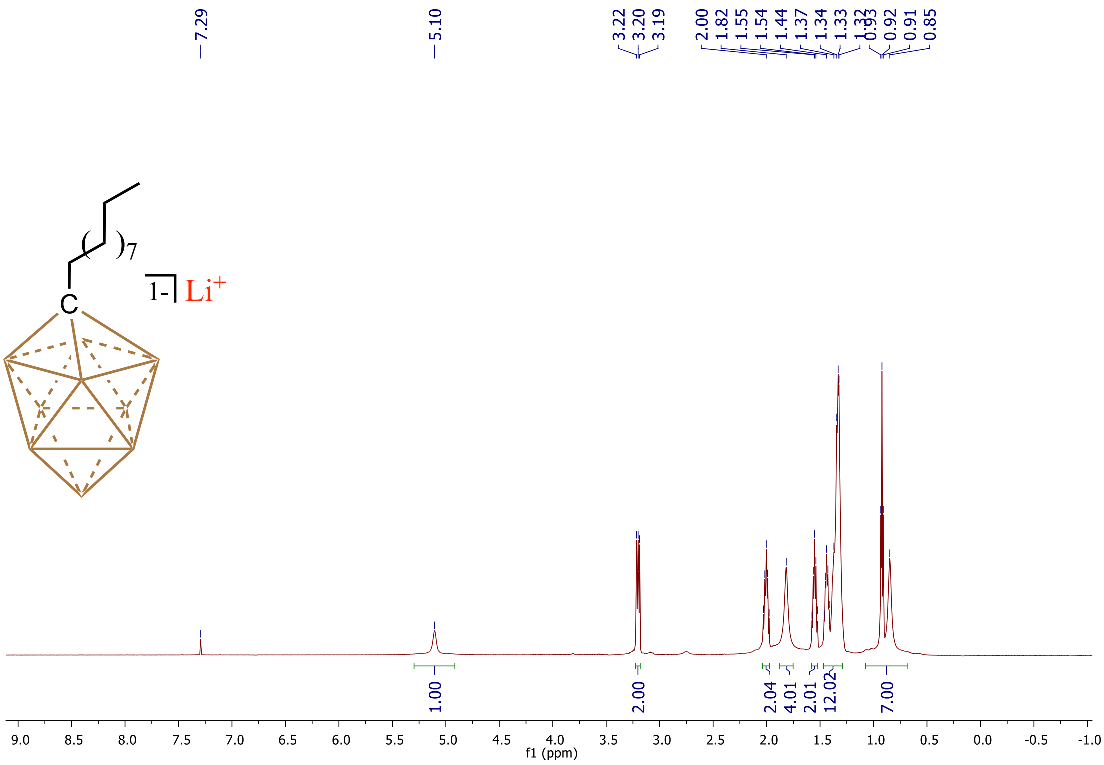

Figure S107. $^{1}H\{^{11}B\}$ NMR spectra of **LiC10** ([Li^{+}][$C_{10}H_{21}CB_9H_9{}^{1-}$]) in anhydrous $CDCl_3$

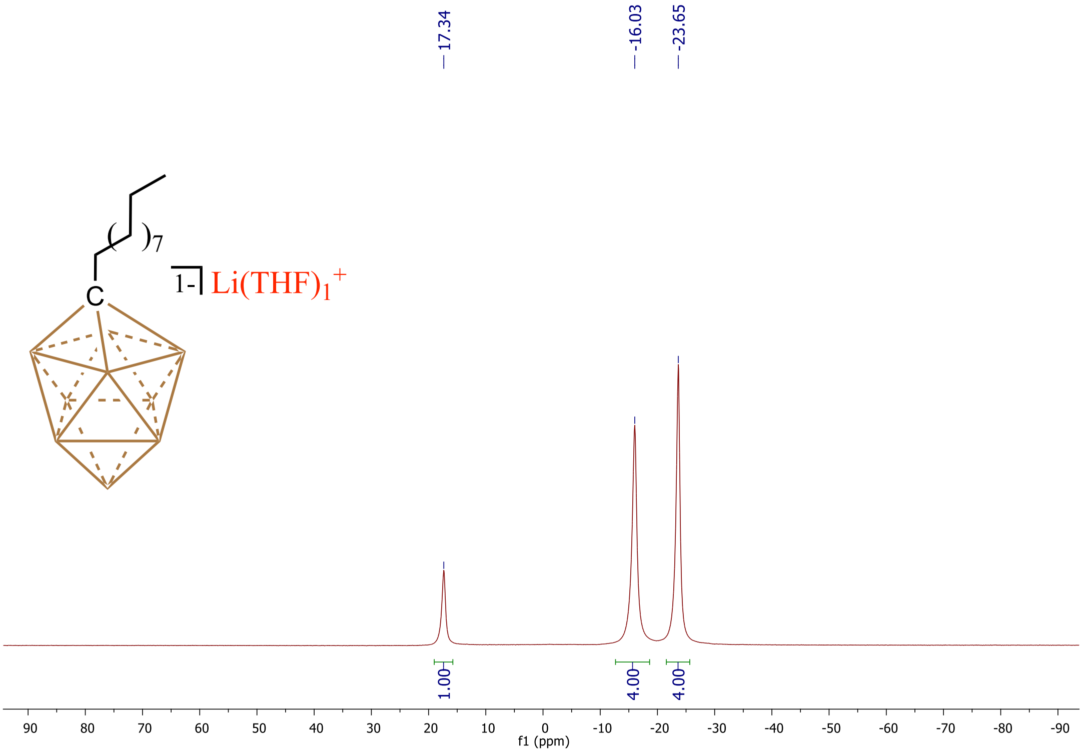

Figure S108. $^{11}B\{^{1}H\}$ NMR spectra of **LiC10-THF** ($[Li(THF)_1{}^{+}][C_{10}H_{21}CB_9H_9{}^{1-}]$) in anhydrous $CDCl_3$

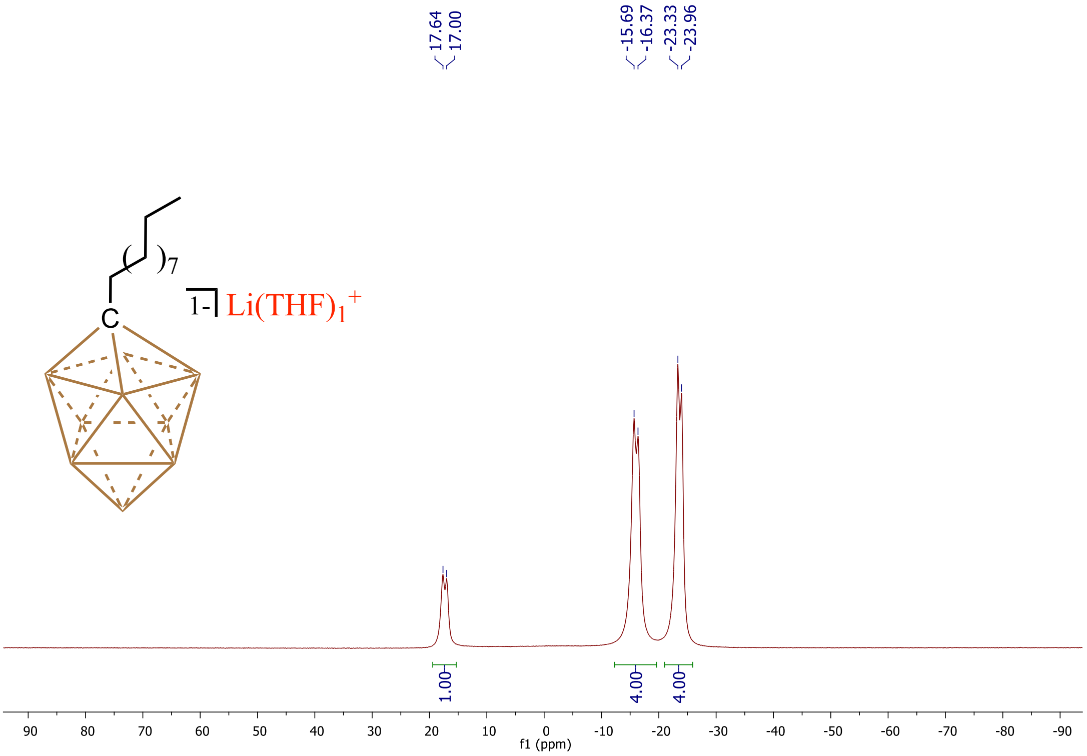

Figure S109. ^{11}B NMR spectra of **LiC10-THF** ($[Li(THF)_1{}^{+}][C_{10}H_{21}CB_9H_9{}^{1-}]$) in anhydrous $CDCl_3$

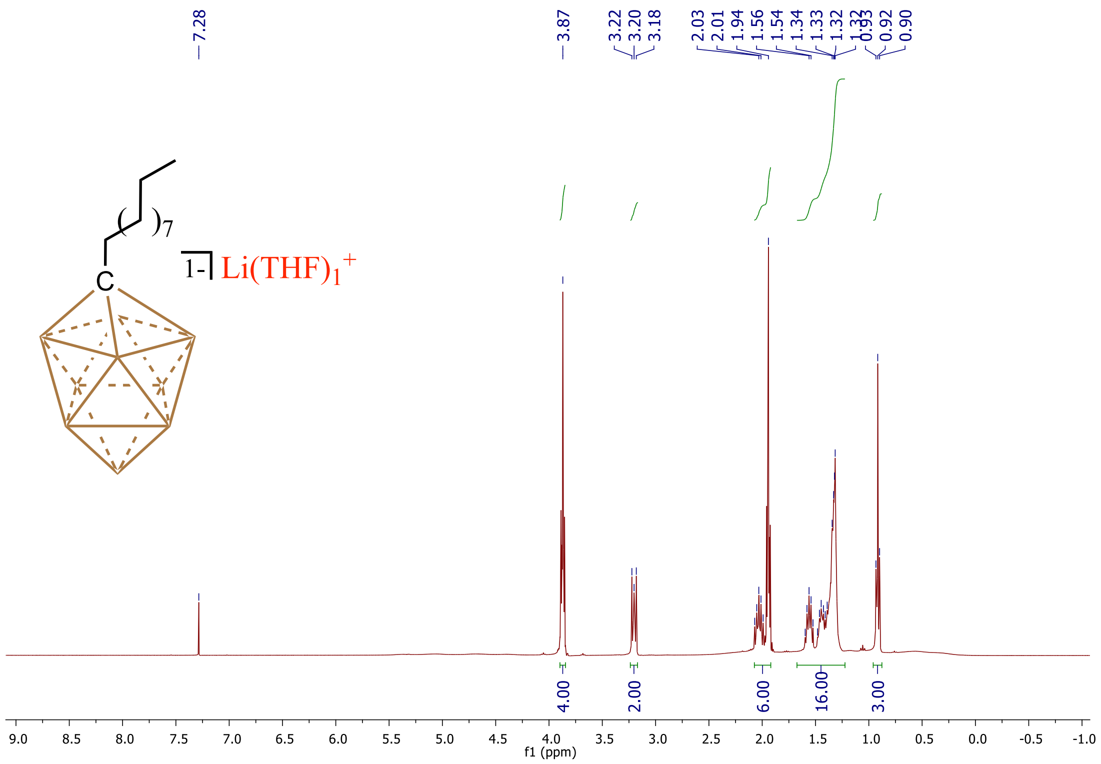

Figure S110. ^{1}H NMR spectra of **LiC$_{10}$-THF** ([Li(THF)$_1$$^+$][$C_{10}H_{21}CB_9H_9$$^{1-}$]) in anhydrous $CDCl_3$

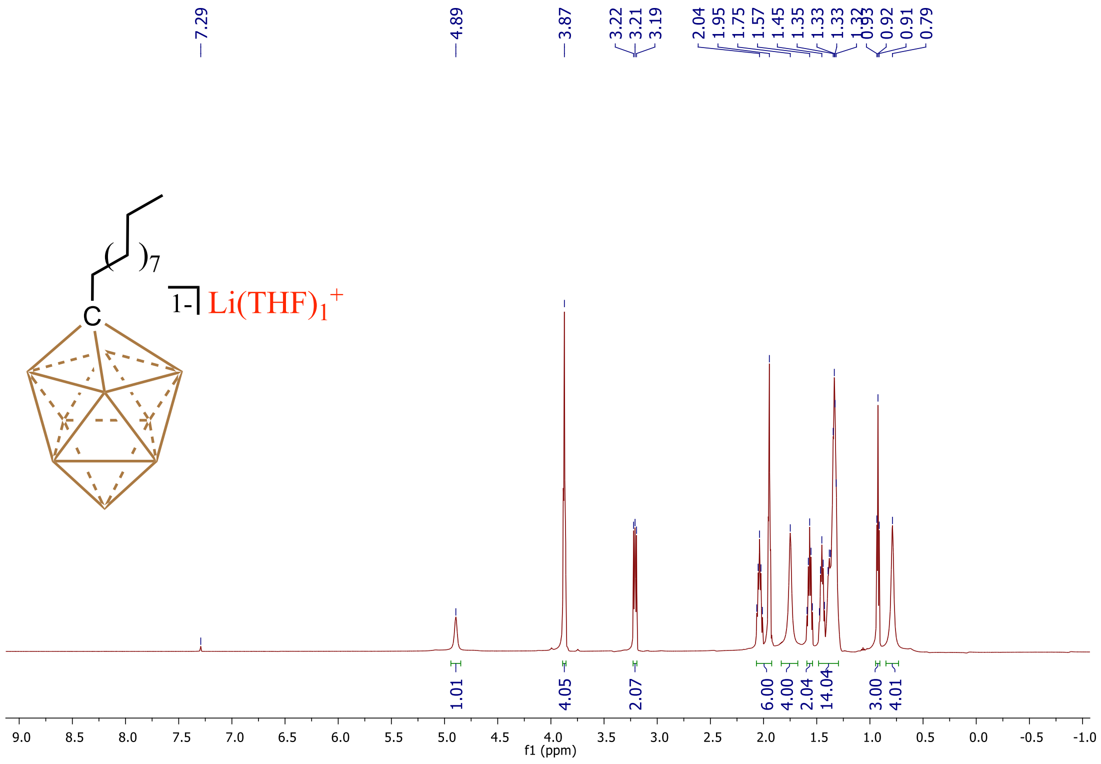

Figure S111. ^{1}H{^{11}B} NMR spectra of **LiC$_{10}$-THF** ([Li(THF)$_1$$^+$][$C_{10}H_{21}CB_9H_9$$^{1-}$]) in anhydrous $CDCl_3$

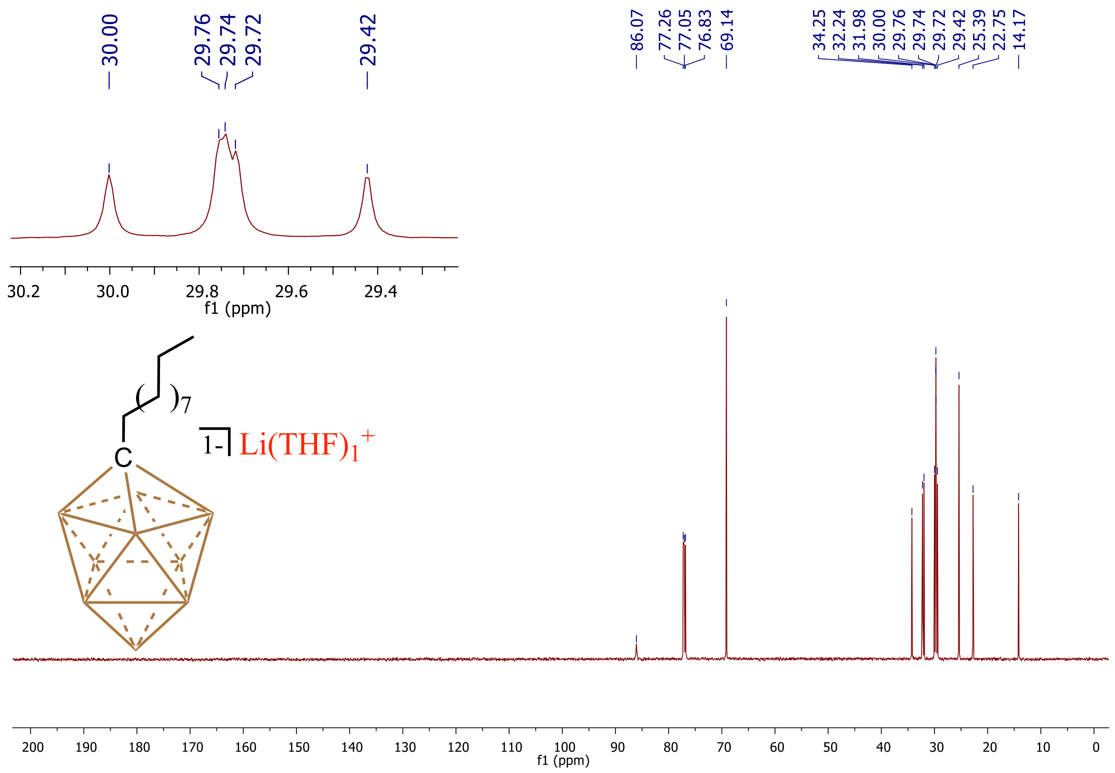

Figure S112. ^{13}C NMR spectra of **LiC$_{10}$-THF** ($[Li(THF)_1^+][C_{10}H_{21}CB_9H_9^{1-}]$) in anhydrous $CDCl_3$

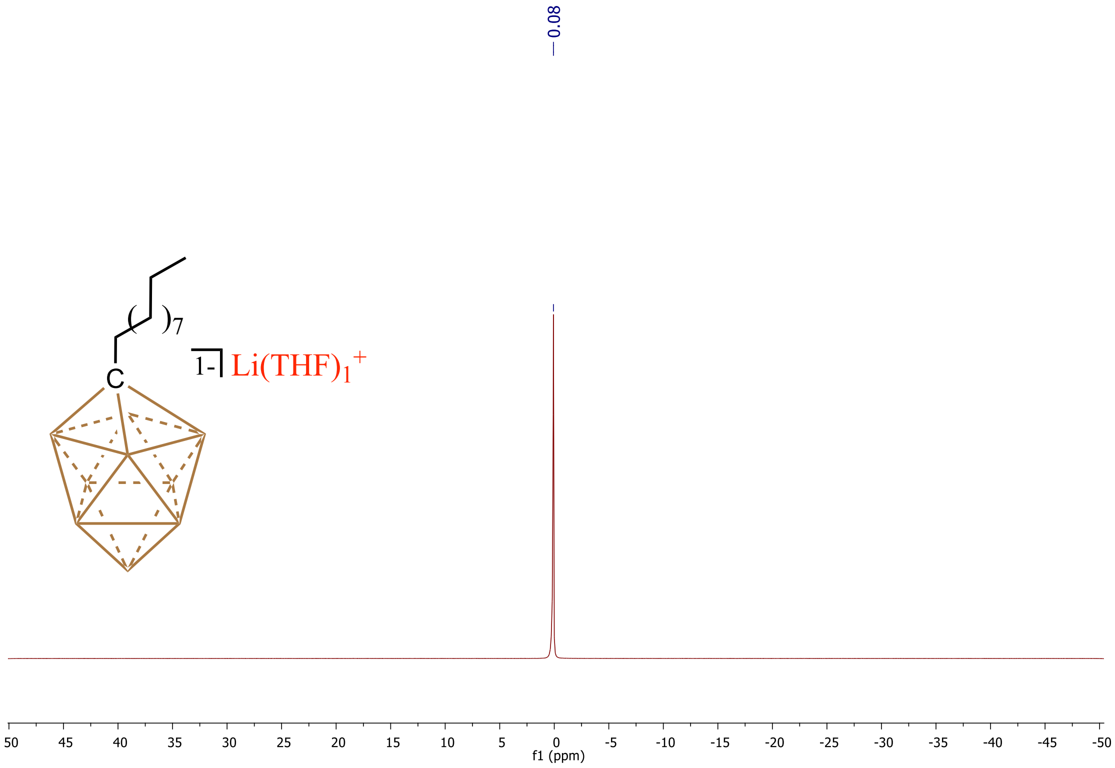

Figure S113. ^{7}Li NMR spectra of **LiC$_{10}$-THF** ($[Li(THF)_1^+][C_{10}H_{21}CB_9H_9^{1-}]$) in anhydrous $CDCl_3$

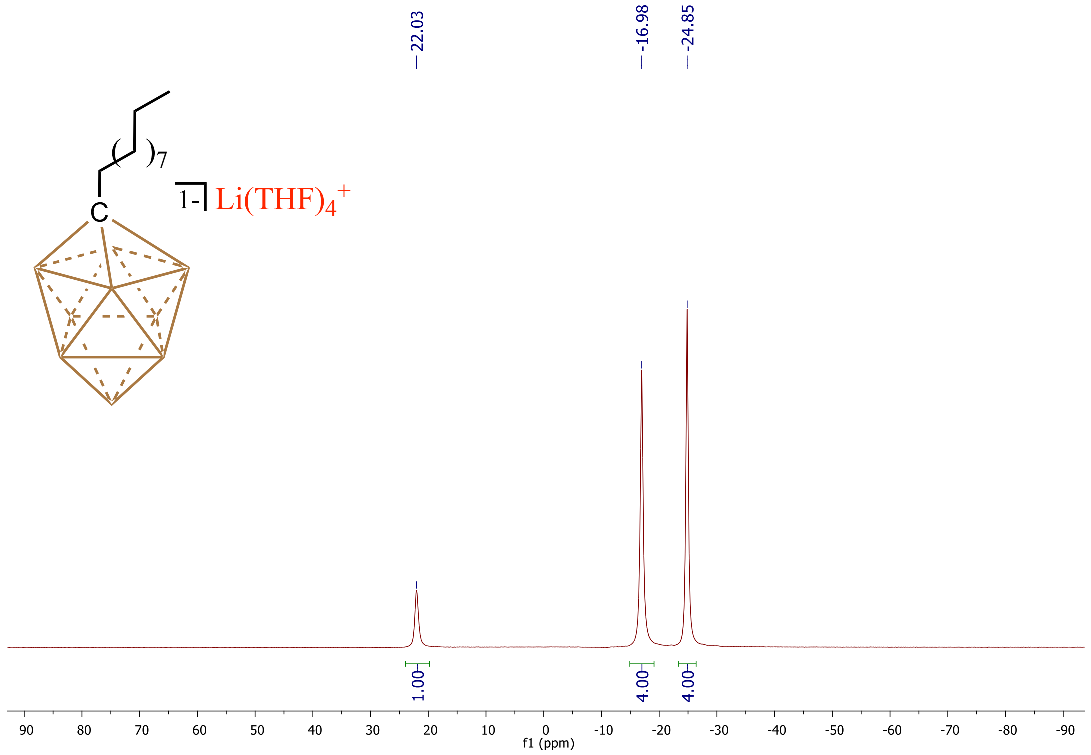

Figure S114. $^{11}B\{^{1}H\}$ NMR spectra of **LiC$_{10}$-THF$_4$** ($[Li(THF)_4^{+}][C_{10}H_{21}CB_9H_9^{1-}]$) in anhydrous $CDCl_3$

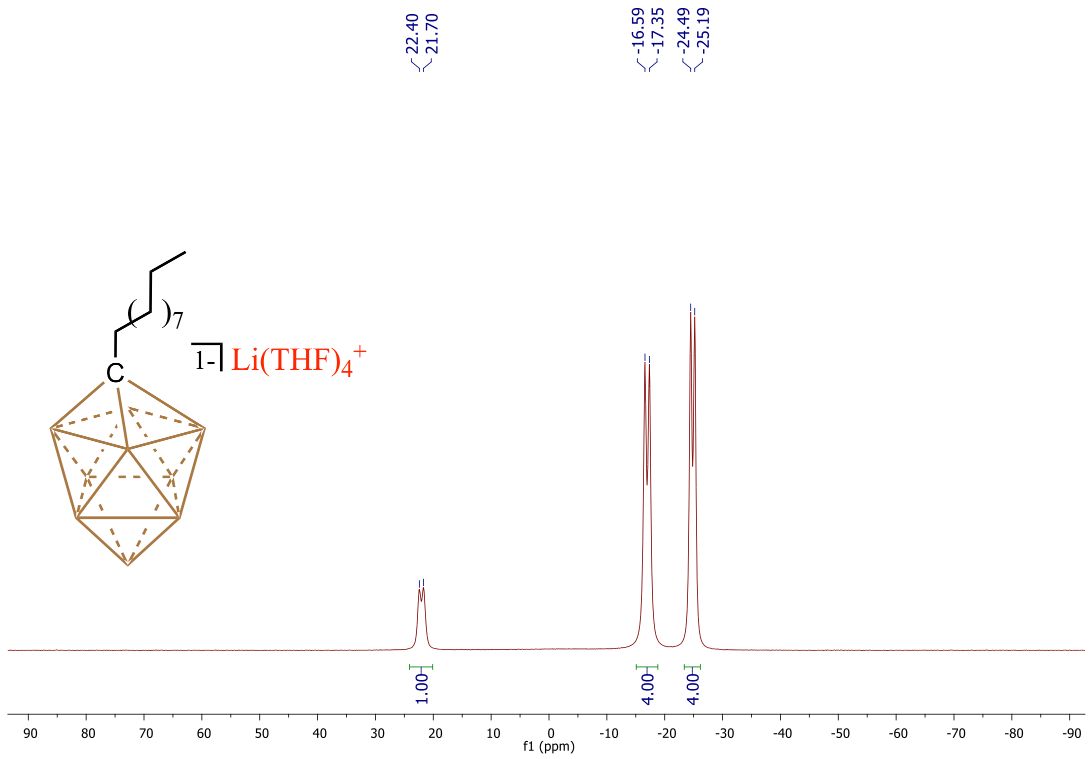

Figure S115. ^{11}B NMR spectra of **LiC$_{10}$-THF$_4$** ($[Li(THF)_4^{+}][C_{10}H_{21}CB_9H_9^{1-}]$) in anhydrous $CDCl_3$

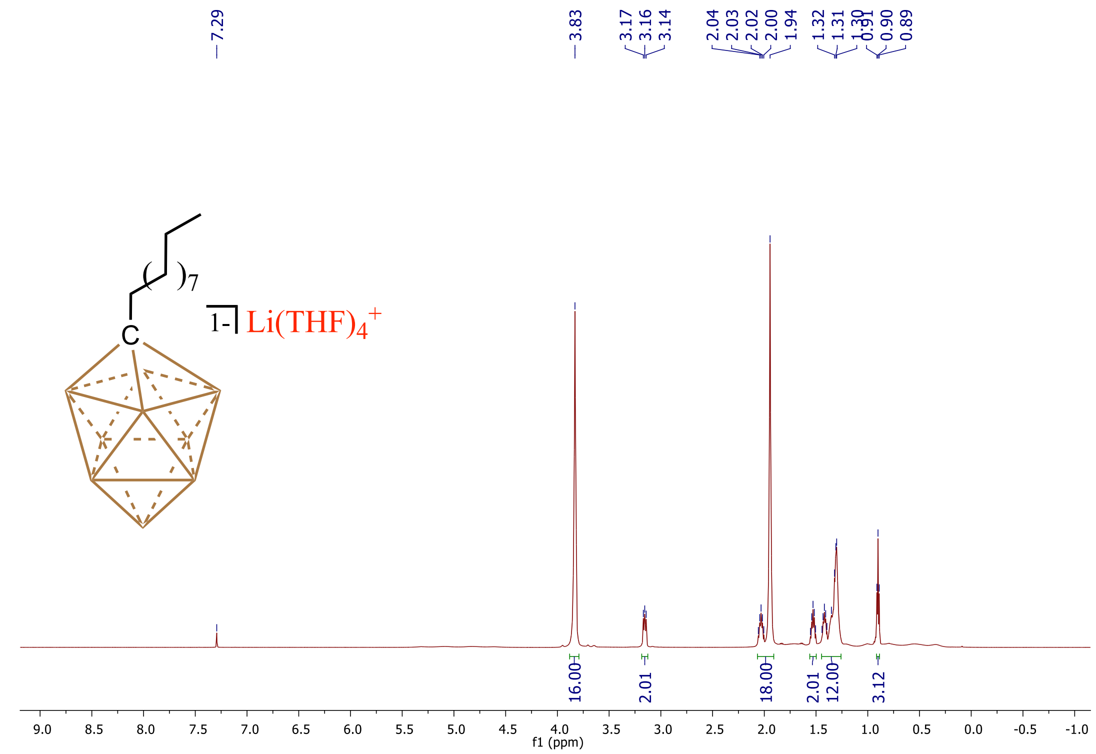

Figure S116. ^{1}H NMR spectra of **LiC₁₀-THF₄** ($[Li(THF)_4^+][C_{10}H_{21}CB_9H_9^{1-}]$) in anhydrous $CDCl_3$.

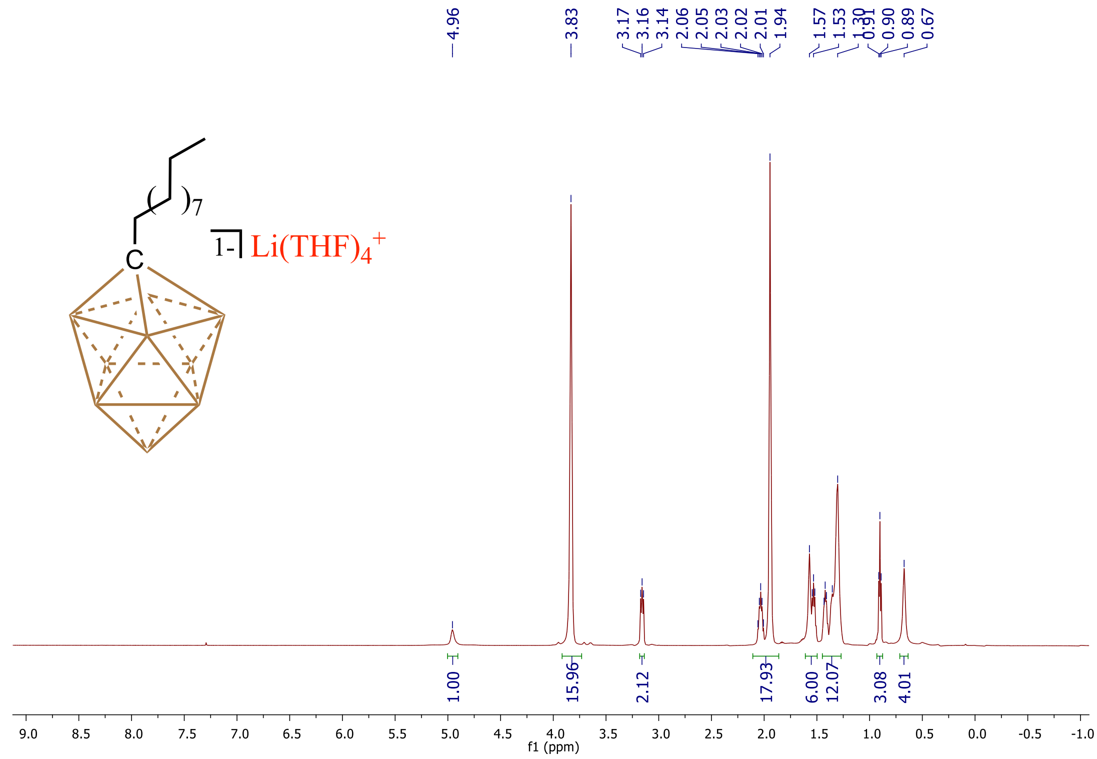

Figure S117. $^{1}H\{^{11}B\}$ NMR spectra **LiC₁₀-THF₄** ($[Li(THF)_4^+][C_{10}H_{21}CB_9H_9^{1-}]$) in anhydrous $CDCl_3$

Synthesis of [2Li(THF)$_n^+$][$B_{10}H_{10}^{2-}$] where n = 0, 2 – Figures S118 – S131

110 mg of [NH_4^+]$_2$[$B_{10}H_{10}^{2-}$] was partially dissolved in 3 mL dry THF and stirred on an excess (~1.2 mol equiv.) of LiH overnight. The reaction was pipette filtered twice through Celite and microfiber filter paper, collected into a vial, and the excess THF removed under vacuum at room temperature to yield the coordinatively saturated **[2Li(THF)$_2^+$][$B_{10}H_{10}^{2-}$]** (270 mg, 90%). Further desolvation at 100 °C for 12 hours yielded the THF-free **[2Li$^+$][$B_{10}H_{10}^{2-}$]**.

n = 0: $^{11}B\{^1H\}$ NMR (128 MHz, d$_6$-acetone) δ -0.50 (s, 2 B-H), -28.27 (s, 8 B-H). ^{11}B NMR (128 MHz, d$_6$-acetone) δ -0.51 (d, *J* = 139.0 Hz, 2 B-H), -28.28 (d, *J* = 123.8 Hz, 8 B-H). ^1H NMR (400 MHz, d$_6$-acetone) δ 3.67 (d, 1 B-H), 2.95 (d, *J* = 138.3 Hz, 1 B-H), 0.21 (dd, *J* = 253.4, 122.3 Hz, 8H). ^7Li NMR (156 MHz, d$_6$-acetone) δ 1.51.

n = 2: $^{11}B\{^1H\}$ NMR (128 MHz, d$_6$-acetone) δ 0.06 (s, 2 B-H), -27.71 (s, 8 B-H). ^{11}B NMR (128 MHz, d$_6$-acetone) δ 0.54 (d, *J* = 137.0 Hz, 2 B-H), -27.71 (d, *J* = 122.9 Hz, 8 B-H). ^1H NMR (400 MHz, d$_6$-acetone) δ 3.82-3.47 (d, *J*=140 Hz, 1 B-H), 3.62 (m, 16H), 3.13-2.78 (d, *J*=140 Hz, 1 B-H), 1.79 (m, 16H), 0.69 - -0.26 (dd, *J* = 253.4, 122.3 Hz, 8H). ^7Li NMR (156 MHz, d$_6$-acetone) δ 1.47.

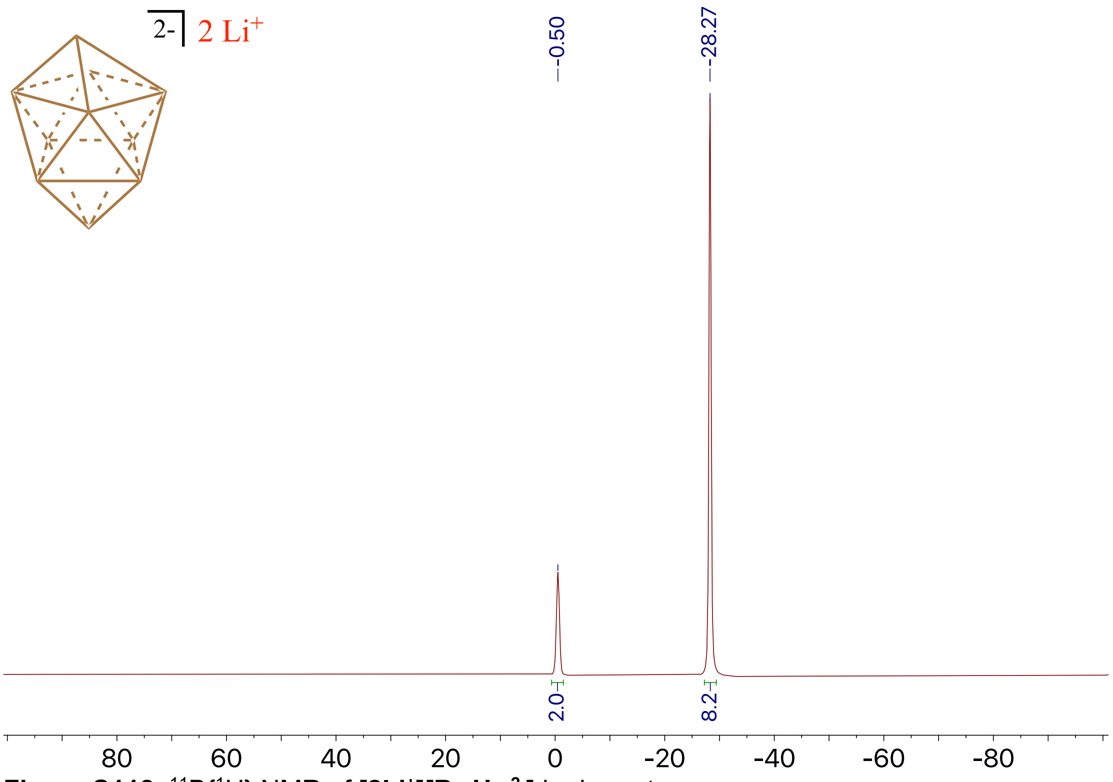

Figure S118. $^{11}B\{^1H\}$ NMR of **[2Li$^+$][$B_{10}H_{10}^{2-}$]** in d$_6$-acetone.

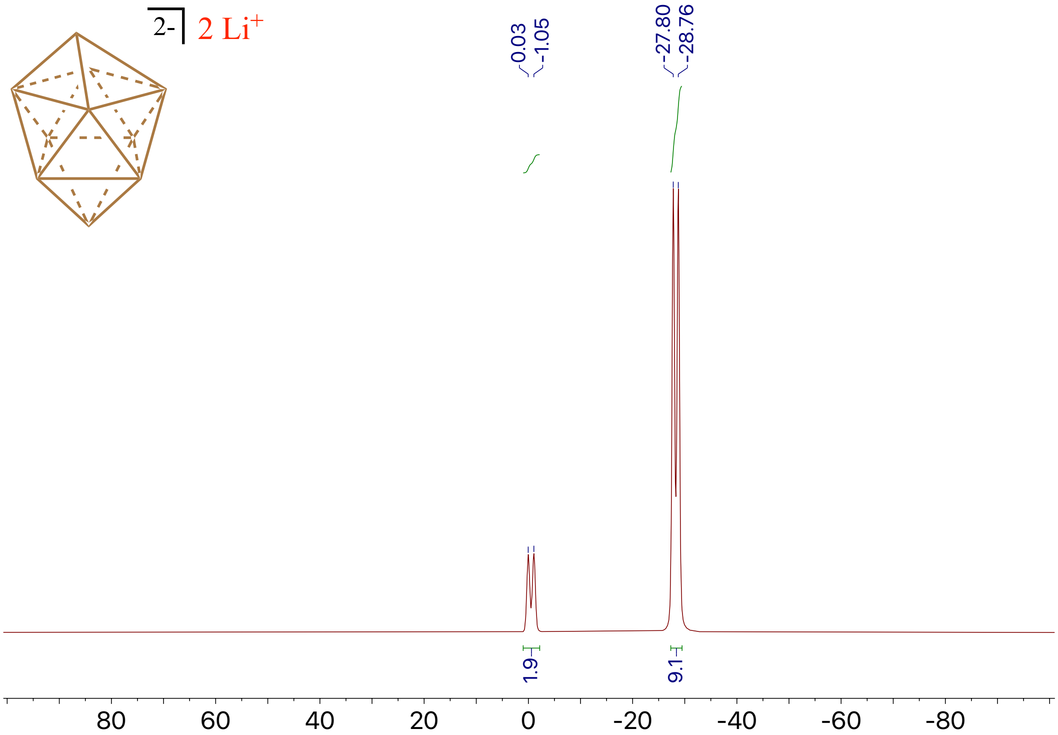

Figure S119. ^{11}B NMR of **[2Li^{+}][$B_{10}H_{10}^{2-}$]** in d_6-acetone.

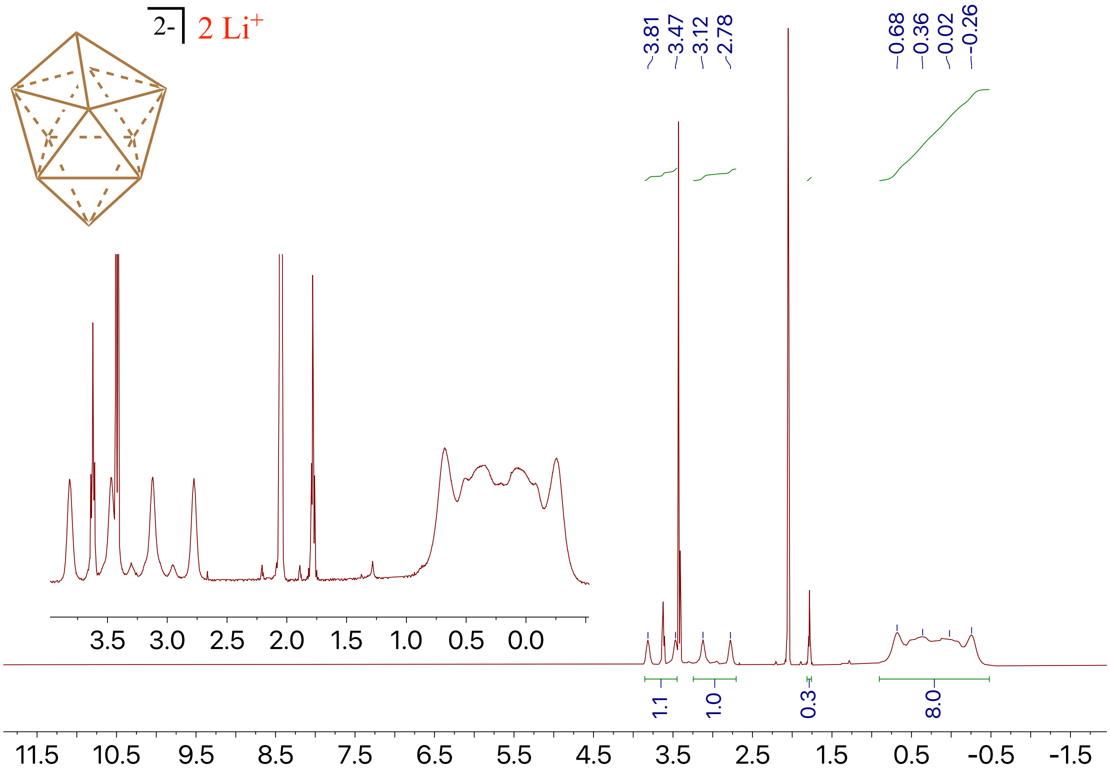

Figure S120. ^{1}H NMR of **[2Li^{+}][$B_{10}H_{10}^{2-}$]** in d_6-acetone. Note: water peaks arise at 3.4 ppm. There is roughly 7.5 THF molecules per 100 **[2Li^{+}][$B_{10}H_{10}^{2-}$]**.

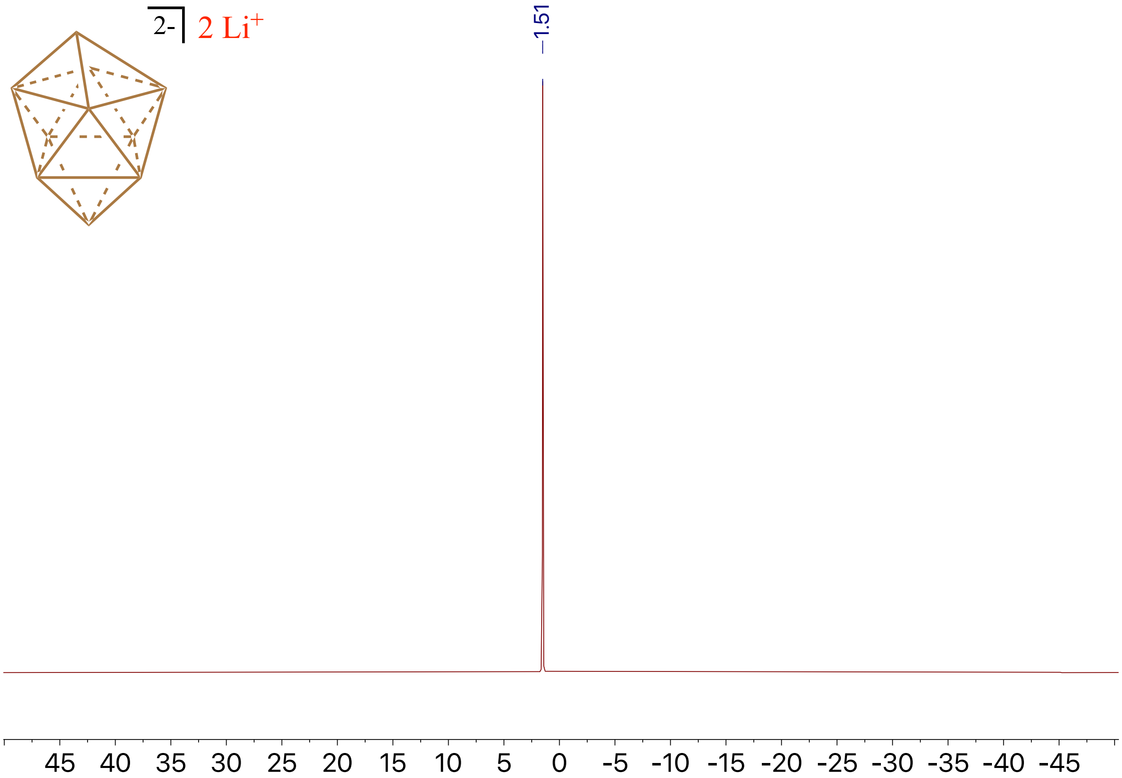

Figure S121. ^{7}Li NMR **[2Li^{+}][B$_{10}$H$_{10}$$^{2-}$]** in d$_6$-acetone.

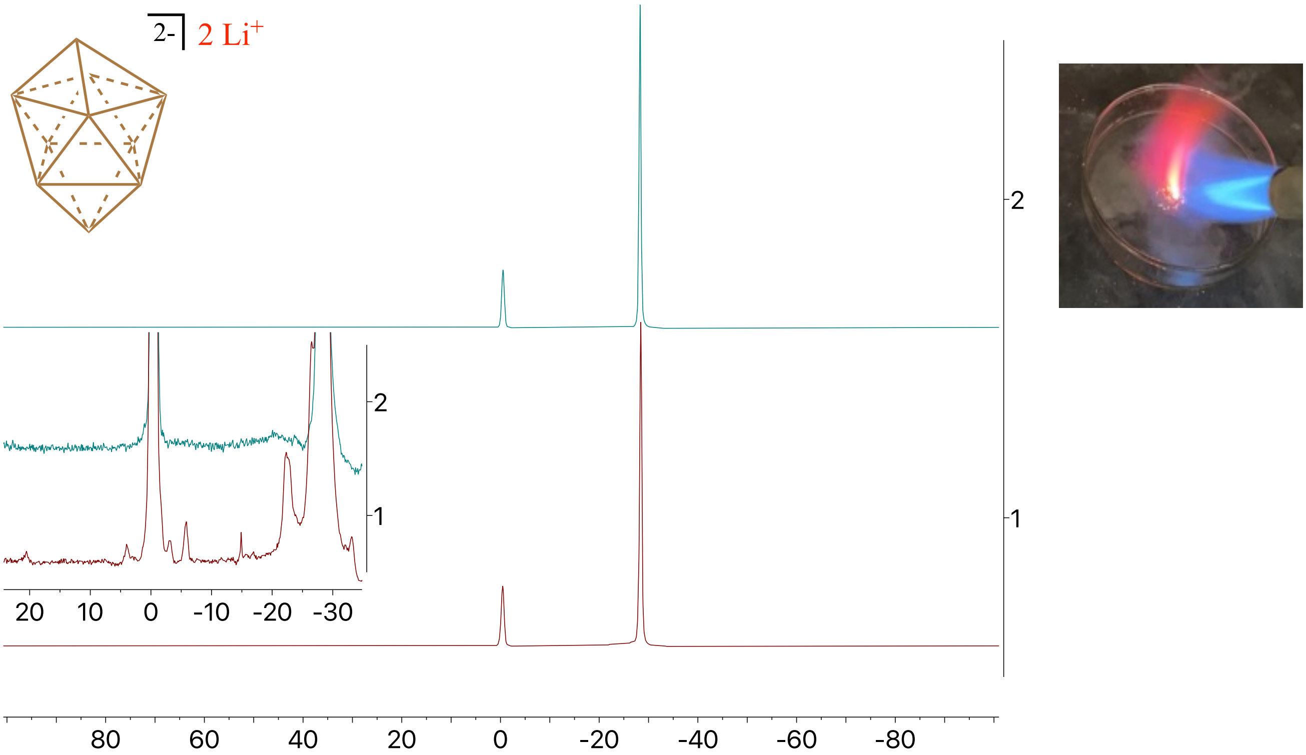

Figure S122. ^{11}B{^{1}H} NMR of **[2Li^{+}][B$_{10}$H$_{10}$$^{2-}$]** before (above) and after (below) burning 3s in d$_6$-acetone. Minor decomposition into boric acid observed at 20 ppm.

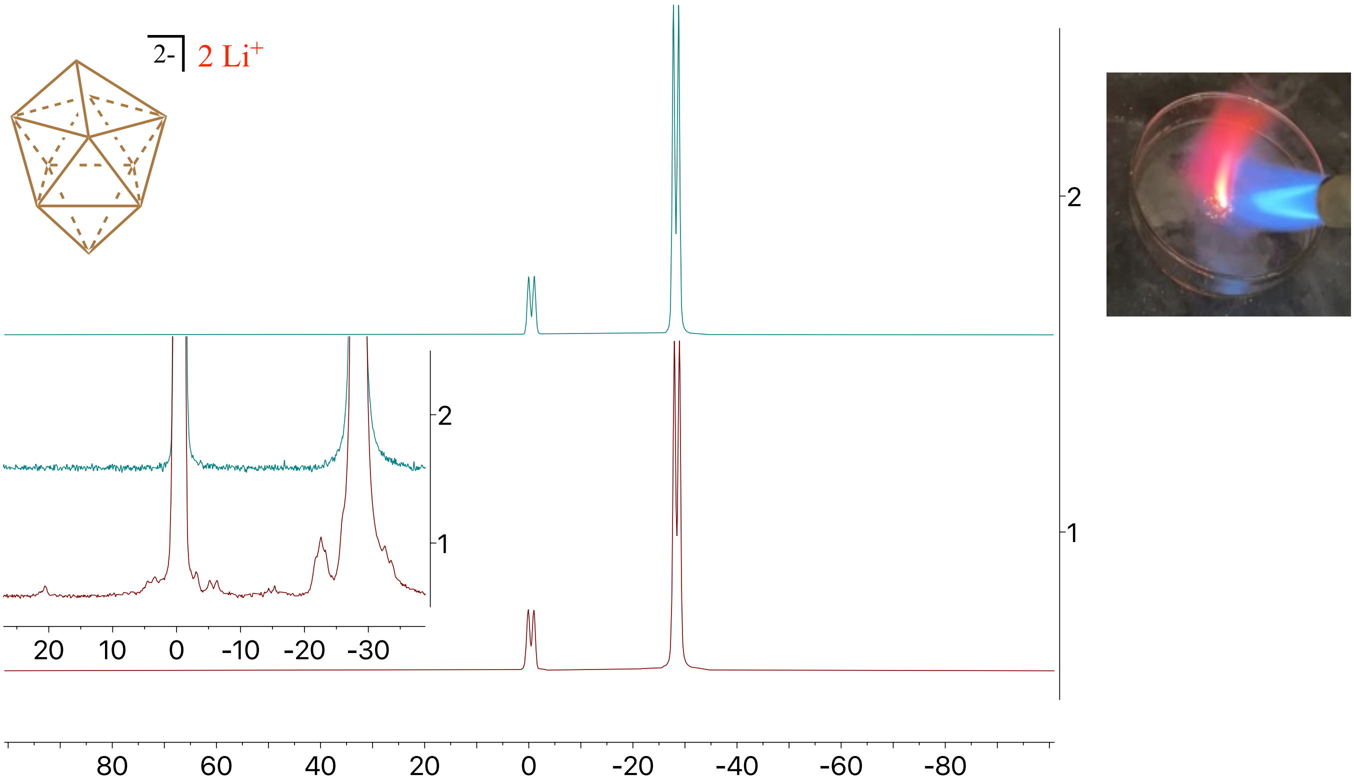

Figure S123. ^{11}B NMR of **[2Li$^+$][B$_{10}$H$_{10}$$^{2-}$]** before (above) and after (below) burning 3s in d$_6$-acetone. Minor decomposition into boric acid observed at 20 ppm.

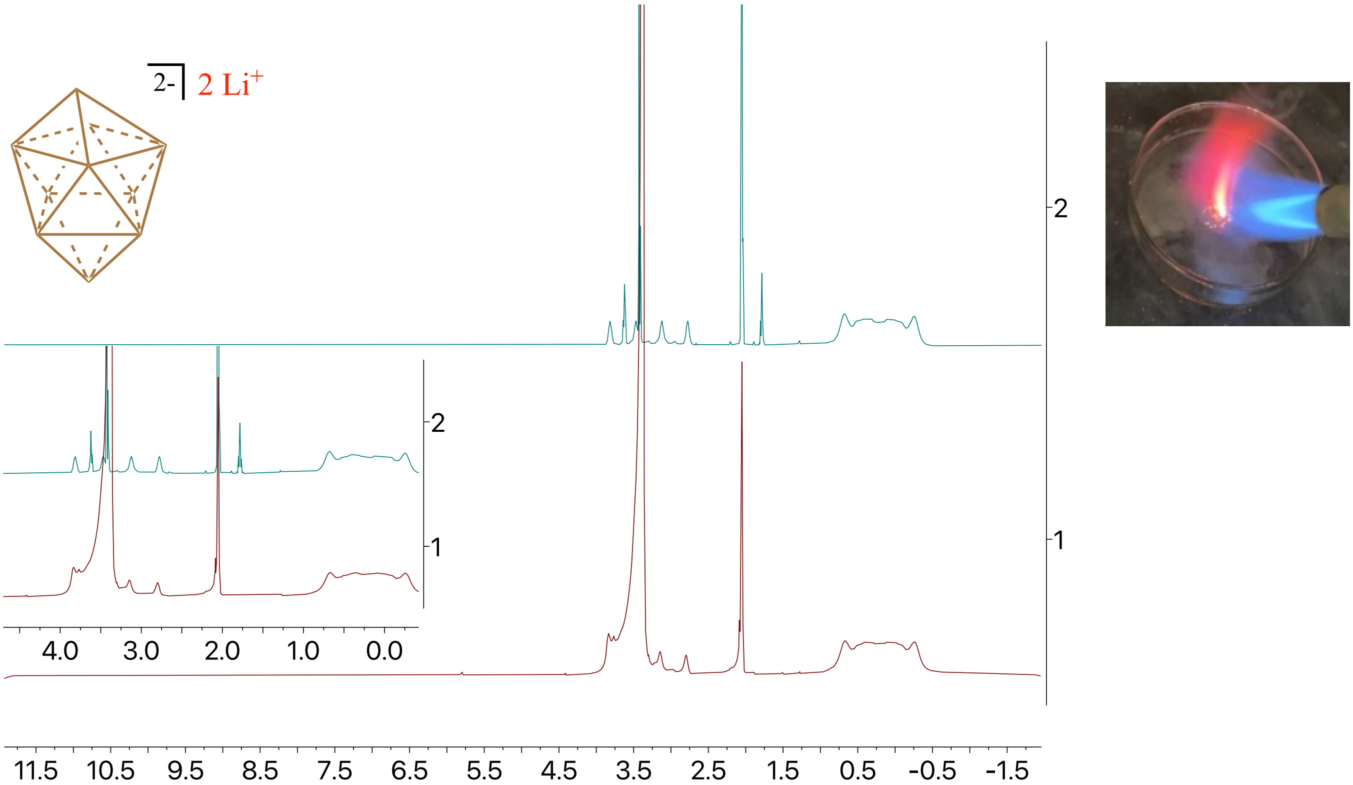

Figure S124. ^{1}H NMR of **[2Li$^+$][B$_{10}$H$_{10}$$^{2-}$]** before (above) and after (below) burning 3s in d$_6$-acetone. Note: water peaks arise at 3.4 ppm.

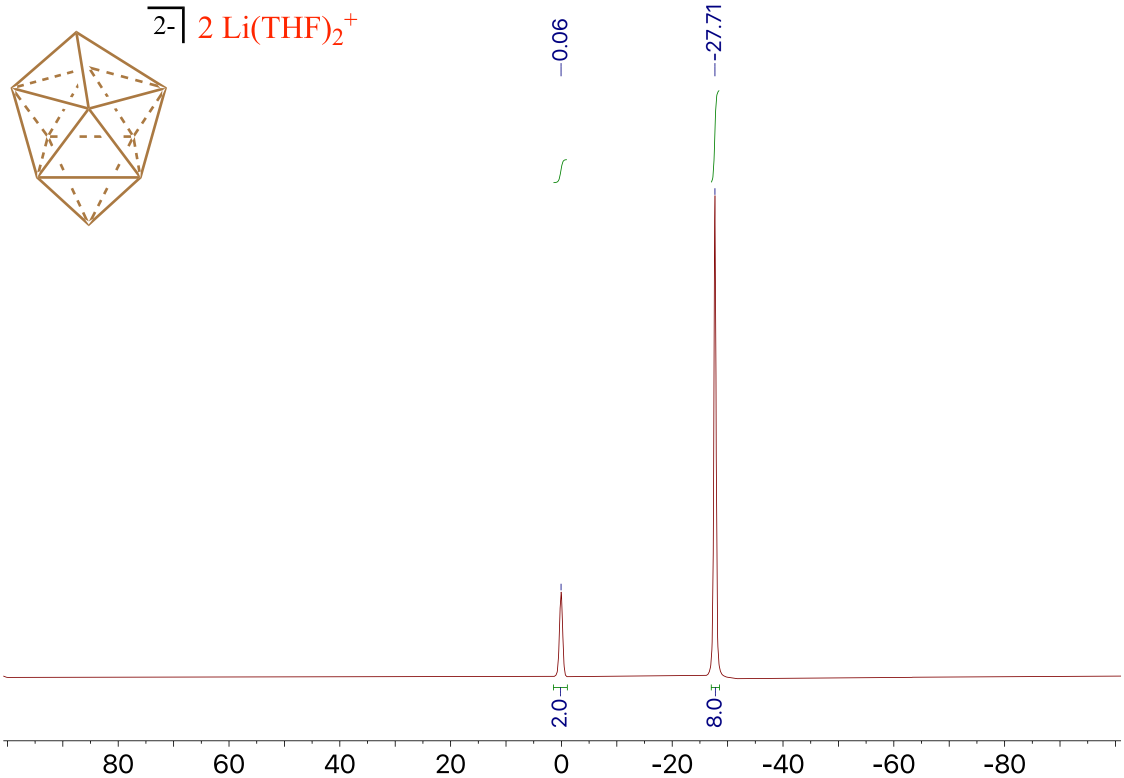

Figure S125. $^{11}B\{^1H\}$ NMR of **[2Li(THF)$_2^+$][B$_{10}$H$_{10}^{2-}$]** in d$_6$-acetone.

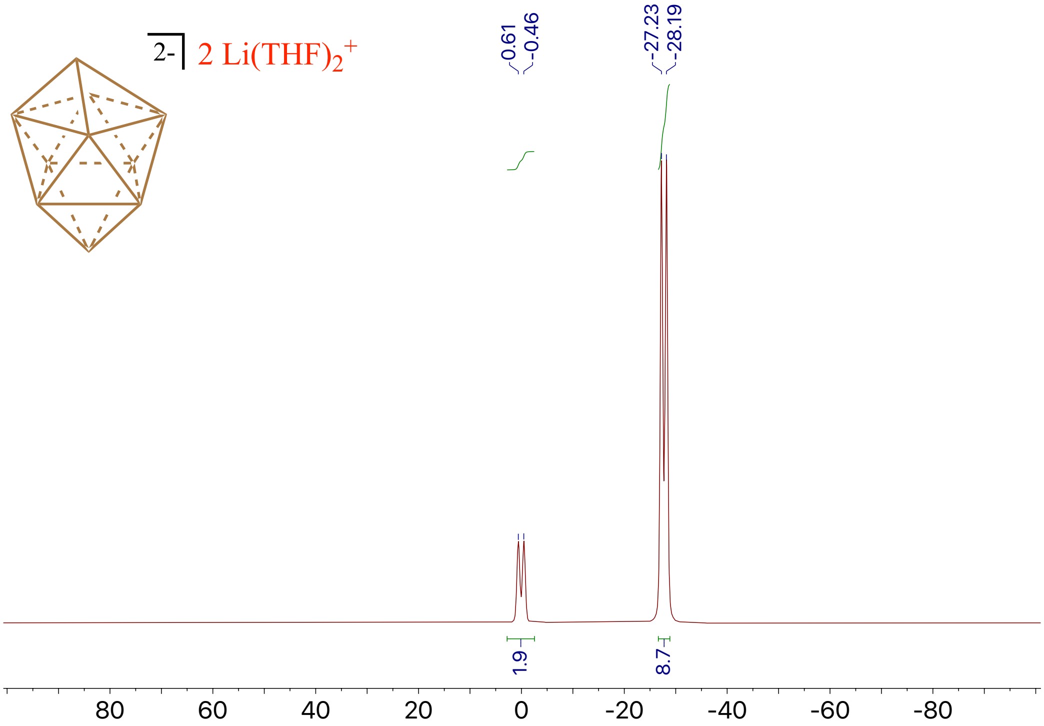

Figure S126. ^{11}B NMR of **[2Li(THF)$_2^+$][B$_{10}$H$_{10}^{2-}$]** in d$_6$-acetone.

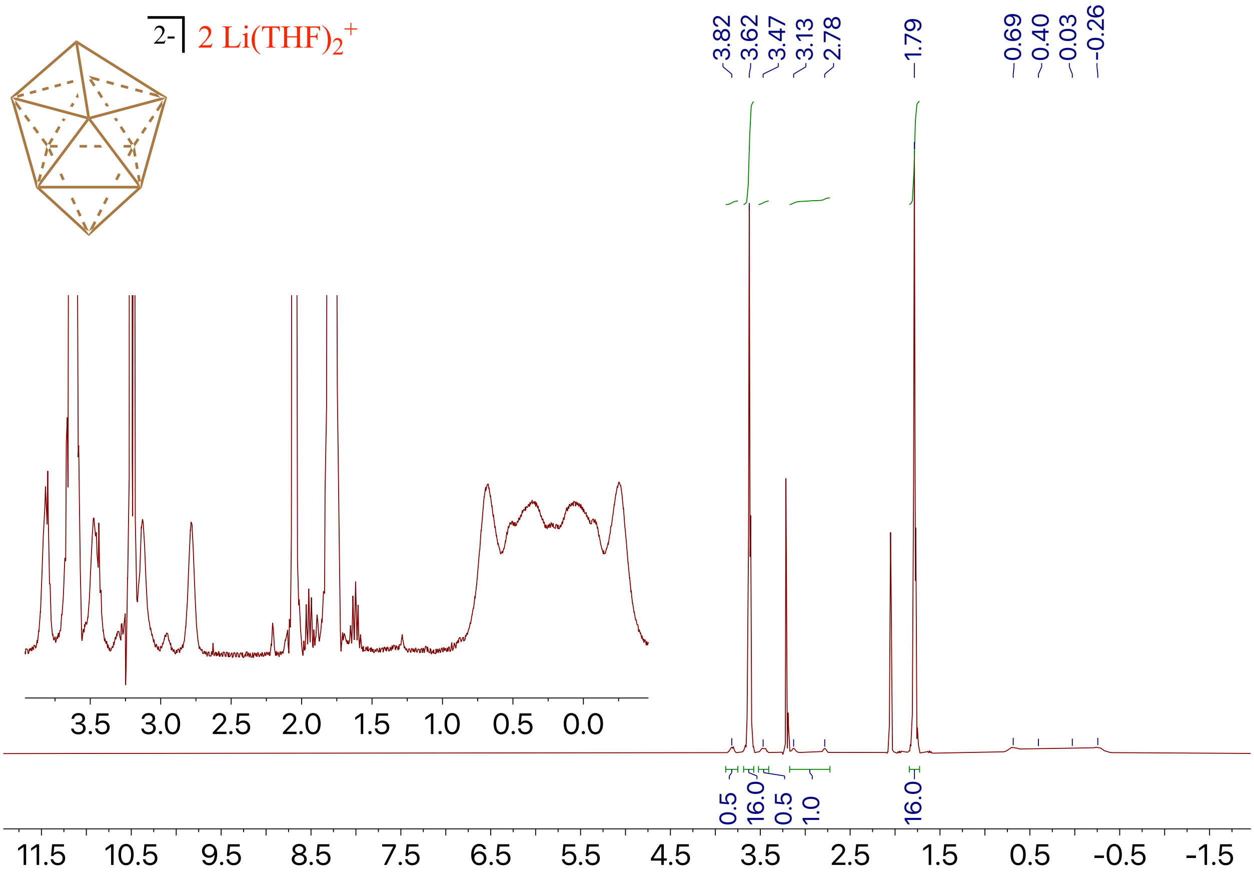

Figure S127. ^{1}H NMR of **[2Li(THF)$_2$$^{+}$][B$_{10}H_{10}$$^{2-}$]** in d$_6$-acetone. Note: water peaks arise at 2.8 ppm.

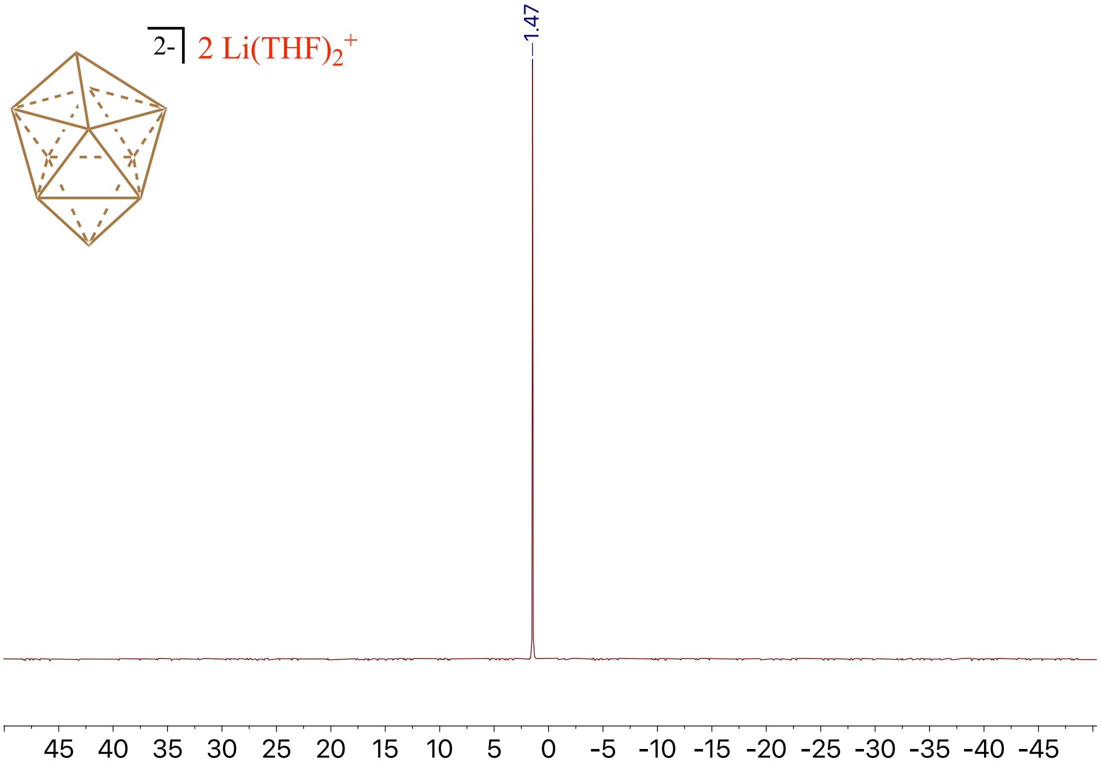

Figure S128. ^{7}Li NMR of **[2Li(THF)$_2$$^{+}$][B$_{10}H_{10}$$^{2-}$]** in d$_6$-acetone.

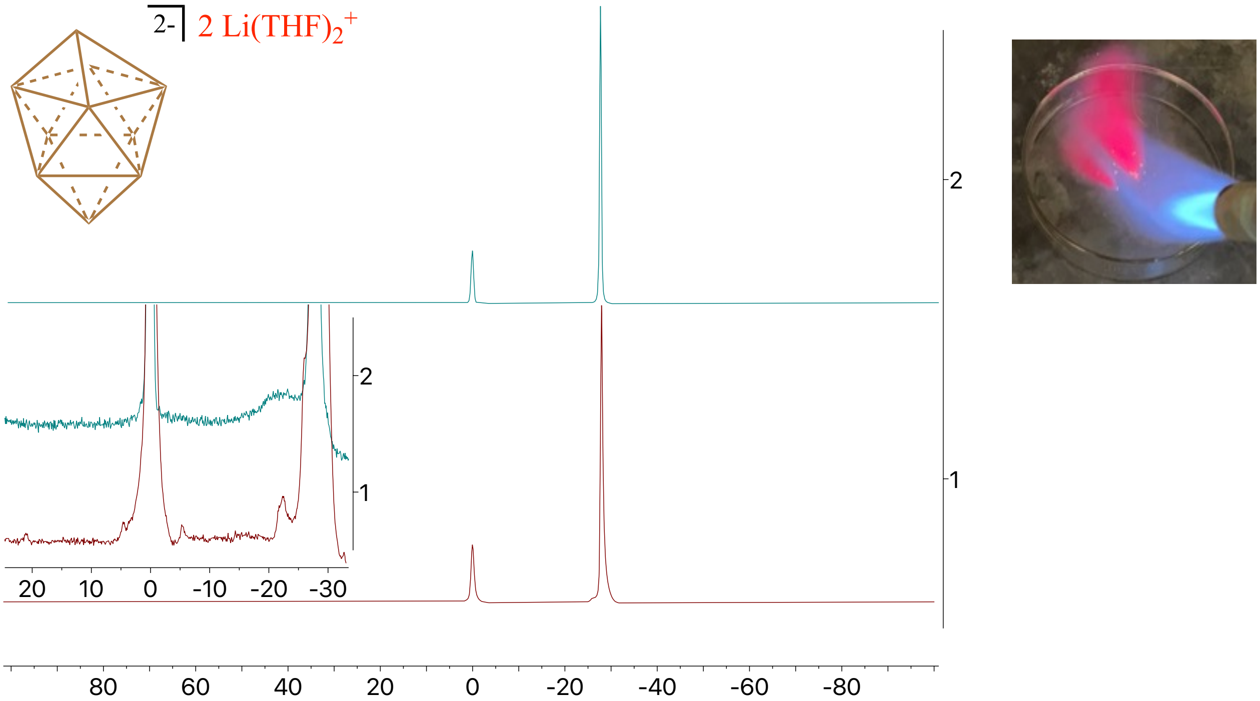

Figure S129. $^{11}B\{^1H\}$ NMR of **[2Li(THF)$_2^+$][B$_{10}$H$_{10}^{2-}$]** before (above) and after (below) burning 3s in d$_6$-acetone.

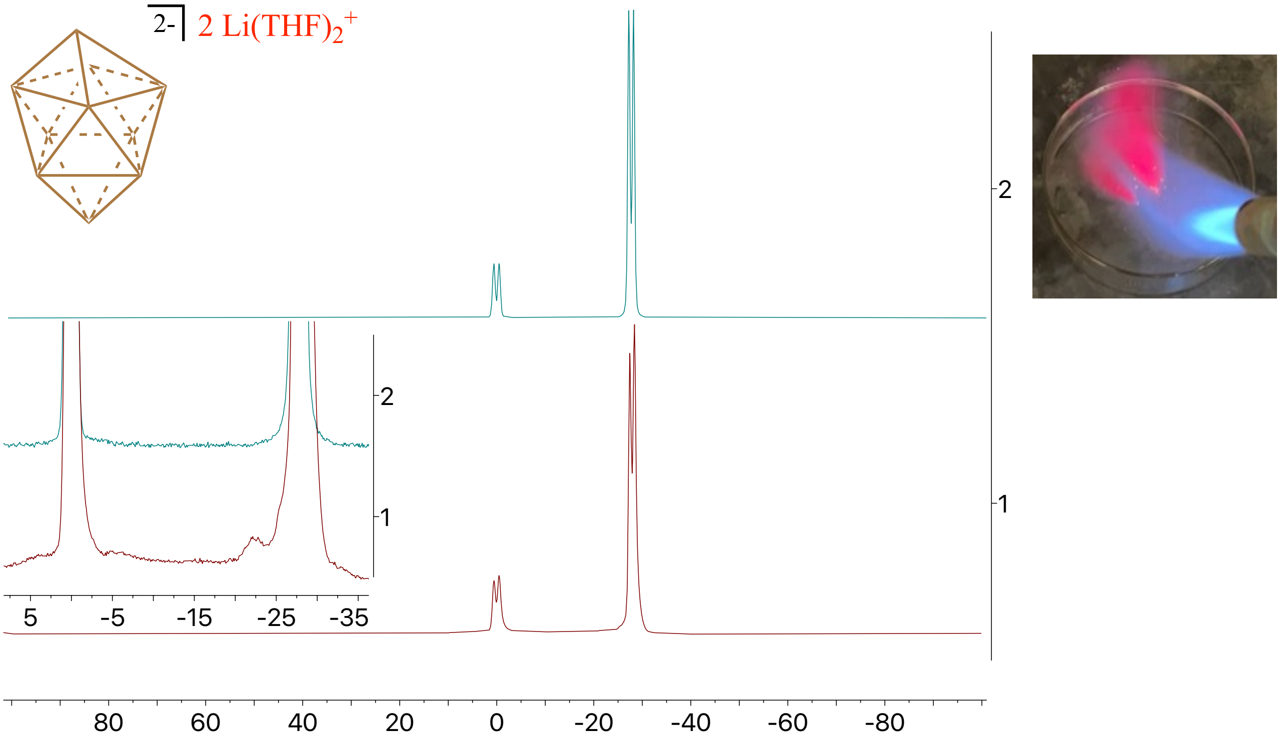

Figure S130. ^{11}B NMR of **[2Li(THF)$_2^+$][B$_{10}$H$_{10}^{2-}$]** before (above) and after (below) burning 3s in d$_6$-acetone.

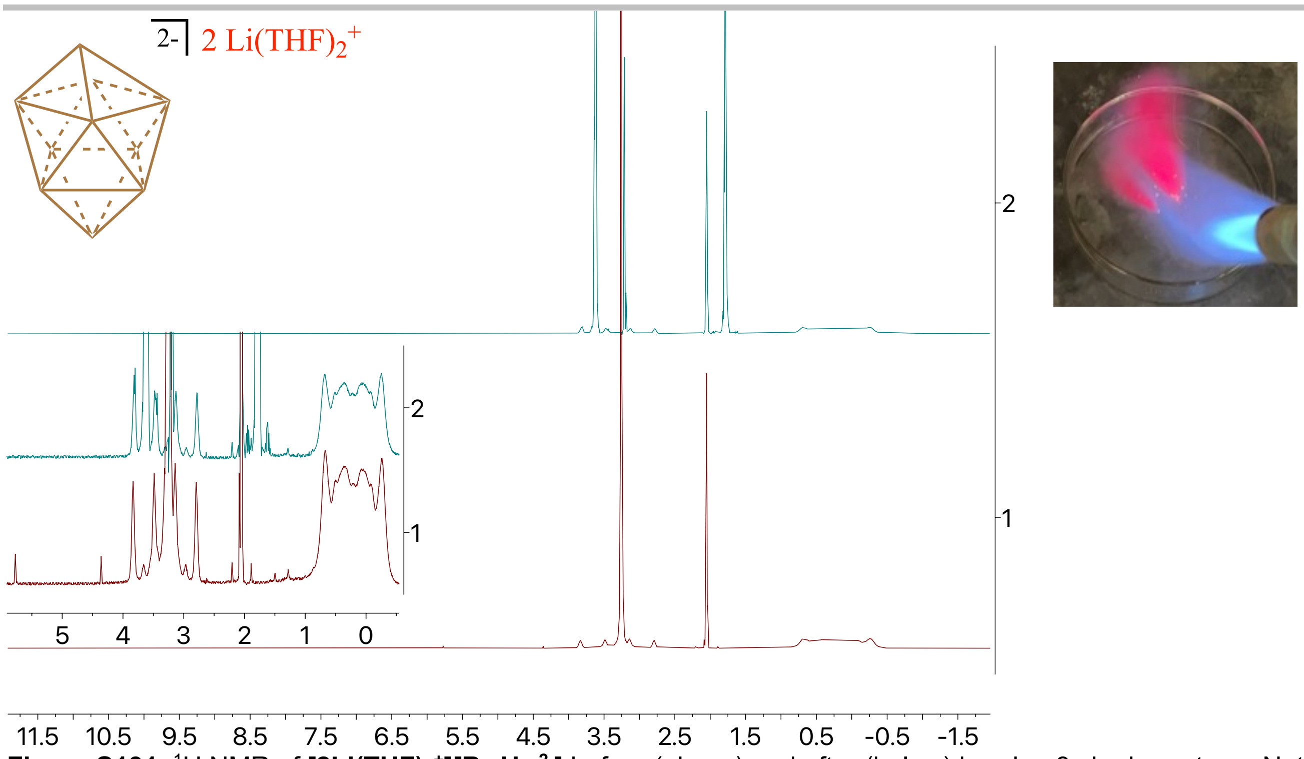

Figure S131. ^{1}H NMR of **[2Li(THF)$_2^+$][B$_{10}$H$_{10}^{2-}$]** before (above) and after (below) burning 3s in d_6-acetone. Note: water peaks arise at 2.8 (above) and 3.1 ppm (below).

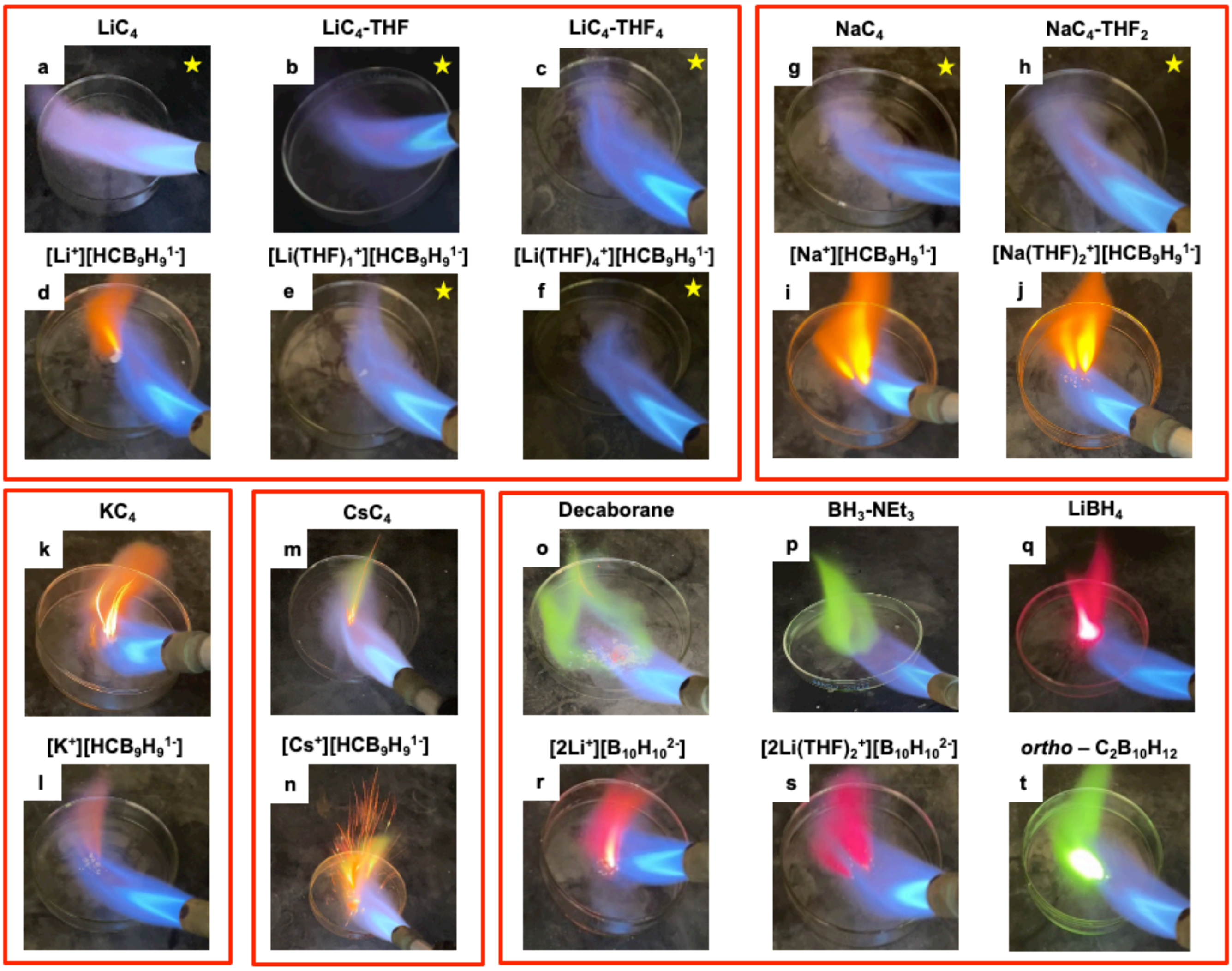

Figure S132. Combustion behavior of C_4 species, alkali metal analogues, and borohydrides. (a-c) combustion resistant LiC_4, LiC_4-THF, and LiC_4-THF_4. (d-f) Non-alkylated parent derivatives $[Li(THF)_n^+][HCB_9H_9^{1-}]$ where n= 0, 1, 4. (g-n) alkali metal cation analogues of $\mathbf{C_4}$ and $[HCB_9H_9^{1-}]$. (o-t) open cluster borohydride decaborane ($B_{10}H_{14}$), adduct-forming liquid borohydride BH_3NEt_3, ionic salt borohydride $LiBH_4$, *closo*- 10-vertex derivative without carbon $B_{10}H_{10}^{2-}$, charge neutral carborane *Ortho*-$C_2B_{10}H_{12}$. Note: Star indicates lack of combustion behavior.

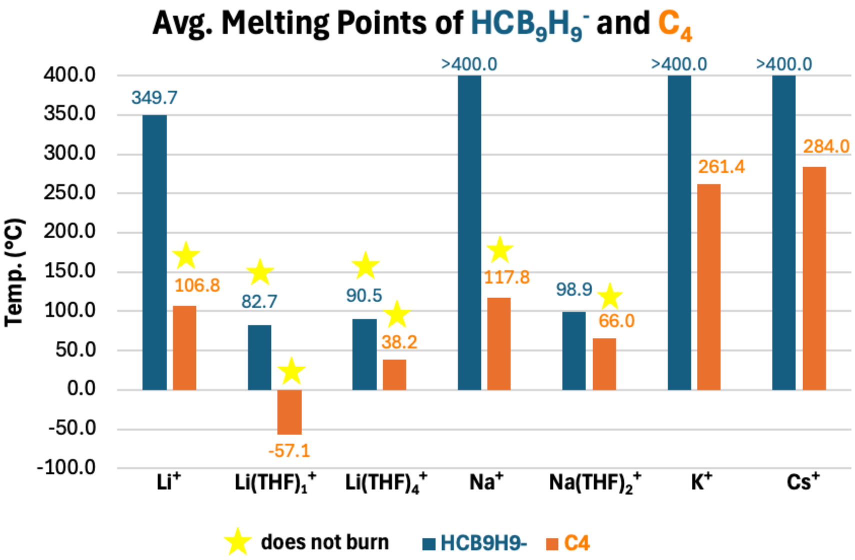

Figure S133. Average melting points of alkali metal cations paired with $[HCB_9H_9^{1-}]$ and $\mathbf{C_4}$ ($[C_4H_9CB_9H_9^{1-}]$).

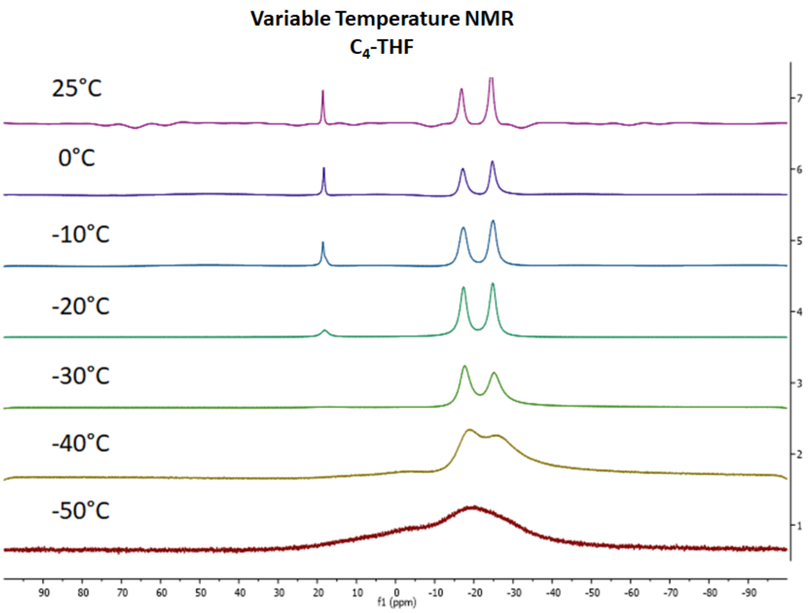

Figure S134. ^{11}B{^{1}H} VT NMR spectra of neat **LiC4-THF** descending from 25°C to -50°C. Near total coalescence of [11]B signals at -50°C corroborates DSC data of **LiC4-THF** which reveals a reversible isotherm at -57°C corresponding to the compound's melting point.

Differential Scanning Calorimetry

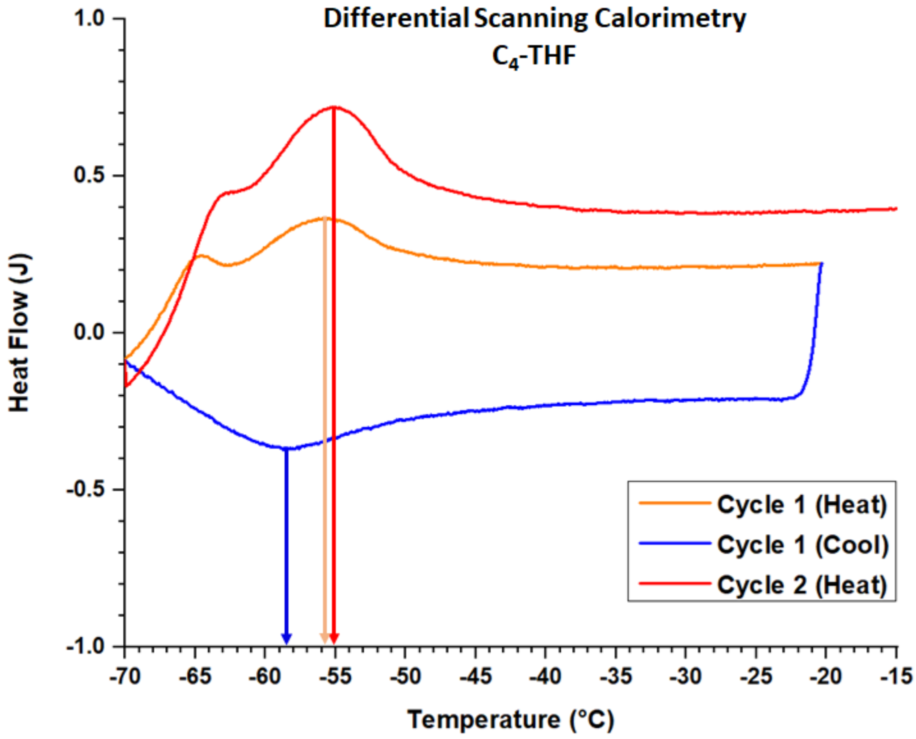

Figure S135. Differential Scanning Calorimetry of neat **LiC4-THF**. Heating cycle 1 was preceded by isothermic cooling at -70°C for 60 minutes followed by a heating cycle operated at a ramp rate of 3K/min. Subsequent cooling and heating cycles were performed to verify the presence of a persistent isotherm indicating the solid/liquid phase transition of **LiC4-THF**.

Mass Spectrometry

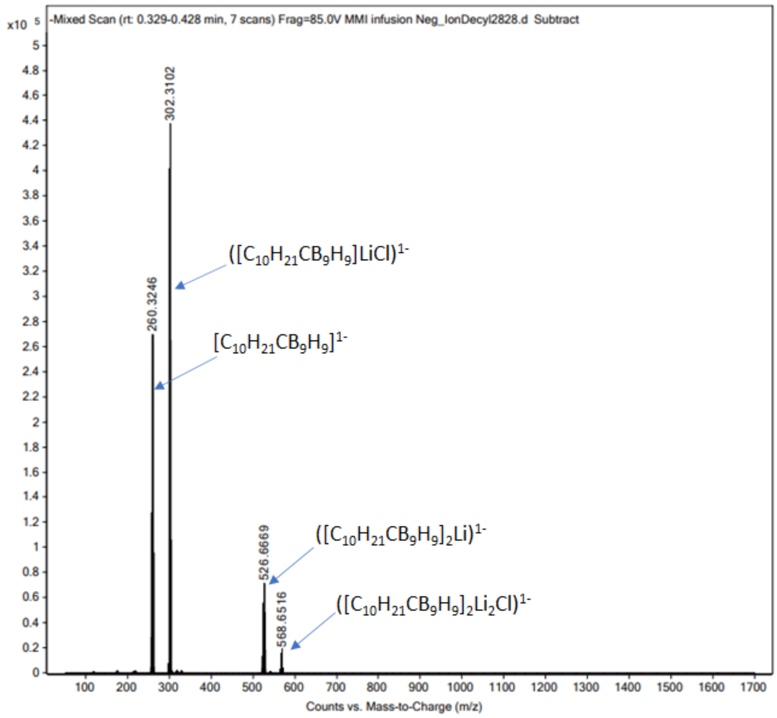

Figure S136. Negative ion mode High Resolution Mass Spectrometry. Calculated: {$C_{10}H_{21}CB_9H_9{}^{1-}$} = 260.3221 m/z; {$C_{10}H_{21}CB_9H_9{}^{1-}$} = 260.3246 m/z.

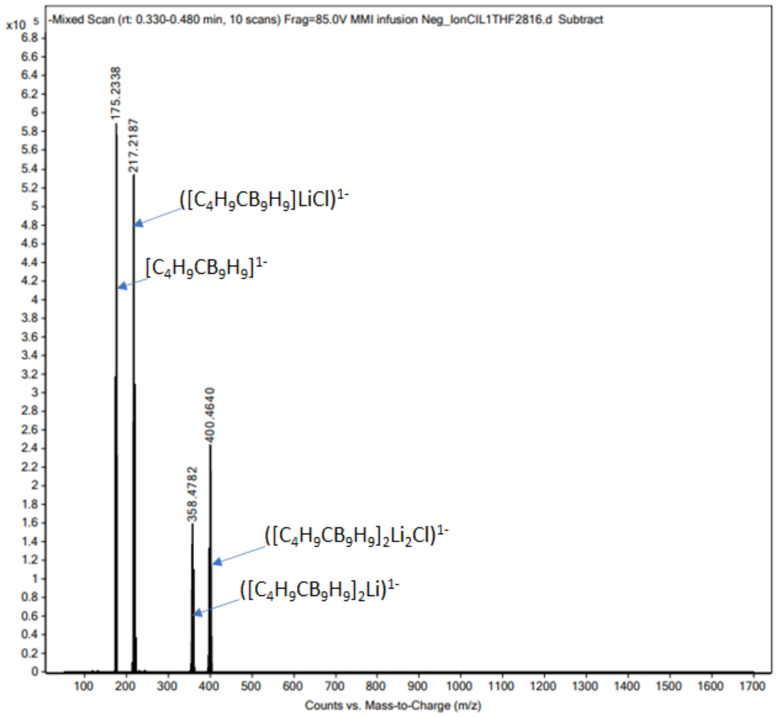

Figure S137. Negative ion mode High Resolution Mass Spectrometry. Calculated: {$C_4H_9CB_9H_9{}^{1-}$} = 175.2318 m/z; Found: {$C_4H_9CB_9H_9{}^{1-}$} = 175.2338 m/z

Electrolyte Images

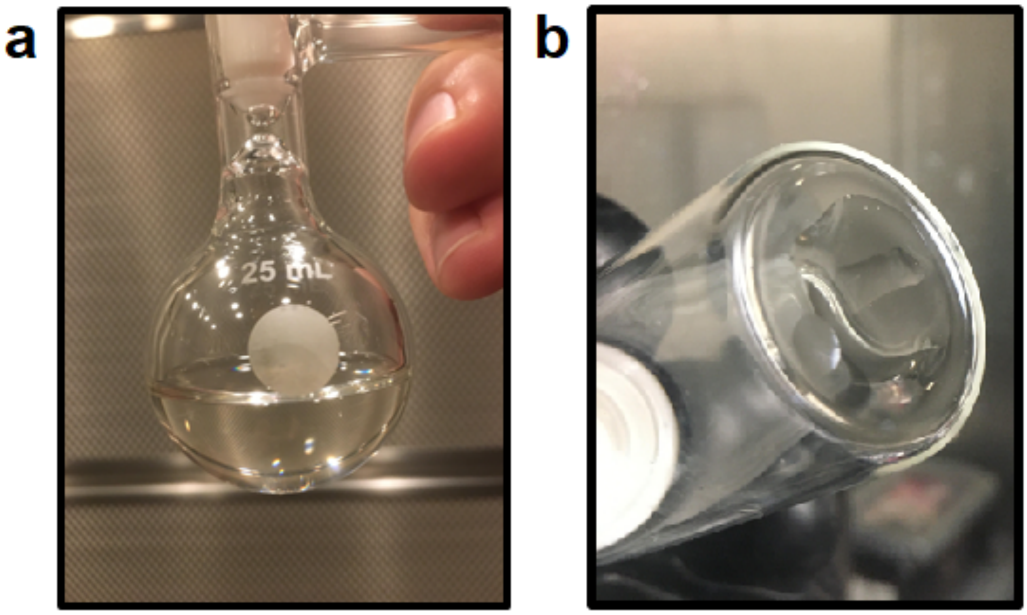

Figure S138. (a) 10 mL of purified **LiC$_4$-THF** suitable for electrochemical characterization (b) image of **LiC$_4$-THF** taken immediately after rest for 60 days in -30°C freezer displays no sign of crystallization.

Computational Methods

DFT calculations

To predict the $[Li(THF)_1^+][C_4H_9CB_9H_9^{1-}]$ **(LiC$_4$-THF)** structure, we performed density functional theory (DFT) calculations, based on the crystal structure of $[Li(THF)_1^+][C_{10}H_{21}CB_9H_9^{1-}]$ **(LiC$_{10}$-THF)** by modifying the carbon chain from decyl ($C_{10}H_{21}$-) to butyl (C_4H_9-) (Fig. S139). All DFT calculations were performed using Vienna *Ab initio* Simulation Package (VASP).[3] The projector-augmented-wave (PAW) potentials were applied to describe the valence electrons[4], and the Perdew, Burke, and Ernzerhof (PBE) flavor of the generalized gradient approximation (GGA) functional was applied to describe the exchange-correlation potentials.[5] We included the D3 correction (PBE-D3) for van der Waals attraction.[6] The Brillouin zone was sampled using the 2 × 2 × 1 Monkhorst-Pack grid. The cutoff energy was set to 500 eV, and the convergence criteria for the electronic structure and the atomic geometry were 10^{-5} eV and 0.01 eV/Å, respectively.

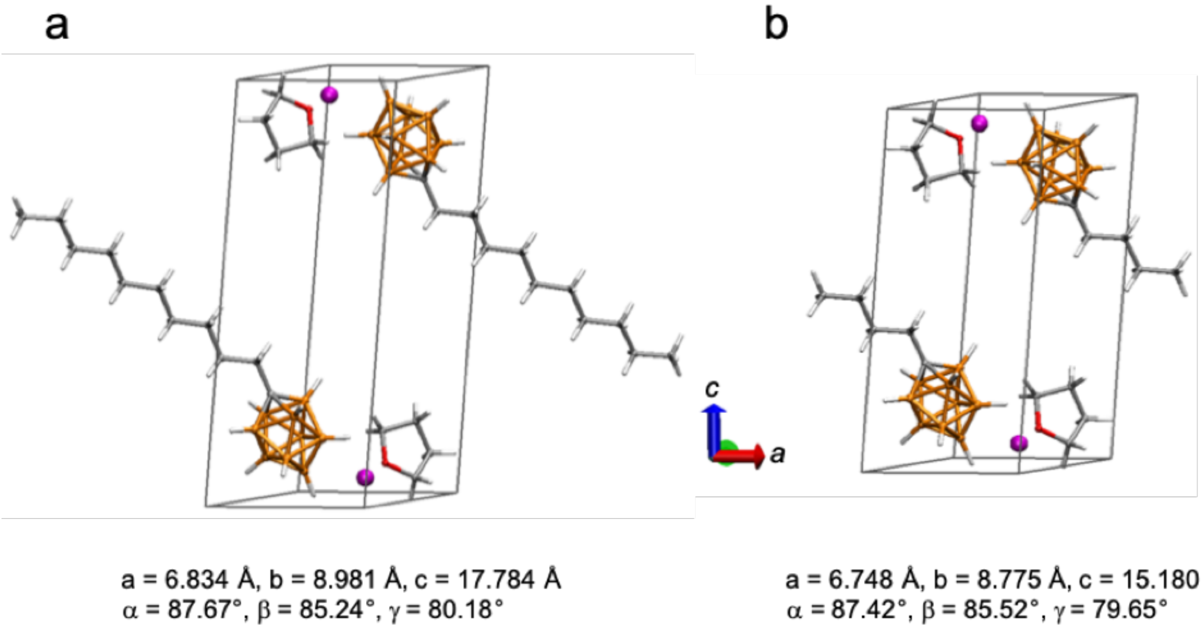

Figure S139. (a) The experimentally determined crystal structure of **LiC$_{10}$-THF** ($[Li(THF)_1^+][C_{10}H_{21}CB_9H_9^{1-}]$) and (b) the predicted structure of **LiC$_4$-THF** ($[Li(THF)_1^+][C_4H_9CB_9H_9^{1-}]$) at the DFT-D3 level.

Single Crystal Images - vl382at (LiC_{10}-THF)

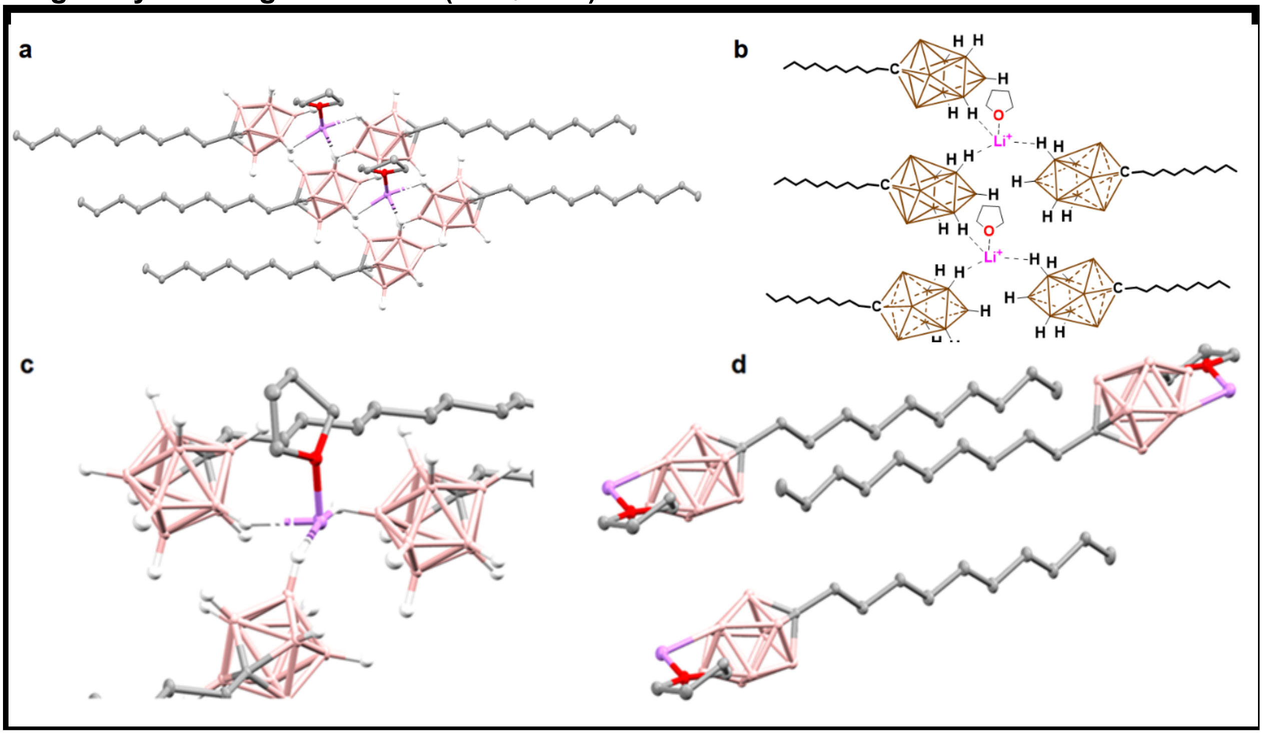

Figure S140. (a) Single crystal characterization of **LiC_{10}-THF** analogue next to (b) drawn representation of structure displaying B-H-Li interactions for clarity. (c) distorted tetrahedral coordination environment around Li^+ occupied by 3 carboranyl B-H and 1 THF molecule (d) VDWs contributions to crystal packing arrangement.

Crystal Data and Structure Refinement: vl382at (LiC_{10}-THF)

Diffraction data were collected on a Bruker D8 Venture Duo diffractometer with a Bruker Photon III CPAD detector using Mo K_α radiation (λ = 0.71073 Å) from an IµS micro-source. Data were collected at 100 K by performing 0.5° ϕ- and ω-scans, integrated using SAINT[13], and scaled using SADABS[14]. The structure was solved by direct methods using SHELXT[15] and refined against F^2 on all data by full-matrix least squares with SHELXL-2018/3[16] following established refinement strategies.[17] All non-hydrogen atoms were refined anisotropically; except where specified below for all carborane cage hydrogen atoms, all hydrogen atoms were included into the model at geometrically calculated positions and refined using a riding model. The isotropic displacement parameters of all hydrogen atoms were fixed to 1.2 times the U value of the atoms they are linked to (1.5 times for methyl groups). Crystal and data quality details, as well as a summary of the residual refinement values, are listed in the accompanying table.

Compound **vl382at** crystallizes in the centrosymmetric triclinic space group *P*-1 with half of one molecule of dimeric **vl382** per asymmetric unit.

All carborane cage hydrogens were located in the Fourier synthesis and refined semi-freely with the help of distance restraints (target B–H distance 1.12(2) Å), and their U_{iso} values were constrained to 1.2 times the U_{eq} value of the boron atoms to which they bind.

Table S1. Crystal data and structure refinement for vl382at.

Identification code	vl382at	
Empirical formula	C30 H76 B18 Li2 O2	
Formula weight	677.36	
Temperature	100(2) K	
Wavelength	0.71073 Å	
Crystal system	Triclinic	
Space group	P-1	
Unit cell dimensions	a = 6.8342(3) Å	α = 87.6667(19)°.
	b = 8.9814(5) Å	β = 85.2398(19)°.
	c = 17.7841(8) Å	γ = 80.180(2)°.
Volume	1071.50(9) Å^3	
Z	1	
Density (calculated)	1.050 Mg/m^3	
Absorption coefficient	0.054 mm^{-1}	
F(000)	368	
Crystal color	colourless	
Crystal size	0.164 x 0.147 x 0.097 mm^3	
Theta range for data collection	2.299 to 31.506°	
Index ranges	-10 <= *h* <= 10, -13 <= *k* <= 13, -26 <= *l* <= 26	
Reflections collected	44152	
Independent reflections	7126 [R(int) = 0.0513]	
Completeness to theta = 25.242°	100.0 %	
Absorption correction	Semi-empirical from equivalents	
Refinement method	Full-matrix least-squares on F^2	
Data / restraints / parameters	7126 / 9 / 264	
Goodness-of-fit on F^2	1.098	
Final R indices [I>2sigma(I) = 6006 data]	R1 = 0.0546, wR2 = 0.1358	
R indices (all data, 0.68 Å)	R1 = 0.0658, wR2 = 0.1424	
Largest diff. peak and hole	0.512 and -0.253 e.Å^{-3}	

Table S2. Atomic coordinates (x 10^4) and equivalent isotropic displacement parameters (Å^2 x 10^3) for vl382at. U(eq) is defined as one third of the trace of the orthogonalized U^{ij} tensor.

Atom	x	y	z	U(eq)
B(1)	12439(2)	5770(1)	2284(1)	13(1)
B(2)	12991(2)	3793(1)	1996(1)	14(1)
B(3)	10465(2)	3960(1)	1676(1)	14(1)
B(4)	9920(2)	5939(1)	1968(1)	13(1)
B(5)	13991(2)	5245(1)	1431(1)	13(1)
B(6)	12598(2)	3970(1)	1001(1)	14(1)
B(7)	10428(2)	5494(1)	984(1)	14(1)
B(8)	11813(2)	6773(1)	1410(1)	14(1)
B(9)	12750(2)	5731(1)	645(1)	14(1)
C(1)	10975(1)	4548(1)	2468(1)	13(1)
C(2)	10226(1)	4055(1)	3250(1)	16(1)
C(3)	8007(2)	3960(1)	3340(1)	19(1)
C(4)	7377(2)	3377(1)	4126(1)	18(1)
C(5)	5167(2)	3254(1)	4237(1)	21(1)
C(6)	4600(2)	2558(1)	5003(1)	21(1)
C(7)	2397(2)	2428(1)	5128(1)	20(1)
C(8)	1865(2)	1641(1)	5875(1)	21(1)
C(9)	-308(2)	1428(1)	5969(1)	19(1)
C(10)	-832(2)	551(1)	6689(1)	21(1)
C(11)	-2992(2)	300(1)	6750(1)	25(1)
Li(1)	16191(3)	6462(2)	467(1)	21(1)
O(1)	16221(1)	8490(1)	735(1)	17(1)
C(12)	17668(2)	9362(1)	374(1)	19(1)
C(13)	17757(2)	10628(1)	906(1)	23(1)
C(14)	17104(2)	9977(1)	1673(1)	18(1)
C(15)	15513(2)	9117(1)	1468(1)	18(1)

Table S3. Bond lengths [Å] and angles [°] for vl382at.

B(1)-C(1)	1.6143(14)
B(1)-B(5)	1.8068(15)
B(1)-B(8)	1.8086(15)
B(1)-B(4)	1.8347(15)
B(1)-B(2)	1.8358(15)
B(1)-H(1)	1.094(11)
B(2)-C(1)	1.6125(14)
B(2)-B(5)	1.8049(15)
B(2)-B(6)	1.8072(16)
B(2)-B(3)	1.8422(15)
B(2)-H(2)	1.081(11)
B(3)-C(1)	1.6099(14)
B(3)-B(7)	1.8080(16)
B(3)-B(6)	1.8104(15)
B(3)-B(4)	1.8398(15)
B(3)-H(3)	1.097(11)
B(4)-C(1)	1.6069(14)
B(4)-B(7)	1.8030(15)
B(4)-B(8)	1.8097(15)
B(4)-H(4)	1.090(11)
B(5)-B(9)	1.6984(15)
B(5)-B(6)	1.8346(15)
B(5)-B(8)	1.8462(15)
B(5)-Li(1)	2.525(2)
B(5)-H(5)	1.121(11)
B(6)-B(9)	1.6960(16)
B(6)-B(7)	1.8400(16)
B(6)-Li(1)#1	2.696(2)
B(6)-H(6)	1.103(11)
B(7)-B(9)	1.6951(15)
B(7)-B(8)	1.8307(15)
B(7)-H(7)	1.103(11)
B(8)-B(9)	1.7009(15)
B(8)-H(8)	1.094(11)
B(9)-Li(1)	2.539(2)
B(9)-Li(1)#1	2.812(2)
B(9)-H(9)	1.100(12)

C(1)-C(2)	1.5158(13)
C(2)-C(3)	1.5283(14)
C(2)-H(2A)	0.9900
C(2)-H(2B)	0.9900
C(3)-C(4)	1.5268(14)
C(3)-H(3A)	0.9900
C(3)-H(3B)	0.9900
C(4)-C(5)	1.5290(14)
C(4)-H(4A)	0.9900
C(4)-H(4B)	0.9900
C(5)-C(6)	1.5246(14)
C(5)-H(5A)	0.9900
C(5)-H(5B)	0.9900
C(6)-C(7)	1.5275(14)
C(6)-H(6A)	0.9900
C(6)-H(6B)	0.9900
C(7)-C(8)	1.5261(14)
C(7)-H(7A)	0.9900
C(7)-H(7B)	0.9900
C(8)-C(9)	1.5245(14)
C(8)-H(8A)	0.9900
C(8)-H(8B)	0.9900
C(9)-C(10)	1.5246(14)
C(9)-H(9A)	0.9900
C(9)-H(9B)	0.9900
C(10)-C(11)	1.5254(15)
C(10)-H(10A)	0.9900
C(10)-H(10B)	0.9900
C(11)-H(11A)	0.9800
C(11)-H(11B)	0.9800
C(11)-H(11C)	0.9800
Li(1)-O(1)	1.905(2)
Li(1)-H(5)	2.063(13)
Li(1)-H(9)	2.271(15)
O(1)-C(15)	1.4524(12)
O(1)-C(12)	1.4569(12)
C(12)-C(13)	1.5199(15)
C(12)-H(12A)	0.9900
C(12)-H(12B)	0.9900

C(13)-C(14)	1.5255(15)
C(13)-H(13A)	0.9900
C(13)-H(13B)	0.9900
C(14)-C(15)	1.5122(14)
C(14)-H(14A)	0.9900
C(14)-H(14B)	0.9900
C(15)-H(15A)	0.9900
C(15)-H(15B)	0.9900
C(1)-B(1)-B(5)	108.88(8)
C(1)-B(1)-B(8)	108.92(7)
B(5)-B(1)-B(8)	61.41(6)
C(1)-B(1)-B(4)	55.09(6)
B(5)-B(1)-B(4)	102.40(7)
B(8)-B(1)-B(4)	59.56(6)
C(1)-B(1)-B(2)	55.28(6)
B(5)-B(1)-B(2)	59.40(6)
B(8)-B(1)-B(2)	102.44(7)
B(4)-B(1)-B(2)	90.12(7)
C(1)-B(1)-H(1)	118.3(7)
B(5)-B(1)-H(1)	122.8(7)
B(8)-B(1)-H(1)	123.6(7)
B(4)-B(1)-H(1)	130.2(7)
B(2)-B(1)-H(1)	128.9(7)
C(1)-B(2)-B(5)	109.05(8)
C(1)-B(2)-B(6)	108.77(8)
B(5)-B(2)-B(6)	61.05(6)
C(1)-B(2)-B(1)	55.37(6)
B(5)-B(2)-B(1)	59.50(6)
B(6)-B(2)-B(1)	102.08(7)
C(1)-B(2)-B(3)	55.07(6)
B(5)-B(2)-B(3)	102.24(7)
B(6)-B(2)-B(3)	59.47(6)
B(1)-B(2)-B(3)	90.01(7)
C(1)-B(2)-H(2)	119.3(7)
B(5)-B(2)-H(2)	122.5(7)
B(6)-B(2)-H(2)	122.6(7)
B(1)-B(2)-H(2)	130.1(7)
B(3)-B(2)-H(2)	130.0(7)
C(1)-B(3)-B(7)	108.47(8)

C(1)-B(3)-B(6)	108.73(7)
B(7)-B(3)-B(6)	61.13(6)
C(1)-B(3)-B(4)	55.04(6)
B(7)-B(3)-B(4)	59.23(6)
B(6)-B(3)-B(4)	102.04(7)
C(1)-B(3)-B(2)	55.20(6)
B(7)-B(3)-B(2)	101.80(7)
B(6)-B(3)-B(2)	59.30(6)
B(4)-B(3)-B(2)	89.76(7)
C(1)-B(3)-H(3)	120.1(7)
B(7)-B(3)-H(3)	122.6(7)
B(6)-B(3)-H(3)	121.7(7)
B(4)-B(3)-H(3)	131.0(7)
B(2)-B(3)-H(3)	129.9(7)
C(1)-B(4)-B(7)	108.85(8)
C(1)-B(4)-B(8)	109.20(7)
B(7)-B(4)-B(8)	60.89(6)
C(1)-B(4)-B(1)	55.47(6)
B(7)-B(4)-B(1)	101.91(7)
B(8)-B(4)-B(1)	59.50(6)
C(1)-B(4)-B(3)	55.19(6)
B(7)-B(4)-B(3)	59.50(6)
B(8)-B(4)-B(3)	102.32(7)
B(1)-B(4)-B(3)	90.12(7)
C(1)-B(4)-H(4)	118.8(7)
B(7)-B(4)-H(4)	123.4(7)
B(8)-B(4)-H(4)	122.2(7)
B(1)-B(4)-H(4)	129.2(7)
B(3)-B(4)-H(4)	130.5(7)
B(9)-B(5)-B(2)	112.13(8)
B(9)-B(5)-B(1)	111.91(8)
B(2)-B(5)-B(1)	61.10(6)
B(9)-B(5)-B(6)	57.22(6)
B(2)-B(5)-B(6)	59.54(6)
B(1)-B(5)-B(6)	102.14(7)
B(9)-B(5)-B(8)	57.17(6)
B(2)-B(5)-B(8)	102.18(7)
B(1)-B(5)-B(8)	59.34(6)
B(6)-B(5)-B(8)	90.03(7)

B(9)-B(5)-Li(1)	70.84(7)
B(2)-B(5)-Li(1)	159.80(8)
B(1)-B(5)-Li(1)	137.80(8)
B(6)-B(5)-Li(1)	112.28(7)
B(8)-B(5)-Li(1)	96.02(7)
B(9)-B(5)-H(5)	123.1(7)
B(2)-B(5)-H(5)	116.5(7)
B(1)-B(5)-H(5)	116.4(7)
B(6)-B(5)-H(5)	132.8(7)
B(8)-B(5)-H(5)	132.3(7)
Li(1)-B(5)-H(5)	53.4(7)
B(9)-B(6)-B(2)	112.13(8)
B(9)-B(6)-B(3)	112.10(8)
B(2)-B(6)-B(3)	61.22(6)
B(9)-B(6)-B(5)	57.35(6)
B(2)-B(6)-B(5)	59.41(6)
B(3)-B(6)-B(5)	102.32(7)
B(9)-B(6)-B(7)	57.11(6)
B(2)-B(6)-B(7)	101.92(7)
B(3)-B(6)-B(7)	59.37(6)
B(5)-B(6)-B(7)	89.84(7)
B(9)-B(6)-Li(1)#1	75.86(7)
B(2)-B(6)-Li(1)#1	152.49(8)
B(3)-B(6)-Li(1)#1	142.27(8)
B(5)-B(6)-Li(1)#1	111.88(7)
B(7)-B(6)-Li(1)#1	104.16(7)
B(9)-B(6)-H(6)	120.5(7)
B(2)-B(6)-H(6)	119.5(7)
B(3)-B(6)-H(6)	117.2(7)
B(5)-B(6)-H(6)	133.5(7)
B(7)-B(6)-H(6)	130.4(7)
Li(1)#1-B(6)-H(6)	44.8(7)
B(9)-B(7)-B(4)	112.54(8)
B(9)-B(7)-B(3)	112.26(8)
B(4)-B(7)-B(3)	61.26(6)
B(9)-B(7)-B(8)	57.53(6)
B(4)-B(7)-B(8)	59.74(6)
B(3)-B(7)-B(8)	102.75(7)
B(9)-B(7)-B(6)	57.16(6)

B(4)-B(7)-B(6)	102.32(7)
B(3)-B(7)-B(6)	59.50(6)
B(8)-B(7)-B(6)	90.35(7)
B(9)-B(7)-H(7)	119.9(7)
B(4)-B(7)-H(7)	120.0(7)
B(3)-B(7)-H(7)	117.0(7)
B(8)-B(7)-H(7)	133.7(7)
B(6)-B(7)-H(7)	129.3(7)
B(9)-B(8)-B(1)	111.70(8)
B(9)-B(8)-B(4)	111.92(8)
B(1)-B(8)-B(4)	60.93(6)
B(9)-B(8)-B(7)	57.23(6)
B(1)-B(8)-B(7)	101.85(7)
B(4)-B(8)-B(7)	59.37(6)
B(9)-B(8)-B(5)	57.04(6)
B(1)-B(8)-B(5)	59.24(6)
B(4)-B(8)-B(5)	101.83(7)
B(7)-B(8)-B(5)	89.77(7)
B(9)-B(8)-H(8)	119.9(7)
B(1)-B(8)-H(8)	119.4(7)
B(4)-B(8)-H(8)	119.2(7)
B(7)-B(8)-H(8)	131.6(7)
B(5)-B(8)-H(8)	131.8(7)
B(7)-B(9)-B(6)	65.72(6)
B(7)-B(9)-B(5)	99.75(7)
B(6)-B(9)-B(5)	65.43(7)
B(7)-B(9)-B(8)	65.24(6)
B(6)-B(9)-B(8)	100.07(7)
B(5)-B(9)-B(8)	65.79(7)
B(7)-B(9)-Li(1)	164.50(8)
B(6)-B(9)-Li(1)	117.17(8)
B(5)-B(9)-Li(1)	69.97(7)
B(8)-B(9)-Li(1)	99.49(7)
B(7)-B(9)-Li(1)#1	103.95(7)
B(6)-B(9)-Li(1)#1	68.36(6)
B(5)-B(9)-Li(1)#1	111.65(7)
B(8)-B(9)-Li(1)#1	167.27(8)
Li(1)-B(9)-Li(1)#1	90.86(7)
B(7)-B(9)-H(9)	127.9(8)

B(6)-B(9)-H(9)	129.7(8)
B(5)-B(9)-H(9)	132.4(8)
B(8)-B(9)-H(9)	130.2(8)
Li(1)-B(9)-H(9)	63.4(8)
Li(1)#1-B(9)-H(9)	61.4(8)
C(2)-C(1)-B(4)	126.08(8)
C(2)-C(1)-B(3)	126.73(8)
B(4)-C(1)-B(3)	69.77(7)
C(2)-C(1)-B(2)	126.30(8)
B(4)-C(1)-B(2)	107.61(8)
B(3)-C(1)-B(2)	69.73(7)
C(2)-C(1)-B(1)	125.72(8)
B(4)-C(1)-B(1)	69.44(6)
B(3)-C(1)-B(1)	107.55(7)
B(2)-C(1)-B(1)	69.35(6)
C(1)-C(2)-C(3)	114.73(8)
C(1)-C(2)-H(2A)	108.6
C(3)-C(2)-H(2A)	108.6
C(1)-C(2)-H(2B)	108.6
C(3)-C(2)-H(2B)	108.6
H(2A)-C(2)-H(2B)	107.6
C(4)-C(3)-C(2)	111.98(9)
C(4)-C(3)-H(3A)	109.2
C(2)-C(3)-H(3A)	109.2
C(4)-C(3)-H(3B)	109.2
C(2)-C(3)-H(3B)	109.2
H(3A)-C(3)-H(3B)	107.9
C(3)-C(4)-C(5)	113.55(9)
C(3)-C(4)-H(4A)	108.9
C(5)-C(4)-H(4A)	108.9
C(3)-C(4)-H(4B)	108.9
C(5)-C(4)-H(4B)	108.9
H(4A)-C(4)-H(4B)	107.7
C(6)-C(5)-C(4)	112.76(9)
C(6)-C(5)-H(5A)	109.0
C(4)-C(5)-H(5A)	109.0
C(6)-C(5)-H(5B)	109.0
C(4)-C(5)-H(5B)	109.0
H(5A)-C(5)-H(5B)	107.8

C(5)-C(6)-C(7)	113.73(9)
C(5)-C(6)-H(6A)	108.8
C(7)-C(6)-H(6A)	108.8
C(5)-C(6)-H(6B)	108.8
C(7)-C(6)-H(6B)	108.8
H(6A)-C(6)-H(6B)	107.7
C(8)-C(7)-C(6)	113.46(9)
C(8)-C(7)-H(7A)	108.9
C(6)-C(7)-H(7A)	108.9
C(8)-C(7)-H(7B)	108.9
C(6)-C(7)-H(7B)	108.9
H(7A)-C(7)-H(7B)	107.7
C(9)-C(8)-C(7)	112.87(9)
C(9)-C(8)-H(8A)	109.0
C(7)-C(8)-H(8A)	109.0
C(9)-C(8)-H(8B)	109.0
C(7)-C(8)-H(8B)	109.0
H(8A)-C(8)-H(8B)	107.8
C(8)-C(9)-C(10)	113.58(9)
C(8)-C(9)-H(9A)	108.8
C(10)-C(9)-H(9A)	108.8
C(8)-C(9)-H(9B)	108.8
C(10)-C(9)-H(9B)	108.8
H(9A)-C(9)-H(9B)	107.7
C(9)-C(10)-C(11)	112.27(10)
C(9)-C(10)-H(10A)	109.1
C(11)-C(10)-H(10A)	109.1
C(9)-C(10)-H(10B)	109.1
C(11)-C(10)-H(10B)	109.1
H(10A)-C(10)-H(10B)	107.9
C(10)-C(11)-H(11A)	109.5
C(10)-C(11)-H(11B)	109.5
H(11A)-C(11)-H(11B)	109.5
C(10)-C(11)-H(11C)	109.5
H(11A)-C(11)-H(11C)	109.5
H(11B)-C(11)-H(11C)	109.5
O(1)-Li(1)-B(5)	109.74(9)
O(1)-Li(1)-B(9)	113.22(9)
B(5)-Li(1)-B(9)	39.19(4)

O(1)-Li(1)-B(6)#1	111.16(9)
B(5)-Li(1)-B(6)#1	135.07(8)
B(9)-Li(1)-B(6)#1	105.76(8)
O(1)-Li(1)-B(9)#1	146.32(10)
B(5)-Li(1)-B(9)#1	103.48(7)
B(9)-Li(1)-B(9)#1	89.14(7)
B(6)#1-Li(1)-B(9)#1	35.78(4)
O(1)-Li(1)-H(5)	107.3(4)
B(5)-Li(1)-H(5)	25.9(3)
B(9)-Li(1)-H(5)	64.7(3)
B(6)#1-Li(1)-H(5)	140.7(4)
B(9)#1-Li(1)-H(5)	105.0(4)
O(1)-Li(1)-H(9)	113.8(4)
B(5)-Li(1)-H(9)	64.6(3)
B(9)-Li(1)-H(9)	25.7(3)
B(6)#1-Li(1)-H(9)	82.3(3)
B(9)#1-Li(1)-H(9)	75.6(4)
H(5)-Li(1)-H(9)	89.5(4)
C(15)-O(1)-C(12)	108.88(7)
C(15)-O(1)-Li(1)	124.81(8)
C(12)-O(1)-Li(1)	121.60(8)
O(1)-C(12)-C(13)	106.19(8)
O(1)-C(12)-H(12A)	110.5
C(13)-C(12)-H(12A)	110.5
O(1)-C(12)-H(12B)	110.5
C(13)-C(12)-H(12B)	110.5
H(12A)-C(12)-H(12B)	108.7
C(12)-C(13)-C(14)	102.90(8)
C(12)-C(13)-H(13A)	111.2
C(14)-C(13)-H(13A)	111.2
C(12)-C(13)-H(13B)	111.2
C(14)-C(13)-H(13B)	111.2
H(13A)-C(13)-H(13B)	109.1
C(15)-C(14)-C(13)	101.55(8)
C(15)-C(14)-H(14A)	111.5
C(13)-C(14)-H(14A)	111.5
C(15)-C(14)-H(14B)	111.5
C(13)-C(14)-H(14B)	111.5
H(14A)-C(14)-H(14B)	109.3

O(1)-C(15)-C(14)	105.23(8)
O(1)-C(15)-H(15A)	110.7
C(14)-C(15)-H(15A)	110.7
O(1)-C(15)-H(15B)	110.7
C(14)-C(15)-H(15B)	110.7
H(15A)-C(15)-H(15B)	108.8

Symmetry transformations used to generate equivalent atoms: #1 -x+3,-y+1,-z

Table S4. Anisotropic displacement parameters (Å^2 x 10^3) for vl382at. The anisotropic displacement factor exponent takes the form: $-2\pi^2[h^2 a^{*2}U^{11} + ... + 2 h k a^* b^* U^{12}]$

Atom	U^{11}	U^{22}	U^{33}	U^{23}	U^{13}	U^{12}
B(1)	9(1)	15(1)	15(1)	0(1)	0(1)	-3(1)
B(2)	10(1)	14(1)	16(1)	1(1)	2(1)	-1(1)
B(3)	11(1)	15(1)	16(1)	-2(1)	2(1)	-4(1)
B(4)	9(1)	15(1)	15(1)	0(1)	1(1)	-2(1)
B(5)	8(1)	16(1)	15(1)	1(1)	1(1)	-2(1)
B(6)	11(1)	16(1)	15(1)	-2(1)	2(1)	-2(1)
B(7)	9(1)	17(1)	15(1)	0(1)	0(1)	-2(1)
B(8)	9(1)	14(1)	17(1)	1(1)	1(1)	-2(1)
B(9)	9(1)	18(1)	14(1)	2(1)	1(1)	-2(1)
C(1)	9(1)	14(1)	14(1)	0(1)	2(1)	-3(1)
C(2)	12(1)	20(1)	15(1)	2(1)	3(1)	-4(1)
C(3)	12(1)	27(1)	19(1)	4(1)	3(1)	-6(1)
C(4)	14(1)	23(1)	17(1)	1(1)	5(1)	-6(1)
C(5)	16(1)	28(1)	20(1)	1(1)	4(1)	-9(1)
C(6)	16(1)	27(1)	20(1)	1(1)	5(1)	-8(1)
C(7)	16(1)	26(1)	19(1)	-1(1)	5(1)	-9(1)
C(8)	16(1)	26(1)	20(1)	0(1)	5(1)	-7(1)
C(9)	17(1)	23(1)	18(1)	-2(1)	6(1)	-8(1)
C(10)	20(1)	20(1)	21(1)	0(1)	7(1)	-4(1)
C(11)	23(1)	25(1)	27(1)	-4(1)	11(1)	-10(1)
Li(1)	17(1)	22(1)	24(1)	-1(1)	2(1)	-5(1)
O(1)	15(1)	19(1)	18(1)	-2(1)	3(1)	-7(1)
C(12)	18(1)	21(1)	19(1)	2(1)	3(1)	-7(1)
C(13)	24(1)	18(1)	27(1)	-1(1)	4(1)	-9(1)
C(14)	14(1)	18(1)	21(1)	-3(1)	-1(1)	-2(1)
C(15)	13(1)	22(1)	18(1)	-3(1)	2(1)	-4(1)

Table S5. Hydrogen coordinates (x 10^4) and isotropic displacement parameters (Å^2 x 10^3) for vl382at.

Atom	x	y	z	U(eq)
H(1)	12970(20)	6290(14)	2757(7)	16
H(2)	13929(19)	2832(13)	2237(7)	17
H(3)	9497(19)	3104(14)	1667(7)	17
H(4)	8536(18)	6613(14)	2197(7)	16
H(5)	15623(17)	5258(15)	1450(7)	16
H(6)	13040(20)	2976(14)	643(7)	17
H(7)	9205(18)	5667(15)	605(7)	17
H(8)	11690(20)	8005(13)	1377(7)	16
H(9)	13240(20)	6094(17)	72(7)	28(4)
H(2A)	10500	4775	3619	19
H(2B)	10991	3050	3376	19
H(3A)	7225	4975	3247	23
H(3B)	7706	3277	2958	23
H(4A)	7679	4065	4506	22
H(4B)	8176	2368	4218	22
H(5A)	4365	4276	4186	25
H(5B)	4838	2630	3834	25
H(6A)	4939	3181	5404	25
H(6B)	5406	1537	5052	25
H(7A)	1593	3454	5112	24
H(7B)	2036	1859	4709	24
H(8A)	2729	640	5906	25
H(8B)	2138	2246	6296	25
H(9A)	-602	885	5529	23
H(9B)	-1167	2433	5971	23
H(10A)	-601	1114	7131	25
H(10B)	59	-441	6698	25
H(11A)	-3881	1277	6787	37
H(11B)	-3236	-327	7200	37
H(11C)	-3246	-212	6300	37
H(12A)	17245	9781	-121	23
H(12B)	18991	8716	295	23
H(13A)	16837	11558	771	27
H(13B)	19124	10858	898	27

H(14A)	18219	9295	1894	21
H(14B)	16555	10786	2031	21
H(15A)	15343	8302	1847	21
H(15B)	14222	9803	1438	21

References

[1] [a] Brellochs, B.; Bačkovský, J.; Štíbr, B.; Jelínek, T.; Holub, J.; Bakardjiev, M.; Hnyk, D.; Hofmann, M.; Císarová, I.; Wrackmeyer, B. *Eur. J. Inorg. Chem.* **2004**, *2004*, 3605 [b] Ringstrand, B.; Bateman, D.; Shoemaker, R. K.; Janousek, Z. *Collection of Czechoslovak Chem. Commun.* **2009**, 419.

[2] Jelinek, T.; Baldwin, P.; Scheidt, W. R.; Reed, C. A. *Inorg. Chem.* **1993**, *32*, 1982.

[3] [a] Kresse, G.; Hafner, J. *Physical Review B* 1994, *49*, 14251 [b] Kresse, G.; Furthmüller, J. *Phys. Rev. B* 1996, *54*, 11169.

[4] Blöchl, P. E. J. *Phys. Rev. B* **1994**, *50*, 17953.

[5] Perdew, J. P.; Burke, K.; Ernzerhof, M. *Phys. Rev. Lett.***1996**, *77*, 3865.

[6] Johnson, E. R.; Becke, A. D. *J. Chem.Phys.* **2006**, *124*, 174104.

[7] Plimpton, S. *J. Comput. Phys.* **1995**, *117*, 1.

[8] Rappe, A. K.; Casewit, C. J.; Colwell, K. S.; Goddard, W. A.; Skiff, W. M. *J. Am. Chem. Soc.* **1992**, *114*, 10024.

[9] Salanne, M. *Phys. Chem. Chem.Phys.* **2015**, *17*, 14270.

[10] Youngs, T. G. A.; Hardacre, C. *ChemPhysChem* **2008**, *9*, 1548.

[11] Frisch, M. J.; Trucks, G. W.; Schlegel, H. B.; Scuseria, G. E.; Robb, M. A.; Cheeseman, J. R.; Scalmani, G.; Barone, V.; Petersson, G. A.; Nakatsuji, H.; Li, X.; Caricato, M.; Marenich, A. V.; Bloino, J.; Janesko, B. G.; Gomperts, R.; Mennucci, B.; Hratchian, H. P.; Ortiz, J. V.; Izmaylov, A. F.; Sonnenberg, J. L.; Williams; Ding, F.; Lipparini, F.; Egidi, F.; Goings, J.; Peng, B.; Petrone, A.; Henderson, T.; Ranasinghe, D.; Zakrzewski, V. G.; Gao, J.; Rega, N.; Zheng, G.; Liang, W.; Hada, M.; Ehara, M.; Toyota, K.; Fukuda, R.; Hasegawa, J.; Ishida, M.; Nakajima, T.; Honda, Y.; Kitao, O.; Nakai, H.; Vreven, T.; Throssell, K.; Montgomery Jr., J. A.; Peralta, J. E.; Ogliaro, F.; Bearpark, M. J.; Heyd, J. J.; Brothers, E. N.; Kudin, K. N.; Staroverov, V. N.; Keith, T. A.; Kobayashi, R.; Normand, J.; Raghavachari, K.; Rendell, A. P.; Burant, J. C.; Iyengar, S. S.; Tomasi, J.; Cossi, M.; Millam, J. M.; Klene, M.; Adamo, C.; Cammi, R.; Ochterski, J. W.; Martin, R. L.; Morokuma, K.; Farkas, O.; Foresman, J. B.; Fox, D. J. Wallingford, CT, **2016**.

[12] Humphrey, W.; Dalke, A.; Schulten, K. *J. Mol. Graph.* 1996, *14*, 33.

[13] SAINT, V8.30A, Bruker Analytical X-Ray Systems, Madison, WI, **2012**.

[14] SADABS, 2.03, Bruker Analytical X-Ray Systems, Madison, WI, **2016**.

[15] Sheldrick, G. *Acta Crysta. A* **2015**, *71*, 3.

[16] Sheldrick, G. *Acta Crysta. C* **2015**, *71*, 3.

[17] Müller, P. *Crystallogr. Rev. b***2009**, *15*, 57.
