# Supplementary material for: Combustion Resistant Borohydrides and Their Chemical Interactions with Li-Metal Surfaces: An Experimental and Theoretical Study
Source: ACS Cent Sci. 2025 Apr 23;11(5):734–41. doi: 10.1021/acscentsci.5c00043 (PMC12164935; doi:10.1021/acscentsci.5c00043)
Supplement: Supplementary file 3 [file oc5c00043_si_003.pdf]

oc-2025-00043n.R1

Name: Peer Review Information for "Combustion Resistant Borohydrides and their Chemical Interactions with Li-Metal Surfaces: An Experimental and Theoretical Study"

First Round of Reviewer Comments

Reviewer: 1

Comments to the Author

The work of Goddard, Guo, Lavallo and coworkers presents the synthesis of a number of highly stable carborane clusters with lithium counterions, and presents an exploration of their physical properties. The compounds do not combust in an open flame, and are stable on a Li surface for a month, undergoing only a cage opening process that is reversed when the Li is removed. One of the compounds is also an ionic liquid, which is very interesting given its very high stability. The work is very thoroughly performed, is strongly interdisciplinary, and will have an impact on a number of applications (e.g. battery electrolytes, ionic liquids), making it suitable and worthy of publication in ACS Central Science. I found the work to be quite fascinating and believe it will make a great addition to the journal. There is little to complain about, other than a couple of minor things:

- Most of the NMR spectra are missing from the SI, they just appear as blank spaces despite opening the PDF file in a number of different programs. I assume they are fine but it would be good to see them.

- Fig 5 - the colored "(b)"s in part d are not defined. Are they supposed to be unoccupied sites?

Reviewer: 2

Comments to the Author

The authors describe the very surprising behavior of a carborane salt which is combustion resistant in flame tests and not reduced upon exposure Li metal. Another great example of the versatile chemistry of this class of compounds. The compound has attractive physical properties for potential energy storage applications and is a wonderful demonstration of how tuning chemical structure can lead to significant advances in material properties, topics of broad interest to the scientific community. The authors included theoretical work that rationalizes the observed resistance to irreversible decomposition by Li metal makes for a complete story with opportunities for further advances and applications in material science.

On line 33 of page 2: it may be of interest to mention that boranes were also investigated for high energy fuels and additives for applications beyond just rocket engines, as mentioned in reference 6.

My judgement is that the manuscript is ready to publish as-is. But revisions need to be made to the supporting information document as nearly all the figures in the supporting information pdf file are missing or incomplete. This was confirmed with the Journal Office Administrator (case CSCSI0268700). Figures with issues are: S1, S2, S3, S4, S5 through S7 have only 1 image, S8 through S27, S29 through S35, S36 through S38 have only 1 image, S39 through S54, S56 through S57 have only 1 image, S58 through S61, S66 through S68 have only 1 image, S75 through S77 have only 1 image, S82 through S86, S87 through S90 have only 1 image, S91 through S94, S95 through S97 have only 1 image, S98 through S100, S101 through S103 have only 1 image, S118 through S131.

Reviewer: 3

Comments to the Author

This work investigates combustion-resistant borohydrides and their chemical interactions with lithium (Li) metal surfaces. This research identifies Li+ salts of carborane anions that are resistant to combustion, even under direct flame exposure. One of the key discoveries is that a Li+ carborane salt can form a unique ionic liquid that remains stable in the presence of Li metal. This stability allows for direct interaction studies of the carborane anion with Li-metal surfaces in a solvent-free environment. The study employs experimental techniques (such as XPS, SEM, and NMR) and computational simulations

(DFT, MD simulations) to confirm that these materials do not undergo irreversible decomposition when exposed to Li metal. However, to be published, the work could be improved considering major corrections.

1) If I understood the described model, Figure 1 shows the synthesis details where two species were synthesized: LiC10-THF and LiC4-THF. Then, for the balls and sticks model shown, gray, white, and pink are related to carbon, hydrogen, and boron, right? If so, this should be described, and the implicit hydrogen model for the decyl and butyl groups should be well described.

2) I don't know if that occurred only with the file I have downloaded, but Figures S1-S7 with NMR data are not shown, and others as well.

3) The first citation of the data from Supporting Material is due to the NMR in Figures S69-S74. However, as shown at the beginning of the results, all the discussions about the synthesis described in Figure 1 contain information supported by the SI that could be used, but they were not used. All the NMR data from SI are not being appropriately shown; and they are missing from the PDF file, at least when I open the file in two different softwares. As all the discussions in the main text are based on the NMR data in the SI, it became a complicated evaluation. Beyond that, many of the data included in the SI were not used and discussed in the main text. The description of the Supporting Information section (at the end of the main manuscript) is quite poor as well. It should be improved with details about the available data. In general, the results' organization and discussion are confusing and can be improved. The care the authors had to write the Introduction was not the same as the results and discussion.

4) Figure 5(d) shows the closo- and arachno- shapes for the anion. It presents a (b) atomic position in blue and red, which seems to be the position where Li is located when coordinating with the anion structure. Is that interpretation correct, or are these voids created when the structure is rearranged? Anyway, this label in Figure was not described even in the main text. The authors should clarify that in a revised version.

5) In the computational methods section, the authors described that they used a quantum mechanical approach to derive charges for the carborane anion. Because the Mulliken method always overstimates the partial atomic charges in gas phase, and this should be pronounced for isolated ions, could you explain this choice instead of a method that can reproduce the dipole moment better based on the electrostatic potential and also based on ion pair or cluster?

6) Thinking about the charge dispersion and the care that the authors had with the van der Waals treatment for the DFT calculations, why use B3LYP/6-31G(d,p) level of theory to derive the charges? Why not use dispersion correction and an adequate basis set for anionic species? The authors should justify this choice.

7) While the study provides solid chemical stability insights, long-term cycling tests for battery applications are not extensively discussed, and electrochemical performance metrics (e.g., ionic conductivity, SEI formation over repeated cycles) need further validation. If that is not the focus of the manuscript, maybe the authors should clarify this to the readers. Could you explain more about this aspect?

8) Could the authors comment about the costs and complexity to get the carborane based compounds compared to conventional electrolyte materials?

Author's Response to Peer Review Comments:

Dear Editorial Team we have adressed the reviewers comments point by point below and now believe the manuscript is ready for publication. Revised Manuscript files have been uploaded accordingly.

Reviewer 1:

The work of Goddard, Guo, Lavallo and coworkers presents the synthesis of a number of highly stable carborane clusters with lithium counterions, and presents an exploration of their physical properties. The compounds do not combust in an open flame, and are stable on a Li surface for a month, undergoing only a cage opening process that is reversed when the Li is removed. One of the compounds is also an ionic liquid, which is very interesting given its very high stability. The work is very thoroughly performed, is strongly

interdisciplinary, and will have an impact on a number of applications (e.g. battery electrolytes, ionic liquids), making it suitable and worthy of publication in ACS Central Science. I found the work to be quite fascinating and believe it will make a great addition to the journal. There is little to complain about, other than a couple of minor things:

R1 Comment 1:
Most of the NMR spectra are missing from the SI, they just appear as blank spaces despite opening the PDF file in a number of different programs. I assume they are fine but it would be good to see them.

Response:
We thank the reviewer for pointing out this error. This has been brought to attention by all of the referees and may have resulted from a file upload error. We have taken the time to make sure the NMR spectra are indeed present and the file format of the images is compatible with the submission portal.

R1 Comment 2:
Fig 5 - the colored "(b)"s in part d are not defined. Are they supposed to be unoccupied sites?
Response:
Thank you for bringing this to our attention. Indeed, the "(b)" s are intended to denote icosahedral sites occupied by Li upon reduction of the cluster to form the arachno- species described in our MD simulations.
The following revisions have been made to the main text and figure 4 caption for clarification:
"...(d) Schematic figure representing the reversible cage opening reaction of the carborane molecule ("b" denotes icosahedral site occupied by Li+ following reduction)."
"...as one or two Li atoms are incorporated as Li+ into the cluster (Li+ occupation denoted by "b").

Reviewer 2:
The authors describe the very surprising behavior of a carborane salt which is combustion resistant in flame tests and not reduced upon exposure Li metal. Another great example of the versatile chemistry of this class of compounds. The compound has attractive physical properties for potential energy storage applications and is a wonderful demonstration of how tuning chemical structure can lead to significant advances in material properties, topics of broad interest to the scientific community. The authors included theoretical work

that rationalizes the observed resistance to irreversible decomposition by Li metal makes for a complete story with opportunities for further advances and applications in material science.

R2 Comment 1:
On line 33 of page 2: it may be of interest to mention that boranes were also investigated for high energy fuels and additives for applications beyond just rocket engines, as mentioned in reference 6.
Response:
Thank you for the comment. This is an excellent suggestion considering the versatile nature of borohydrides is a key takeaway from this work. We think the reader will take great interest in the combustion properties of borohydrides outlined in reference 6 considering the properties of the materials presented within this manuscript exhibit the opposite behavior.
The following revision has been made to the main text in support of this correction:
"…At the beginning of the cold war the energy content and combustibility of these materials drew the attention of military powers and the petroleum industry as potential candidates for high energy rocket fuels and gasoline additives, respectively. Ultimately their application as high energy fuels was not effective because the extremely hard BO byproducts destroyed the rocket engines. 3-6"

R2 Comment 2: My judgement is that the manuscript is ready to publish as-is. But revisions need to be made to the supporting information document as nearly all the figures in the supporting information pdf file are missing or incomplete. This was confirmed with the Journal Office Administrator (case CSCSI0268700). Figures with issues are: S1, S2, S3, S4, S5 through S7 have only 1 image, S8 through S27, S29 through S35, S36 through S38 have only 1 image, S39 through S54, S56 through S57 have only 1 image, S58 through S61, S66 through S68 have only 1 image, S75 through S77 have only 1 image, S82 through S86, S87 through S90 have only 1 image, S91 through S94, S95 through S97 have only 1 image, S98 through S100, S101 through S103 have only 1 image, S118 through S131.
Response:
We thank the reviewer for pointing out this error. This has been brought to attention by all of the referees and may have resulted from a file upload error. We have taken the time to make sure the NMR spectra are indeed present and the file format of the images is compatible with the submission portal.

Reviewer 3:
This work investigates combustion-resistant borohydrides and their chemical interactions with lithium (Li) metal surfaces. This research identifies Li+ salts of carborane anions that are resistant to combustion, even under direct flame exposure. One of the key discoveries is that a Li+ carborane salt can form a unique ionic liquid that remains stable in the presence of Li metal. This stability allows for direct interaction studies of the carborane anion with Li-metal surfaces in a solvent-free environment. The study employs experimental techniques (such as XPS, SEM, and NMR) and computational simulations (DFT, MD simulations) to confirm that these materials do not undergo irreversible decomposition when exposed to Li metal. However, to be published, the work could be improved considering major corrections.

R3 Comment 1:
If I understood the described model, Figure 1 shows the synthesis details where two species were synthesized: LiC10-THF and LiC4-THF. Then, for the balls and sticks model shown, gray, white, and pink are related to carbon, hydrogen, and boron, right? If so, this should be described, and the implicit hydrogen model for the decyl and butyl groups should be well described.

Response:
We thank the reviewer for pointing out the colors defining individual nuclei have not been defined. Indeed, gray represents Carbon, white hydrogen, pink boron, and purple lithium. The following highlighted changes have been made to the caption of Fig. 1 in support of this correction:
"Figure 1. (a) Synthetic scheme detailing [HCB9H9 1-] functionalization and desolvation procedure (brown, unlabeled vertices indicate B-H). (b) Solid-state structure of LiC10-THF depicting distorted tetrahedral coordination environment of Li+ (Boron, pink; Carbon, grey; Oxygen, red; Lithium, purple; Hydrogen, white. Hydrogen omitted from alkyl moiety for clarity) . (c) Photo of room temperature ionic liquid LiC4 -THF

R3 Comment 2:
I don't know if that occurred only with the file I have downloaded, but Figures S1-S7 with NMR data are not shown, and others as well.

Response:
We thank the reviewer for pointing out this error. This has been brought to attention by all of the referees and may have resulted from a file upload error. We have taken the time to make sure the NMR spectra are indeed present and the file format of the images is compatible with the submission portal. Please see the updated supplementary information with referenced NMR spectra.

R3 Comment 3:
The first citation of the data from Supporting Material is due to the NMR in Figures S69-S74. However, as shown at the beginning of the results, all the discussions about the synthesis described in Figure 1 contain information supported by the SI that could be used, but they were not used.

Response:
We thank the reviewer for pointing out the ambiguity here. The synthetic scheme in Fig. 1 is truncated for brevity – the full synthetic route used in this study consists of a series of salt metatheses which substantially aid in the purity of the resulting materials. That being said, the synthetic scheme outlined in Fig. 1a is not inaccurate – you can obtain the same compounds through the scheme outlined here. To direct the reader to the preferred approach to obtaining these compounds, we have made the following changes to the main text.
While compound LiC10-THF4 could be obtained directly following alkylation of the dianionic [CB9H9]2- species, a modified literature procedure was employed to achieve Li salts of alkylated carborane species in high purity (Fig. 1a, S8-10, 58-84, 104-117).
Additionally, the Supporting Information description has been updated to read "detailed synthetic information" in service to this point as well as a later note.

C3 Continued:
"All the NMR data from SI are not being appropriately shown; and they are missing from the PDF file... As all the discussions in the main text are based on the NMR data in the SI, it became a complicated evaluation."
As the other referees have pointed out, all of the NMR data was missing in the submitted SI. We suspect this may have resulted from an improper file formatting of the spectra images. We have taken the time to make sure the NMR spectra are indeed present and the file format is compatible with the submission portal.

C3 Continued:
"...Beyond that, many of the data included in the SI were not used and discussed in the

main text."
Thank you for pointing this out. We have taken the time to ensure all of the SI spectra are referenced within the main text in service of clarity to the reader.

C3 Continued:
"…The description of the Supporting Information section (at the end of the main manuscript) is quite poor as well. It should be improved with details about the available data."
We thank the reviewer and editor for pointing out additional detail is required here. The following changes have been made at the end of the manuscript in the Supporting Information section:
Supporting Information:
"Contains additional experimental details, materials, and methods including detailed synthetic information, accompanying multinuclear NMR (11B, 1H, 13C, 7Li, 23Na), theoretical calculations, and crystal structure refinement.

C3 Continued:
In general, the results' organization and discussion are confusing and can be improved. The care the authors had to write the Introduction was not the same as the results and discussion.
Thank you for the feedback. We hope the modifications and attention given to the main text with the help of your comments have resulted in a manuscript that is more accessible to the reader. It is a challenge to organize the large number of supporting NMR spectra in the SI while maintaining coherency in the main text. We found many unreferenced SI images during the editing process and feel confident the main text will now direct the reader to the appropriate spectra. Ultimately, we feel the edits made to this end provide a lot of clarity to our overall process while concentrating full, multinuclear characterization of individual compounds appropriately in the supporting document.

R3 Comment 4:
Figure 5(d) shows the closo- and arachno- shapes for the anion. It presents a (b) atomic position in blue and red, which seems to be the position where Li is located when coordinating with the anion structure. Is that interpretation correct, or are these voids created when the structure is rearranged? Anyway, this label in Figure was not described even in the main text. The authors should clarify that in a revised version.

Response:
We thank the reviewer for pointing out the issue with clarity in this portion of the

manuscript. Please see our response to Reviewer 1, Comment 2 where we have addressed this point.

R3 Comment 5:
In the computational methods section, the authors described that they used a quantum mechanical approach to derive charges for the carborane anion. Because the Mulliken method always overestimates the partial atomic charges in gas phase, and this should be pronounced for isolated ions, could you explain this choice instead of a method that can reproduce the dipole moment better based on the electrostatic potential and also based on ion pair or cluster?

Response:
We thank the reviewer for catching this mistake. Indeed, there is no discussion of these results in the main text as we intend for the MD study on transport properties to be explored in a separate paper alluded to in the conclusion of this manuscript. We apologize for the confusion as we did not intend to include this portion in the supporting document.

R3 Comment 6:
Thinking about the charge dispersion and the care that the authors had with the van der Waals treatment for the DFT calculations, why use B3LYP/6-31G(d,p) level of theory to derive the charges? Why not use dispersion correction and an adequate basis set for anionic species? The authors should justify this choice.

Response:
We apologize for the confusion these results have introduced however we are appreciate the reviewers interest in this experiment. Please see our response to your previous comment.

R3 Comment 7:
While the study provides solid chemical stability insights, long-term cycling tests for battery applications are not extensively discussed, and electrochemical performance metrics (e.g., ionic conductivity, SEI formation over repeated cycles) need further validation. If that is not the focus of the manuscript, maybe the authors should clarify this to the readers. Could you explain more about this aspect?

Response:
We thank the reviewer for acknowledging the materials presented herein may beget interesting electrochemical behavior. However, this paper specifically focuses on chemical

properties not electrochemical properties, thus is outside the scope of this work. However, in a future manuscript we will outline this aspect of the materials.

R3 Comment 8:
Could the authors comment about the costs and complexity to get the carborane based compounds compared to conventional electrolyte materials?

Response:
The synthesis of the carboranes is not that difficult if you have decaborane-14. Decaborane is rather expensive but can be reliably sourced from several companies including Aldrich.

oc-2025-00043n.R2

Name: Peer Review Information for "Combustion Resistant Borohydrides and their Chemical Interactions with Li-Metal Surfaces: An Experimental and Theoretical Study"

Second Round of Reviewer Comments

Reviewer: 3

Comments to the Author

The authors answered well all the issues and now the manuscript is suitable for publication in ACS Central Science.

Author's Response to Peer Review Comments:

1. The author list has been updated to reflect the correct authors.

2. The SI page numbering has been corrected and the authors have been updated.

3. The shortened synopsis (under 200 characters with spaces) can be found at the very end of the manuscript (MS).

4. The MS abstract had a small typo in the last sentence : "but ~ but". The "~ but" was removed for clarity.

5. Our previous submissions included a short film showing the ionic liquid does not burn (included as a Web Enhanced Object) and the paper referenced "Movie S1" in it. However, we do not see the file type in "Step 3: File Upload". Therefore, we have included it again.
